# NANOG is required to establish the competence for germ-layer differentiation in the basal tetrapod axolotl

**DOI:** 10.1371/journal.pbio.3002121

**Published:** 2023-06-14

**Authors:** Luke A. Simpson, Darren Crowley, Teri Forey, Helena Acosta, Zoltan Ferjentsik, Jodie Chatfield, Alexander Payne, Benjamin S. Simpson, Catherine Redwood, James E. Dixon, Nadine Holmes, Fei Sang, Ramiro Alberio, Matthew Loose, Andrew D. Johnson

**Affiliations:** 1 School of Life Sciences, University of Nottingham, Queens Medical Centre, Nottingham, United Kingdom; 2 Tumour Immunogenomics and Immunosurveillance Laboratory, University College London Cancer Institute, London, United Kingdom; 3 Cancer Dynamics Laboratory, The Francis Crick Institute, London, United Kingdom; 4 School of Biosciences, University of Nottingham, Sutton Bonington Campus, Loughborough, United Kingdom; University of Edinburgh, UNITED KINGDOM

## Abstract

Pluripotency defines the unlimited potential of individual cells of vertebrate embryos, from which all adult somatic cells and germ cells are derived. Understanding how the programming of pluripotency evolved has been obscured in part by a lack of data from lower vertebrates; in model systems such as frogs and zebrafish, the function of the pluripotency genes NANOG and POU5F1 have diverged. Here, we investigated how the axolotl ortholog of NANOG programs pluripotency during development. Axolotl NANOG is absolutely required for gastrulation and germ-layer commitment. We show that in axolotl primitive ectoderm (animal caps; ACs) NANOG and NODAL activity, as well as the epigenetic modifying enzyme DPY30, are required for the mass deposition of H3K4me3 in pluripotent chromatin. We also demonstrate that all 3 protein activities are required for ACs to establish the competency to differentiate toward mesoderm. Our results suggest the ancient function of NANOG may be establishing the competence for lineage differentiation in early cells. These observations provide insights into embryonic development in the tetrapod ancestor from which terrestrial vertebrates evolved.

## Abstract 2

The role of Nanog and Nodal in establishing pluripotency in an amphibian, the axolotl, indicates that the gene regulatory networks underlying pluripotency are conserved and provided the basis for the evolution of mesoderm.

## Introduction

### Pluripotency across vertebrates

In mammals, pluripotency is defined as the competency of a single cell to contribute to both the soma and germ line [1–3]. Many divergent strategies have evolved throughout the animal kingdom for producing all necessary embryonic cell types. For example, many nonmammalian model organisms such as chicks, frogs, and zebrafish do not form both the soma and the germ line from a common pool of cells, instead, maternally inherited determinants segregate the germ cells. In contrast, mammals, and other amphibians such as the axolotl, induce germ cells and soma from the same cells. This mechanism of germ cell determination, known as epigenesis, appears to be basal while the mechanism observed in chicks, frogs, and zebrafish, known as preformation, appears to be derived [4–8]. It is unclear as to whether the lack of requirement for germ cell competence in animals exhibiting preformation has led to changes in the mechanisms governing pluripotency.

The pluripotency gene regulatory network (GRN) has been extensively studied in mammals in vitro. It has long been established that the transcription factor NANOG, as well as POU5F1 and SOX2 cooperate to support mammalian pluripotency [9–14]. In the mouse, NANOG itself is absolutely required for the reprogramming of somatic cells back to a pluripotent state in vitro [15]. Moreover *Nanog*-null mice fail to establish a pluripotent epiblast and die preimplantation [14]. NANOG has been implicated in a variety of roles to support pluripotency as an independent transcription factor through regulation of the epigenome [16–18]. In contrast, *Nanog* is not required for the maintenance of mouse embryonic stem cells (mESC) and *Nanog-*null *m*ESC retain their competence to form all embryonic cell types in the context of chimeras [10]. Together, this suggests that NANOG is required for the initial establishment of the pluripotent state in mammals but is not required for its maintenance.

Despite the fact that NANOG, POU5F1, and SOX2 genes appear to be highly conserved throughout vertebrates [19–22], little is known about their function in non-mammals. Axolotl have retained both *Nanog* and *Pou5f1* genes; however, the former has been lost in frogs, while the latter has been lost in zebrafish, frogs, and chicks [20,21,23]. Axolotls are therefore a useful model for studying the origins of cell determination in the tetrapod ancestor from which mammals, amphibians, and reptiles evolved [4].

Given the role of NANOG in programming pluripotency in mammalian embryos [14], we investigated the role of NANOG during early axolotl development to gain insights into the mechanisms governing pluripotency in basal tetrapods. We show that zygotically expressed NANOG is required for gastrulation. Animal caps (ACs) depleted of NANOG lack the competence to differentiate into embryonic germ layers in response to inductive activin signals. Unlike in zebrafish, we find no evidence that NANOG activates the zygotic genome. In addition, we find that NANOG-depleted embryos lack the active transcription-associated marks H3K4me3 and H3K27ac. Moreover, depletion of NODAL signalling or depletion of the epigenetic modifying enzyme DPY30 both result in a similar loss of H3K4me3 and H3K27ac and an inability to form mesoderm in response to inductive cues. This is reminiscent of findings in human ESC (hESC) whereby NANOG, SMAD2, and DPY30 form a complex to regulate H3K4me3 deposition at SMAD2 target gene loci, suggesting that this mechanism may be conserved between axolotls and humans [16].

## Results

### Nanog is required for the development of axolotl embryos

**Fig 1:**
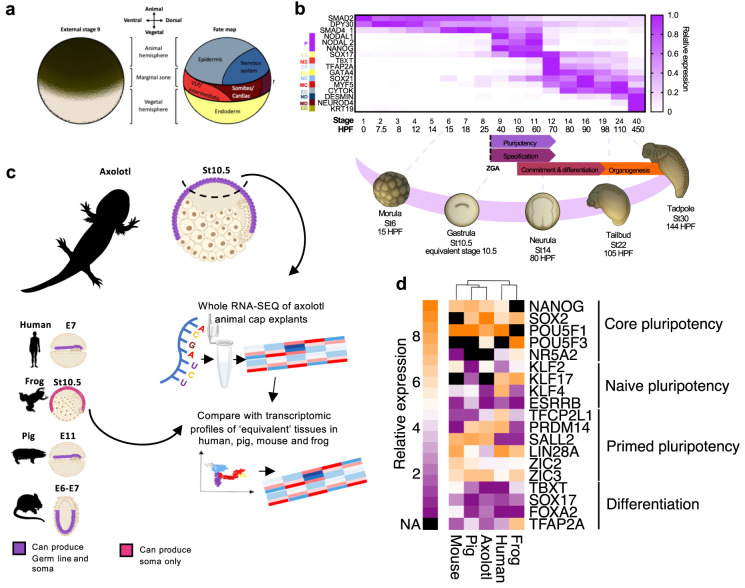
AC pluripotency resembles that of mammals and is under the control of Nanog. (**a**) Diagram showing axolotl embryo external view at stage 9, prior to gastrulation and the approximate cell fates of each region. (**b**) Overview of axolotl early development. Axolotl staging was done as described previously and in line with Bordzilovskaya and Dettlaf [13,23]. Approximate HPF at 18°C. Pluripotency (P), endoderm specification (general) (ES), mesoderm specification (general) (MS), ectoderm specification (general) (EcS), mesoderm commitment (MC), endoderm commitment (EC), ectoderm commitment (EcC), mesoderm differentiation (MD), endoderm differentiation (ED), Neural differentiation (ND). Data from Jiang and colleagues [24]. (**c**) Outline of transcriptomic data collection and cross-species comparison (for axolotl samples *n* = 3, 15 pooled explants per sample). (**d**) Key pluripotency and differentiation gene expression in the peri-gastrula primitive ectoderm of axolotl, mice, frogs, humans, and pigs. NA indicates that transcript information is not available in the source data. AC, animal cap; HPF, hours post fertilisation; ZGA, zygotic genome activation.

Stages 1 to 7 (24 hours post fertilisation; HPF) of axolotl development comprise of a series of cleavage divisions before the maternal to zygotic transition (MZT, also known as zygotic genome activation; ZGA) occurs around blastula stages 8 to 9 (approximately 30 HPF). During this period, maternal mRNAs are degraded, and the first zygotic genes are transcribed [24]. Our previous observations have shown that *Nanog* and *Pou5f1* transcripts are expressed throughout the animal hemisphere which in axolotl, gives rise to the ectoderm, neurectoderm, mesoderm, PGCs, and endoderm [8,13,25,26] (Fig 1A). *Nanog* and *Pou5f1* transcripts are detectable in the animal hemisphere from around stage 9 (approx. 40 HPF) through to stage 12 (late-gastrula, early neurula approx. 70 HPF) [23]. Transcriptomics of early axolotl development [24] show that *Nanog* transcripts appear shortly after ZGA; however, *Pou5f1* can be detected from the 1-cell stage suggesting maternal inheritance (Fig 1B, S1 Table). Transcripts of both genes sharply reduce between stages 11 to 12 (approx. 60 to 70 HPF), concomitant with the increased expression of germ-layer specification genes: *Tbxt*, *Tfap2a*, and *Gata4*, suggesting that these stages define the window of pluripotency in the axolotl.

To assess genome-wide expression profiles of axolotl pluripotent tissues, we performed RNA sequencing (RNA-seq) on axolotl ACs explanted at stage 10.5 (mid-gastrula stage; 55 HPF) (Fig 1C and 1D, S2 Table). RNA-seq revealed the pluripotency genes *Nanog* and *Pou5f1* were expressed (S2 Table), consistent with our previous observations from in situ hybridisation [23]. We compared the relative expression of several genes associated with core, naïve, and primed pluripotency integrating bulk RNA sequencing and sc-RNASeq [27–30] with peri-gastrulation *Xenopus* tropicalis AC (Stage 10.5), pig (E11), mouse (TS9-10) epiblast cells as well as peri-implantation human epiblast cells (E7). All organisms showed similar expression profiles of the core pluripotency factor *SOX2* (Fig 1D). Axolotl, pig, mouse, and human pluripotent cells showed similar expression profiles for the pluripotency genes *NANOG* and *POU5F1*, which are not encoded by the *Xenopus* genome [20,21,23]. Interestingly, *POU5F3*, which has been associated with pluripotency in *Xenopus* was highly expressed in both axolotl ACs and *Xenopus*; however, *Xenopus* pluripotency factor *NR5A2* was not expressed highly in any of the organisms investigated. *PRDM14* was highly expressed in axolotl, pig, and human but not mouse or frog. Other pluripotency genes, such as *LIN28A* showed similar expression between all organisms apart from axolotl, human-axolotl, and pig-axolotl, respectively. ZIC3 was highly expressed in all 5 of the organisms while differentiation genes were lowly expressed, consistent with the notion that the stages represent a later pluripotent state. Our observations suggest that all 5 organisms share a common set of genes associated with increased cellular potency; however, some genes relative expression show greater conservation than others such as *NANOG*, *POU5F1*, and *ZIC3*.

Mouse pluripotent cells are classified as either naïve or primed, defined by their developmental potential and their dependence on *Nodal* signalling [3]. In axolotl, both *Nodal* and *Nanog* genes are transcribed between stages 8 and 9 (blastula and late blastula, 30 and 40 HPF, respectively) in the AC following ZGA [13,23] (Fig 1B). Moreover, there is no stage at which *Nanog* is expressed in the absence of *Nodal*, suggesting that their co-expression represents basal pluripotency.

**Fig 2:**
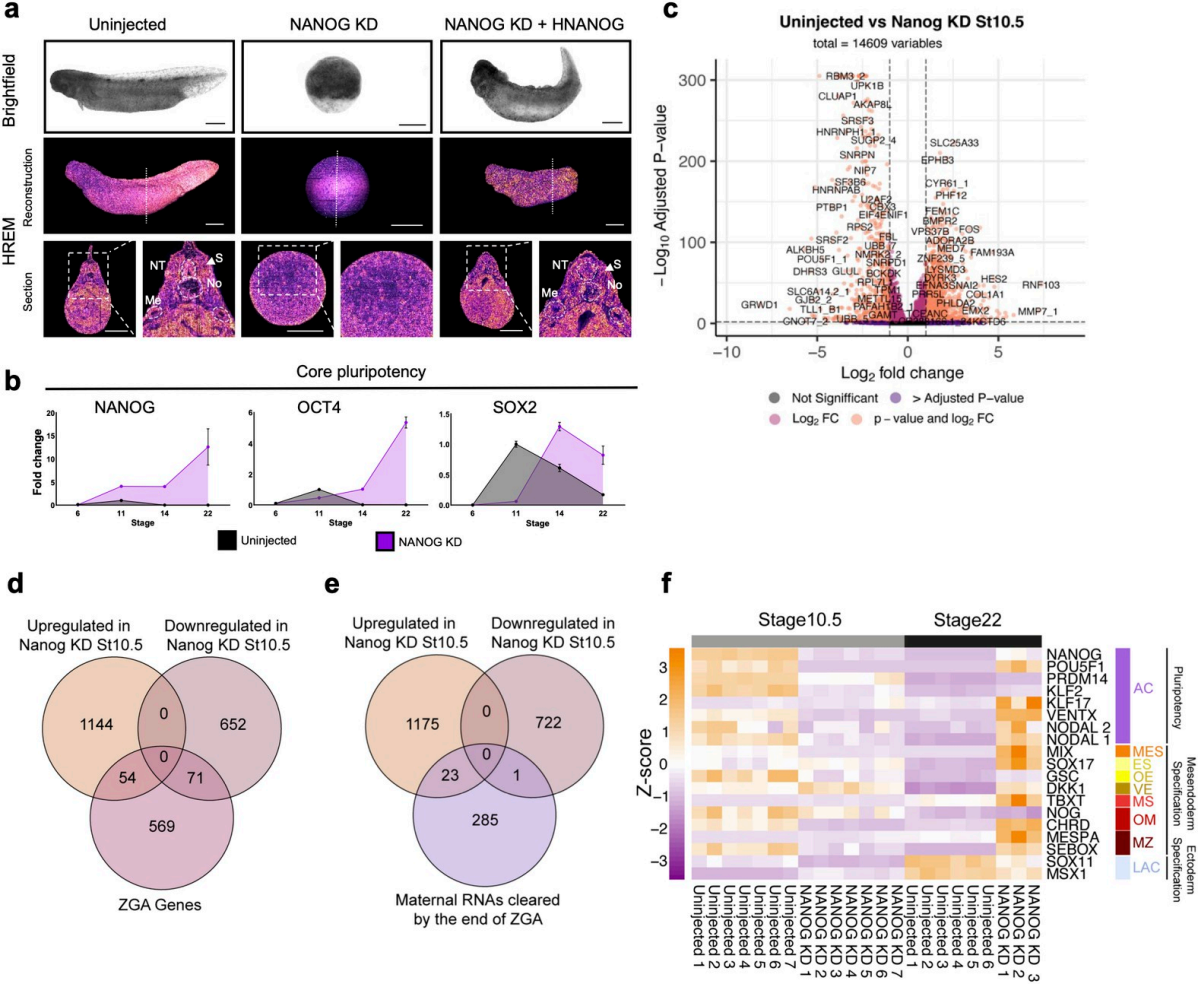
Nanog is required for gastrulation. (**a**) Brightfield images and HREM reconstructions of uninjected and NANOG translation MO depleted and hNANOG rescued embryos at equivalent stage 40. Dotted line marks plane of section reconstruction. Dashed lines highlight: somites (S), neural tube (NT) notochord (No), mesonephric ducts (Me). Scale bar, 1 mm. (**b**) Expression of core pluripotency genes at different developmental stages with and without NANOG translation MO KD (*n* = 3, 10 pooled embryos per experimental condition). (**c**) Volcano plot showing significant DEGs in NANOG translation MO KD embryos compared to equivalent stage 10.5 uninjected embryos. Vertical dotted line indicates a Log2 fold change of 1.5. Horizontal line indicates padj threshold of 0.01 on an -log10 scale. Orange points indicate significantly DEGs. Expression of core pluripotency genes at different developmental stages with and without NANOG translation MO KD. (**d**) The number of maternally inherited genes, usually cleared by ZGA, which are differentially expressed (>1 Log2 FC, padj < 0.01) in NANOG translation MO KD at equivalent stage 10.5. (**e**) The number of zygotically genome activation-associated genes, which are differentially expressed (>1 Log2 FC, padj < 0.01) in NANOG translation MO KD at equivalent stage 10.5. (**f**) Z-score heatmap indicating relative gene expression of cell type marker genes at equivalent stage 10.5 and 22 in uninjected (*n* = 7 and 6, respectively) and NANOG translation MO depleted (*n* = 7 and 3, respectively) embryos. Animal cap (AC), mesendoderm specification (general) (MES), endoderm specification (general) (ES), organiser endoderm (OE), vegetal endoderm (VE), mesoderm specification (general) (MS), organiser mesoderm (OM), marginal zone (MZ), late animal cap (fated to ectoderm; LAC). The data underlying this figure are available in S1 Images and in S1 Data. FC, fold change; DEG, differentially expressed gene; HREM, high-resolution episcopic microscopy; St, stage; ZGA, zygotic genome activation.

To understand the role of the zygotic NANOG protein in axolotl development, we employed a knockdown approach. Injection of translation inhibiting antisense morpholinos (MO) at the 1-cell stage directed against NANOG resulted in the complete arrest of development prior to the onset of gastrulation (Figs 2A and S1A–S1D). Western blotting confirmed that at a stage equivalent to 10.5 in uninjected sibling embryos (55 HPF), the NANOG protein was undetectable (S1B Fig). Moreover, splice blocking morpholinos also produced an identical phenotype (S1C Fig). Gastrulation could be rescued in NANOG knockdowns (KD) by co-injection of 100 pg human *NANOG* (*hNANOG*) RNA at the 1-cell stage (Figs 2A and S1D), demonstrating both the specificity of the morpholinos effects and that *hNANOG* can functionally substitute for axolotl NANOG. High-resolution episcopic microscopy (HREM) of NANOG morphants showed no evidence of either involution or ingression, which characterise axolotl gastrulation [31] (Figs 2A and S1E). Morphant embryos maintained this arrested morphology even as sibling control embryos developed beyond stage 40 (Tadpole, 450 HPF). This phenotype was somewhat unexpected given that *Xenopus KD* of *Ventx1/2*, which has been proposed to be functionally equivalent to NANOG, does complete gastrulation and displays a truncation along the A-P axis [32]. Phenotypically, axolotl NANOG morphants show a greater resemblance to NANOG-null zebrafish, which similarly fail to gastrulate [33,34]. We hypothesised that axolotl NANOG morphants might be unable to gastrulate due to a premature loss of pluripotency and premature differentiation. However, gene expression analysis of NANOG morphants across multiple time points shows persistent expression of *Nanog*, *Pou5f1*, and *Sox2 mRNAs* long after uninjected siblings completed gastrulation and reached stage 22 (Tailbud, 105 HPF), indicating that pluripotency gene transcription is not lost (Fig 2B).

### Germ-layer commitment requires NANOG activity

**Fig 3:**
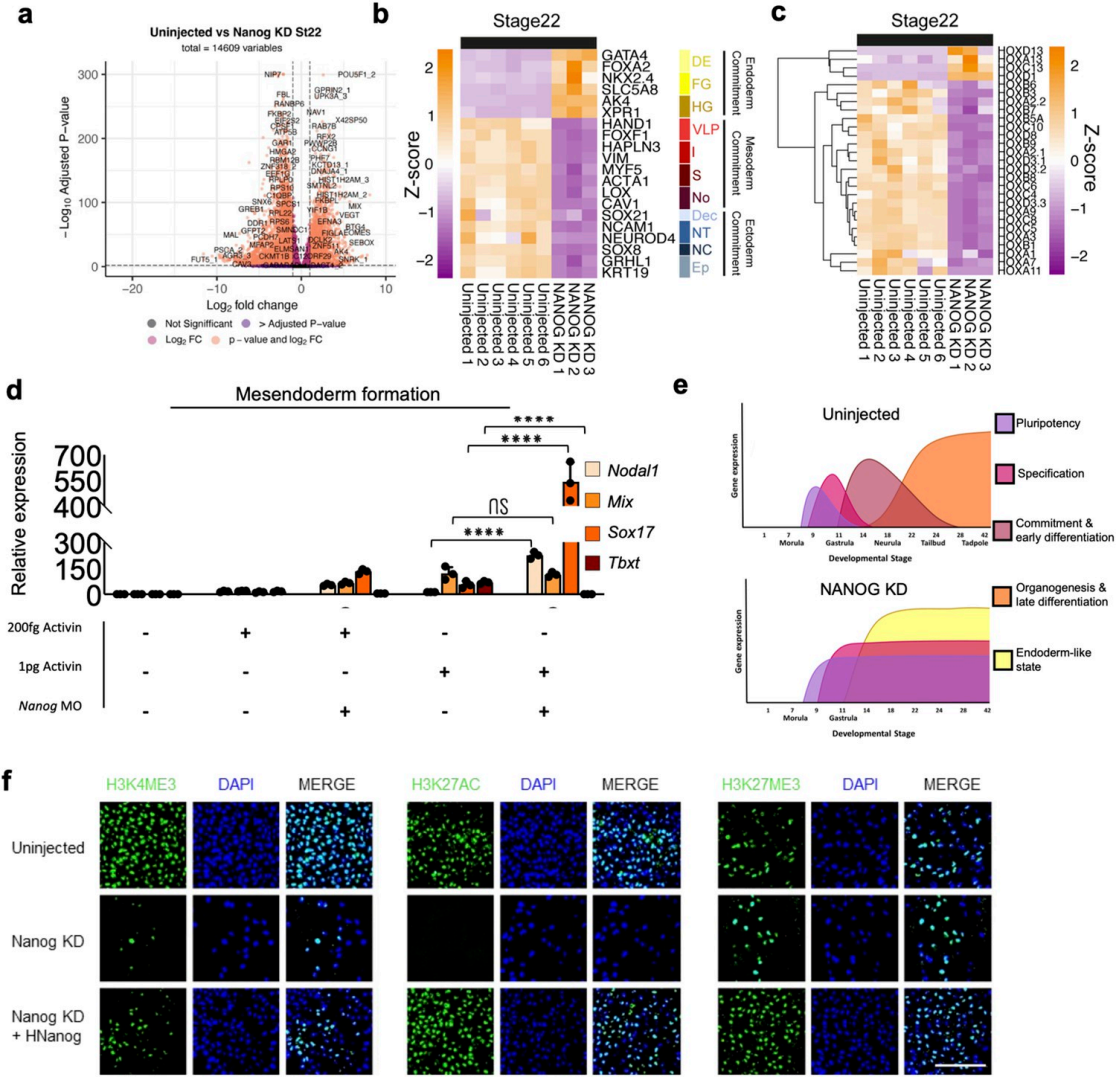
Nanog is required for differentiation in response to inductive signals. (**a**) Volcano plot showing significant DEGs in NANOG translation MO KD embryos compared to equivalent stage 22 uninjected embryos. Vertical dotted line indicates a fold change 1 Log2. Horizontal line indicates padj threshold of 0.01 on an -log10 scale. Orange points indicate significantly DEGs. (**b**) Z-score heatmaps indicating relative gene expression of cell type marker genes at stage 22 uninjected (*n* = 6) and at equivalent stage NANOG translation MO-depleted (*n* = 3) embryos. Definitive endoderm (general) (DE), foregut (FG), hindgut (HG), ventral-lateral plate (VLP), intermediate mesoderm (I), somite (S), notochord (No), definitive ectoderm (general) (Dec), neural tube (NT), neural crest (NC), epidermal progenitors (EP). (**c**) Expression of Hox genes in response to NANOG translation MO KD in equivalent stage 22 embryos. (**d**) QPCR of germ-layer markers of uninjected and NANOG translation MO-depleted stage 22 equivalent caps following treatment with different activin concentrations (*n* = 15). Asterisks represent the adjusted *p*-value obtained from Tukey’s multiple comparisons test following one-way ANOVA, * = *P* ≤ 0.05, ** = = *P* ≤ 0.01, *** = *P* ≤ 0.001, *** = *P* ≤ 0.001, **** = *P* ≤ 0.0001, ns = *P* > 0.05. (**e**) Diagrammatic representation of the effect of NANOG translation MO depletion, which prevents sequential waves of gene expression post specification. (**f**) Uninjected, NANOG translation MO depleted and hNANOG rescued AC explants cultured to equivalent stage 10.5 and stained for H3K4me3, H3K27ac, H3K27me3, and DAPI (*n* = 3). Scale bar, 60 μm. The data underlying this figure are available in S1 Images and in S1 Data. AC, animal cap; DEG, differentially expressed gene; ns, non-significant.

To better characterise NANOG loss of function (LOF), we transcriptionally profiled uninjected embryos at stages 10.5 and 22 alongside matched sibling NANOG KD embryos (approximately 55 HPF and 105 HPF; Figs 2A–2F and 3A–3C and S2A–S2K). At equivalent stage 10.5, 1,198 genes showed significant up-regulation (>1 Log2 Fold change; FC, FDR <0.01) and 708 genes were significantly down-regulated (< -1 Log2 FC, FDR <0.01) (Fig 2C, S3 and S4 Tables) in the NANOG KD embryos. Gene Ontology enrichment analysis of NANOG morphant transcriptomes showed significant positive enrichment (FDR <0.02) for numerous gene sets, including DNA-binding transcription activator activity, sequence-specific double-stranded DNA binding, *cis*-regulatory region sequence-specific binding, and negatively enriched for single-stranded RNA binding and methyltransferase activity (S2A Fig and S5 Table). KEGG pathway analysis also showed significant (FDR <0.01) positive and negative enrichment of a diverse range of pathways (S2B Fig and S6 Table). This suggests that the NANOG LOF phenotype has wide-ranging effects on the transcriptional machinery that coordinate development. It has been suggested that NANOG LOF in zebrafish results in improper ZGA [33]. While many genes were differentially expressed in NANOG KD embryos at equivalent stage 10.5, we detected 11,753 genes with a TPM greater than 1 compared to 11,462 genes in the uninjected controls (S3 Table). ZGA in axolotl takes place between stage 8 and 9 [24] (25 and 40 HPF). We identified which genes were exclusively expressed >1 TPM after stage 7 (18 HPF) and before stage 10 (50 HPF) (S7 Table). Of 694 “ZGA genes,” 54 were significantly (FDR <0.01) up-regulated and 71 were down-regulated in NANOG KD embryos at a time point equivalent to stage 10.5 (55 HPF) (Fig 2D). We also asked if maternal clearance of RNA was affected by NANOG KD. We identified a total of 309 “maternally inherited” RNAs that were cleared by the end of ZGA at stage 9 (40 HPF) in uninjected embryos (S8 Table). Of these genes, only 23 were significantly (FDR <0.01) up-regulated, and 1 gene was down-regulated (Fig 2E). Together, this suggests that while NANOG may have some role in the regulation of nascent zygotic genes, but ZGA itself is largely unaffected by NANOG KD in the axolotl.

Given that pluripotency is defined by the competence of cells to form mature cell types, we next investigated the expression of germ-layer specification and commitment genes in our transcriptomic data. To rule out delayed lineage commitment or lineage commitment in the absence of the morphogenic movements of gastrulation, we investigated an uninjected morphant gene expression at equivalent stages 10.5 and 22 (55 and 105 HPF). At stage 10.5 transcription of pluripotency markers *Nanog*, *Pou5f1*, *Prdm14*, and *Klf2* were significantly down-regulated in NANOG-depleted embryos (FDR <0.01). By stage 22 however, many pluripotency and mesendoderm specification genes which are normally down-regulated by this stage [24], such as *Mix*, *Sox17*, *Tbxt*, *Chrd*, and *Mespa* showed expression levels in morphants similar to, or higher than, those of uninjected embryos at stage 10.5 (Figs 2F and S1G). This suggests that NANOG may be required to progress beyond early lineage specification. Accordingly, genes involved in mesoderm or ectoderm commitment, including several members of the *Hox* gene family, were significantly (FDR <0.01) down-regulated in morphants compared to stage 22 controls. In contrast, endodermal commitment genes such as *Gata4*, *FoxA2*, *Ak4*, and *Xpr1* showed significantly increased transcription compared to uninjected embryos (Fig 3A–3C and S4 Table). Gene set enrichment analysis (GSEA) using amphibian cell type marker gene sets [35] (S3A and S3B Fig) showed enrichment of lateral plate, notochord, cardiac mesoderm, neural tube, neural crest, and epidermal progenitor marker gene sets among the significantly (FDR <0.01) down-regulated genes in NANOG morphants. Moreover, morphants showed loss of late differentiation markers compared to stage 28 (144 HPF) controls (S1H Fig), suggesting NANOG depletion causes embryos to form endoderm-like tissue that does not fully differentiate.

Together, these data suggest that NANOG LOF has little effect on ZGA but is required for gastrulation. The NANOG LOF phenotype appears to affect multiple pathways relating to transcription and translational regulation. Curiously though NANOG morphants fail to commit to germ layers or form mature cell types in the absence of the morphogenic movements of gastrulation despite expressing many early lineage specification genes including master regulators of gastrulation. NANOG is therefore required to coordinate lineage specification and commitment in the cells of the animal hemisphere. Depletion, therefore, prevents the subsequent waves of gene expression that would normally drive embryogenesis and cell maturation (Fig 3E).

### NANOG-depleted ACs cannot respond properly to inductive signals

To address if NANOG LOF resulted in a lack of competency to form individual germ layers or simply a lack of proper differentiation cues, we tested the developmental potential of NANOG*-*depleted AC directly using explants (Figs 3D and S4A–S4F). Explanted caps normally differentiate into epidermis, marked by increased expression of *Grhl1* and *Cytok*, and down-regulation of *Nanog* and *Pou5f1* transcripts. Injection of *Activin A* (*Activin*) mRNAs at the 1-cell stage can divert the fate of these cells to other lineages including mesoderm, endoderm, or PGCs in a dose-dependent manner [8,13]. We have previously demonstrated that 1 pg of RNA encoding activin injected at the 1-cell stage induces mesoderm, marked by elongation of the animal cap explant and *Tbxt* expression [13]. By titration, we established that 200 fg of RNA is insufficient to divert caps from an epidermal fate to mesoderm (Figs 3D and S4D). Higher levels of activin (100 pg) are required to drive caps to endoderm as marked by *Sox17* expression and a lack of elongation. We utilised this assay to directly test the ability of NANOG-depleted cells to form tissues. Morphant caps with no exogenous *Activin* failed to produce epidermis and maintained high expression of *Nanog* and *Pou5f1 mRNA* levels (S4B Fig). NANOG-depleted caps injected with sub-mesodermal (200 fg) doses of activin showed a large increase in *Sox17*, but not *Tbxt* expression, and 1 pg of activin also induced high levels of endoderm marker *Sox17*, also without induction of *Tbxt* (Fig 3D). These data suggest that AC’s depleted of NANOG are hypersensitised to TGF-ß signalling.

In amphibians, the foregut derives from organiser endoderm that originates in the animal hemisphere of the embryo, while the hindgut is produced from the yolky cells of the vegetal hemisphere [35–38]. Morphant caps injected with 100 pg of exogenous activin at the 1-cell stage did not show increased expression of foregut marker *Nkx2*.*6* or hindgut marker *Hnf4g* over uninjected controls, though these caps did express *C8b*, an endoderm commitment marker expressed post gastrulation (S4E Fig). This suggests that in the absence of NANOG, the cells of the animal hemisphere arrest endoderm development at the progenitor stage.

Together, these data suggest that NANOG-depleted AC’s cannot produce mesoderm even in the presence exogenous ACTIVIN signalling. While NANOG KD AC are able to activate some early markers of definitive endoderm, we do not find any evidence of more mature endodermal subtypes. Moreover, NANOG-depleted caps appear to hypersensitised to TGF-ß signalling. The vegetal pole cells of axolotl appear to be NANOG-independent we surmise, therefore that hindgut endoderm expression in morphant embryos is derived from the vegetal pole, which does not express *Nanog* mRNA [23], while anterior foregut endoderm, as well as mesoderm and ectoderm development from animal hemisphere is arrested by NANOG depletion in line with *Nanog’s* expression profile.

### NANOG and NODAL activities are required for gastrulation in axolotl

In hESC, NANOG synergises with NODAL signals to deposit H3K4me3 on promoters of transcriptionally active genes [16]. We investigated if there is any evidence for this mechanism in axolotl ACs. We performed immunostaining in stage 10.5 uninjected embryos and equivalent stage NANOG KD embryo AC explants using antibodies for H3K4me3, H3K27ac, and H3K27me3. While all 3 marks are readily detectable in uninjected nuclei (Fig 3F), expression of H3K4me3 was greatly reduced in NANOG morphant caps and H3K27Ac was not detectable. H3K27me3, by contrast, was unaffected. H3K4me3 and H3K27Ac were rescued at stage 10.5 when NANOG MO was co-injected with *hNANOG* RNA (100 pg) at the 1-cell stage. We also showed that native H3, H3K36me3, and phospho-pol II were unaffected by NANOG depletion (S4A–S4C Fig).

These findings show that NANOG morphant ACs are transcriptionally active, but morphant transcription is not sufficient to drive embryogenesis beyond the blastula stage. The loss of the H3K4me3 is reminiscent of hESC in which NANOG maintains active transcription marks in pluripotent chromatin [16].

**Fig 4:**
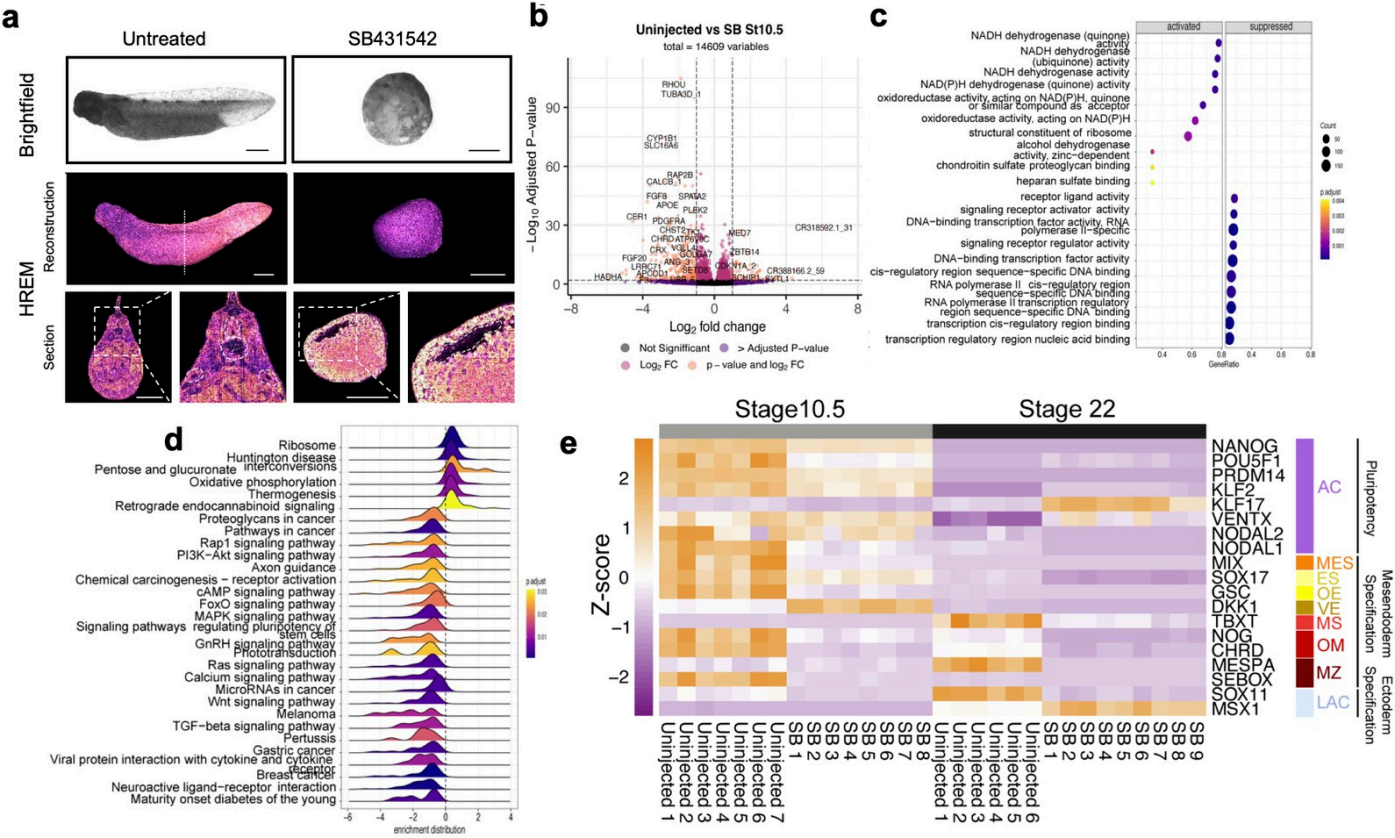
Nodal signalling programs early germ-layer specification. (**a**) Brightfield and HREM images of stage 40 uninjected and equivalent stage SB431542-treated embryos. Dotted line marks plane of section reconstruction. Somites (S), neural tube (NT) Notochord (No), Mesonephric ducts (Me), Blastocoel (B) (*n* = 25 and 2, respectively). Scale bar, 1 mm. (**b**) Volcano plots showing significant DEGs in SB-treated embryos compared to equivalent stage 10.5 uninjected embryos. Vertical dotted line indicates a fold change 1 Log2. Horizontal line indicates padj threshold of 0.01 on an -log10 scale. Orange points indicate significantly DEGs. (**c**) Dot plots showing significantly enriched (padj <0.01) GO database biological process gene sets within SB-treated DEGs at equivalent stage 10.5. (**d**) Ridgeline plots showing significantly enriched (padj <0.01) KEGG pathway gene sets within SB-treated DEGs at equivalent stage 10.5. (**e**) Z-score heatmap indicating relative gene expression of cell type marker genes at stages equivalent to 10.5 and 22 in uninjected (*n* = 7 and 6, respectively) and SB431542 (*n* = 8 and 9, respectively) treated embryos. Animal cap (AC), mesendoderm specification (general) (MES), endoderm specification (general) (ES), organiser endoderm (OE), vegetal endoderm (VE), mesoderm specification (general) (MS), organiser mesoderm (OM), marginal zone (MZ), late animal cap (fated to ectoderm; LAC). The data underlying this figure are available in S1 Images and in S1 Data. DEG, differentially expressed gene; HREM, high-resolution episcopic microscopy.

**Fig 5:**
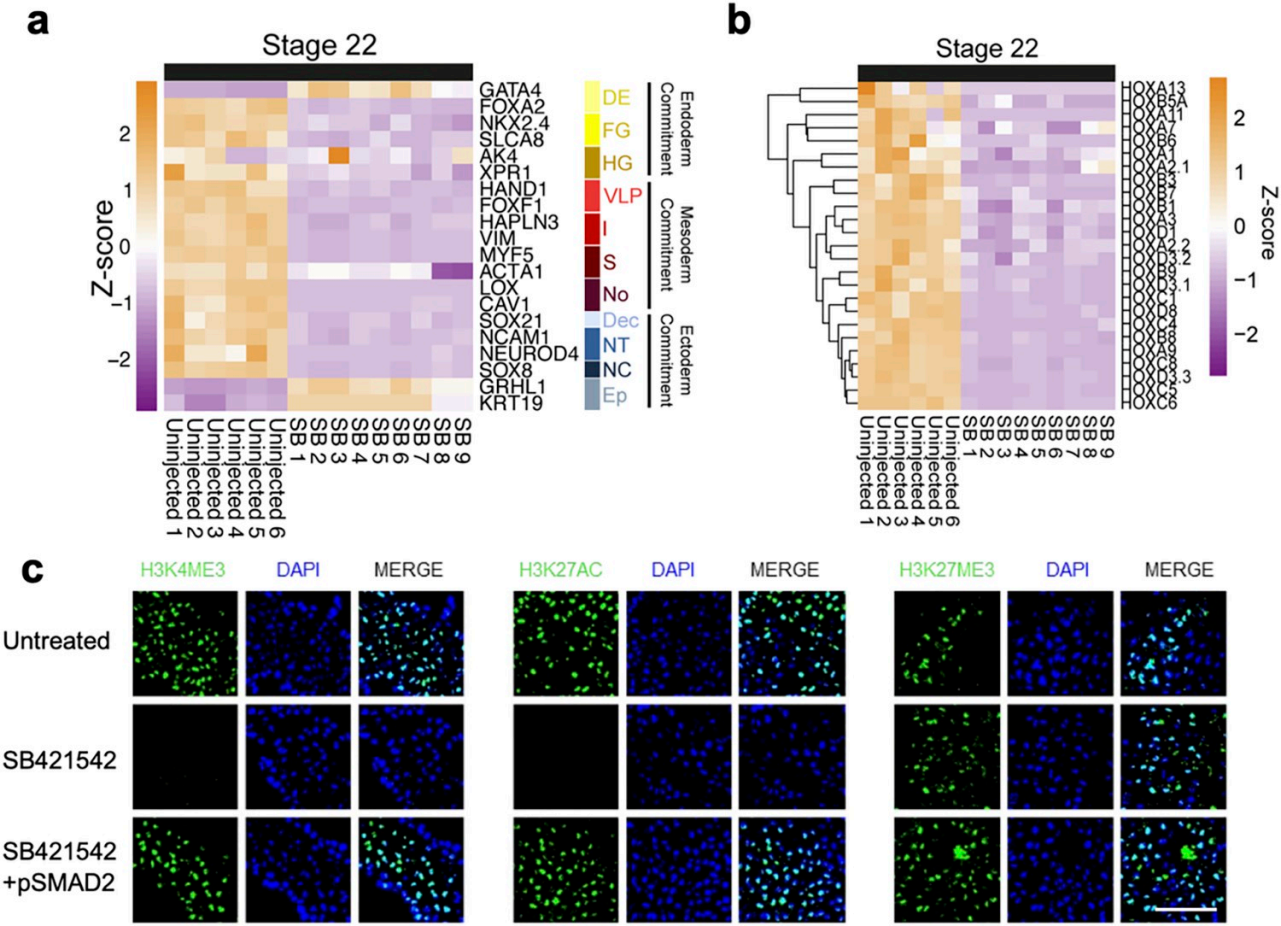
Nodal signal depletion prevents germ-layer commitment and depletes activating epigenetic marks. (**a**) Z-score heatmap indicating relative gene expression of cell type marker genes in SB431542-treated embryos at a stage equivalent to 22 in uninjected (*n* = 6 and 9, respectively). Definitive endoderm (general) (DE), foregut (FG), hindgut (HG), ventral-lateral plate (VLP), intermediate mesoderm (I), somite (S), notochord (No), definitive ectoderm (general) (Dec), neural tube (NT), neural crest (NC), epidermal progenitors (EP). (**b**) Gene expression of Hox genes in SB431542-treated embryos at equivalent stage 22. (**c**) Uninjected, SB431542-treated, and SMAD2 rescued AC explants cultured to stage equivalent to 10.5 and stained for H3K4me3, H3K27ac, H3K27me3, and DAPI (*n* = 3). Scale bar, 60 μm. The data underlying this figure are available in S1 Images and in S1 Data. AC, animal cap.

NANOG’s epigenetic regulation in hESC depends on interaction with SMAD2/3, downstream of active NODAL signalling [16]; indeed, NODAL signalling itself promotes pluripotency in hESC. Because of this, we investigated whether NODAL signalling functions in pluripotent ACs. We have previously shown axolotl mesoderm induction is mediated by ACTIVIN*/*NODAL signalling and that axolotls share a conserved mesoderm GRN with mammals [13]. NODAL inhibition, either by morpholino or SB431542 (SB), results in a phenotype resembling the axolotl NANOG morphant, arresting development prior to gastrulation [13]. We reconfirmed this observation using HREM demonstrating the absence of morphological development following SB exposure (Fig 4A). Transcriptomes of stage 10.5 equivalent time point SB-treated embryos and GSEA using the GO biological process database showed that SB-treated embryos were significantly (FDR <0.0001) negatively enriched for receptor ligand activity, signalling receptor activity, and DNA-binding transcription activity gene sets (Fig 4B and 4C). GSEA of the same data using the KEGG database showed significant (FDR <0.001) negative enrichment of the TGF-ß signalling pathway, Wnt signalling pathway, and signalling pathways regulating pluripotency of stem cells gene sets (Fig 4D). As expected, SB embryos showed significant down-regulation of mesendodermal specification genes (Figs 4E and S7A). However, NODAL inhibition, while completely blocking mesendoderm specification, had no significant effect on pluripotency gene expression, in contrast to NANOG KD. Like NANOG morphants, however, stage 22 equivalent-time point SB-treated embryos lacked mesodermal commitment markers, as well as neurectoderm markers, confirmed by GSEA using amphibian cell type marker gene sets [35] (Figs 5A and S6–S8). At equivalent stage 22, SB up-regulated genes were enriched for markers of epidermal cell types. Interestingly, like NANOG depletion, markers of foregut were down-regulated, but we did not observe an effect on definitive endoderm, nor hindgut markers (Figs 5A and 5B and S6–S8). This is consistent with *Nodal* expression being confined to the animal hemisphere [13] and suggests that hindgut progenitor commitment is also independent of ACTIVIN/NODAL signalling. Indeed, vegetal pole explants treated with SB and analysed for endodermal gene expression showed that expression was unaffected by SB (S7D Fig), indicating that hindgut-like cells form independent of NODAL signalling.

Interestingly, endoderm is hypothesised to be the most ancient metazoan germ layer [39,40], and may have predated the evolution of NODAL signalling [41] and the NANOG gene [19,32,42], suggesting that vegetal pole endoderm may reflect an ancient metazoan state. Together, these data reinforce that NODAL signalling is required for differentiation of AC to mesendodermal fates.

### NANOG, NODAL, and DPY30 remodel the epigenome post-ZGA

We next asked if the effects of SB treatment could affect the epigenetic landscape of developing embryos [16]. Immunostaining confirmed that NODAL signal inhibition also results in a widespread loss of H3K4me3 and H3K27ac at 55 HPF but does not reduce H3K27me3 (Fig 5C), similar to NANOG depletion. Injection of 1 pg of mRNA encoding a constitutively active (signalling independent) SMAD2 variant [43] at the 1-cell stage was able to rescue H3K4me3 and H3K27ac in SB-treated caps. However, this was insufficient to rescue the development of SB-treated embryos. Given the phenotypic similarities between NANOG depletion and SB treatment, this may suggest NANOG and SMAD2 regulate a common set of genes via an epigenetic mechanism as proposed in hESC [16].

Given the lack of available antibodies for use in axolotl, we performed a luciferase complementation to test if the axolotl NANOG can physically interact with SMAD2. Indeed, transient expression of NANOG and SMAD2 proteins, each with a luciferase subunit fusion protein, produced a signal far above that of the background proteins suggesting the 2 proteins can interact (S7E Fig).

Given that both NANOG and NODAL signal depletion resulted in a failure to properly form germ layers or establish H3K4me3 and H3K27ac on the genome, we investigated the role of the epigenetic modifying enzyme DPY30 (S9–S12 Figs). DPY30 catalyses tri-methylation of H3K4 residues as part of the complex with NANOG and SMAD2/3 in hESC [16]. Unlike the NANOG KD or SB phenotypes, however, DPY30 KD embryos arrested post-gastrulation (S9A and S9B Fig). HREM showed these morphants lacked a notochord, somites, or other mesodermal structures, had a multilayered AC and rudiments of a neural tube. Embryos resembling wild type were rescued with 95% efficiency by co-injection of 200 pg RNA encoding human DPY30 with the morpholino at the 1-cell stage, demonstrating specificity and high conservation of function. Moreover, H3K4me3 became undetectable in ACs after DPY30 depletion, and H3K27ac was also diminished, shown by immunostaining (S11E Fig).

Given the singular H3K4 tri-methyltransferase activity of DPY30, these data suggest that loss of H3K27ac may be an indirect effect of DPY30 depletion. Transcriptomes confirmed that stage 10.5 DPY30 morphants had significantly reduced expression of the pluripotency factor *Pou5f1* and lineage specification genes *Mix*, *Sox17*, and *Tbxt* at stage 10.5 but failed to transcriptionally silence these genes (S9C and S10 Figs), also resembling NANOG morphants. DPY30 LOF also resulted in a significant reduction in expression of mesodermal and foregut endoderm markers and reduced *Hox* gene expression at a stage equivalent to 22 in the uninjected embryos (FDR <0.01; S9D and S9E and S10 Figs and S4 Table). Hindgut endoderm and neural plate markers were not, however, down-regulated. GSEA using cell type marker gene sets confirmed that genes down-regulated in response to DPY30 KD were significantly enriched for mesodermal tissue markers (FDR <0.01) (S12A and S12B Fig). By contrast, the most up-regulated genes were enriched for blastula and organiser endoderm markers. Additionally, markers of late mesodermal differentiation such as *Myf5*, *Hand1*, and *Vim* were also down-regulated in the morphants (S9D and S11A Figs), while endoderm and ectoderm markers were much less affected.

These results show that loss of DPY30/H3K4me3 alone is sufficient to prevent mesodermal formation. Indeed, if there is a co-operative role between NANOG, SMAD2, and DPY30 in axolotl as proposed in hESC [16], in which the factors regulate the epigenome, this is not required for the morphogenic movements of gastrulation but rather for the proper formation of cell types, particularly mesoderm. This result is also consistent with observations in mammals which show different phenotypes between NANOG, DPY30, and NODAL signal depletion [14,16,44,45]. Like axolotl, mouse embryos depleted of DPY30 also show neural development in the absence of mesoderm [16], suggesting morphological consequences of DPY30 KD are similar in axolotl and mammals.

**Fig 6:**
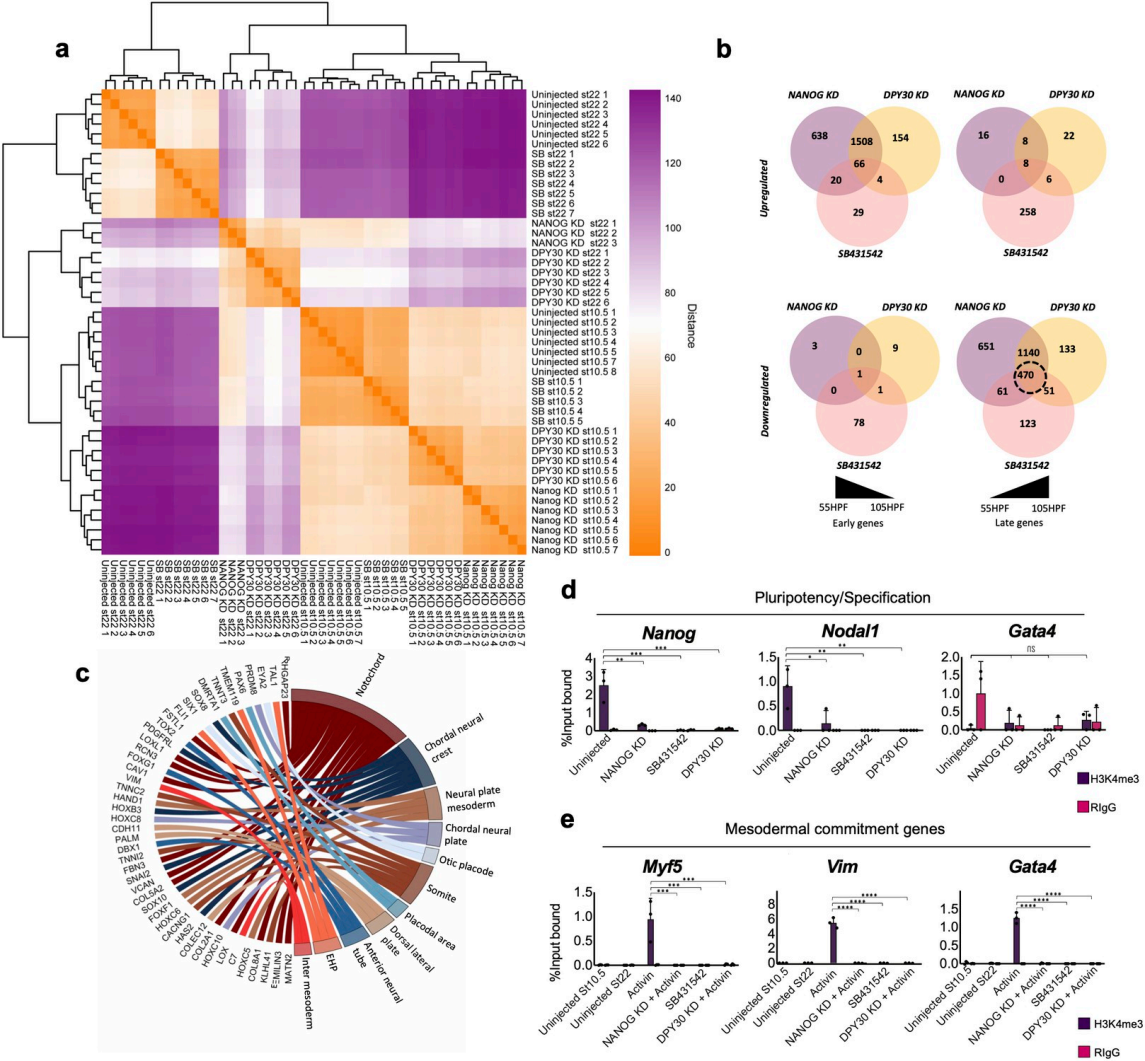
NANOG, SMAD2, and DPY30 gene target loci lack H3K4me3. (**a**) Heatmap shows sample-sample distance, orange colouring indicates a greater similarity, purple colouring indicates a greater difference in sample transcriptomes, hierarchal clustering has been applied to heatmap rows and columns. (**b**) Venn diagrams of unique and overlapping DEGs in NANOG translation MO, DPY30 translation MO, and NODAL-depleted embryos at stages equivalent to 10.5 and 22 in controls. (**c**) Chord diagram showing significantly (FDR <0.01) enriched amphibian cell type-specific markers in overlapping down-regulated genes. (**d**) H3K4me3 ChIP of equivalent stage 10.5 uninjected, NANOG translation MO KD, DPY30 translation MO KD, and SB431542-treated caps followed by qPCR using probes directed at gene promoter regions (50 pooled explants per experimental condition, individual points show technical replicates). Dots show individual data points. Asterisks represent the adjusted *p*-value obtained from Tukey’s multiple comparisons test following one-way ANOVA, * = *P* ≤ 0.05, ** = = *P* ≤ 0.01, *** = *P* ≤ 0.001, *** = *P* ≤ 0.001, **** = *P* ≤ 0.0001, ns = *P* > 0.05. (**e**) H3K4me3 ChIP-qPCR of equivalent stages 10.5 and 22, uninjected caps, stage 22 Activin-treated caps with and without NANOG translation MO and DPY30 translation MO depletion as well as SB431542-treated caps (50 pooled explants per experimental condition, individual points show technical replicates). Statistics are as described in d. The data underlying this figure are available in S1 Data. DEG, differentially expressed gene; HPF, hours post fertilization; ns, non-significant.

To test for overlap between the effects of NODAL signalling inhibition, or depletion of NANOG or DPY30, we compared transcriptomes from embryos under each treatment regime (Fig 6A–6C). We identified several groups of overlapping and nonoverlapping gene targets, consistent with the notion that NANOG, SMAD2, and DPY30 may have overlapping and separate functions. We identified 1,508 “early activated” genes whose expression is usually reduced between stages 10.5 and 22 that are significantly up-regulated in NANOG and DPY30 morphants, including pluripotency and early specification genes. We also observed overlap in genes normally up-regulated between stages 10.5 and 22 that are instead down-regulated by each treatment. GSEA showed down-regulated genes were significantly enriched for markers of mesoderm and neural tissues (Fig 6C). Remarkably, however, while mesendodermal specification genes are down-regulated by NODAL signalling inhibition, they are up-regulated by NANOG or DPY30 KD at later stages, suggesting SMAD2/3 can act independent of interaction with NANOG in early specification events. These transcriptional effects were then correlated with changes in H3K4me3 levels using ChIP-qPCR (Figs 4D, 4E and S13). ChIP revealed H3K4me3 levels on promoters of *Eef1a1* or *Krt19 (Cytokeratin)* were unaffected in stage 10.5 equivalent caps under each experimental condition (*P* > 0.05; S13A Fig), suggesting expression is independent of NANOG/SMAD2/DPY30. However, H3K4me3 was reduced under all 3 conditions on the *Nanog* and *Nodal1* promoters (*P* < 0.05; Fig 6D).

We then tested the effects of each experimental regime on mesoderm induction by performing ChIP on stage 22 equivalent caps that had also been co-injected with 1 pg of activin RNA along with either the NANOG or *DPY30* MO. H3K4me3 levels were then compared with caps at the same stage that were untreated or exposed to SB. In addition, we included stage 10.5 equivalent untreated caps to determine if the H3K4me3 mark is induced by *Activin* or laid down prior to mesoderm inducing signals (Figs 6E and S13B). ChIP revealed that activin induced deposition of H3K4me3 on promoters of mesodermal commitment genes *Myf5* and *Vim*, and this was prevented by either NANOG or DPY30 KD (*P* > 0.001). This mark was also absent in uninjected and SB-treated ACs correlating with the absence of expression. Both *Nodal1* and *Gata4* promoters also show reduced enrichment of H3K4me3 in response to depletion of NANOG and DPY30 (*P* > 0.0001; Figs 6E and S13B). Like uninjected caps, those treated with SB also showed no enrichment of H3K4me3 at *Nodal1* and *Gata4* promoters. While expression of these genes is not present in uninjected or SB-treated caps, they were activated after either NANOG or DPY30 depletion, and their expression was not extinguished post-gastrulation. Together, this suggests that H3K4me3 is lost at the promoters of several SMAD2 gene targets when either NANOG, DPY30, or NODAL signalling is depleted but that the loss of the mark post-activation does not prevent subsequent transcription absolutely.

## Discussion

Despite Nanog’s requirement for the establishment of the pluripotent state [14,15] and strong sequence conservation across vertebrates [19], little is known about Nanog’s function in development outside of mammals. Here, we set out to investigate the role of NANOG in axolotl development. We find that NANOG is absolutely required for morphogenic movements of gastrulation. In the absence of NANOG, embryos fail to activate numerous ectodermal or mesodermal commitment markers. In addition, NANOG-depleted AC explants are unable to form mesoderm in response to *Activin*; this suggests that in whole embryos depleted of NANOG the lack of germ-layer differentiation is due not to an absence of differentiation cues but rather they lack the competence to properly respond to inductive signals. NANOG morphants also displayed loss of H3K4Me3 and H3K27ac. Further investigations revealed many overlapping effects between the depletion of NANOG, DPY30, and NODAL signalling on gene transcription. We observed that while SB treatment does not directly reduce the expression of pluripotency genes, SB treatment results in negative enrichment of TGFß pathway members, and signalling pathways known to regulate the pluripotency of stem cells, including MAPK pathway and WNT signalling pathways. NODAL signal depletion also resulted in a failure to form mesodermal cell derivatives or establish global H3K4me3 and H3K27ac. Given that depletion of the methyltransferase DPY30 was also sufficient to perturb the formation of mesoderm both in whole embryos and in response to activin in ex vivo explants, this suggests the acquisition of H3K4me3 may convey the competency for mesodermal differentiation in pluripotent cells. Moreover, this acquisition requires NANOG, SMAD2, and DPY30.

While we are unable to rule out the possibility that these are separate, overlapping activities, however, given the existence of a NANOG-SMAD2 DPY30 complex in hESC [16], these factors may regulate a core set of factors directly by modulating the epigenome as a complex (S14 Fig). This hypothesis could explain our observations that AC depleted of NANOG appeared to be hypersensitised to *Activin*. In this scenario, NANOG may act as a rheostat of SMAD2 activity, as has been shown for SMAD1/5 in mouse ESC [46]. In AC depleted of NANOG, free phosphor-SMAD2 would be in greater abundance and thus free to activate targets it would only usually induce at higher levels (S4F Fig). Regardless of whether the epigenetic effects of NANOG depletion are direct or indirect, this does suggest that Nanog may have a conserved role in regulating the competence to produce mesodermal subtypes via regulation of the epigenome in terrestrial vertebrates [16].

Unexpectedly, our observations of NANOG’s function in axolotl development did not closely align with observations in other nonmammalian vertebrates. Anuran amphibians (frogs) such as *Xenopus tropicalis* and *Xenopus laevis*, lack a *Nanog* gene [23]. It has been proposed that *Vent* family of transcription factors fulfils the role of *Nanog* in frogs [32,42]. Evidence presented by Scerbo and colleagues does suggest that *Vent* and *Nanog* do share common ancestry, and we did observe some common gene targets. However, our observed axolotl NANOG KD phenotype showed little resemblance to the phenotype of *Vent1/2* KD in *Xenopus* [32]. Curiously, our observed phenotype more closely resembled *Nanog* KD in zebrafish [33] or *Nanog*-null mice [14]. We did not, however, see a large-scale failure to establish ZGA or clear maternal mRNA transcripts as reported by Lee and colleagues [33]. The role of Nanog in zebrafish is subject to debate with some suggesting that zebrafish *Nanog* only marginally effects ZGA and that zebrafish *Nanog* primarily regulates the yolk syncytium, a teleost specific extra embryonic tissue [34]. While there are some common functions of the yolk syncytium and amphibian organiser, it may be that zebrafish *Nanog* has in part co-opted regulation of these tissues.

One explanation for why no common function for Nanog has been identified outside of mammals is that the pluripotency and germ line GRNs may be intrinsically tied and the most well-studied nonmammalian organisms such as frogs and zebrafish have diverged as a result of changing their mode of germ cell determination [5,6,47,48]. Both pluripotent cells and germ cells share many common transcriptional regulators, including *Nanog* and *Pou5f1* that are not expressed in other cell types, they also share many epigenetic features [49]. Interestingly, a recent study demonstrated that another pluripotency factor POU5F3 that has been retained in frogs and zebrafish in place of pluripotency factor POU5F1 has changed function substantially in these organisms, correlating with evolution of deterministic PGC specification [50]. It is then conceivable that **Nanog’s function** may have similarly diverged in these organisms.

## Methods

### Axolotl embryos and explants

Procedures involving animals have been approved by the Animal Welfare Ethics Committee Review Board, University of Nottingham and adhered to the Home Office Code of Practice guidelines for the Housing and Care of Animals used in Scientific Procedures (License PPL3003396, https://www.gov.uk/government/publications/code-of-practice-for-the-housing-and-care-of-animals-bred-supplied-or-used-for-scientific-purposes). Embryos were collected following matings as described previously [13]. For microinjection, embryos were manually de-jellied and cultured in 1× modified Barth’s solution (MBS) with 4% Ficoll (Sigma). Embryos were staged as previously described and in line with Bordzilovskaya and Dettlaf’s stages of axolotl [13,23]. From stage 7 onwards, embryos were maintained in 0.2× MBS, and dissected explants were maintained in 0.7× Marc’s modified ringers solution (MMR). Culture solutions were supplemented with antibiotics (50 μg/ml penicillin and streptomycin and 50 μg/ml kanamycin), 100 μg/ml ampicillin and 50 μg/ml fungizone.

### Morpholino and RNA microinjections

Morpholino oligonucleotides (GeneTools, LLC, OR) were designed to block translation of target proteins. Intron/exon boundaries were predicted by homology. Sequences were obtained from our axolotl genomic resource (website URL not finalised) [25], or via the axolotl genome website [26]. The morpholino sequences used were as follows: Translation MO: *Nanog*, 5′- GGTCAATCCAAAAGCTCCTCCTAAG-3′; Splice MO: *Nanog* 5′-GGCAGGACTGAAACAAAACGAAGAC-3′; Translation MO: DPY30 5′-ATGCTTTGTTCCGACTCCATTGTGA-3′ and 5′-TTATCGTAGCCCGTCACTCCAGCTC-3′. A nonspecific morpholino was injected in each experiment at equivalent levels to the specific splice morpholino combinations: MO: Control, 5′-GGATTTCAAGGTTGTTTACCTGCCG-3′. Each morpholino experiment was repeated at least 3 times, and the efficacy of the splice morpholinos was tested by PCR in each experiment using primers detailed in Table 1. The Activin-nodal inhibitor SB431542 (Sigma) was solubilised in dimethyl sulfoxide and used at a final concentration of 150 μm. In vitro transcription and microinjection mRNAs for microinjection were synthesised using mMessage mMachine (Ambion) from plasmids encoding; *Xenopus* eFGF, *Xenopus* Smad2C (XSmad2C), human *Nanog*, and human DPY30. All mRNA or morpholino microinjections were carried out at the 1 cell stage.

### High-resolution electron microscopy (HREM)

Samples were fixed overnight at 4°C in 4% PFA in PBS before being dehydrated overnight in increasing concentrations of methanol (50%, 70%, 80%, 90%, and 100%) before being mounted in JB4 medium with acridine orange (SIGMA) and processed for HREM as described by Mohun and Weninger [51]. Section TIFF images were trimmed using the image processor function on photoshop before being imported into Osirix MD version 12.5.0 to produce embryo 3D reconstructions using the magma shader.

### Western blotting

For western blot analysis, whole embryos were lysed in RIPA buffer and homogenised using a dounce homogeniser. Cell lysates were then centrifuged at 13,000 rpm at 4°C for 5 min. Supernatant was then collected, and protein concentration was assessed using Protein Assay reagent (BIORAD) according to manufacturer’s instructions. Approximately 25 μg of protein was used for each well for subsequent SDS-PAGE performed using standards methods. To test antibodies raised against axolotl NANOG, synthetic poly-A RNA encoding *Nanog*-HA were injected into mature *Xenopus* oocytes, from which lysates were prepared for western blotting. In addition, an uninjected *Xenopus* oocyte lysate and a multiple antigen containing cell lysate were also used as HA-negative and positive controls, respectively. Axolotl NANOG antibodies were produced by DundeeCell and raised against antigenic peptides specific to axolotl NANOG. All other antibodies are commercially available and listed in Table 2 as well as the used concentrations.

### Immunofluorescence

Prior to immunofluorescence, embryos were incubated with an increasing gradient of sucrose/PBS solution (30%, 50% 80% sucrose/PBS) prior to mounting in OCT compound and frozen at −80° overnight. The blocks were transferred to −20° 2 h before sectioning. Antigen retrieval was performed by boiling the slides in 0.01 M citrate buffer (pH 6.0) for 10 min. Sections were permeabilized with 1% Triton X-100 in PBS for 15 min. Slides were then immersed in blocking solution (PBS supplemented with 5% BSA for 2 h). After blocking, sections were incubated with primary antibody overnight at 4°C in a humidified chamber. Slides were then washed 3 times with 0.1% Tween-20/PBS before being incubated with a fluorescent secondary antibody (Table 2) for 45 min at room temperature. Slides were treated with mounted with Fluoroshield with DAPI (Sigma) and sealed with nail varnish. Slides were kept at −20°C until observed.

### First-strand cDNA synthesis was used to prepare samples for qPCR analysis

A reaction consisting of 1 μl of Oligo(dT)_20_ (50 μM), 2 μg of total RNA, and 1 μl of 10 mM dNTP mix, was topped up to 13 μl using sterile distilled water. The reaction was heated to 65°C for 5 min, and then incubated at 4°C for 5 min. The tube was briefly centrifuged before the addition of 4 μl 5× First-Strand Buffer, 1 μl 0.1 M DTT, 1 μl RNaseOUT recombinant RNase inhibitor (40 units/μl), and 1 μl SuperScript III RT (200 units/μl). All reagents from Thermo Fisher Scientific. The reaction mix was then heated to 25°C for 5 min, 50°C for 60 min, and 70°C for 15 min, before being chilled to 4°C.

Each qPCR reaction contained 5 μl of SYBR-Green Jumpstart Taq readyMix (SIGMA), 1 μl (10 μM [final]) of each of the forward and reverse primers (SIGMA), 2 μl of nuclease-free water, and 1 μl of cDNA template. Each reaction was prepared in triplicate on an ABI FAST Systems 0.2 ml 96-well PCR plate (STARLAB), and then sealed with an Optical Adhesive cover (Life Technologies).

The following qPCR conditions were utilised for each run on the QuantStudio 6 Flex (Life Technologies) qPCR instrument: The plate was heated to 105°C to activate the Jumpstart Taq before being held at 50°C for 2 min. The initial denaturation step was at 94°C for 10 min, followed by 40 cycles of 94°C for 15 s (denaturation) and 60°C for 1 min (annealing and extension). The raw data was then extracted to be analysed by comparative CT (cycle threshold). An endogenous control gene was chosen for each qPCR run to normalise the data in order to compare relative fold change of target genes among cDNA samples.

The double delta CT value (ΔΔCT) was calculated using the following formula: ΔCT target—ΔCT reference = ΔΔCT. As all calculations are in logarithm base 2, the expression fold change of each target gene was calculated using 2^-ΔΔCt.

### RNA-seq analysis

For staged uninjected, NANOG KD, SB43-treated, and DPY30 KD axolotl samples, RNA-seq, each sample was mapped to the transcriptome assembly described previously [52] using RSEM with default parameters. This assembly was used as it also contains several developmental genes identified by our group in previous publications. Two of the Nanog KD stage 22 biological replicates were removed based on their lack of correlation with all other samples. The resulting count files were input to edgeR to calculate differentially expressed genes (DEGs) (FDR < 0.05, logFC > 1). Heatmaps were drawn using the log2 TPM values in R using heatmap.3 with default cluster settings.

The 17 stages of wild-type axolotl early development was acquired by mapping the reads from the Jiang and colleagues (2017) datasets [24] to the same transcriptome assembly as before. Mean TPM values for each stage were calculated in R. These values were then used to determine whether each gene was “early,” “late,” or “global.” Genes that were only expressed with a TPM > 10 before stage 12 were classed as “early,” genes that were only expressed with a TPM > 10 on or after stage 12 were classed as “late.” The remaining genes were all considered global and were divided into “global high” and “global low” based on whether they had a TPM > 10 in every stage of the Jiang data or not. In order to construct the heatmap displaying every sample condition versus every sample condition, the heatmap row dendrogram was split at a height of 21, and each cluster was assigned the most common expression pattern of the genes within that cluster.

### Comparison of human, pig, *Xenopus*, and tissues

In total, single-cell transcriptomes from 144 pig peri-gastrulation stage epiblast cells from Ramos-Ibeas and colleagues [28], 152 human peri-gastrulation stage epiblast cells retrieved from Petropoulos and colleagues [29] were merged by calculating the geometric mean across all cells to get the simulated average count for each pluripotent tissue. This was then compared with whole RNA-seq data from st10.5 *Xenopus* ACs retrieved from Angerilli and colleagues [27] and stage 10.5 axolotl ACs (our study). Given the differences in sequencing methodology and sample preparation etc., we employed quantile normalisation to compare the relative abundance of expressed genes.

### Gene set enrichment using *Xenopus* cell type markers

The R package hypeR was used [53] to perform a custom GSEA of DEGs using a list of amphibian cell type markers identified by Briggs and colleagues [35]. Analysis was carried out using an FDR threshold of 0.01. Cell types that were significantly enriched in the dataset were plotted using GOchord in R.

### ChIP QPCR

A total of 50 AC explants per experimental condition were processed using the truCHIP Chromatin Shearing Tissue Kit with Formaldehyde (Covaris). Caps were fixed with 1% methanol-free formaldehyde-PBS (Covaris) for 15 min before quenching. Fixed caps were processed according to the manufacturer’s protocols, and 1 ml of chromatin was sheared using the S220 ultra-sonicator (Covaris) in AFA fibre containing vesicles (Covaris). Chromatin shearing was performed under the following conditions: duty cycle 5%, intensity level 4, cycles/burst 200. The shearing program was run for 15 min resulting in chromatin fragments of 100 to 500 bp. Approximately 100 μl of sheared chromatin was used per ChIP using the EZ MAGNA ChIP kit (Millipore) according to the manufacturer’s instructions. Purified DNA was then used for subsequent qPCR analysis. QPCR primers for gene transcription start sites (TSSs) are listed in Table 1.

**Table 1:** Primers used in this study.

Gene	Forward/reverse	Sequence
Acta1	Reverse	ATCCACATCTGCTGGAAGGT
Acta1	Forward	CGCATGCAGAAGGAGATCAC
Tbxt	Forward	CATTGACCACATGTACCAA
Tbxt	Reverse	GATCAAGGGTCAATCGTGAGTTC
C8b TSS	Forward	CGATTCTCAAGCTCGGCAAC
C8b TSS	Reverse	CTGTACTCCAAAGGAAGACTGT
Krt19 (Cytok)	Reverse	GGAGCCGCGTCCATCTC
Krt19 (Cytok)	Forward	AACCACCAAGAGGAATTGCAA
Krt19 (Cytok)TSS	Forward	TATTATAGTGGTGCGGTGCC
Krt19 (Cytok)TSS	Reverse	AGGTGGAGATGCGTAGTTCC
Des	Reverse	GTCTGGATTGGCATGGTGAC
Des	Forward	CGAGATCCGCAACTTGAAGG
Eef1a1	Reverse	CATGCAGCCAACGAACTATGTATT
Eef1a1	Forward	GTATGATGAGGTTCCTGTGCATTG
Endodermin	Reverse	GAAACCCGTAGGTGGACAGAGA
Endodermin	Forward	CTGACCAGGAGGGAAAAGCTT
Fabp1	Reverse	CCGTTCTGCTCCATCTCAGA
Fabp1	Forward	AGCTCCAGTCCCAGGAAATC
Foxa2	Forward	ACGACTGGAGCAGCTACTAC
Foxa2	Reverse	CGGTGTTCACGTAGGACATG
Gata4 TSS	Forward	GCAGGATTGGCAGACACAAG
Gata4 TSS	Reverse	TCTGGGTCCGTCTCCTCA
Grhl1	Reverse	CTTTGTTTGGCCGTGTGCTG
Grhl1 TSS	Forward	CAACCCGAAAGTCCAGTTCC
Grhl1 TSS	Reverse	TTCCAGGCTTCGTCTTCACT
Grhl1	Forward	CGGCAACAAAGGCATACACC
Hand1	Reverse	CTGTCCGCCCTTTCATCTTC
Hand1	Forward	CGGGCCGAGCTGAAGAA
Hnf4g TSS	Forward	CCGAGTTCCAAGTTTTCTGCA
Hnf4g TSS	Reverse	TGAGACTCAGCATTCCAAAGG
Hoxc6	Reverse	TGGCGATTTCGATCCTCCTG
Hoxc6	Forward	ATGAATTCCCACAGTGGCGT
Mix	Reverse	GCTTCTGGGTGGATTTGATTTATAA
Mix	Forward	GTCCAGGATCCAGGTCTGGTT
Myf5	Reverse	GGCGCTGTCAAAGCTGTTG
Myf5	Forward	GGGAGCCCCCTTTCCAA
Myf5 TSS	Reverse	ACTAGGTCCATGCTGTCACC
Myf5 TSS	Forward	CAGATCAGGACCTTGCCCTC
Nanog	Forward	ACTTTACCAAAAAGCGTGACACTAGA
Nanog	Reverse	ACAGAGCACCCAATTTTCCAA
Ncam	Forward	GCCCCTAAGTTGCAAGGCC
Ncam	Reverse	TCTCGTTTGTCTGTGGGGC
Nestin	Forward	GTGGTTGAAGGAGAAAGGCG
Nestin	Reverse	GTAGTCCTCGATCTCCACGG
Neurod4	Reverse	GGTGGCTGACAAAAGAAGGG
Neurod4	Forward	ACTATCACAACCGACCAGCA
Neurog	Reverse	CTCCTCGTCCGAGTAAGA
Neurog	Forward	GTCGTTCAAAACCGAGAG
NKkx2.5 TSS	Forward	GAGGACATGACTGCTCTGG
NKkx2.5 TSS	Reverse	GGTGACGGGGCTGTGGAA
Nodal1	Reverse	GGGTCGGGTGGTACAGCTT
Nodal1	Forward	CCCAGTGGATGAAACGTTCAG
Nodal1 TSS	Forward	CGTTCACAGCCCGACAAATA
Nodal1 TSS	Reverse	ACCCTCAAAGCGAAAATCCG
Nodal2	Reverse	CCCGCTCTGGAATGTACAATTT
Nodal2	Forward	CATACCGCTGTGATGGAAAGT
Odc1	Reverse	CCCGGACCCAGGTTACG
Odc1	Forward	ATGCCCGTCATGAGTAGTACCA
Pax6	Forward	GAAGTGGAGAAGGGAAGAGAAACTG
Pax6	Reverse	TGATGTAAATGAAACTGGTGTCGTG
Pou5f1/Oct4	Reverse	ACATCCGCCTGCGTAAAGC
Pou5f1/Oct4	Forward	GCGGACCTTGAACAGTTTGC
Runx1	Reverse	TTGGGAACTGTCGGTCAATTC
Runx1	Forward	CGCCTCTCTGGTGCATCTG
Sox11	Forward	TGAGCCTGAACTTCTCCTCG
Sox11	Reverse	AGTCTGAGAAGTTGGCCTCC
Sox17	Forward	TTTTTGTGGAAACCTATGGGCCAC
Sox17	Reverse	CGATTTTTATTAGCCGACCACACA
Sox2	Reverse	GGCAGGTACATGCTAATCA
Sox2	Forward	GGTCAAGTCCGAATCGAG
Sox21	Reverse	GTGTGTGTGCGTGTCTTCAT
Sox21	Forward	AGAAGGGCCTTGCAAAATGG
Sox7	Forward	CGAGCTGCTAGAGATGGACA
Sox7	Reverse	GTTGTAGTATGCGGCTGTGG
Sox8	Reverse	TGCTTGCCTCCATGATGAAG
Sox8	Forward	CGGGAGGCCAACTCTACAA
Vim	Reverse	CGTCCTGAAGGCGGTTAATG
Vim	Forward	CGAAGTGGATGCCCTGAAAG
Vim TSS	Forward	GTGACTCAGTCCAGTAGCCT
Vim TSS	Reverse	TCCGGGGCGCTCTTTAATAC

**Table 2:** Antibodies used in this study.

Antibody	Species	Supplier	Catalogue number
Anti-H3K4me3	Rabbit	Abcam	Ab8580
Anti-H3K27ac	Rabbit	Abcam	Ab4729
Anti-H3K36me3	Rabbit	Abcam	Ab9050
AntiH3K27me3	Rabbit	Abcam	Ab6002
Anti-H3	Rabbit	Abcam	Ab1791
Anti-HA	Rabbit	Abcam	Ab9110
Anti-Nanog (axolotl)	Rabbit	Dundee cell	Custom made (available on request)
Anti-PolII	Rabbit	Abcam	Ab5131

## Supporting information

S1 Fig NANOG KD causes aberrant differentiation. (**a**) Validation of antibodies raised against axolotl NANOG using western blotting. Lanes were loaded with lysates made from mature *Xenopus* oocytes following injection with Synthetic poly-A RNA encoding NANOG-HA or a multi-antigen tagged cell lysate. (**b**) Western blot confirming complete KD of NANOG following translation MO injection at equivalent stage 10.5. (**c**) Validation of aberrant splicing in response to *Nanog* splice-morpholino and brightfield image of a stage 22 equivalent time point embryo following an injection of 80 ng *Nanog* splice MO at the 1 cell stage. (**d**) NANOG translation MO KD and rescue efficiencies from a (*n* = 25 per experimental condition). (**e**) HREM imaging of mid-gastrula embryos with and without NANOG translation MO KD at equivalent stage 10.5. Visible structures highlighted: involuting axial mesoderm (AM), ingressing ventral mesoderm (VM), archenteron (A), blastocoel (B). Scale bar, 1 mm (*n* = 2). (**f**) HREM imaging of stage 40 uninjected and stage matched NANOG translation MO KD + hNANOG rescue (dorsal view) (*n* = 2). (**g**) QPCR validation of key transcriptome findings (10 embryos pooled per experimental condition). Dots show individual data points. Asterisks represent the adjusted *p*-value obtained from unpaired one-sided multiple *t* tests, * = *P* ≤ 0.05, ** = = *P* ≤ 0.01, *** = *P* ≤ 0.001, *** = *P* ≤ 0.001, **** = *P* ≤ 0.0001, ns = *P* > 0.05. (**h**) Differentiation markers of uninjected at stage 28 and NANOG translation MO-depleted time point matched embryos (*n* = 10 × 3). Asterisks values same as described in h. The data underlying this figure are available in S1 Images and in S1 Data.
(TIFF)

S2 Fig Differential gene expression following NANOG translation MO KD. (**a**) Dot plots showing significantly enriched (padj <0.01). GO database biological process gene sets within NANOG translation MO KD DEGs at equivalent stage 10.5. (**b**) Ridgeline plots showing significantly enriched (padj <0.01) KEGG pathway gene sets within NANOG translation MO KD DEGs at equivalent stage 10.5. (**c**) Volcano plots showing significant DEGs in stage 22 equivalent stage NANOG translation MO KD embryos compared to stage 10.5 uninjected embryos. Vertical dotted line indicates a Log2 fold change of 1.5. Horizontal line indicates padj threshold of 0.01 on an -log10 scale. Orange points indicate significantly differentially expressed genes. (**d, e**) As with a and b, except stage 22 equivalent NANOG translation MO KD embryos are compared against stage 10.5 uninjected embryos. (**g–i**) As with c–e, except stage 22 equivalent NANOG translation MO KD embryos are compared against stage 10.5 equivalent NANOG translation MO KD embryos. (**j–l**) As with c–e, except NANOG translation MO KD embryos are at a stage equivalent to 22 and are compared against uninjected embryos at stage 22. The data underlying this figure are available in S1 Data.
(PDF)

S3 Fig Enrichment of amphibian cell type markers in NANOG KD DEGs. Chord diagrams showing the results of GSEA of amphibian cell type-specific markers in differentially expressed genes in NANOG translation MO KD. (**a**) NANOG translation morphant up-regulated DEGs are enriched for markers of optic neuron, involuted dorsal mesoderm, pronephric mesenchyme, and neuroendocrine cells at a stage equivalent to 10.5. Down-regulated DEGs at equivalent stage 10.5 are enriched for markers of neuroectoderm and organiser mesoderm. (**b**) Up-regulated DEGs in stage 22 equivalent NANOG translation MO KD embryos are enriched for markers of the blastula stage and germ cells. Down-regulated DEGs are enriched for markers of lateral plate, notochord, and cardiac mesoderm as well as markers of chordal neural crest cells, anterior neural tube, and epidermal progenitor cells.
(TIFF)

S4 Fig NANOG translation morphants are hypersensitive to *Activin*. (**a**) Schematic of morpholino injection regime and animal cap assay. (**b**) Differentiation markers of uninjected and NANOG translation MO-depleted AC explants at a stage equivalent to 22 in uninjected whole embryo controls (*n* = 3, 15 explants pooled per experimental condition). Dots show individual data points. Asterisks represent the adjusted *p*-value obtained from unpaired one-sided multiple *t* tests, * = *P* ≤ 0.05, ** = = *P* ≤ 0.01, *** = *P* ≤ 0.001, *** = *P* ≤ 0.001, **** = *P* ≤ 0.0001, ns = *P* > 0.05. (**c**) Schematic of mesoderm induction animal cap assay. (**d**) Images of cultured uninjected and NANOG translation MO-depleted stage 22 equivalent cap explants following treatment with different activin concentrations corresponding to the bar graph in Fig 3D. (**e**) QPCR showing NANOG translation MO-depleted caps express foregut/hindgut markers but not mature foregut/hindgut markers in response to activin (*n* = 3, 15 explants pooled per experimental condition). Asterisks represent the adjusted *p*-value obtained from Tukey’s multiple comparisons test following one-way ANOVA. Asterisks values same as described in b. (**f**) Schematic: NANOG may act as a rheostat of SMAD2 activity. The data underlying this figure are available in S1 Images and in S1 Data.
(TIFF)

S5 Fig NANOG translation MO KD results in the loss of activating promoter marks. (**a**) Uninjected, NANOG translation MO depleted and hNANOG rescued AC explants cultured to equivalent stage 10.5 and stained for H3, H3K36me3, and DAPI (*n* = 3 per experimental condition). Scale bar, 60 μm. (**b**) Untreated, SB treated and DPY30 translation MO-depleted animal cap explants cultured to equivalent stage 10.5 and stained for H3, H3K36me3, and DAPI (*n* = 3 per experimental condition). Scale bar, 60 μm. (**c**) Untreated, NANOG translation MO depleted, SB treated, and DPY30 translation MO-depleted animal cap explants cultured to equivalent stage 10.5 and stained for phospho-POLII and DAPI (*n* = 3 per experimental condition). Scale bar, 60 μm. (**d**) Vegetal explants form endoderm in a cell-autonomous manner. QPCR of germ-layer markers of animal and vegetal explants compared with animal cap explants treated with different activin concentrations at time points equivalent to stages 10.5 and 30 in uninjected whole embryo controls (*n* = 3, 15 explants pooled per experimental condition). Dots show individual data points. Asterisks represent the adjusted *p*-value obtained from obtained from Tukey’s multiple comparisons test following one-way ANOVA, * = *P* ≤ 0.05, ** = = *P* ≤ 0.01, *** = *P* ≤ 0.001, *** = *P* ≤ 0.001, **** = *P* ≤ 0.0001, ns = *P* > 0.05. The data underlying this figure are available in S1 Images and in S1 Data.
(TIFF)

S6 Fig Differential gene expression following SB treatment. (**a**) Volcano plots showing significant DEGs in stage 22 equivalent SB-treated embryos compared to uninjected stage 10.5 embryos. Vertical dotted line indicates a Log2 fold change of 1.5. Horizontal line indicates padj threshold of 0.01 on an -log10 scale. Orange points indicate significantly differentially expressed genes. (**b**) Dot plots showing significantly enriched (padj <0.01) GO database biological process gene sets within SB-treated DEGs at equivalent stage 10.5. (**c**) Ridgeline plots showing significantly enriched (padj <0.01) KEGG pathway gene sets within SB-treated DEGs at equivalent stage 10.5. (**d–f**) As with a–c, except stage 22 equivalent SB-treated embryos are compared against stage 10.5 equivalent SB-treated embryos. (**g, h, l**) As with a–c, except SB-treated embryos are at a stage equivalent to 22 and are compared against uninjected embryos at stage 22. The data underlying this figure are available in S1 Data.
(TIFF)

S7 Fig Validation of SB transcriptome. (**a**) QPCR validation of key transcriptome findings. Dots show individual data points. Asterisks represent the adjusted *p*-value obtained from unpaired one-sided multiple *t* tests, * = *P* ≤ 0.05, ** = = *P* ≤ 0.01, *** = *P* ≤ 0.001, *** = *P* ≤ 0.001, **** = *P* ≤ 0.0001, ns = *P* > 0.05. (**b**) QPCR analysis of late differentiation markers (*n* = 3, 10 embryos pooled per experimental condition). (**c**) Differentiation marker expression of uninjected and SB-treated AC explants at a stage equivalent to 22 in uninjected whole embryo controls (*n* = 3, 15 explants pooled per experimental condition). (**d**) Vegetal explants can form definitive endoderm even in the presence of SB431542 inhibitor. Vegetal explants from untreated or SB-treated embryos were cultured with or without SB431542 and assayed for endodermal markers at time points equivalent to stages 10.5 and 22 in uninjected whole embryo controls (*n* = 3, 15 explants pooled per experimental condition). Asterisks represent the adjusted *p*-value obtained from Tukey’s multiple comparisons test following one-way ANOVA. Asterisks values same as in a–c. (**e**) Luciferase complementation assay (*n* = 3). Axolotl NANOG can physically interact with axolotl SMAD2, interactions are increased in the presence of constitutively active ALK4, binding is disrupted with SB treatment. The data underlying this figure are available in S1 Data.
(TIFF)

S8 Fig Enrichment of amphibian cell type markers in SB-treated embryo DEGs. (**a**) Chord diagrams showing the results of GSEA of amphibian cell type-specific markers in differentially expressed genes following SB treatment. (**a**) SB-treated embryo down-regulated DEGs are enriched for markers of organiser and marginal zone mesoderm as well as organiser endoderm, neuroectoderm, placodal area, optic neuron, and pronephric mesenchyme at equivalent stage 10.5. (**b**) Up-regulated DEGs in SB-treated embryos are enriched for markers of the ciliated epidermal progenitors, beta ionocytes, goblet cells, ionocytes, hatching gland cells, and cardiac mesoderm at equivalent stage 22. Down-regulated DEGs are enriched for markers of notochord, somitic, lateral plate, dorsal, intermediate, and organiser mesoderm as well as markers of organiser endoderm, chordal neural crest cells, anterior neural tube, otic placode, and endothelial hemangioblast cells. The data underlying this figure are available in S1 Data.
(TIFF)

S9 Fig DPY30 translation MO KD resembles NANOG translation MO KD. (**a**) DPY30 translation MO KD arrests development post-gastrulation. Brightfield and HREM images of uninjected and DPY30 translation MO-depleted embryos. Dotted line marks plane of section reconstruction. Dashed line delimits visible structures: Somites (S), Neural tube (NT) Notochord (No), Mesonephric ducts (Me), Blastocoel (B) (*n* = 2). Scale bar, 1 mm. (**b**) DPY30 KD and rescue efficiencies (*n* = 30 per experimental condition). (**c, d**) Gene expression of key cell type marker genes at equivalent stages 10.5 and 22 in uninjected (*n* = 7 and 6, respectively) and DPY30 translation MO KD (*n* = 6 and 6, respectively) embryos. Cell types: animal cap (AC), mesendoderm specification (general) (MES), endoderm specification (general) (ES), organiser endoderm (OE), vegetal endoderm (VE), mesoderm specification (general) (MS), organiser mesoderm (OM), marginal zone (MZ), definitive endoderm (general) (DE), foregut (FG), hindgut (HG), ventral-lateral plate (VLP), intermediate mesoderm (I), somite (S), notochord (No), definitive ectoderm (general) (Dec), neural tube (NT), neural crest (NC), epidermal progenitors (EP). (**e**) Gene expression of Hox gene family members in DPY30 translation MO KD embryos at a time point equivalent to stage 22. The data underlying this figure are available in S1 Images and in S1 Data.
(TIFF)

S10 Fig Differential gene expression following DPY30 translation MO KD. (**a**) Volcano plots showing significant DEGs in DPY30 translation MO KD embryos compared to equivalent stage 10.5 uninjected embryos. Vertical dotted line indicates a Log2 fold change of 1.5. Horizontal line indicates padj threshold of 0.01 on an -log10 scale. Orange points indicate significantly differentially expressed genes. (**b**) Dot plots showing significantly enriched (padj <0.01) GO database biological process gene sets within DPY30 translation MO KD DEGs at equivalent stage 10.5. (**c**) Ridgeline plots showing significantly enriched (padj <0.01) KEGG pathway gene sets within DPY30 translation MO KD DEGs at equivalent stage 10.5. (**d–f**) As with a–c, except stage 22 equivalent DPY30 translation MO KD embryos are compared against stage 10.5 uninjected embryos. (**g–i**) As with a–c, except stage 22 equivalent DPY30 translation MO KD embryos are compared against stage 10.5 equivalent DPY30 KD embryos. (**j–l**) As with a–c, except DPY30 translation MO KD embryos are at a stage equivalent to 22 and are compared against uninjected embryos at stage 22. The data underlying this figure can be found in S1 Data.
(TIFF)

S11 Fig Validation of DPY30 KD transcriptome. (**a**) QPCR of genes representative of key lineages. Dots show individual data points (10 embryos pooled per experimental condition). Asterisks represent the adjusted *p*-value obtained from unpaired one-sided multiple *t* tests, * = *P* ≤ 0.05, ** = = *P* ≤ 0.01, *** = *P* ≤ 0.001, *** = *P* ≤ 0.001, **** = *P* ≤ 0.0001, ns = *P* > 0.05. (**b**) Differentiation markers of uninjected and Nanog depleted AC explants at stage equivalent to 22 in uninjected controls (*n* = 3, 15 explants pooled per experimental condition). (**c**) DPY30 translation MO depletion prevents mesodermal but not endodermal differentiation in response to activin. QPCR of germ-layer markers of uninjected and DPY30 translation MO-depleted stage 20 equivalent caps following treatment with different activin concentrations (*n* = 3, 15 explants pooled per experimental condition). Asterisks represent the adjusted *p*-value obtained from Tukey’s multiple comparisons test following one-way ANOVA. Asterisks values same as described in a and b. (**d**) DPY30 translation MO depletion reduces mesodermal gene expression and increased neuronal gene expression in response to FGF. QPCR of germ-layer markers of uninjected and DPY30 translation MO-depleted stage 20 equivalent caps following treatment with FGF (15 explants pooled per experimental condition, individual points represent technical repeats). Statistics and asterisks values same as described in d. (**e**) Uninjected, DPY30 translation MO 1 and 2 depleted embryos and hDPY30 rescued animal cap explants cultured to equivalent stage 10.5 and stained for H3K4me3, H3K27ac, H3K27me3, and DAPI (*n* = 3). Scale bar: 60 μm. The data underlying this figure are available in S1 Images and in S1 Data.
(TIFF)

S12 Fig Enrichment of amphibian cell type markers in DPY30 translation MO KD DEGs. Chord diagrams showing the results of GSEA of amphibian cell type-specific markers in differentially expressed genes following DPY30 translation MO KD. (**a**) DPY30 translation MO morphant down-regulated DEGs are enriched for markers of organiser mesoderm and endoderm at equivalent stage 10.5. (**b**) Up-regulated DEGs in DPY30 translation MO KD stage 22 equivalent embryos are significantly enriched for markers of blastula cells and organiser endoderm. Down-regulated DEGs are enriched for markers of lateral plate, notochord, somitic, dorsal lateral plate, and cardiac mesoderm as well as markers of chordal neural crest cells, anterior neural tube, otic placode, chordal neural plate border, anterior neural tube, and epidermal progenitor cells.
(TIFF)

S13 Fig NANOG gene target loci lack H3K4me3. (**a**) H3K4me3 ChIP of equivalent stage 10.5 uninjected, NANOG translation MO KD, DPY30 translation MO KD, and SB-treated caps followed by qPCR using probes directed at gene promoter regions 50 pooled caps per experimental condition, data points represent technical repeats. Dots show individual data points. Asterisks represent the adjusted *p*-value obtained from obtained from Tukey’s multiple comparisons test following one-way ANOVA, * = *P* ≤ 0.05, ** = = *P* ≤ 0.01, *** = *P* ≤ 0.001, *** = *P* ≤ 0.001, **** = *P* ≤ 0.0001, ns = *P* > 0.05. (**b**) H3K4me3 ChIP-qPCR of equivalent stages 10.5 and 22 uninjected caps, stage 22 equivalent *Activin* treated caps with and without NANOG and DPY30 translation MO depletion, as well as stage 22 equivalent SB-treated caps. Statistics and asterisk values same as described in a. The data underlying this figure are available in S1 Images and in S1 Data.
(TIFF)

S14 Fig NANOG may regulate in co-operation with SMAD2 and DPY30. (**a, b**) Schematics of hypothesis outline.
(TIFF)

S1 Table Axolotl early development time course. Publicly available bulk-RNAseq data containing transcriptomes of axolotl embryos from 16 developmental time points [24].
(XLSX)

S2 Table Comparison of the pluripotent transcriptomes of axolotl, humans, pigs, and frogs. Bulk-RNAseq transcriptomes of dissected stage 10.5 axolotl caps compared to stage 10.5 frog animal caps and pseudo bulked pig and human epiblast clusters from SC-RNAseq datasets [27–30].
(XLSX)

S3 Table Control and morphant transcriptomes. Bulk-RNAseq transcriptomes of uninjected, SB431542 treated, NANOG, or DPY30 KD axolotl embryos at equivalent stages 10.5 and 22.
(XLSX)

S4 Table Differential gene expression. Differential gene expression of morphant transcriptomes compared to their stage-matched uninjected siblings.
(XLSX)

S5 Table GO term enrichment in DEGs. Significantly enriched GO terms in the DEGs from S4 Table.
(XLSX)

S6 Table KEGG term enrichment in DEGs. Significantly enriched KEGG terms in the DEGs from S4 Table.
(XLSX)

S7 Table Zygotically expressed axolotl genes. Genes from S1 Table with a TPM >1 after stage 7 (18 HPF) and before stage 10 (50 HPF).
(XLSX)

S8 Table Maternally expressed axolotl genes. Genes from S1 Table with a TPM >1 at stage 1 (Zygote) and were <1 by stage 9 (40 HPF).
(XLSX)

S1 Images Related to Figs 2A and 3F and 4A and 5C and S1C and S1E and S5A–S5D and S9A and S11E and S13A and S13B. (PDF)

S1 Data Related to Figs 2B and 3D and 6D and 6E and S1D and S1G and S1H and S4B and S4E and S5D and S6 and S7A–S7E and S9b and S11A–S11D and S13A and S13B. (XLSX)

## Abbreviations

AC: animal cap
CT: cycle threshold
DEG: differentially expressed gene
GRN: gene regulatory network
GSEA: gene set enrichment analysis
hESC: human embryonic stem cell
HPF: hours post fertilisation
HREM: high-resolution episcopic microscopy
LOF: loss of function
MBS: modified Barth’s solution
mESC: mouse embryonic stem cell
MMR: modified ringers solution
TSS: transcription start site
ZGA: zygotic genome activation
