# Supplementary figures and images for: NANOG is required to establish the competence for germ-layer differentiation in the basal tetrapod axolotl
Source: PLoS Biol. 2023 Jun 14;21(6):e3002121. doi: 10.1371/journal.pbio.3002121 (PMC10599592; doi:10.1371/journal.pbio.3002121)

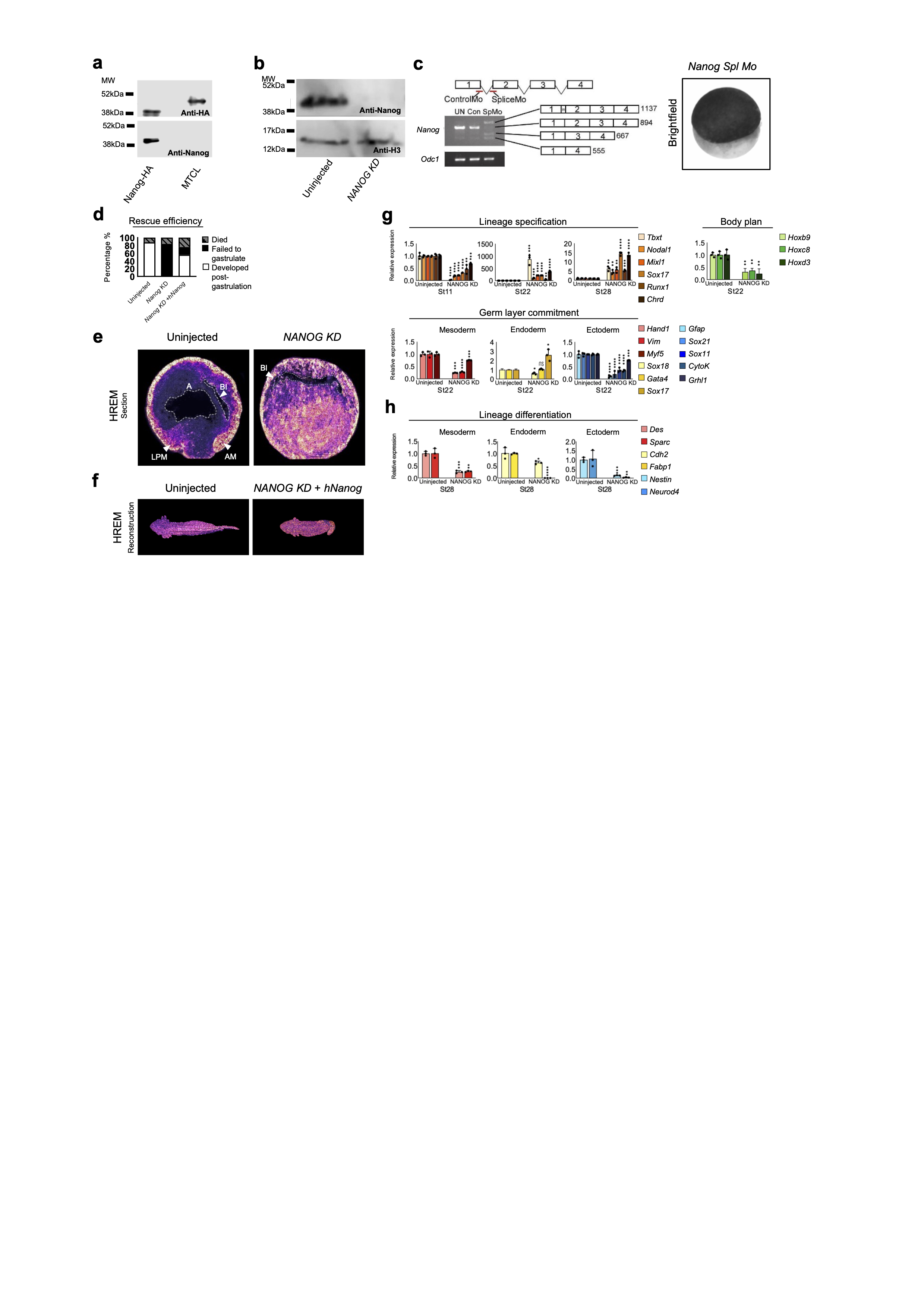

Supplement: S1 Fig — (a) Validation of antibodies raised against axolotl NANOG using western blotting. Lanes were loaded with lysates made from mature Xenopus oocytes following injection with Synthetic poly-A RNA encoding NANOG-HA or a multi-antigen tagged cell lysate. (b) Western blot confirming complete KD of NANOG following translation MO injection at equivalent stage 10.5. (c) Validation of aberrant splicing in response to Nanog splice-morpholino and brightfield image of a stage 22 equivalent time point embryo following an injection of 80 ng Nanog splice MO at the 1 cell stage. (d) NANOG translation MO KD and rescue efficiencies from a (n = 25 per experimental condition). (e) HREM imaging of mid-gastrula embryos with and without NANOG translation MO KD at equivalent stage 10.5. Visible structures highlighted: involuting axial mesoderm (AM), ingressing ventral mesoderm (VM), archenteron (A), blastocoel (B). Scale bar, 1 mm (n = 2). (f) HREM imaging of stage 40 uninjected and stage matched NANOG translation MO KD + hNANOG rescue (dorsal view) (n = 2). (g) QPCR validation of key transcriptome findings (10 embryos pooled per experimental condition). Dots show individual data points. Asterisks represent the adjusted p-value obtained from unpaired one-sided multiple t tests, * = P ≤ 0.05, ** = = P ≤ 0.01, *** = P ≤ 0.001, *** = P ≤ 0.001, **** = P ≤ 0.0001, ns = P > 0.05. (h) Differentiation markers of uninjected at stage 28 and NANOG translation MO-depleted time point matched embryos (n = 10 × 3). Asterisks values same as described in h. The data underlying this figure are available in S1 Images and in S1 Data. (TIFF) [file pbio.3002121.s001.tiff]

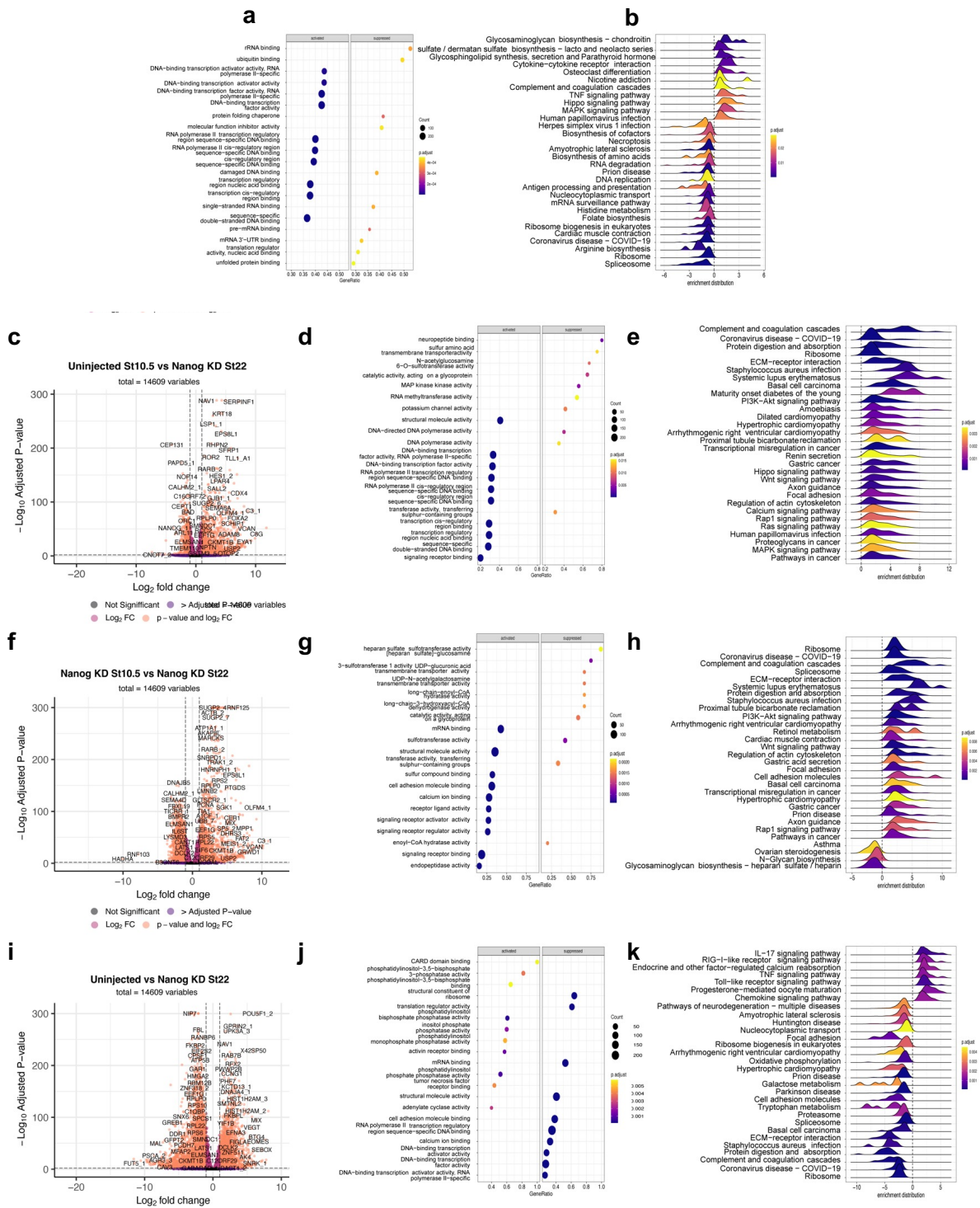

Supplement: S2 Fig — (a) Dot plots showing significantly enriched (padj <0.01). GO database biological process gene sets within NANOG translation MO KD DEGs at equivalent stage 10.5. (b) Ridgeline plots showing significantly enriched (padj <0.01) KEGG pathway gene sets within NANOG translation MO KD DEGs at equivalent stage 10.5. (c) Volcano plots showing significant DEGs in stage 22 equivalent stage NANOG translation MO KD embryos compared to stage 10.5 uninjected embryos. Vertical dotted line indicates a Log2 fold change of 1.5. Horizontal line indicates padj threshold of 0.01 on an -log10 scale. Orange points indicate significantly differentially expressed genes. (d, e) As with a and b, except stage 22 equivalent NANOG translation MO KD embryos are compared against stage 10.5 uninjected embryos. (g–i) As with c–e, except stage 22 equivalent NANOG translation MO KD embryos are compared against stage 10.5 equivalent NANOG translation MO KD embryos. (j–l) As with c–e, except NANOG translation MO KD embryos are at a stage equivalent to 22 and are compared against uninjected embryos at stage 22. The data underlying this figure are available in S1 Data. (PDF) [file pbio.3002121.s002.pdf]

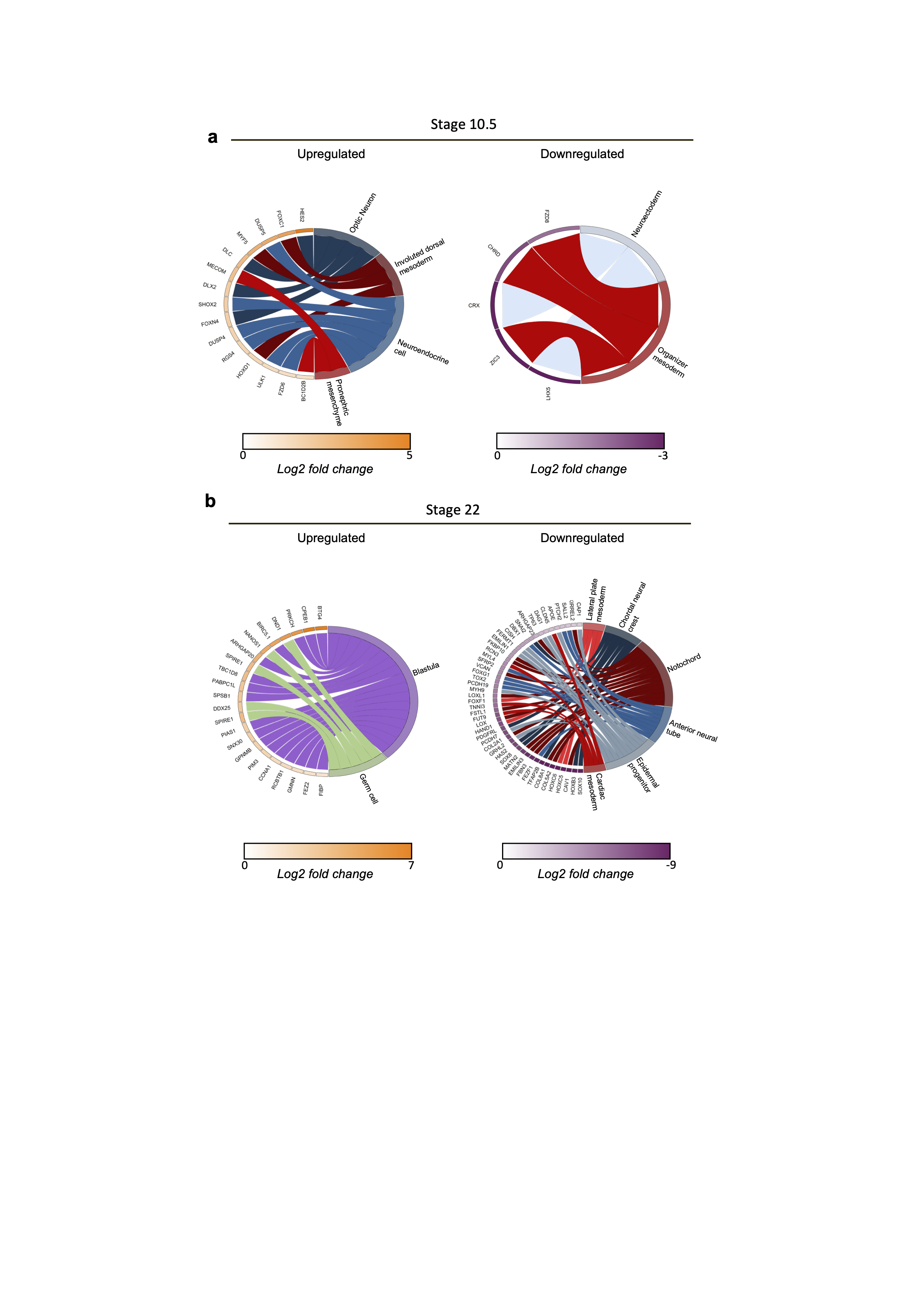

Supplement: S3 Fig — Chord diagrams showing the results of GSEA of amphibian cell type-specific markers in differentially expressed genes in NANOG translation MO KD. (a) NANOG translation morphant up-regulated DEGs are enriched for markers of optic neuron, involuted dorsal mesoderm, pronephric mesenchyme, and neuroendocrine cells at a stage equivalent to 10.5. Down-regulated DEGs at equivalent stage 10.5 are enriched for markers of neuroectoderm and organiser mesoderm. (b) Up-regulated DEGs in stage 22 equivalent NANOG translation MO KD embryos are enriched for markers of the blastula stage and germ cells. Down-regulated DEGs are enriched for markers of lateral plate, notochord, and cardiac mesoderm as well as markers of chordal neural crest cells, anterior neural tube, and epidermal progenitor cells. (TIFF) [file pbio.3002121.s003.tiff]

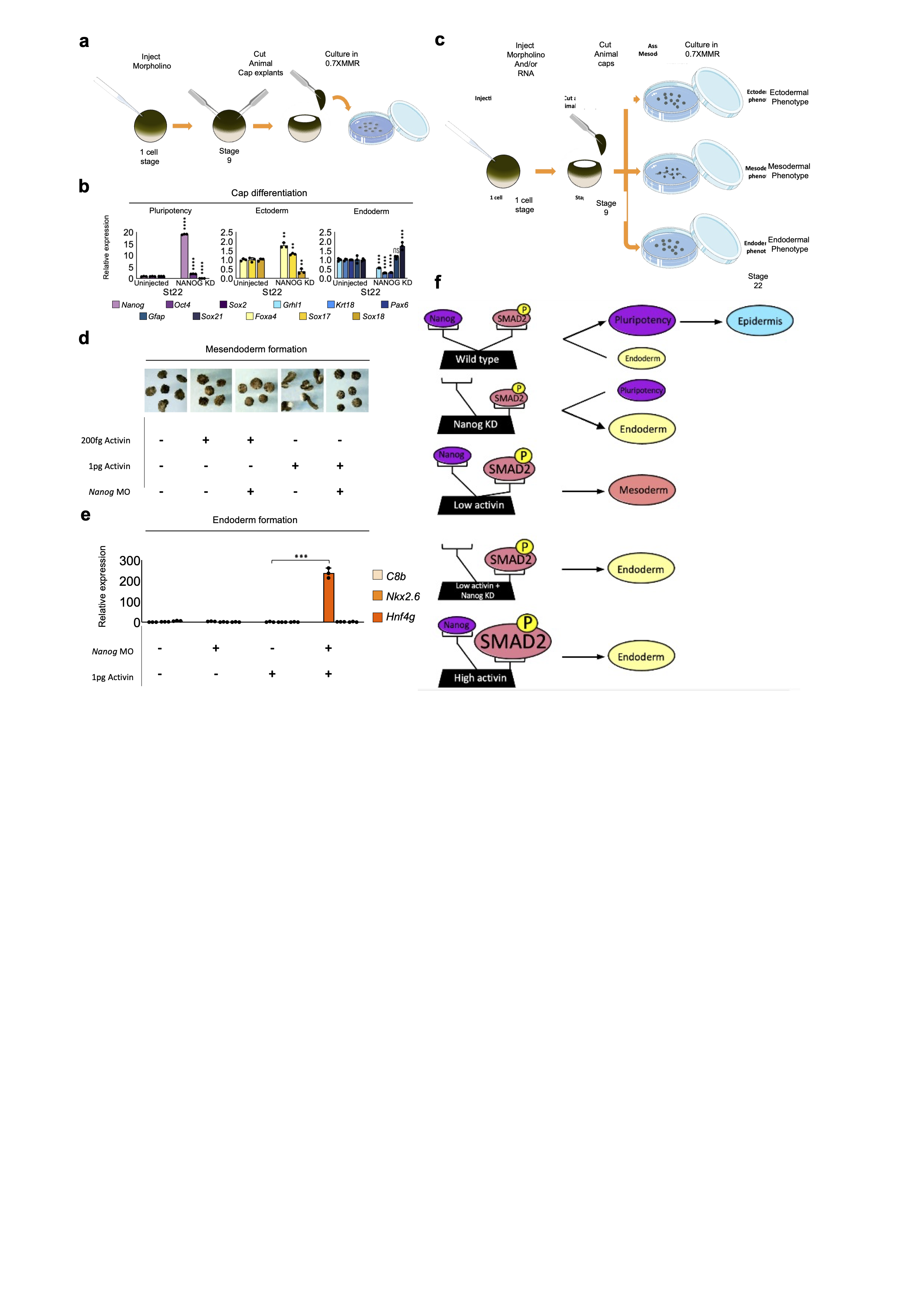

Supplement: S4 Fig — (a) Schematic of morpholino injection regime and animal cap assay. (b) Differentiation markers of uninjected and NANOG translation MO-depleted AC explants at a stage equivalent to 22 in uninjected whole embryo controls (n = 3, 15 explants pooled per experimental condition). Dots show individual data points. Asterisks represent the adjusted p-value obtained from unpaired one-sided multiple t tests, * = P ≤ 0.05, ** = = P ≤ 0.01, *** = P ≤ 0.001, *** = P ≤ 0.001, **** = P ≤ 0.0001, ns = P > 0.05. (c) Schematic of mesoderm induction animal cap assay. (d) Images of cultured uninjected and NANOG translation MO-depleted stage 22 equivalent cap explants following treatment with different activin concentrations corresponding to the bar graph in Fig 3D. (e) QPCR showing NANOG translation MO-depleted caps express foregut/hindgut markers but not mature foregut/hindgut markers in response to activin (n = 3, 15 explants pooled per experimental condition). Asterisks represent the adjusted p-value obtained from Tukey’s multiple comparisons test following one-way ANOVA. Asterisks values same as described in b. (f) Schematic: NANOG may act as a rheostat of SMAD2 activity. The data underlying this figure are available in S1 Images and in S1 Data. (TIFF) [file pbio.3002121.s004.tiff]

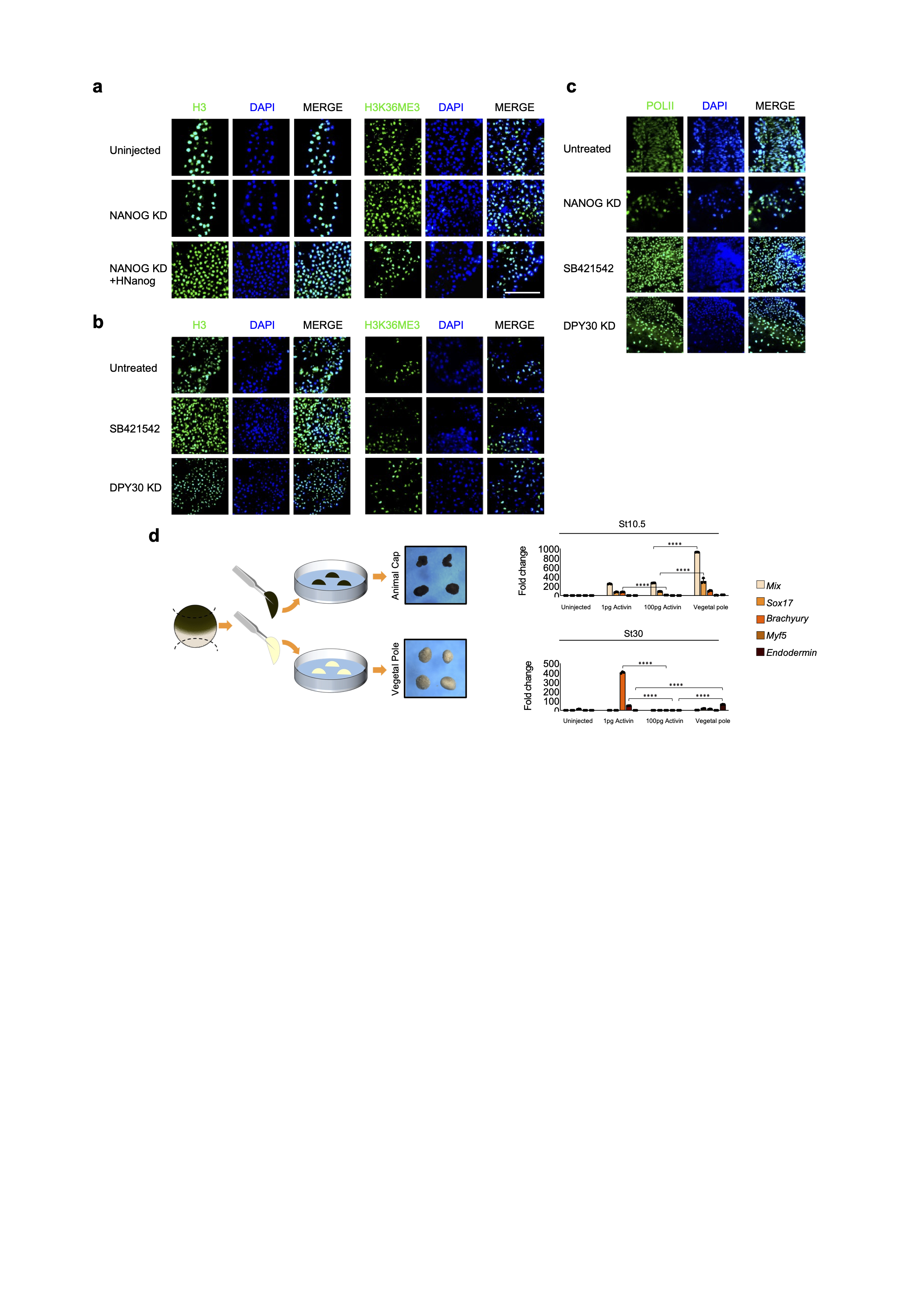

Supplement: S5 Fig — (a) Uninjected, NANOG translation MO depleted and hNANOG rescued AC explants cultured to equivalent stage 10.5 and stained for H3, H3K36me3, and DAPI (n = 3 per experimental condition). Scale bar, 60 μm. (b) Untreated, SB treated and DPY30 translation MO-depleted animal cap explants cultured to equivalent stage 10.5 and stained for H3, H3K36me3, and DAPI (n = 3 per experimental condition). Scale bar, 60 μm. (c) Untreated, NANOG translation MO depleted, SB treated, and DPY30 translation MO-depleted animal cap explants cultured to equivalent stage 10.5 and stained for phospho-POLII and DAPI (n = 3 per experimental condition). Scale bar, 60 μm. (d) Vegetal explants form endoderm in a cell-autonomous manner. QPCR of germ-layer markers of animal and vegetal explants compared with animal cap explants treated with different activin concentrations at time points equivalent to stages 10.5 and 30 in uninjected whole embryo controls (n = 3, 15 explants pooled per experimental condition). Dots show individual data points. Asterisks represent the adjusted p-value obtained from obtained from Tukey’s multiple comparisons test following one-way ANOVA, * = P ≤ 0.05, ** = = P ≤ 0.01, *** = P ≤ 0.001, *** = P ≤ 0.001, **** = P ≤ 0.0001, ns = P > 0.05. The data underlying this figure are available in S1 Images and in S1 Data. (TIFF) [file pbio.3002121.s005.tiff]

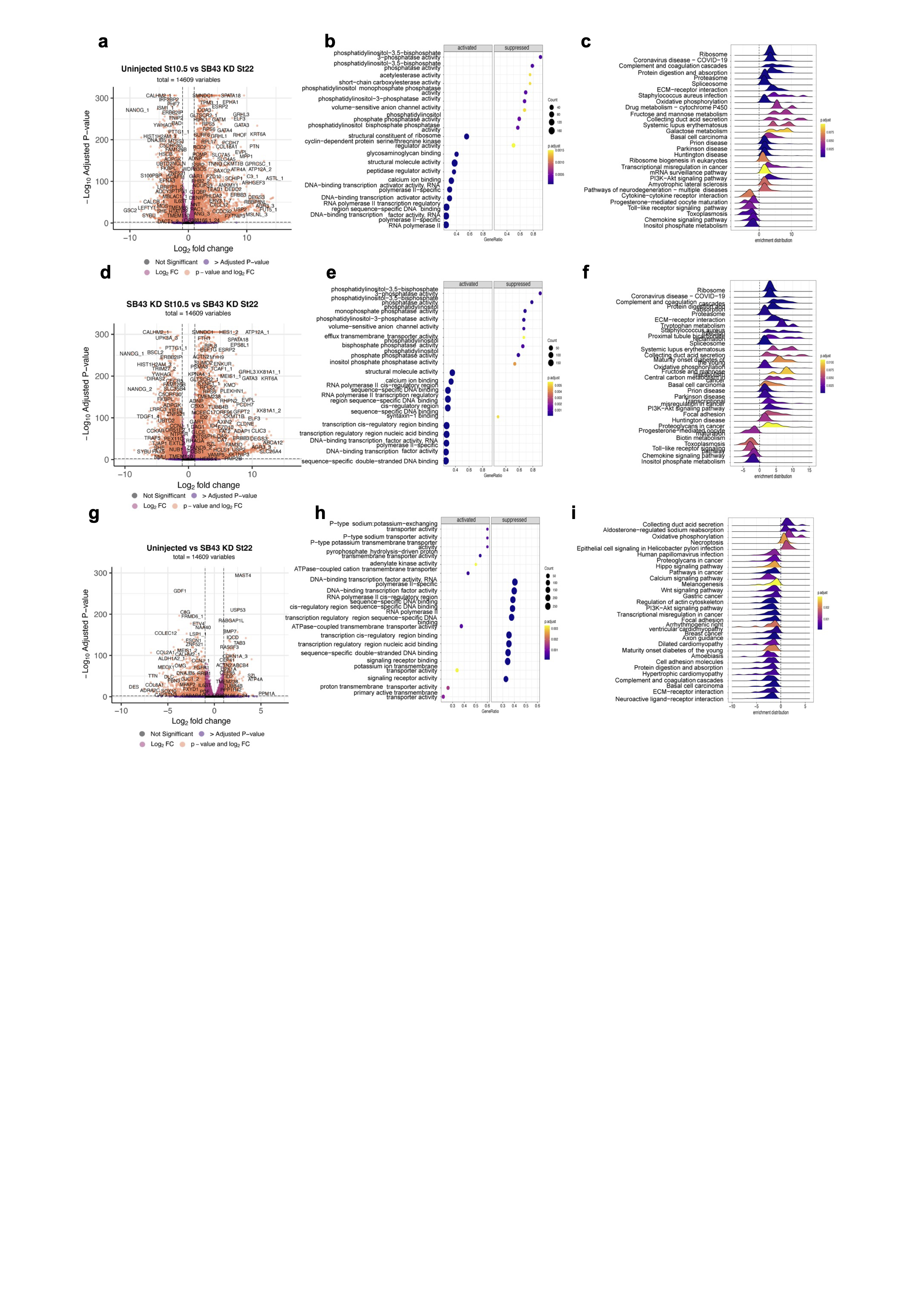

Supplement: S6 Fig — (a) Volcano plots showing significant DEGs in stage 22 equivalent SB-treated embryos compared to uninjected stage 10.5 embryos. Vertical dotted line indicates a Log2 fold change of 1.5. Horizontal line indicates padj threshold of 0.01 on an -log10 scale. Orange points indicate significantly differentially expressed genes. (b) Dot plots showing significantly enriched (padj <0.01) GO database biological process gene sets within SB-treated DEGs at equivalent stage 10.5. (c) Ridgeline plots showing significantly enriched (padj <0.01) KEGG pathway gene sets within SB-treated DEGs at equivalent stage 10.5. (d–f) As with a–c, except stage 22 equivalent SB-treated embryos are compared against stage 10.5 equivalent SB-treated embryos. (g, h, l) As with a–c, except SB-treated embryos are at a stage equivalent to 22 and are compared against uninjected embryos at stage 22. The data underlying this figure are available in S1 Data. (TIFF) [file pbio.3002121.s006.tiff]

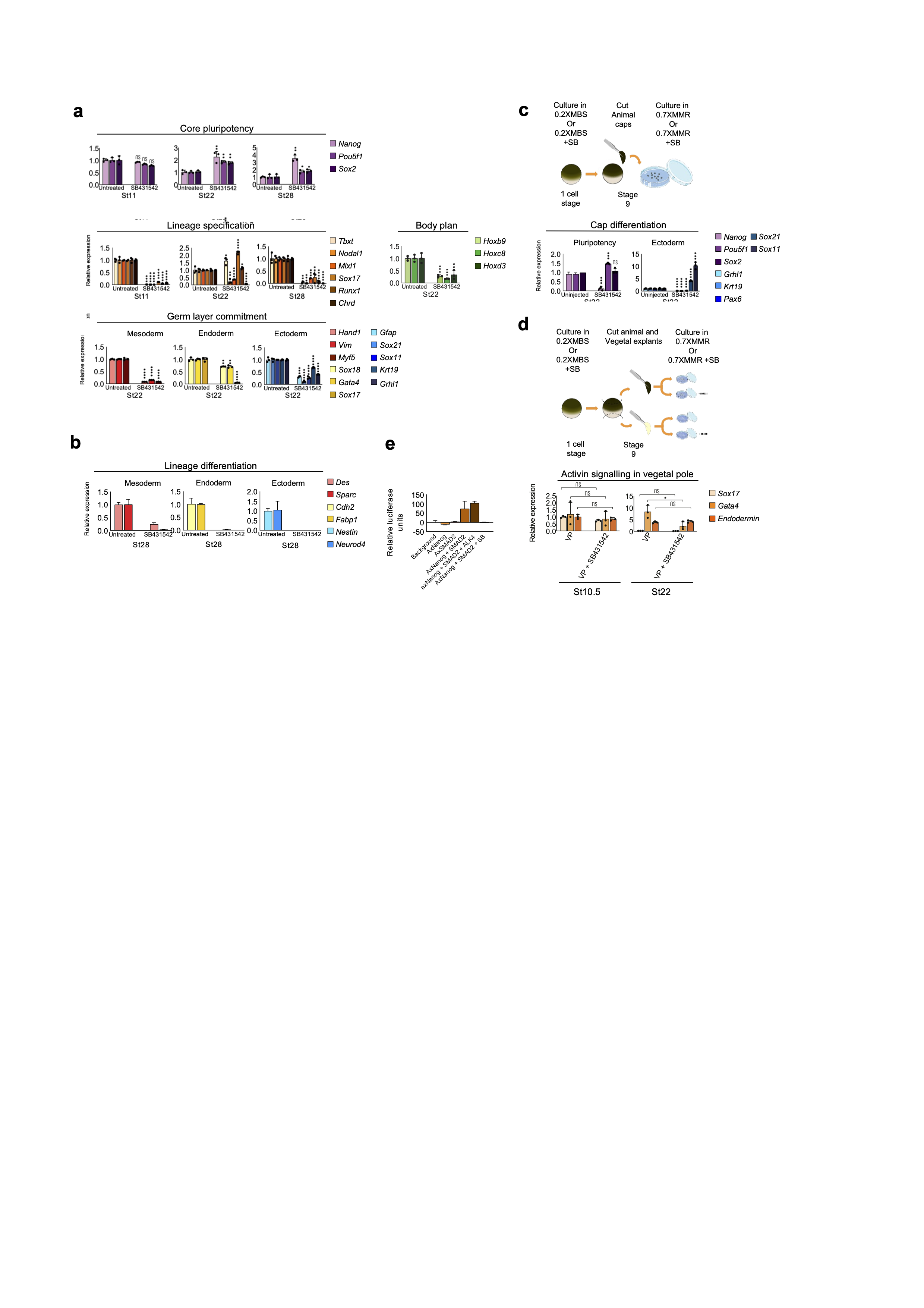

Supplement: S7 Fig — (a) QPCR validation of key transcriptome findings. Dots show individual data points. Asterisks represent the adjusted p-value obtained from unpaired one-sided multiple t tests, * = P ≤ 0.05, ** = = P ≤ 0.01, *** = P ≤ 0.001, *** = P ≤ 0.001, **** = P ≤ 0.0001, ns = P > 0.05. (b) QPCR analysis of late differentiation markers (n = 3, 10 embryos pooled per experimental condition). (c) Differentiation marker expression of uninjected and SB-treated AC explants at a stage equivalent to 22 in uninjected whole embryo controls (n = 3, 15 explants pooled per experimental condition). (d) Vegetal explants can form definitive endoderm even in the presence of SB431542 inhibitor. Vegetal explants from untreated or SB-treated embryos were cultured with or without SB431542 and assayed for endodermal markers at time points equivalent to stages 10.5 and 22 in uninjected whole embryo controls (n = 3, 15 explants pooled per experimental condition). Asterisks represent the adjusted p-value obtained from Tukey’s multiple comparisons test following one-way ANOVA. Asterisks values same as in a–c. (e) Luciferase complementation assay (n = 3). Axolotl NANOG can physically interact with axolotl SMAD2, interactions are increased in the presence of constitutively active ALK4, binding is disrupted with SB treatment. The data underlying this figure are available in S1 Data. (TIFF) [file pbio.3002121.s007.tiff]

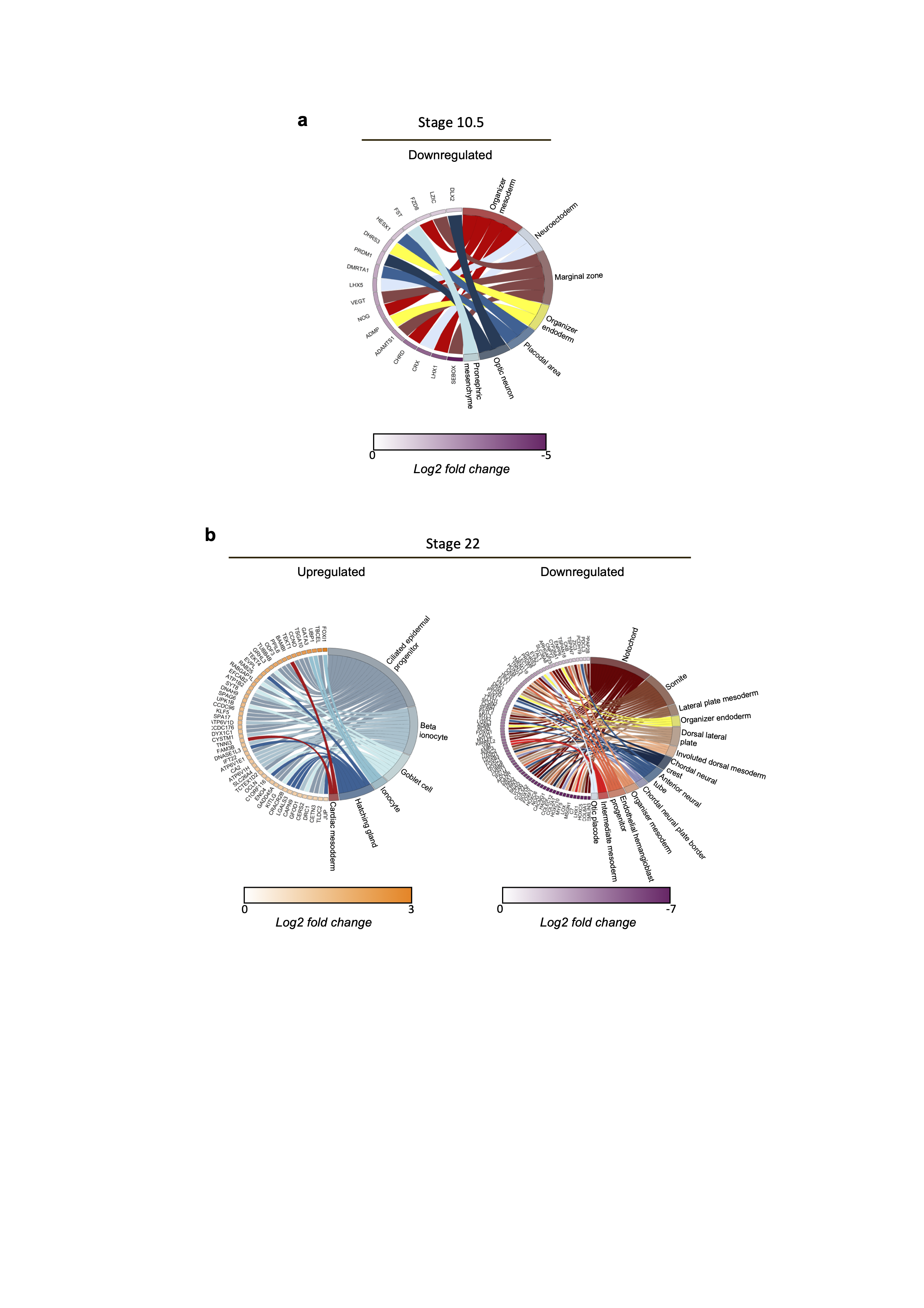

Supplement: S8 Fig — (a) Chord diagrams showing the results of GSEA of amphibian cell type-specific markers in differentially expressed genes following SB treatment. (a) SB-treated embryo down-regulated DEGs are enriched for markers of organiser and marginal zone mesoderm as well as organiser endoderm, neuroectoderm, placodal area, optic neuron, and pronephric mesenchyme at equivalent stage 10.5. (b) Up-regulated DEGs in SB-treated embryos are enriched for markers of the ciliated epidermal progenitors, beta ionocytes, goblet cells, ionocytes, hatching gland cells, and cardiac mesoderm at equivalent stage 22. Down-regulated DEGs are enriched for markers of notochord, somitic, lateral plate, dorsal, intermediate, and organiser mesoderm as well as markers of organiser endoderm, chordal neural crest cells, anterior neural tube, otic placode, and endothelial hemangioblast cells. The data underlying this figure are available in S1 Data. (TIFF) [file pbio.3002121.s008.tiff]

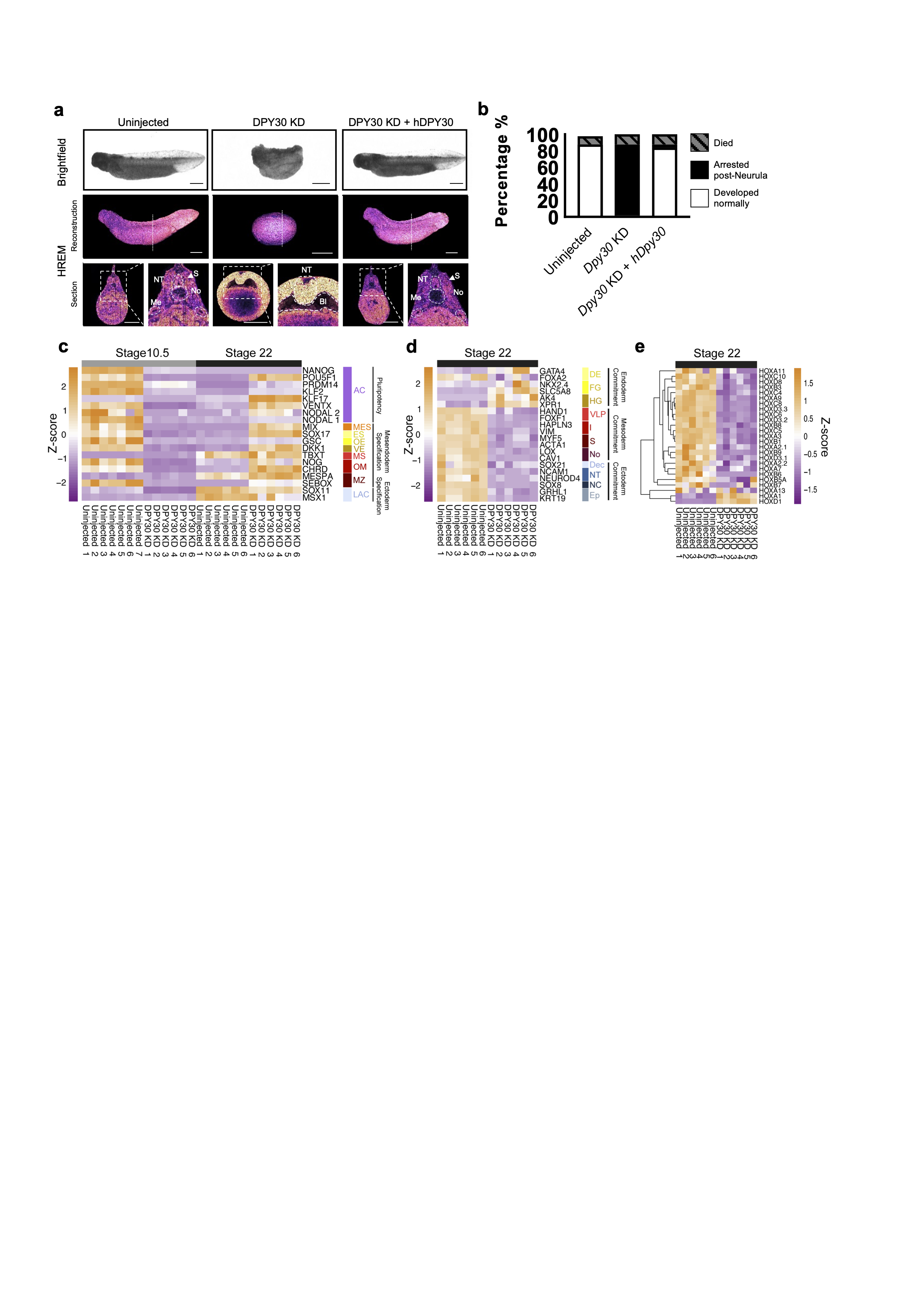

Supplement: S9 Fig — (a) DPY30 translation MO KD arrests development post-gastrulation. Brightfield and HREM images of uninjected and DPY30 translation MO-depleted embryos. Dotted line marks plane of section reconstruction. Dashed line delimits visible structures: Somites (S), Neural tube (NT) Notochord (No), Mesonephric ducts (Me), Blastocoel (B) (n = 2). Scale bar, 1 mm. (b) DPY30 KD and rescue efficiencies (n = 30 per experimental condition). (c, d) Gene expression of key cell type marker genes at equivalent stages 10.5 and 22 in uninjected (n = 7 and 6, respectively) and DPY30 translation MO KD (n = 6 and 6, respectively) embryos. Cell types: animal cap (AC), mesendoderm specification (general) (MES), endoderm specification (general) (ES), organiser endoderm (OE), vegetal endoderm (VE), mesoderm specification (general) (MS), organiser mesoderm (OM), marginal zone (MZ), definitive endoderm (general) (DE), foregut (FG), hindgut (HG), ventral-lateral plate (VLP), intermediate mesoderm (I), somite (S), notochord (No), definitive ectoderm (general) (Dec), neural tube (NT), neural crest (NC), epidermal progenitors (EP). (e) Gene expression of Hox gene family members in DPY30 translation MO KD embryos at a time point equivalent to stage 22. The data underlying this figure are available in S1 Images and in S1 Data. (TIFF) [file pbio.3002121.s009.tiff]

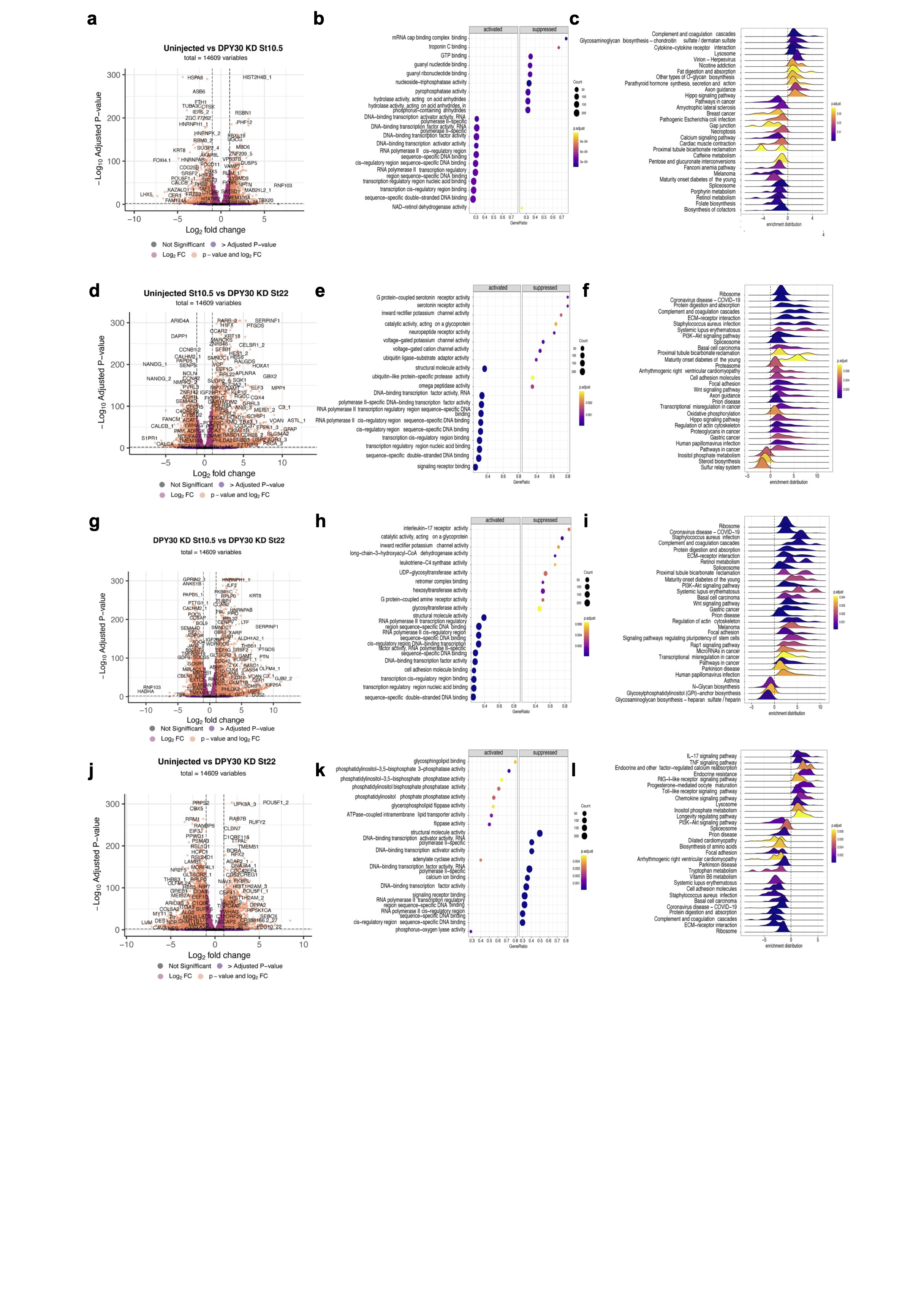

Supplement: S10 Fig — (a) Volcano plots showing significant DEGs in DPY30 translation MO KD embryos compared to equivalent stage 10.5 uninjected embryos. Vertical dotted line indicates a Log2 fold change of 1.5. Horizontal line indicates padj threshold of 0.01 on an -log10 scale. Orange points indicate significantly differentially expressed genes. (b) Dot plots showing significantly enriched (padj <0.01) GO database biological process gene sets within DPY30 translation MO KD DEGs at equivalent stage 10.5. (c) Ridgeline plots showing significantly enriched (padj <0.01) KEGG pathway gene sets within DPY30 translation MO KD DEGs at equivalent stage 10.5. (d–f) As with a–c, except stage 22 equivalent DPY30 translation MO KD embryos are compared against stage 10.5 uninjected embryos. (g–i) As with a–c, except stage 22 equivalent DPY30 translation MO KD embryos are compared against stage 10.5 equivalent DPY30 KD embryos. (j–l) As with a–c, except DPY30 translation MO KD embryos are at a stage equivalent to 22 and are compared against uninjected embryos at stage 22. The data underlying this figure can be found in S1 Data. (TIFF) [file pbio.3002121.s010.tiff]

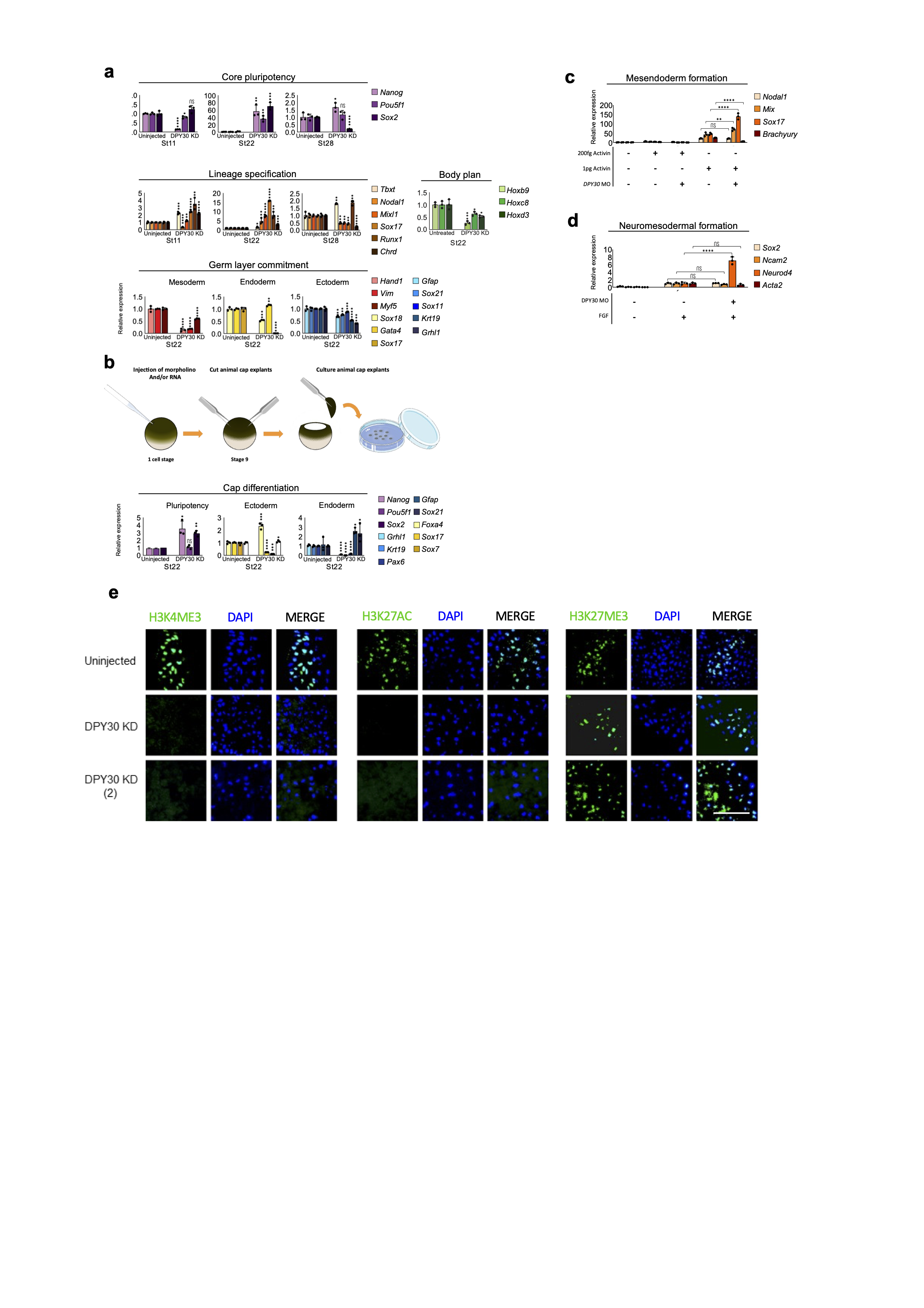

Supplement: S11 Fig — (a) QPCR of genes representative of key lineages. Dots show individual data points (10 embryos pooled per experimental condition). Asterisks represent the adjusted p-value obtained from unpaired one-sided multiple t tests, * = P ≤ 0.05, ** = = P ≤ 0.01, *** = P ≤ 0.001, *** = P ≤ 0.001, **** = P ≤ 0.0001, ns = P > 0.05. (b) Differentiation markers of uninjected and Nanog depleted AC explants at stage equivalent to 22 in uninjected controls (n = 3, 15 explants pooled per experimental condition). (c) DPY30 translation MO depletion prevents mesodermal but not endodermal differentiation in response to activin. QPCR of germ-layer markers of uninjected and DPY30 translation MO-depleted stage 20 equivalent caps following treatment with different activin concentrations (n = 3, 15 explants pooled per experimental condition). Asterisks represent the adjusted p-value obtained from Tukey’s multiple comparisons test following one-way ANOVA. Asterisks values same as described in a and b. (d) DPY30 translation MO depletion reduces mesodermal gene expression and increased neuronal gene expression in response to FGF. QPCR of germ-layer markers of uninjected and DPY30 translation MO-depleted stage 20 equivalent caps following treatment with FGF (15 explants pooled per experimental condition, individual points represent technical repeats). Statistics and asterisks values same as described in d. (e) Uninjected, DPY30 translation MO 1 and 2 depleted embryos and hDPY30 rescued animal cap explants cultured to equivalent stage 10.5 and stained for H3K4me3, H3K27ac, H3K27me3, and DAPI (n = 3). Scale bar: 60 μm. The data underlying this figure are available in S1 Images and in S1 Data. (TIFF) [file pbio.3002121.s011.tiff]

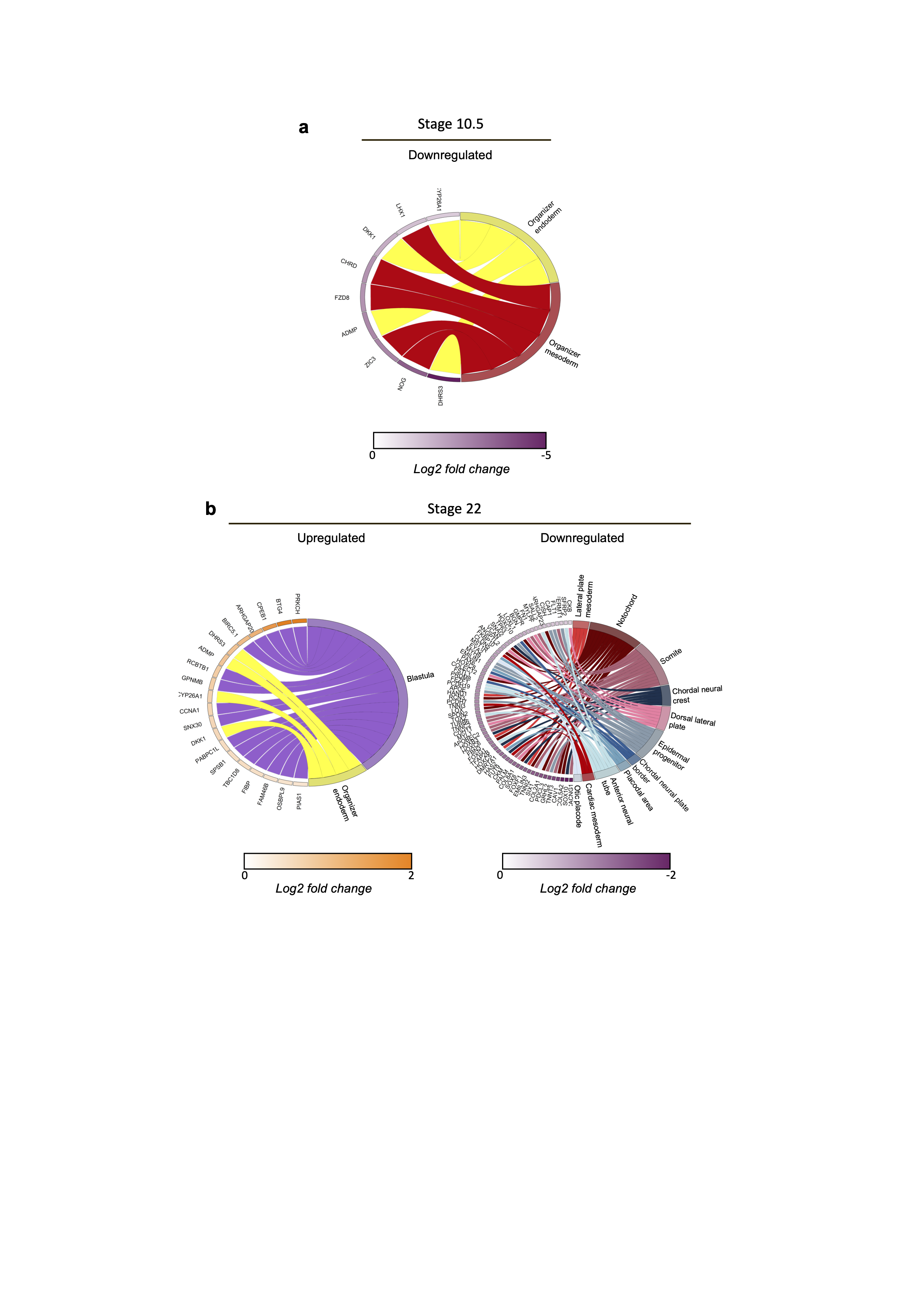

Supplement: S12 Fig — Chord diagrams showing the results of GSEA of amphibian cell type-specific markers in differentially expressed genes following DPY30 translation MO KD. (a) DPY30 translation MO morphant down-regulated DEGs are enriched for markers of organiser mesoderm and endoderm at equivalent stage 10.5. (b) Up-regulated DEGs in DPY30 translation MO KD stage 22 equivalent embryos are significantly enriched for markers of blastula cells and organiser endoderm. Down-regulated DEGs are enriched for markers of lateral plate, notochord, somitic, dorsal lateral plate, and cardiac mesoderm as well as markers of chordal neural crest cells, anterior neural tube, otic placode, chordal neural plate border, anterior neural tube, and epidermal progenitor cells. (TIFF) [file pbio.3002121.s012.tiff]

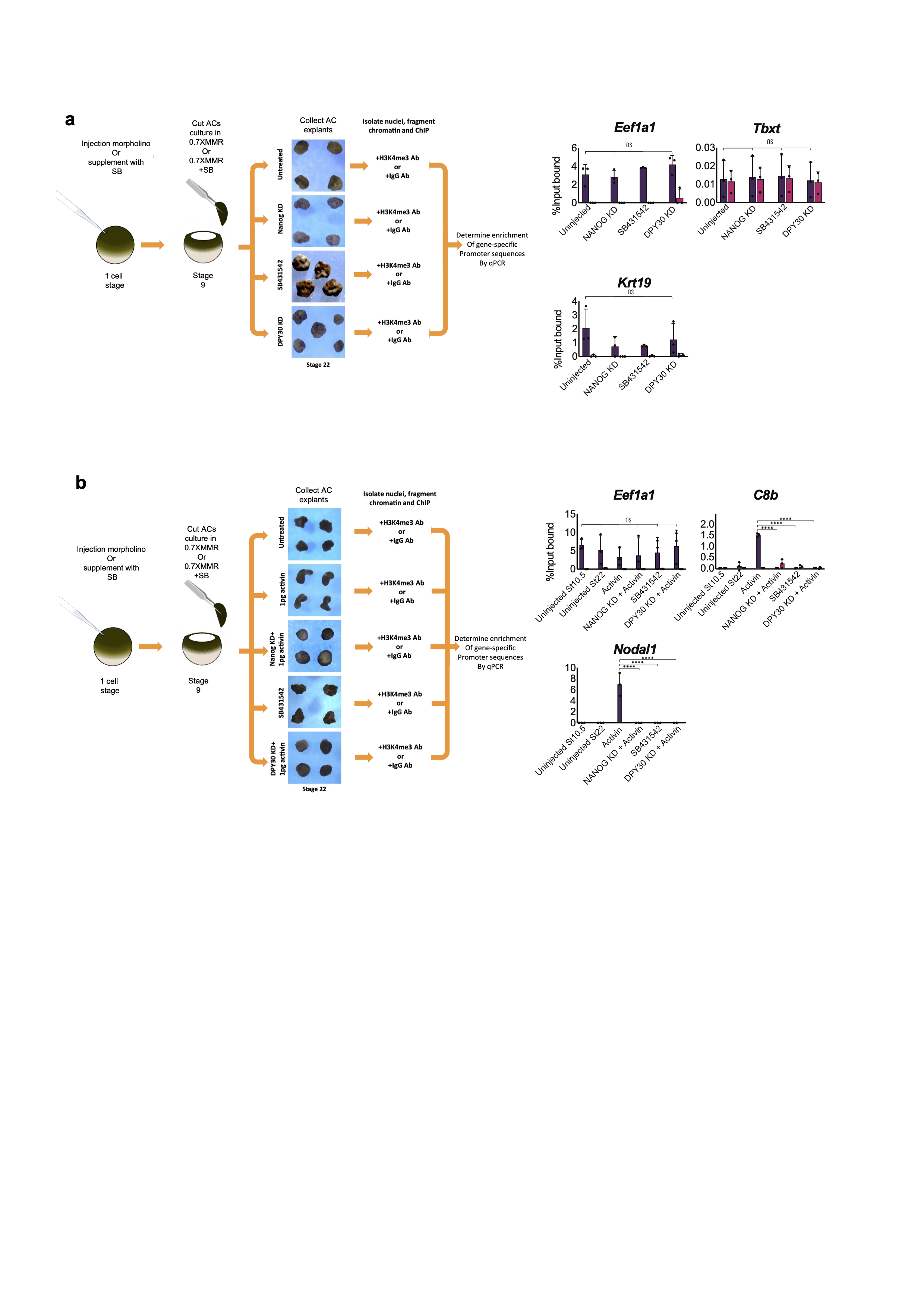

Supplement: S13 Fig — (a) H3K4me3 ChIP of equivalent stage 10.5 uninjected, NANOG translation MO KD, DPY30 translation MO KD, and SB-treated caps followed by qPCR using probes directed at gene promoter regions 50 pooled caps per experimental condition, data points represent technical repeats. Dots show individual data points. Asterisks represent the adjusted p-value obtained from obtained from Tukey’s multiple comparisons test following one-way ANOVA, * = P ≤ 0.05, ** = = P ≤ 0.01, *** = P ≤ 0.001, *** = P ≤ 0.001, **** = P ≤ 0.0001, ns = P > 0.05. (b) H3K4me3 ChIP-qPCR of equivalent stages 10.5 and 22 uninjected caps, stage 22 equivalent Activin treated caps with and without NANOG and DPY30 translation MO depletion, as well as stage 22 equivalent SB-treated caps. Statistics and asterisk values same as described in a. The data underlying this figure are available in S1 Images and in S1 Data. (TIFF) [file pbio.3002121.s013.tiff]

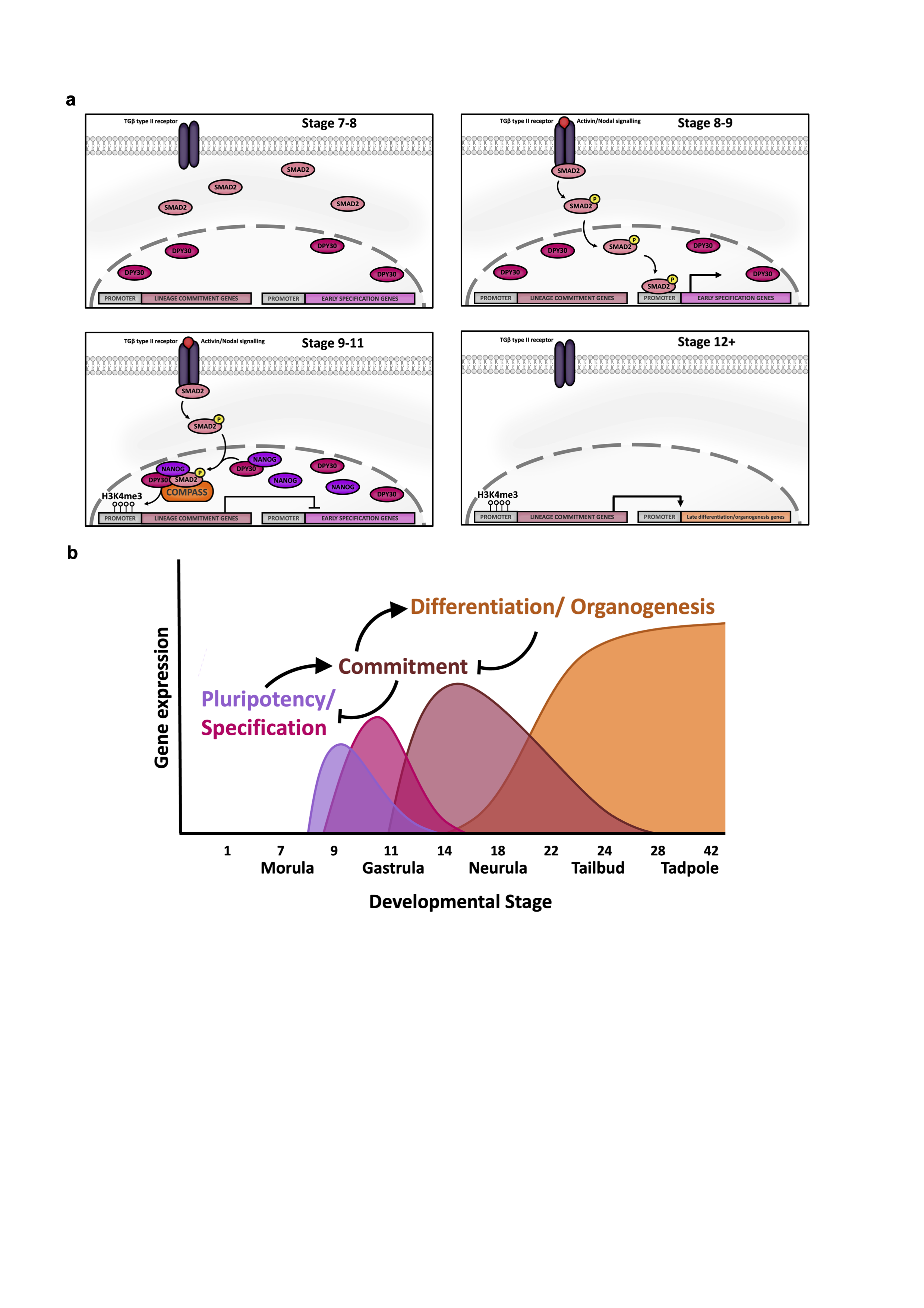

Supplement: S14 Fig — (a, b) Schematics of hypothesis outline. (TIFF) [file pbio.3002121.s014.tiff]
