# Supplementary material for: NANOG is required to establish the competence for germ-layer differentiation in the basal tetrapod axolotl
Source: PLoS Biol. 2023 Jun 14;21(6):e3002121. doi: 10.1371/journal.pbio.3002121 (PMC10599592; doi:10.1371/journal.pbio.3002121)

Raw image for Fig. 2a Uninjected stage 40 Whole embryo brightfeild

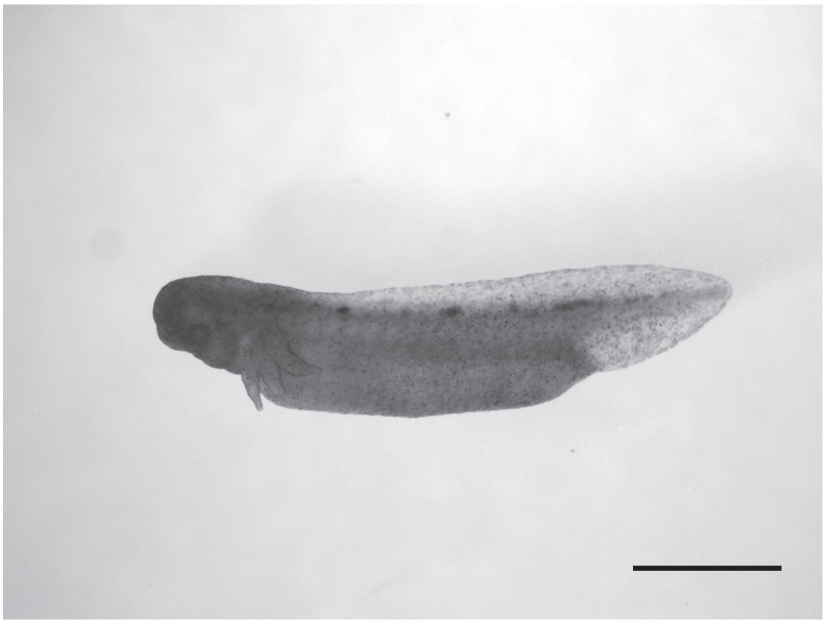

Raw image for Fig. 2a NANOG KD stage 40 timepoint equivalent Whole embryo brightfeild

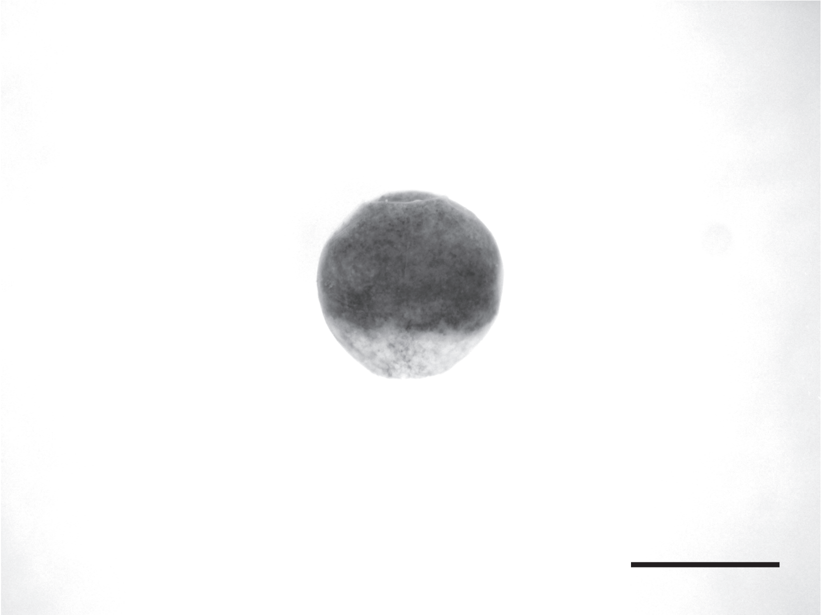

Raw image for Fig. 2a NANOG KD + HNANOG stage 40 timepoint equivalent Whole embryo brightfeild

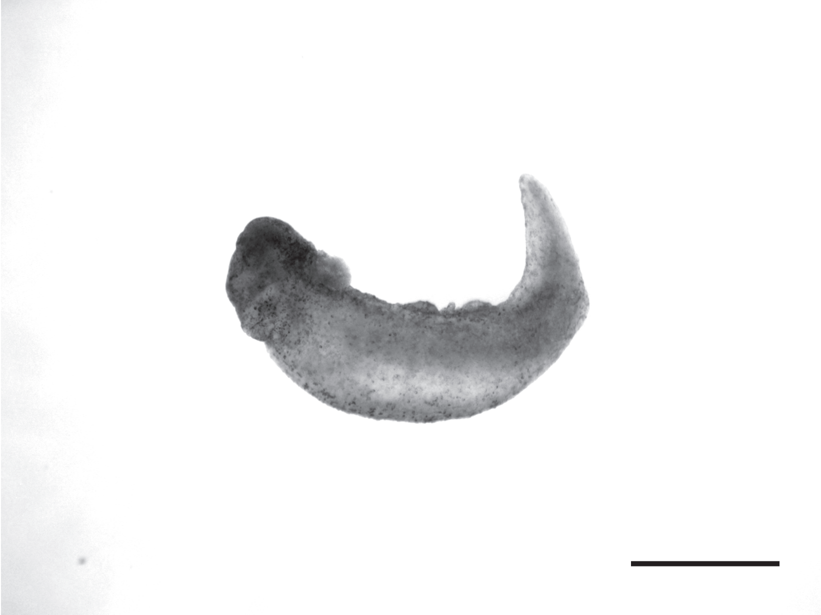

Raw image for Fig. 2a Uninjected stage 40 HREM 3D reconstruction

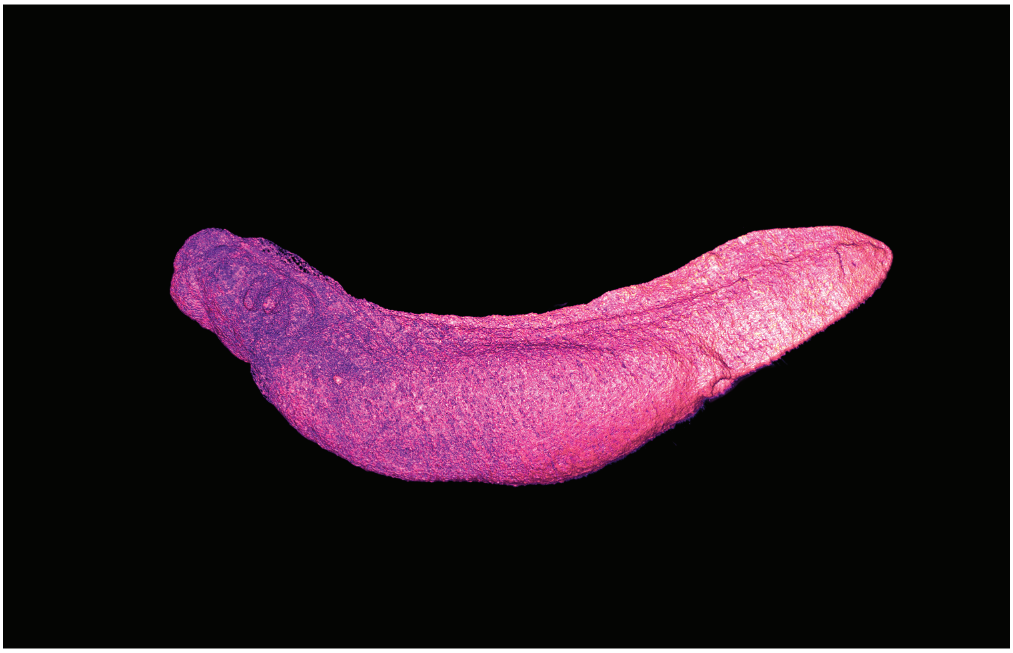

Raw image for Fig. 2a NANOG KD stage 40 timepoint equivalent HREM 3D reconstruction

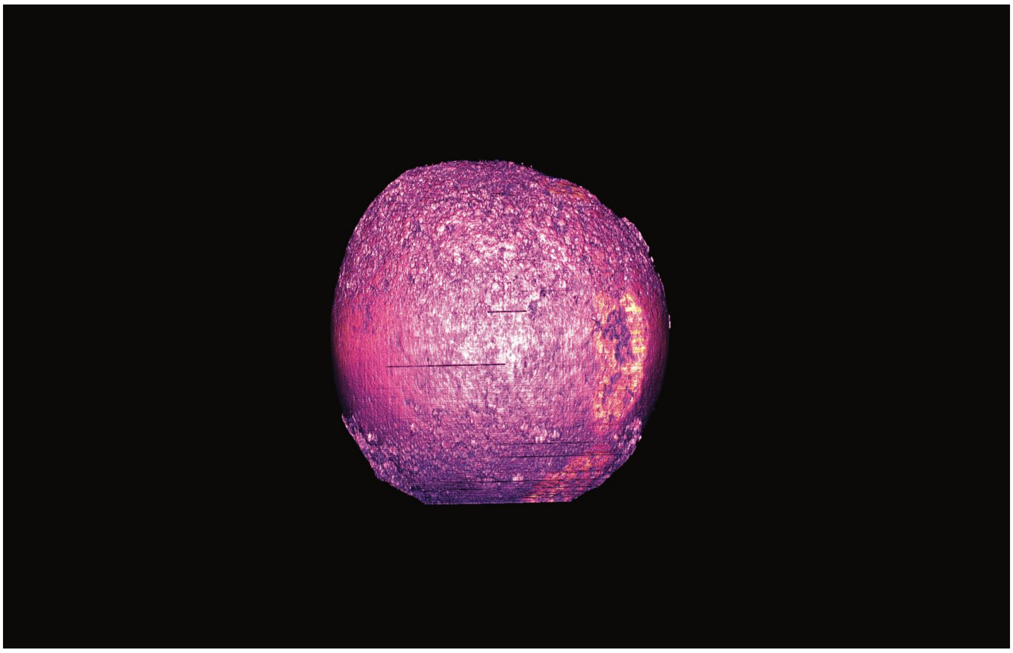

Raw image for Fig. 2a NANOG KD + HNANOG stage 40 timepoint equivalent HREM 3D reconstruction

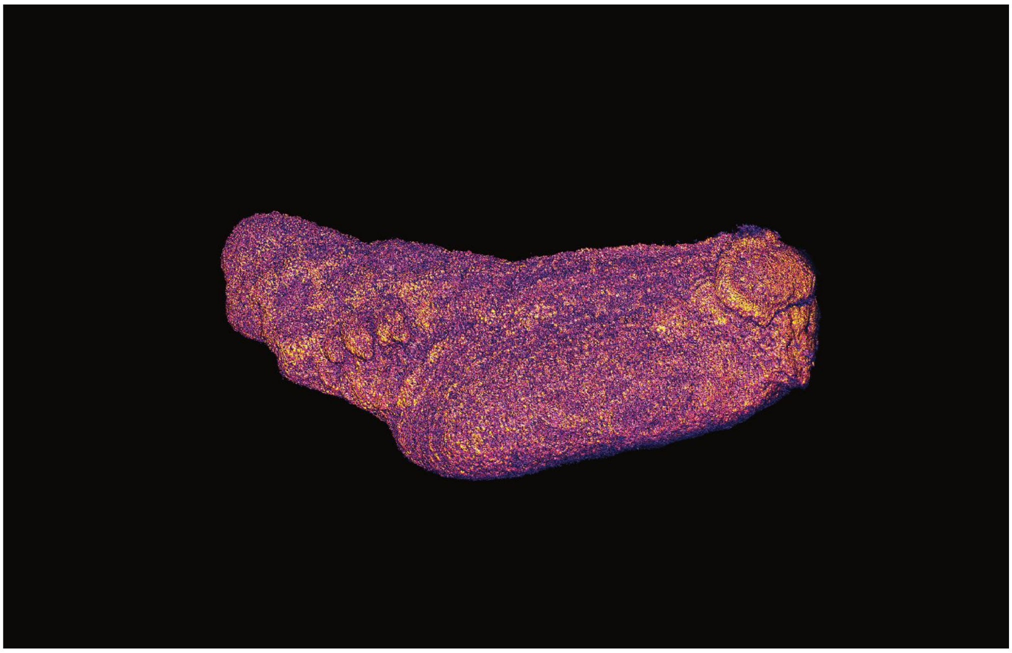

Raw image for Fig. 2a Uninjected stage 40 HREM 3D reconstruction sagital section

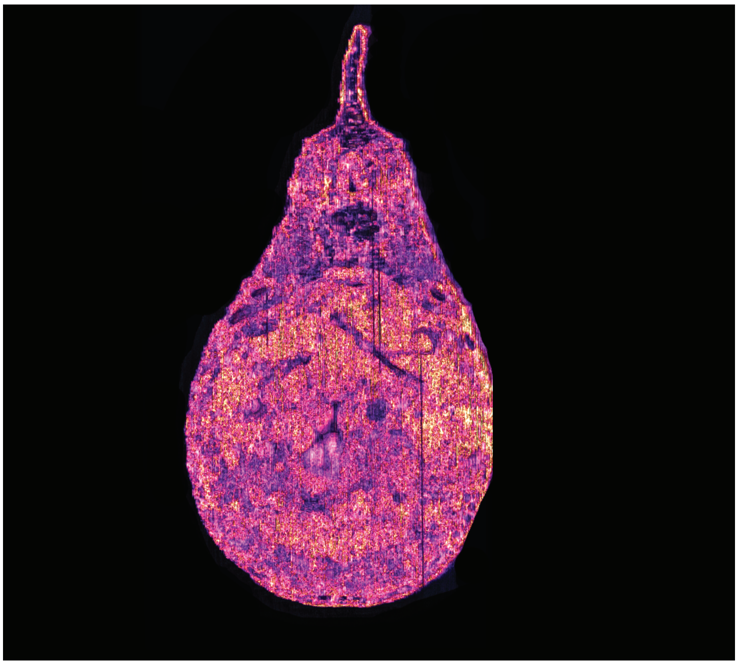

Raw image for Fig. 2a NANOG KD stage 40 timepoint equivalent HREM 3D reconstruction sagital section

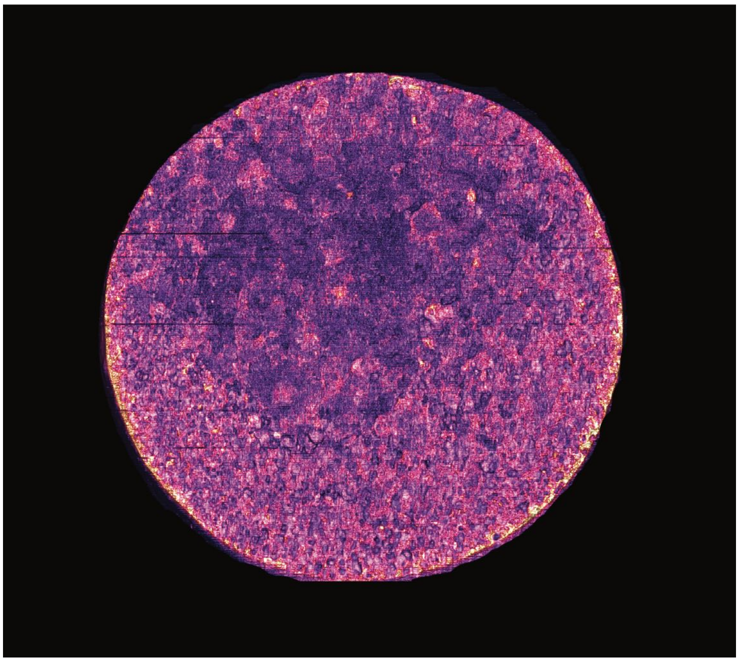

Raw image for Fig. 2a NANOG KD + HNANOG stage 40 timepoint equivalent HREM 3D reconstruction sagital

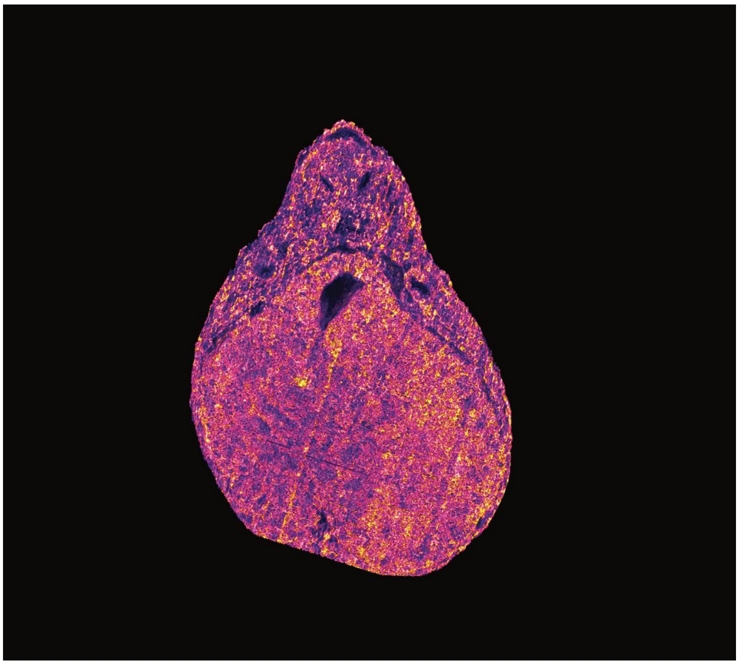

Raw image for Fig. 3f Uninjected stage 10.5 animal cap stained for H3K4me3

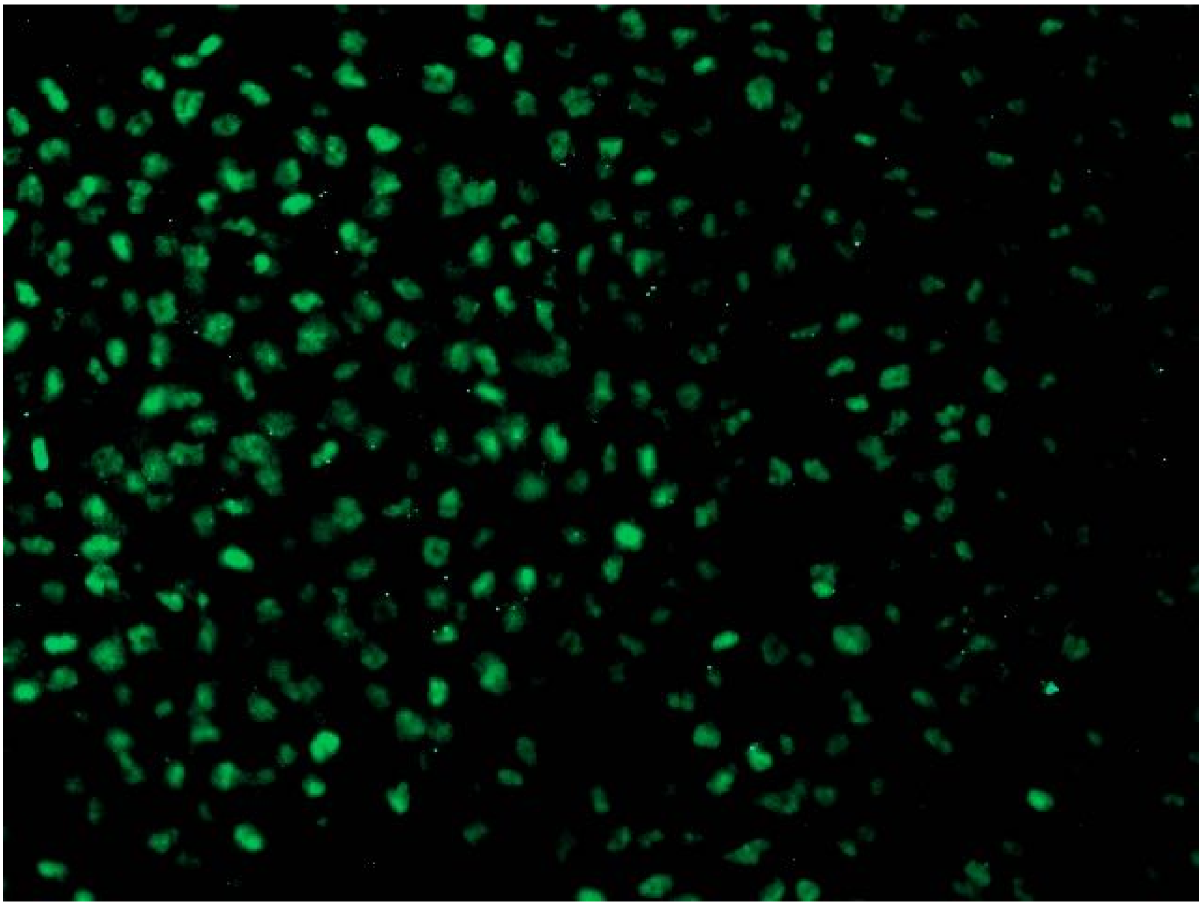

Raw image for Fig. 3f Uninjected stage 10.5 animal cap stained for DAPI

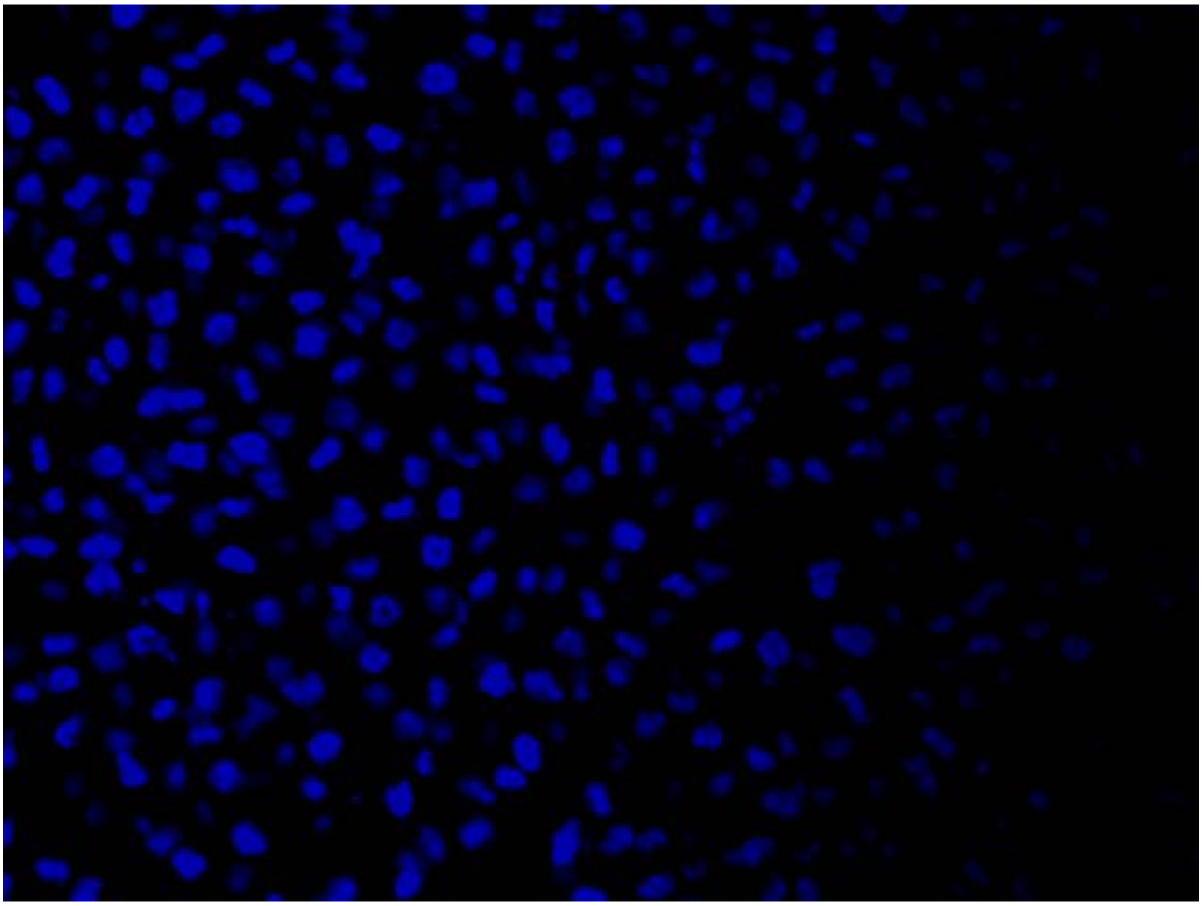

Raw image for Fig. 3f NANOG KD stage 10.5 animal cap stained for H3K4me3

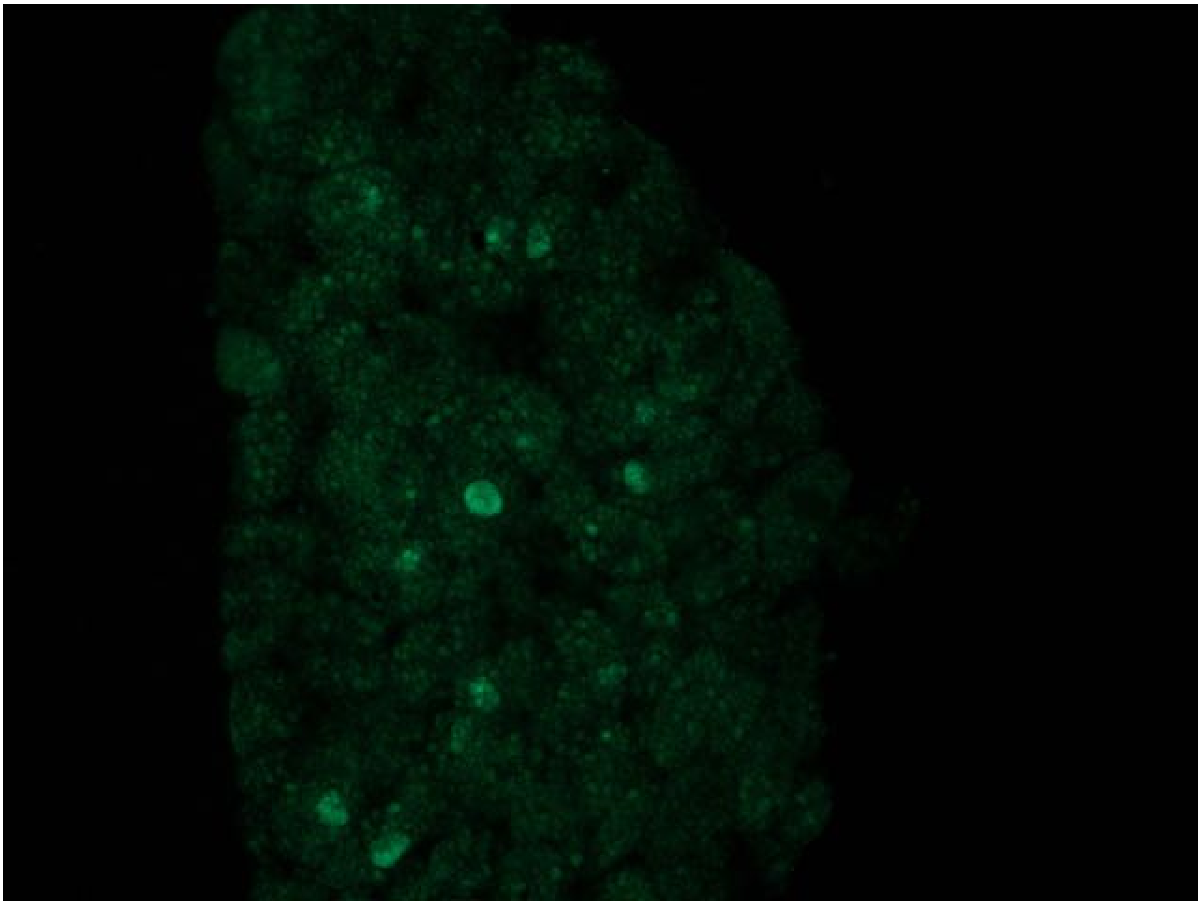

Raw image for Fig. 3f NANOG KD stage 10.5 animal cap stained for DAPI

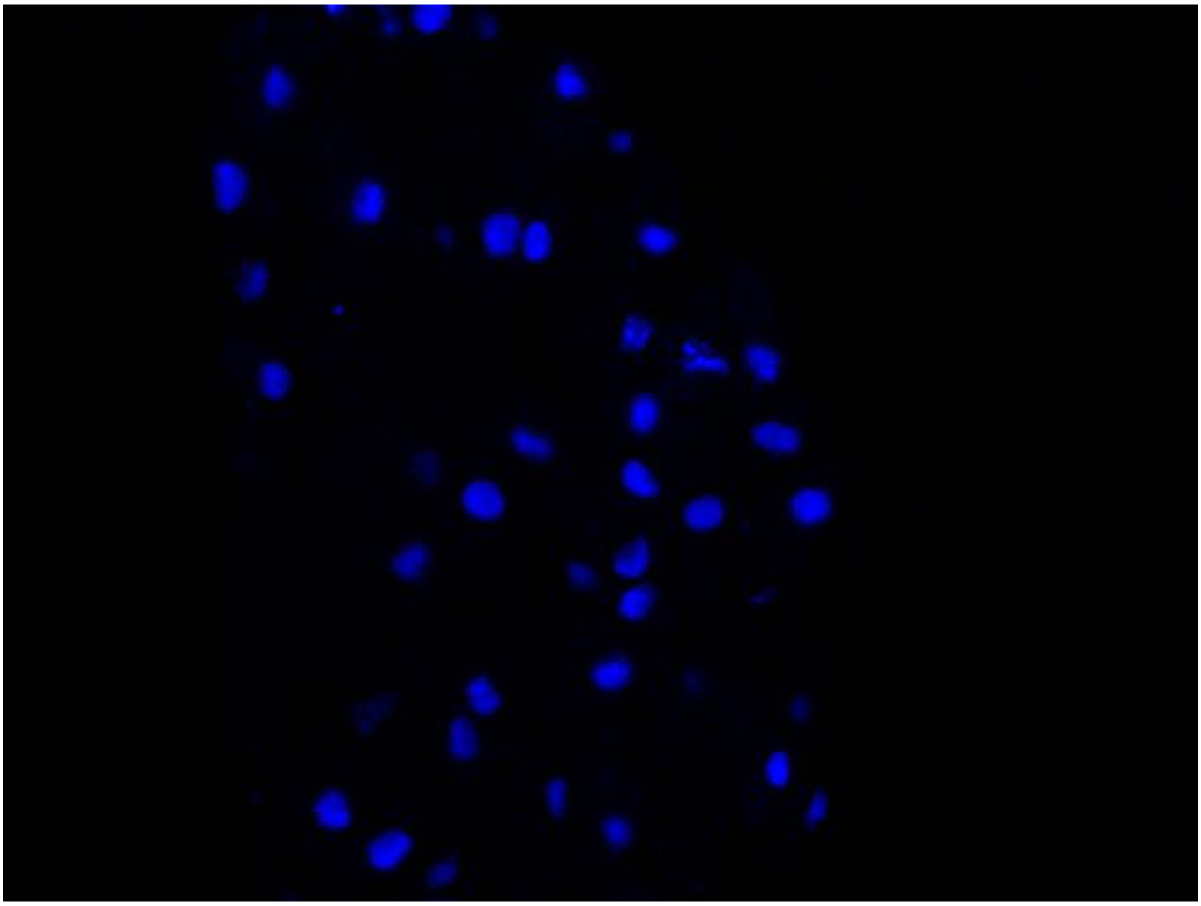

Raw image for Fig. 3f NANOG KD + HNANOG 10.5 animal cap stained for H3K4me3

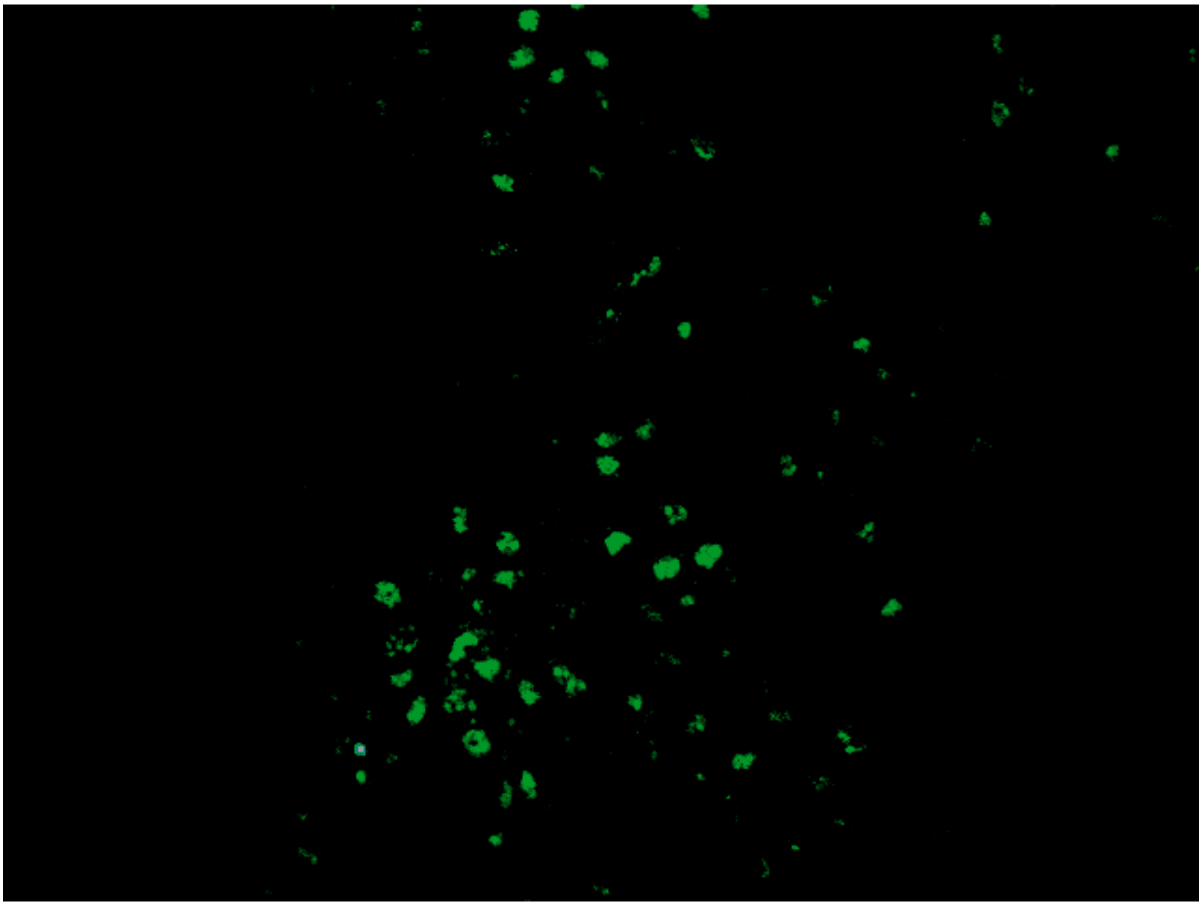

Raw image for Fig. 3f NANOG KD + HNANOG 10.5 animal cap stained for DAPI

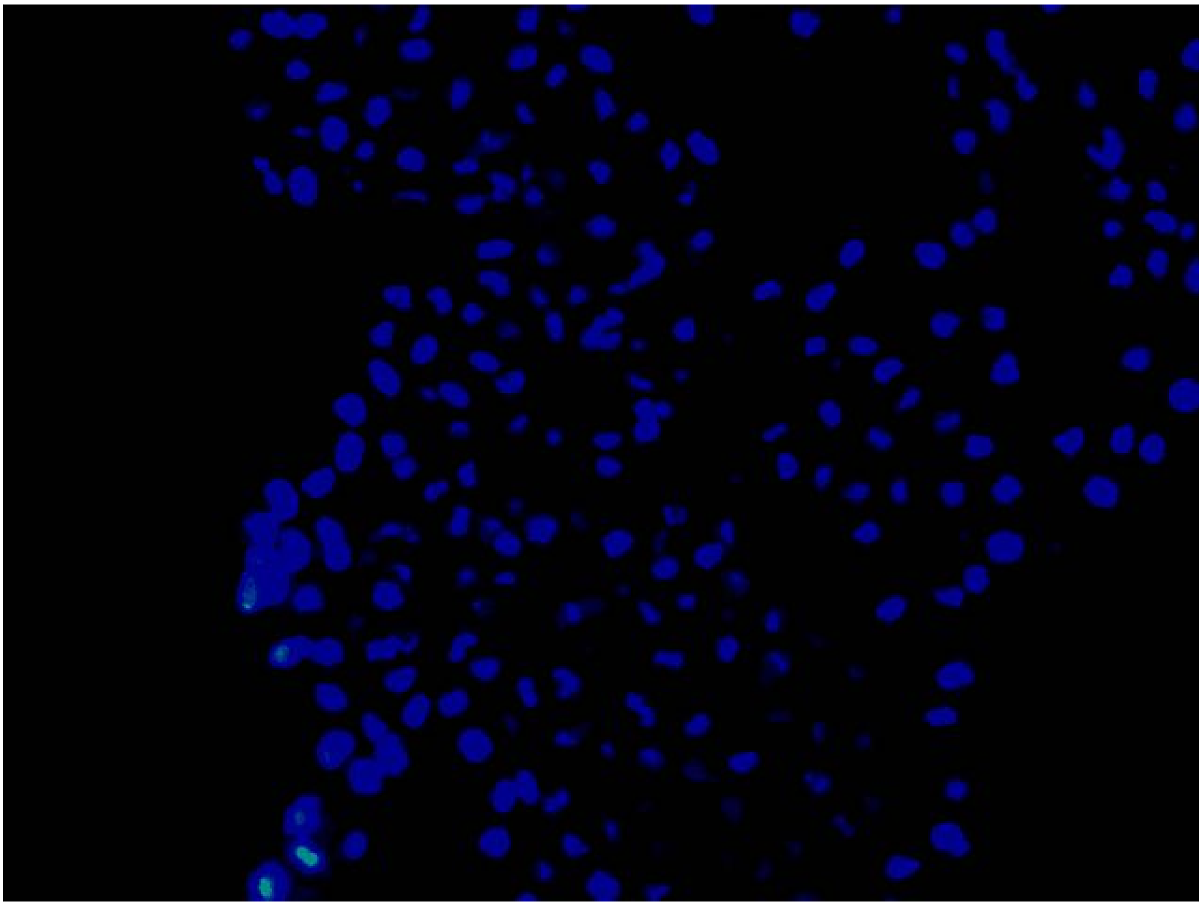

Raw image for Fig. 5c Untreated stage 10.5 animal cap stained for H3K27ac

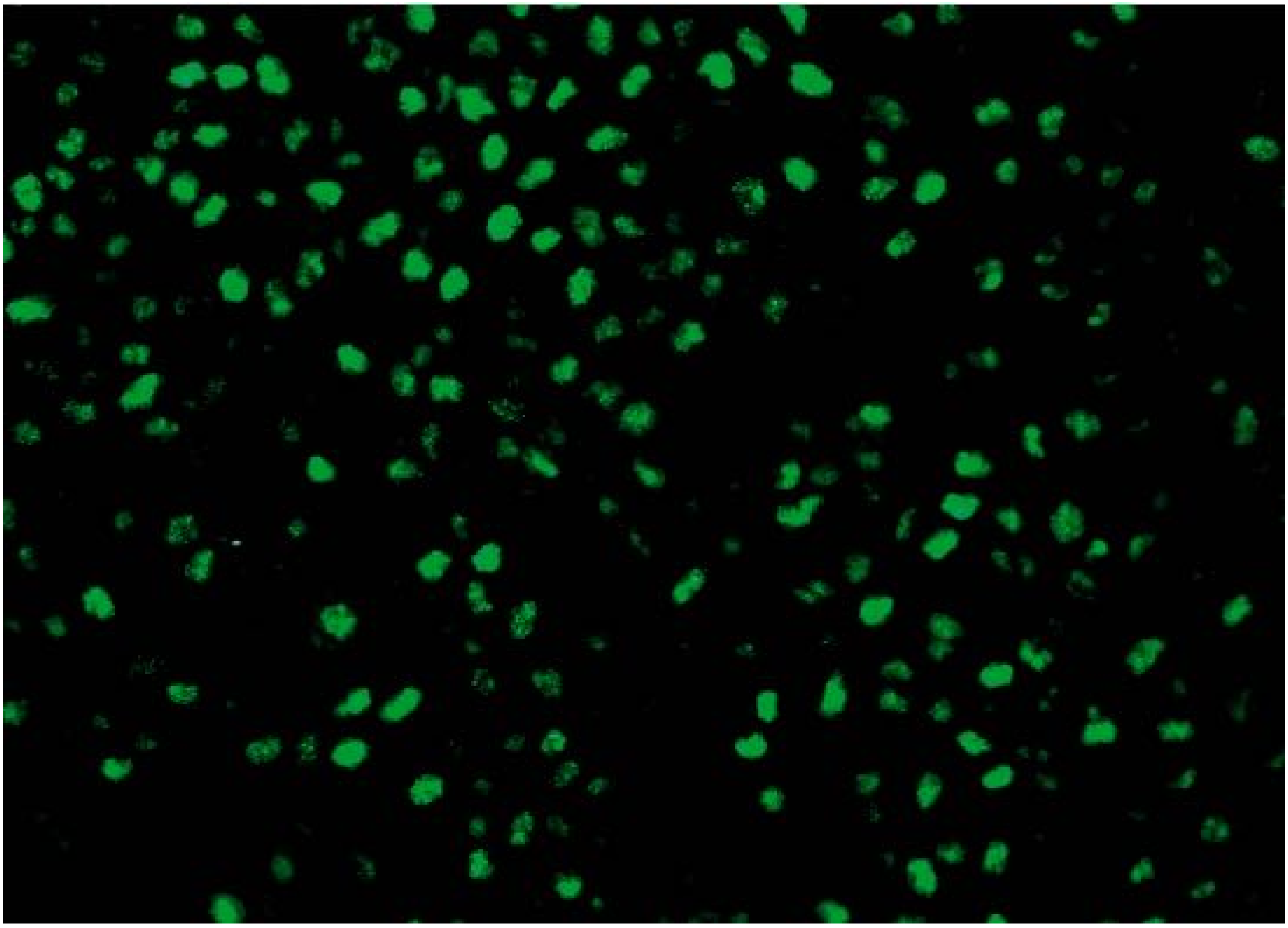

Raw image for Fig. 5c Untreated stage 10.5 animal cap stained for DAPI

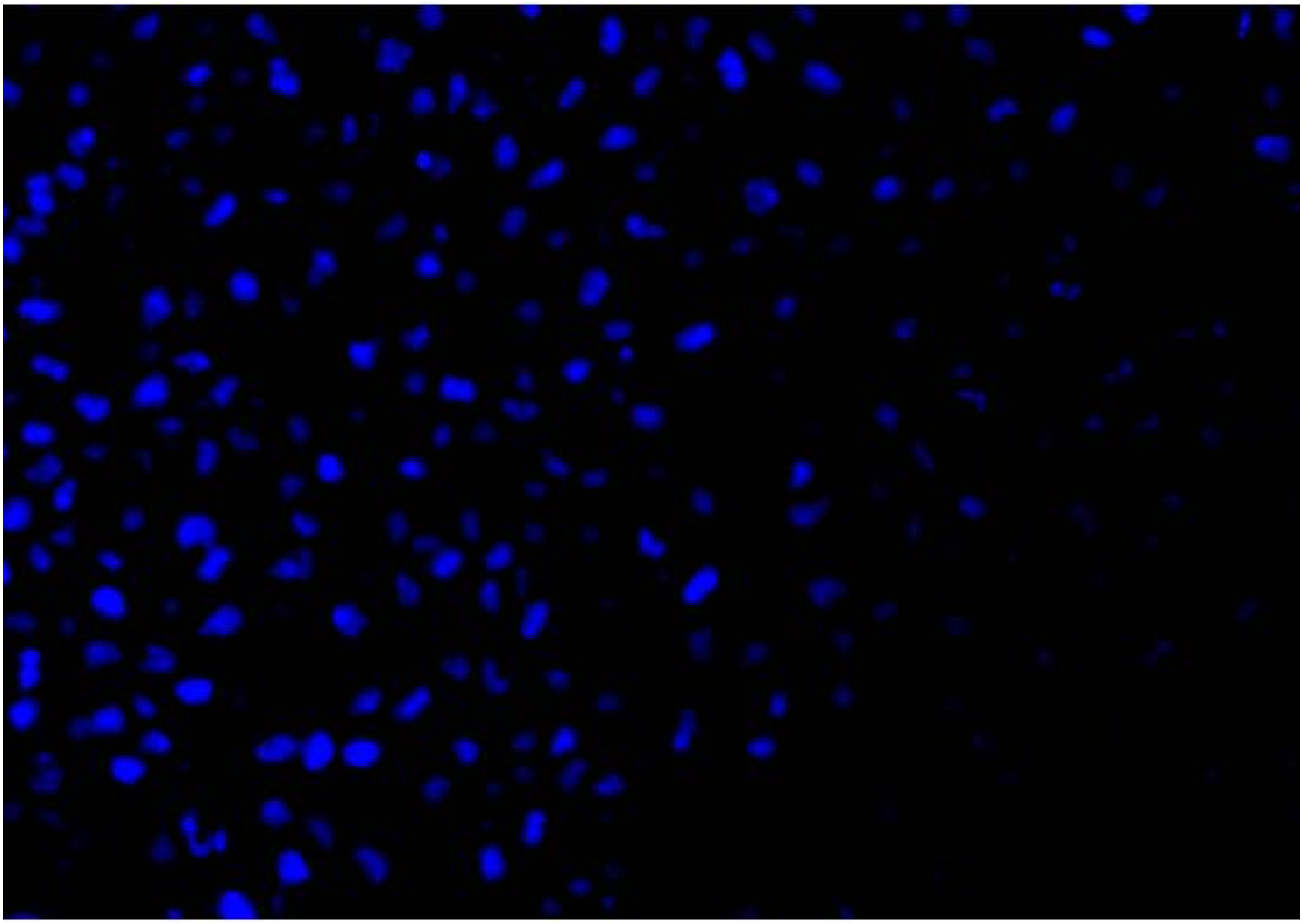

Raw image for Fig. 3f NANOG KD stage 10.5 animal cap stained for H3K27ac

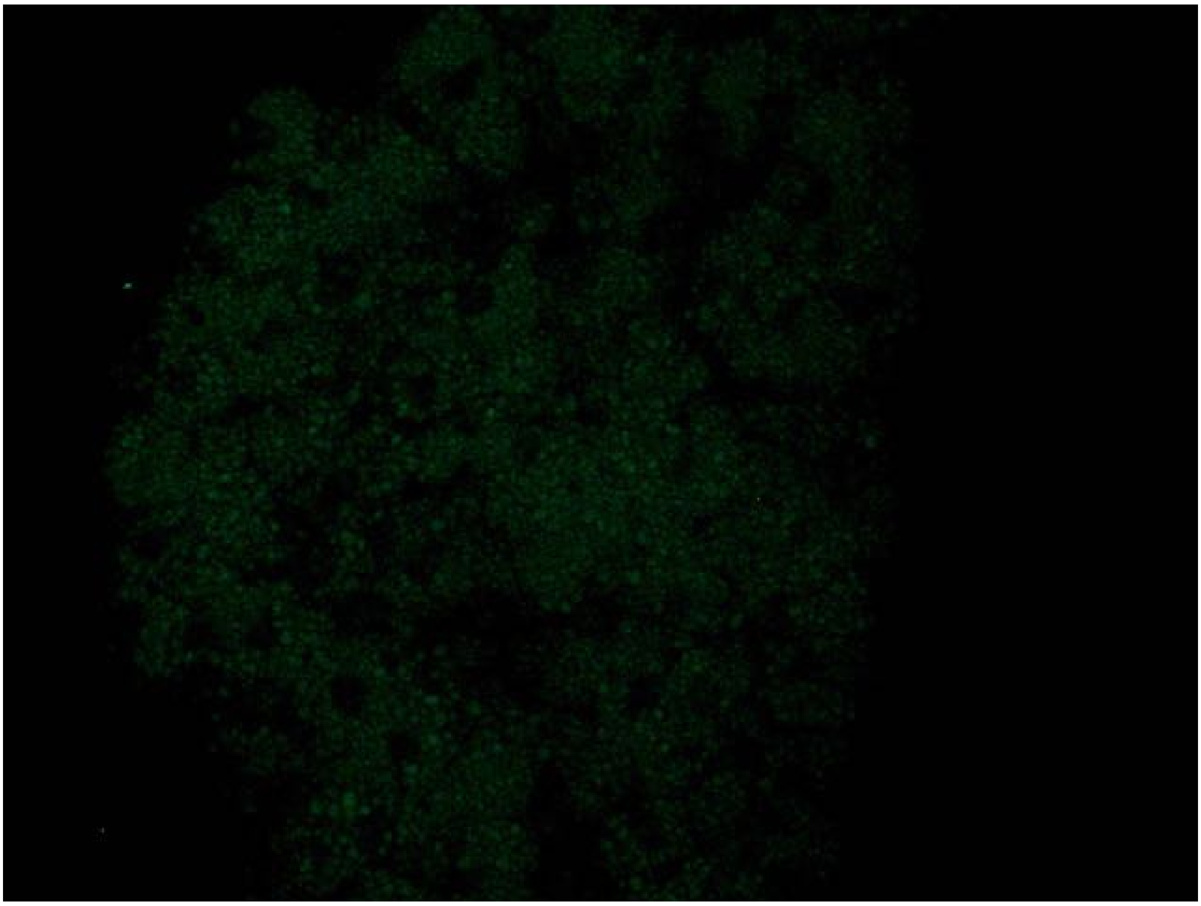

Raw image for Fig. 3f NANOG KD stage 10.5 animal cap stained for DAPI

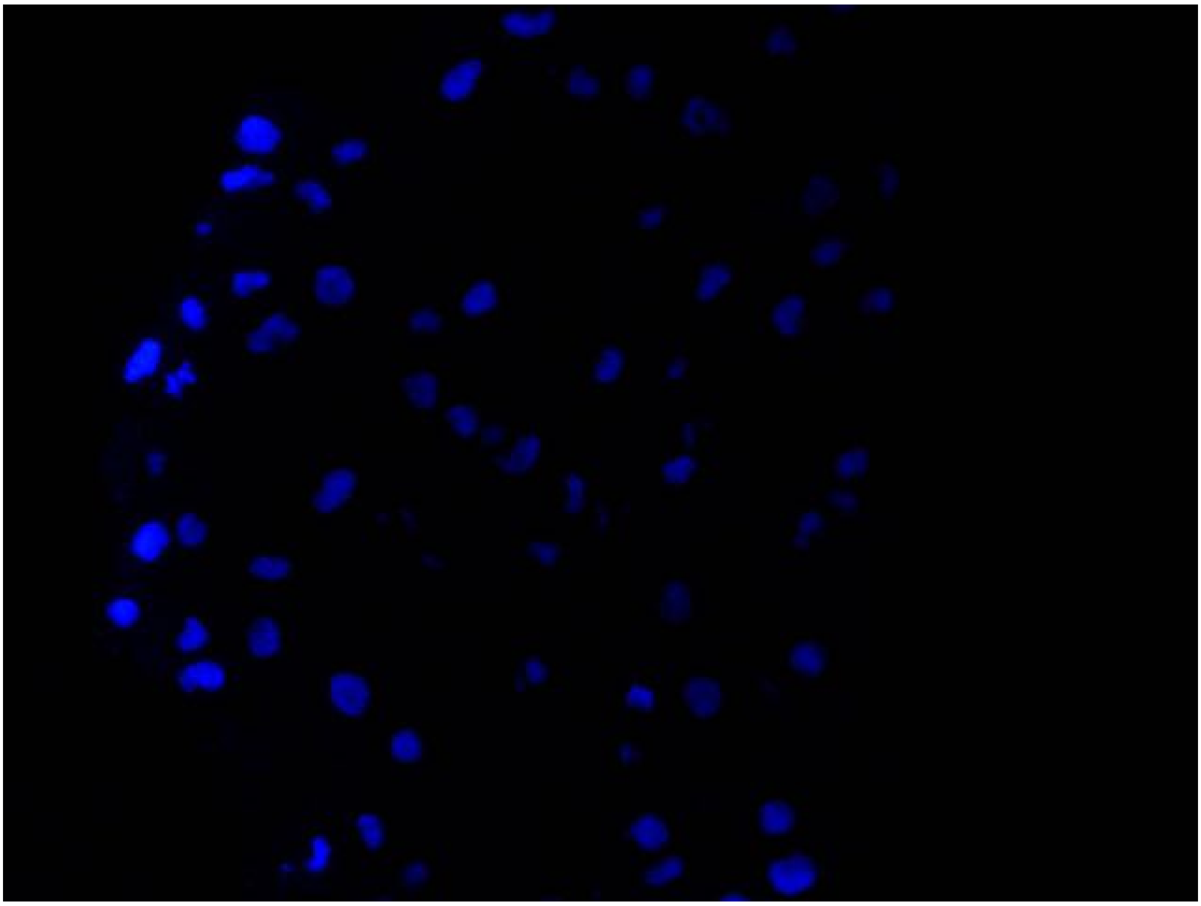

Raw image for Fig. 3f NANOG KD + HNANOG stage 10.5 animal cap stained for H3K27ac

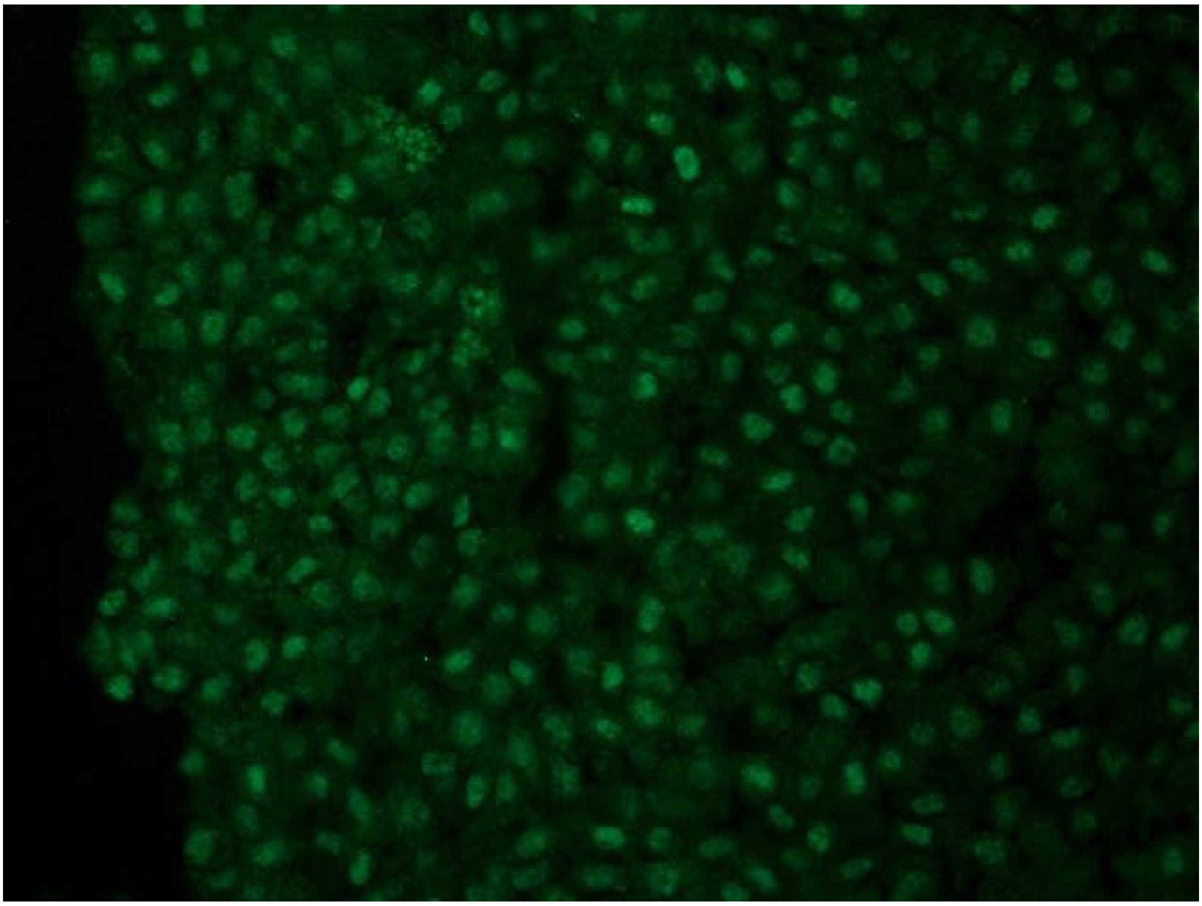

Raw image for Fig. 3f NANOG KD + HNANOG stage 10.5 animal cap stained for DAPI

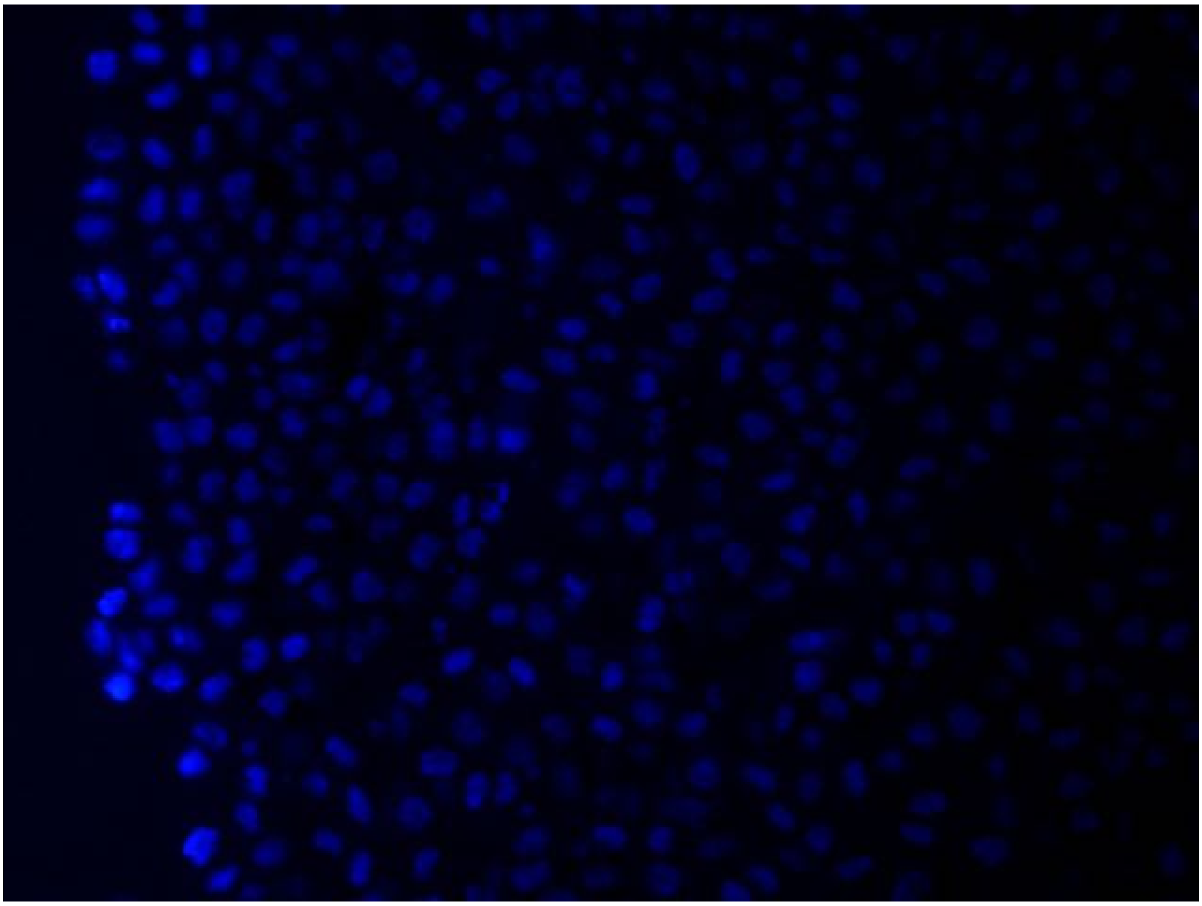

Raw image for Fig. 3f Uninjected stage 10.5 animal cap stained for H3K27me3

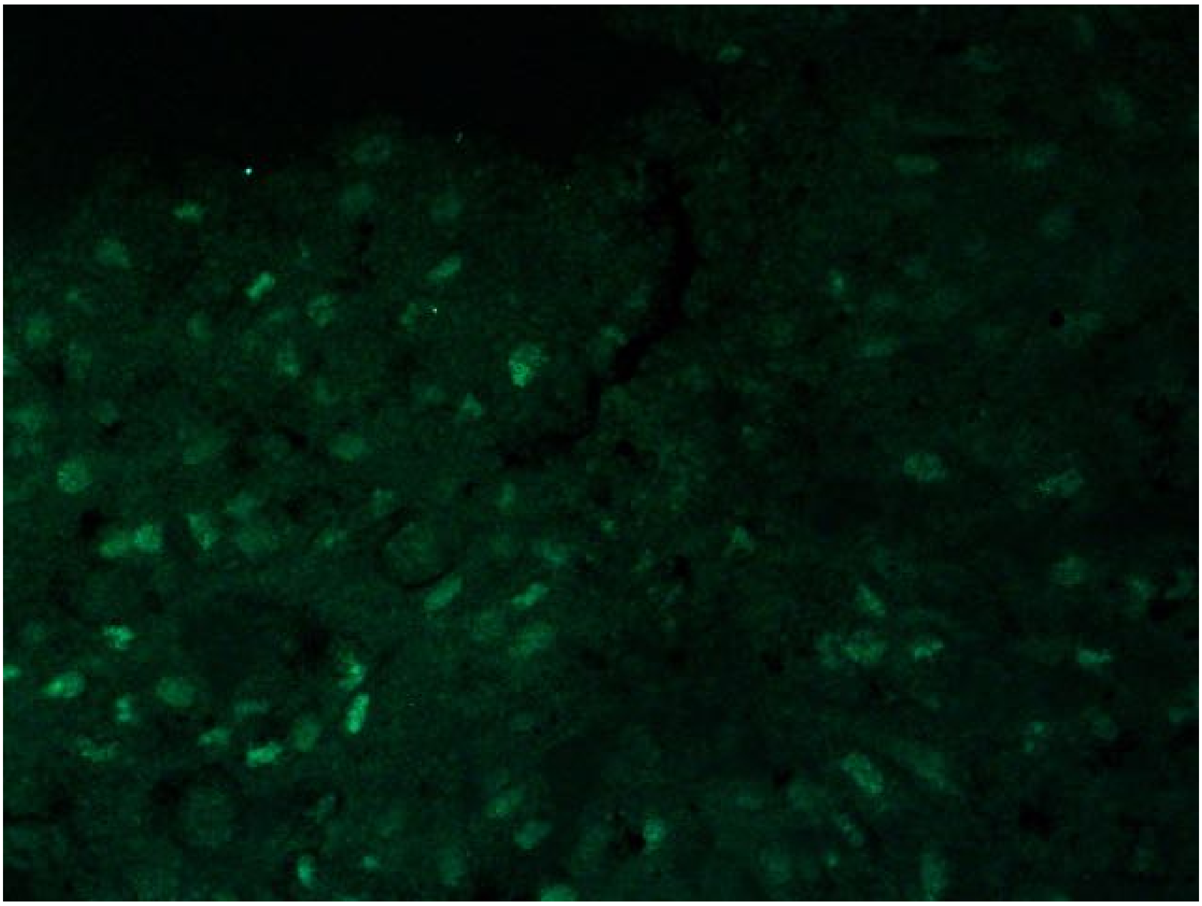

Raw image for Fig. 3f Uninjected stage 10.5 animal cap stained for DAPI

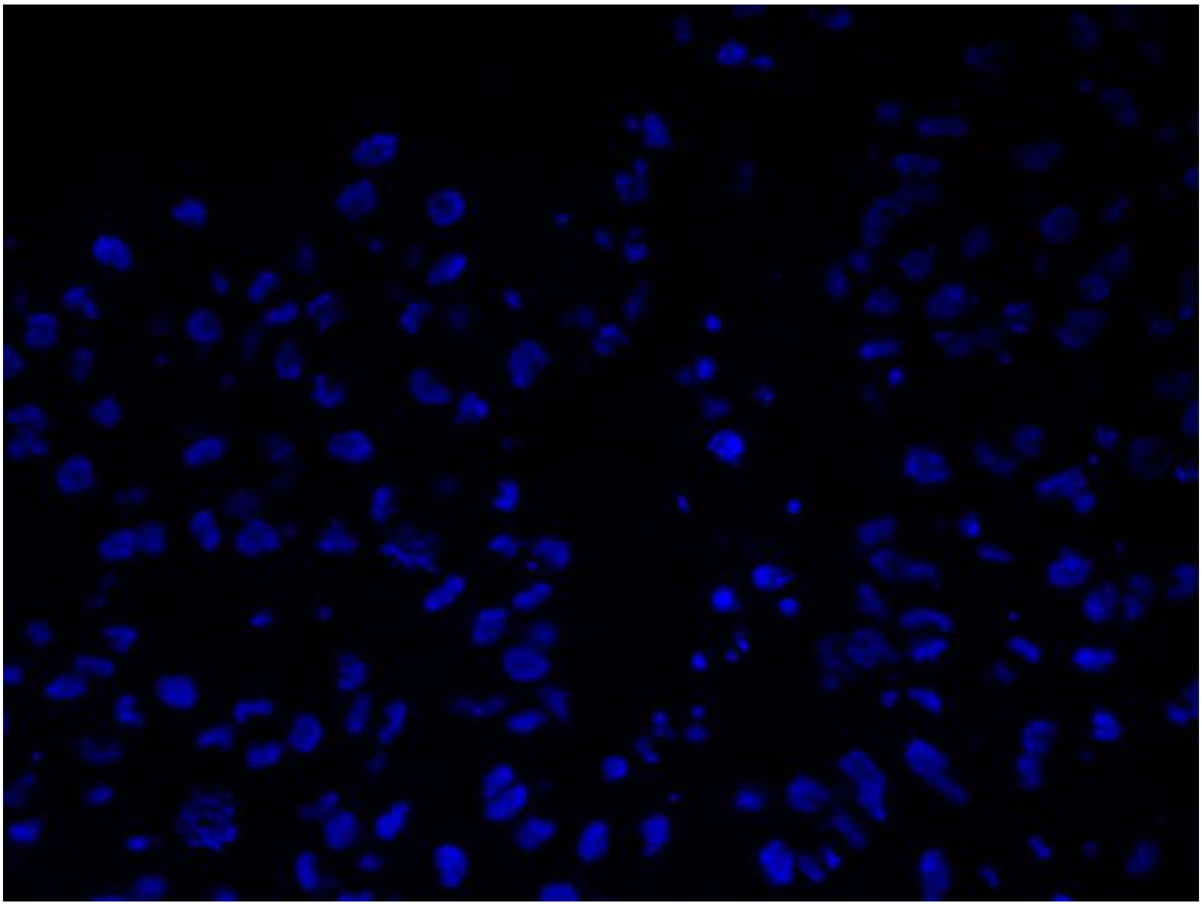

Raw image for Fig. 3f NANOG KD stage 10.5 animal cap stained for H3K27me3

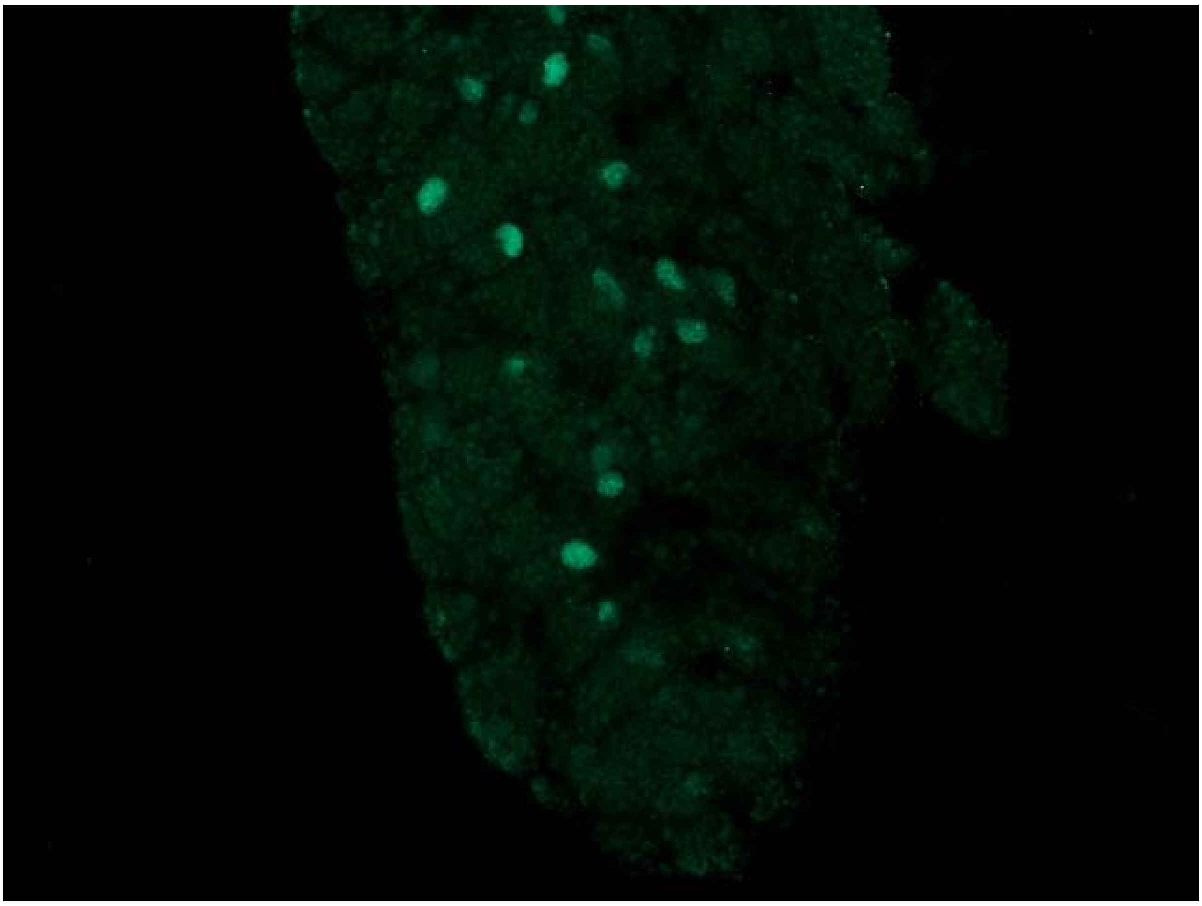

Raw image for Fig. 3f NANOG KD stage 10.5 animal cap stained for DAPI

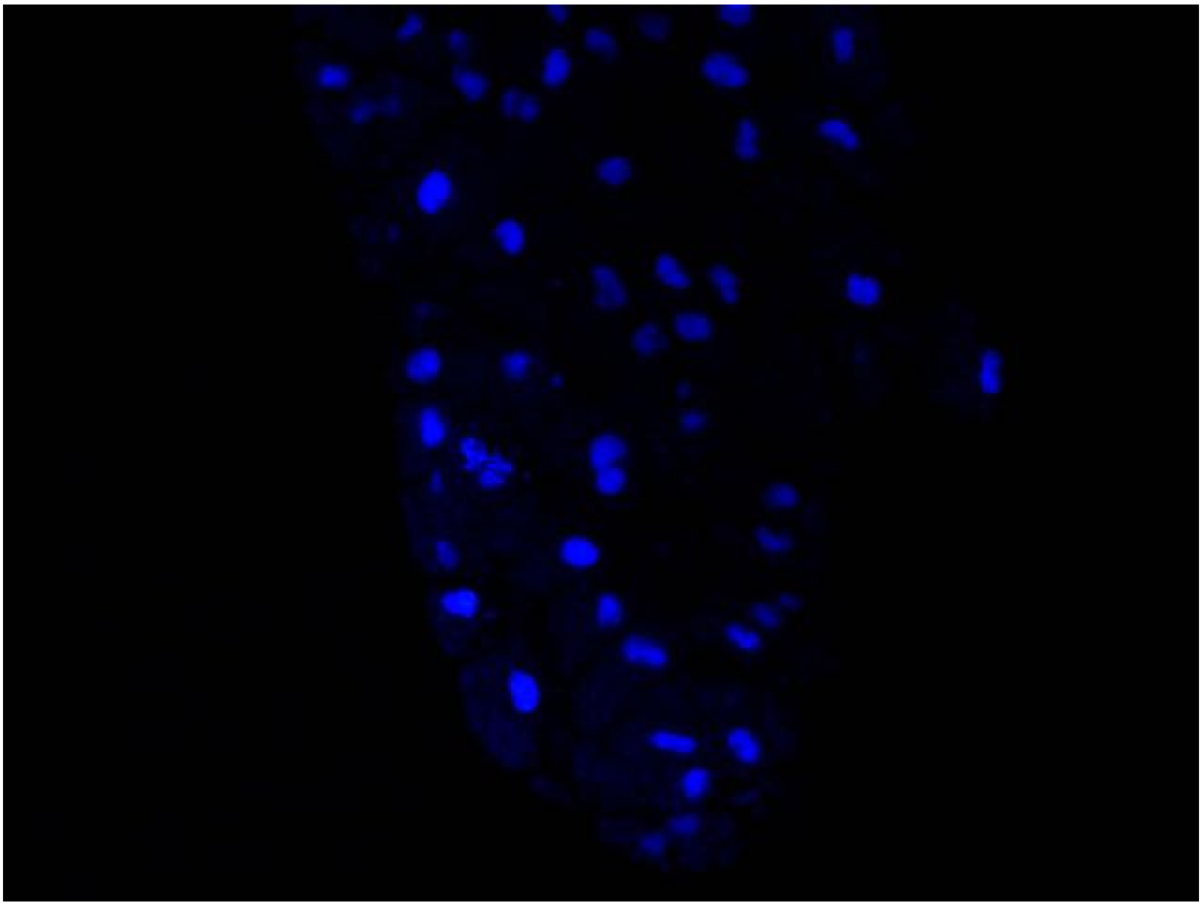

Raw image for Fig. 3f NANOG KD + HNANOG stage 10.5 animal cap stained for H3K27me3

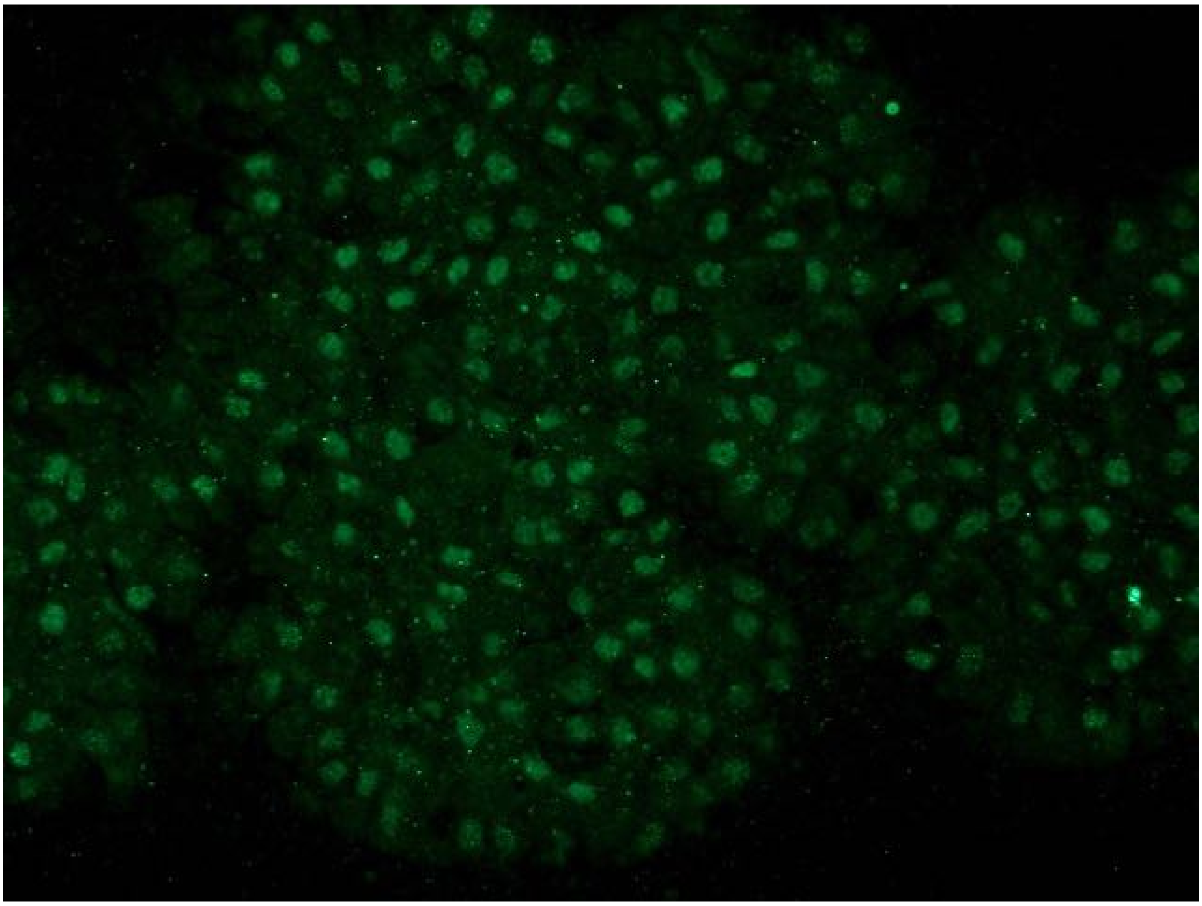

Raw image for Fig. 3f NANOG KD + HNANOG stage 10.5 animal cap stained for DAPI

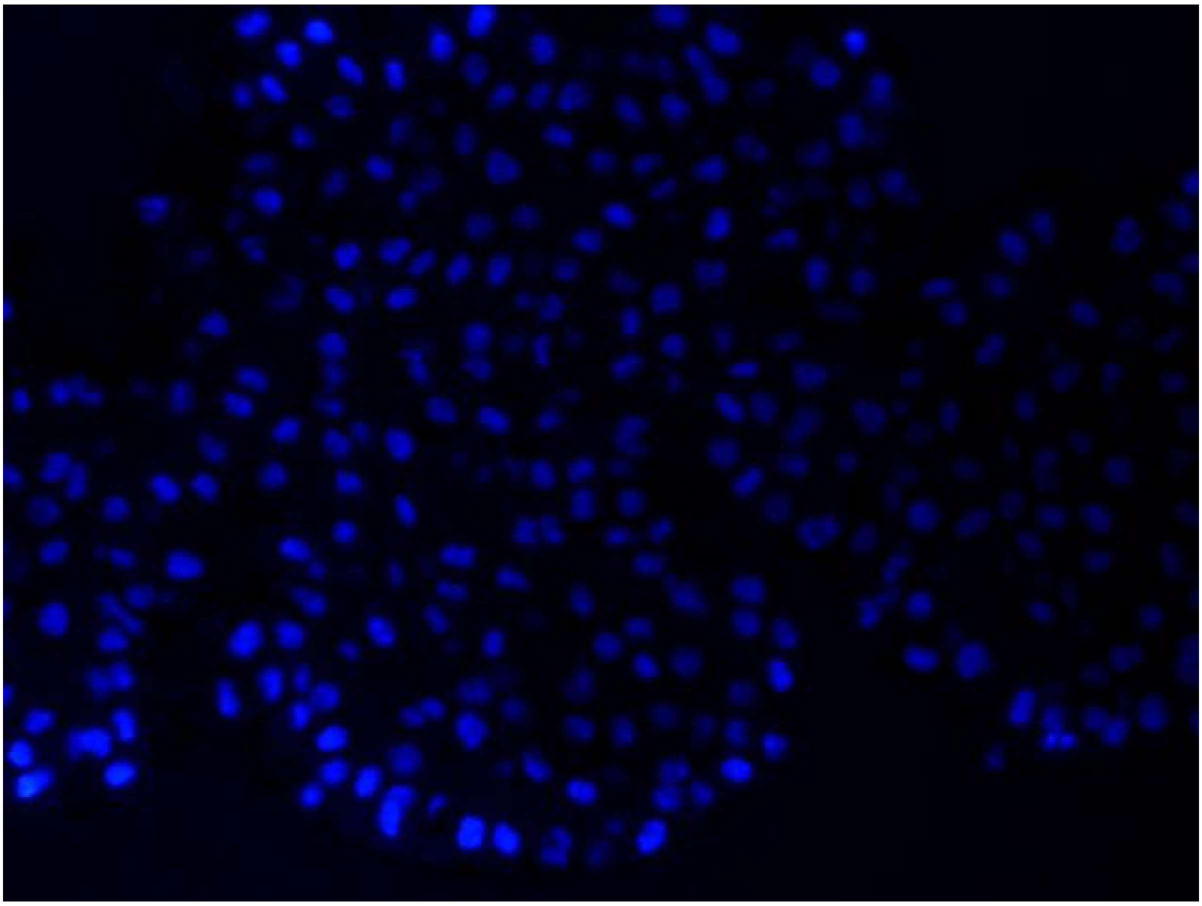

Raw image for Fig. 4a Uninjected stage 40 brightfeild image

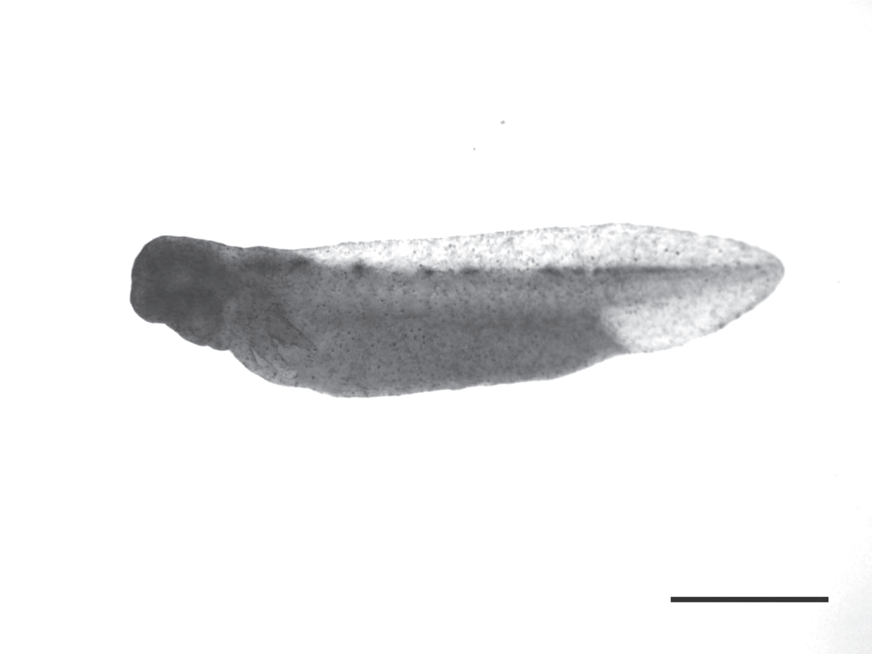

Raw image for Fig. 4a SB431542 treated stage 40 timepoint equivalent brightfeild image

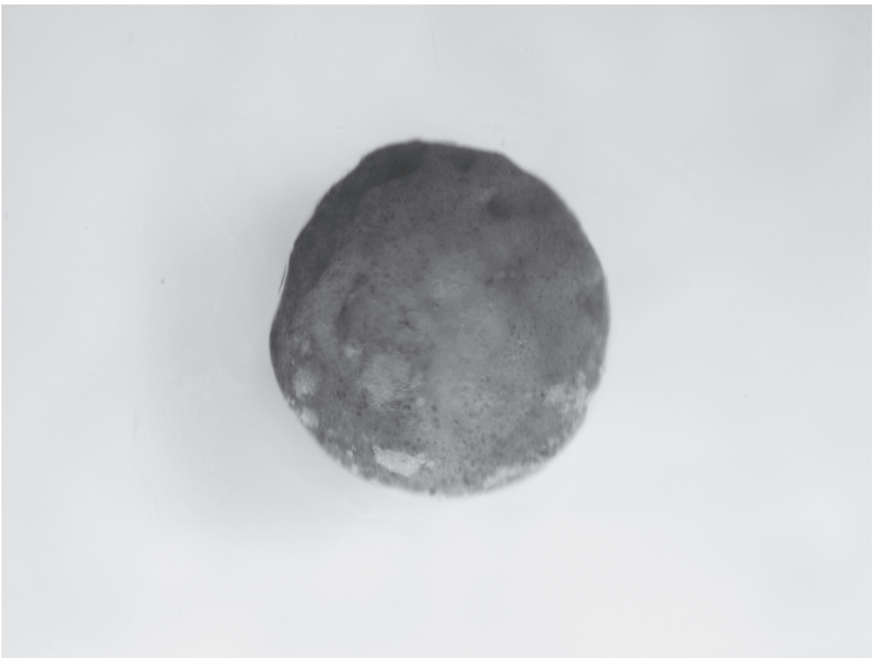

Raw image for Fig. 4a SB431542 treated stage 40 timepoint equivalent HREM 3D reconstruction

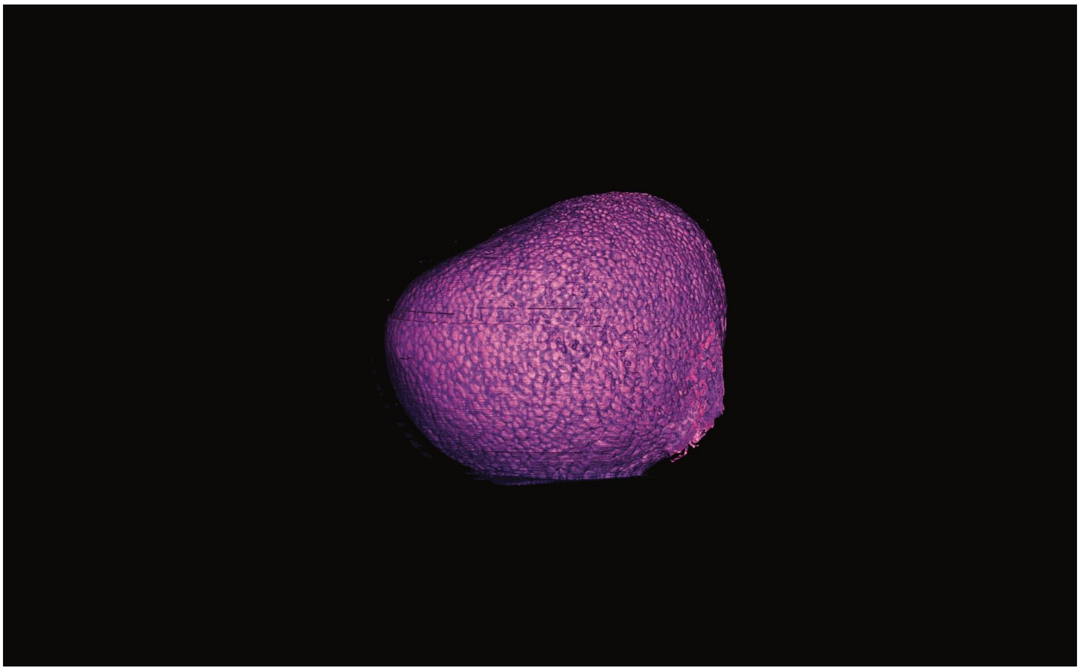

Raw image for Fig. 4a SB431542 treated stage 40 timepoint equivalent HREM 3D reconstruction sagital section

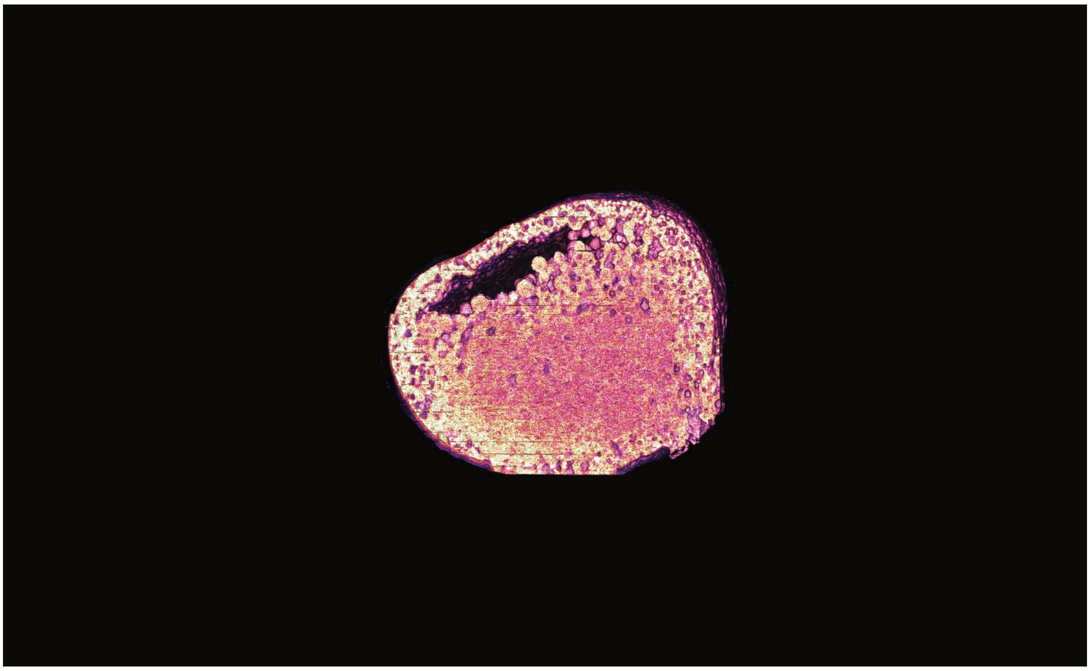

Raw image for Fig. 5c Untreated stage 10.5 animal cap stained for H3K4me3

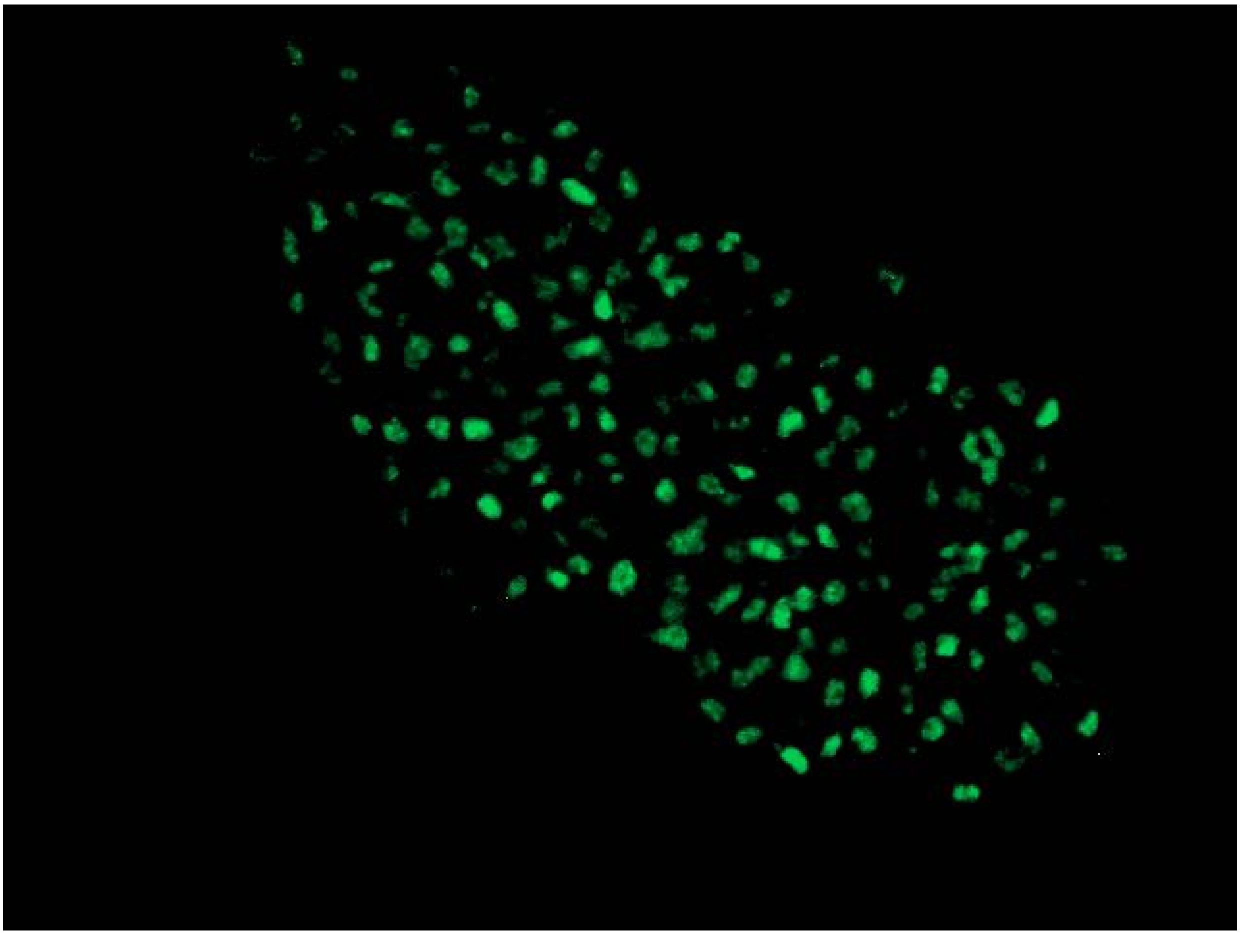

Raw image for Fig. 5c Untreated stage 10.5 animal cap stained for DAPI

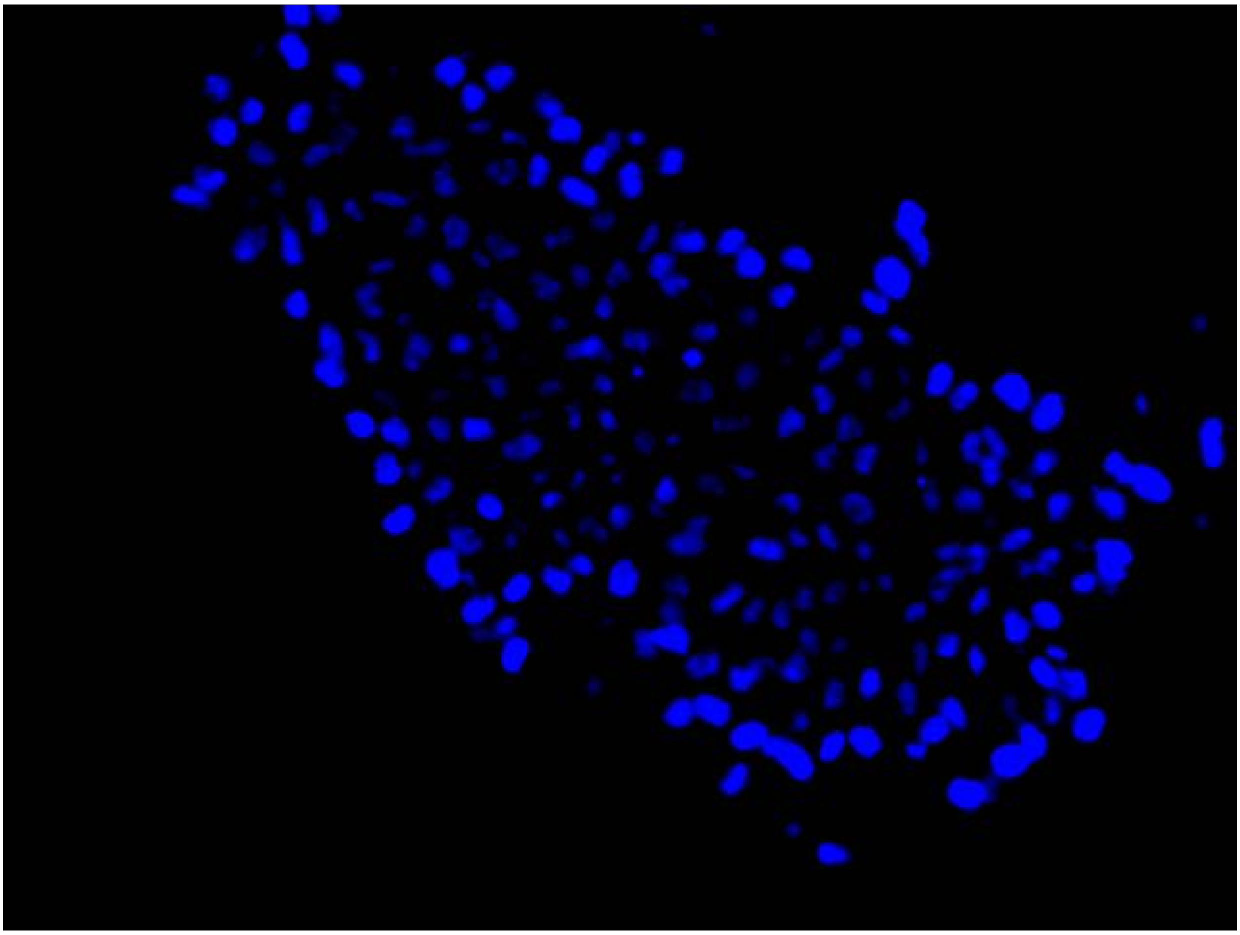

Raw image for Fig. 5c SB431542 treated stage 10.5 animal cap stained for H3K4me3

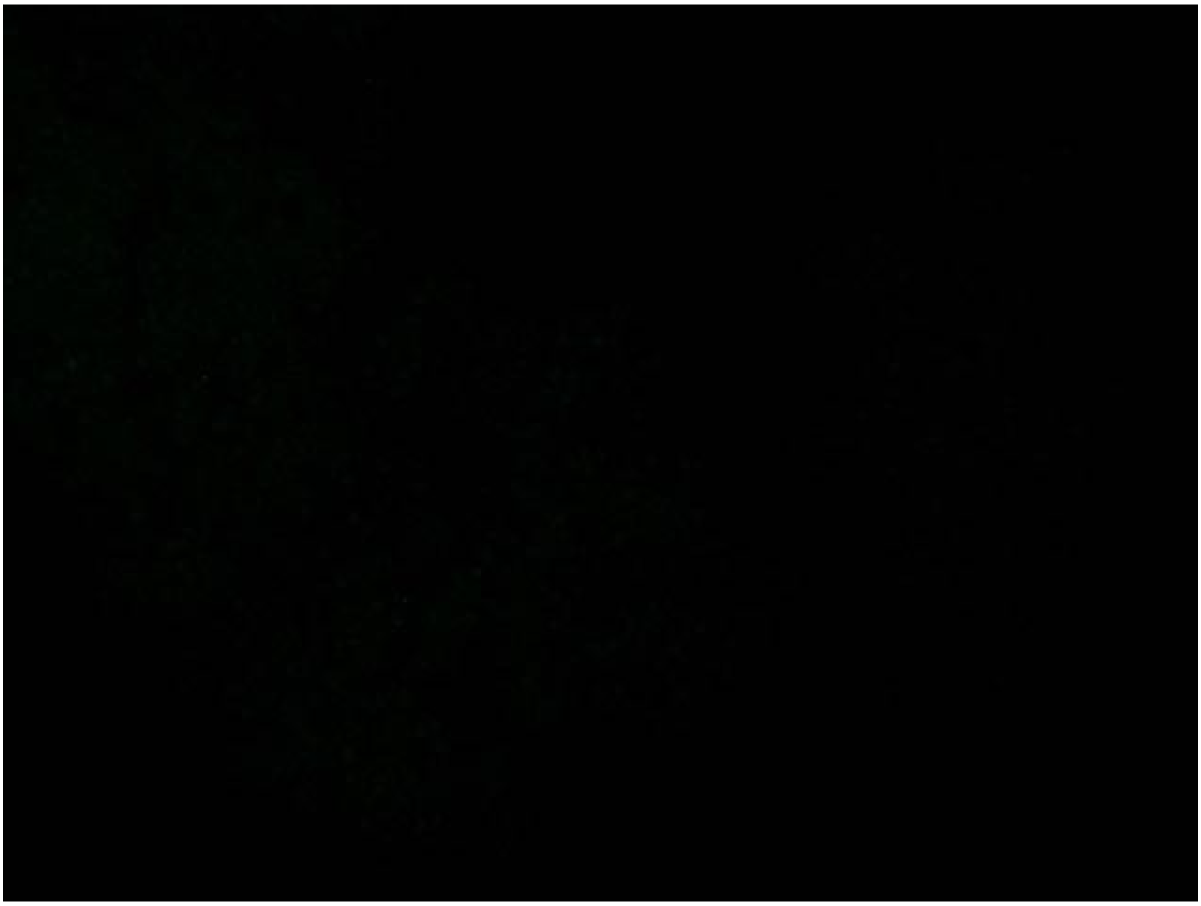

Raw image for Fig. 5c SB431542 treated stage 10.5 animal cap stained for DAPI

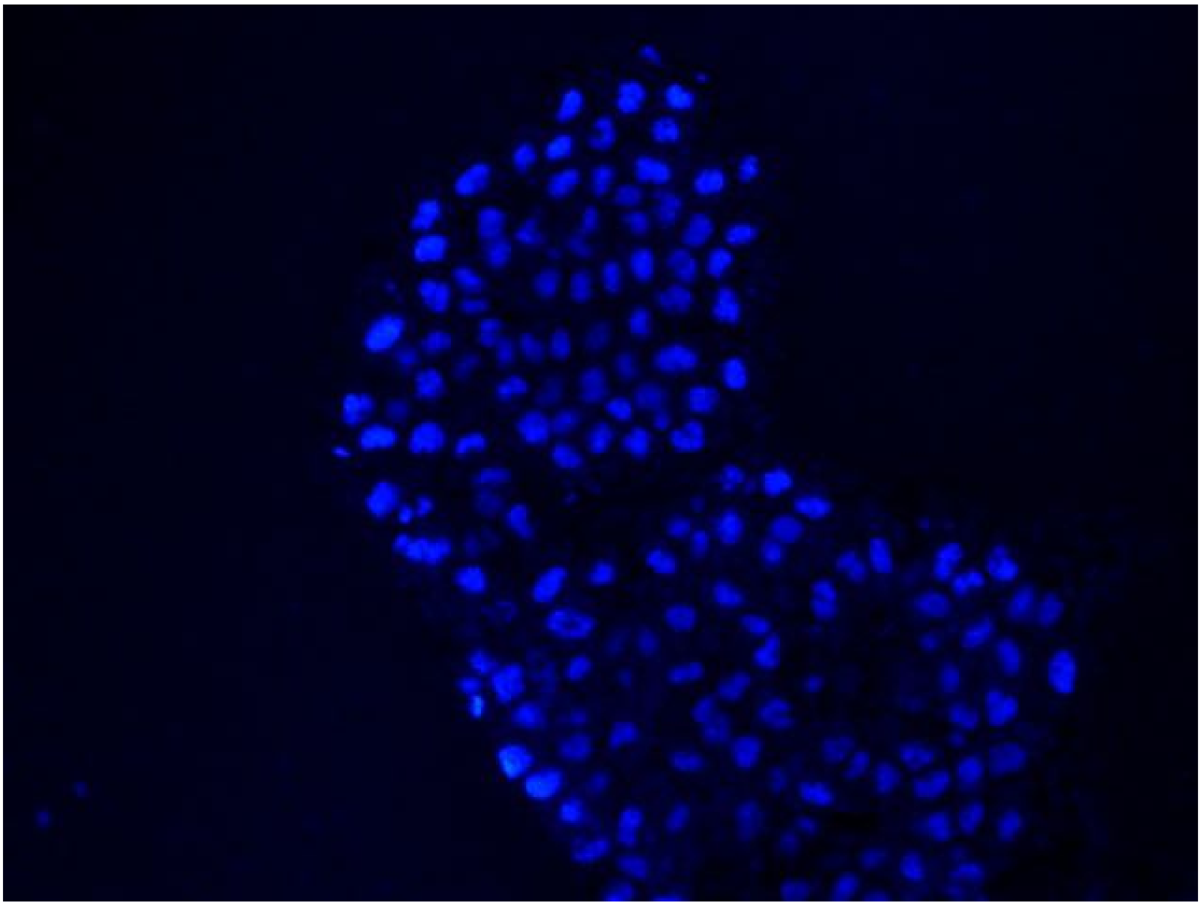

Raw image for Fig. 5c SB431542 + pSMAD2 treated stage 10.5 animal cap stained for H3K4me3

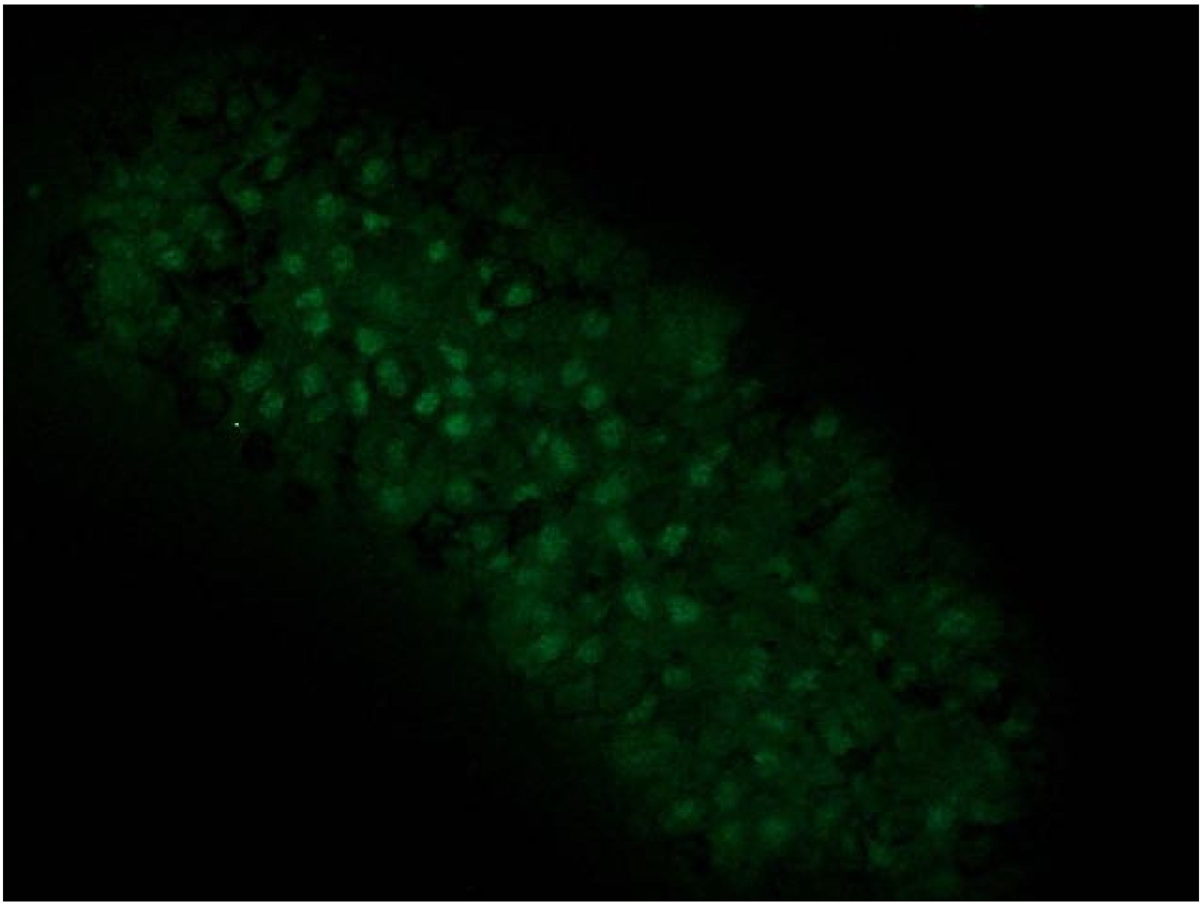

Raw image for Fig. 5c SB431542 treated stage 10.5 animal cap stained for DAPI

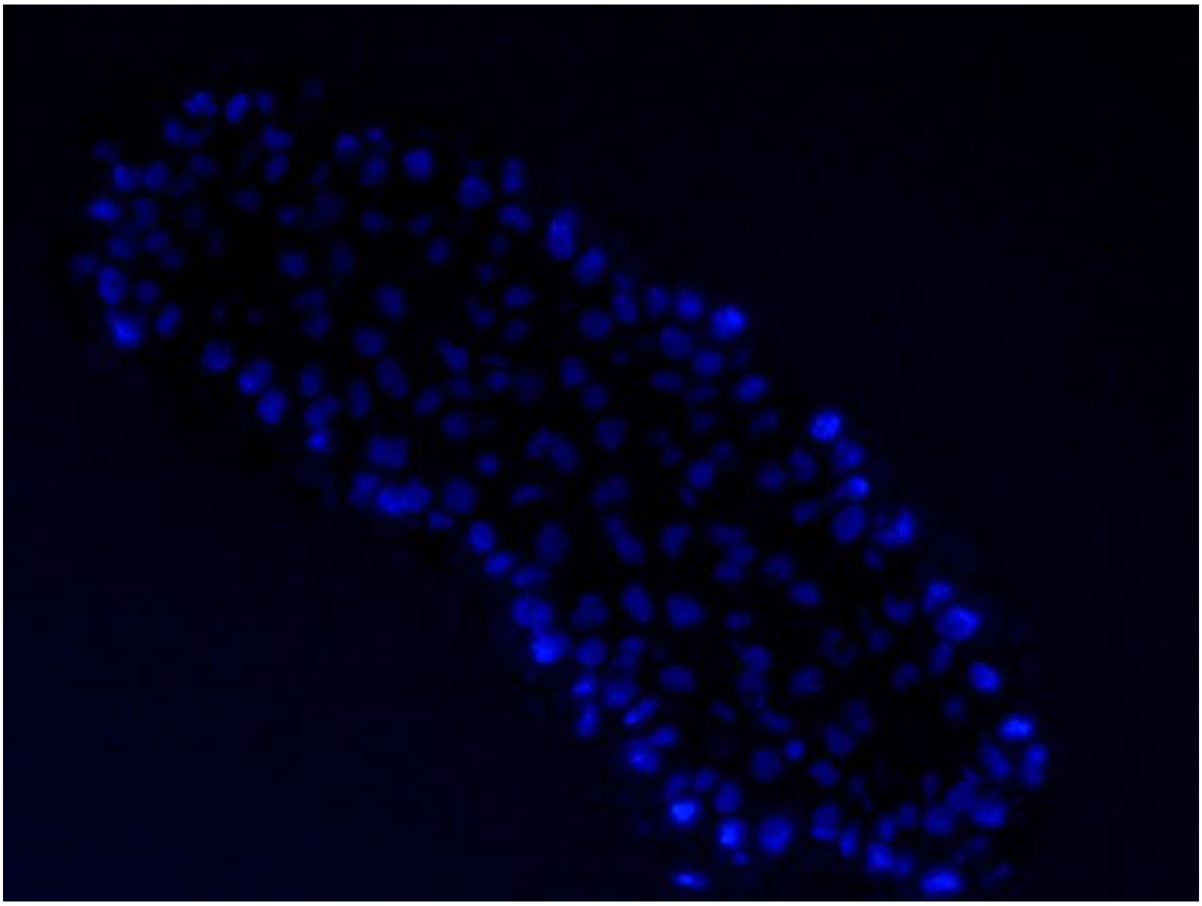

Raw image for Fig. 3f Uninjected stage 10.5 animal cap stained for H3K27ac

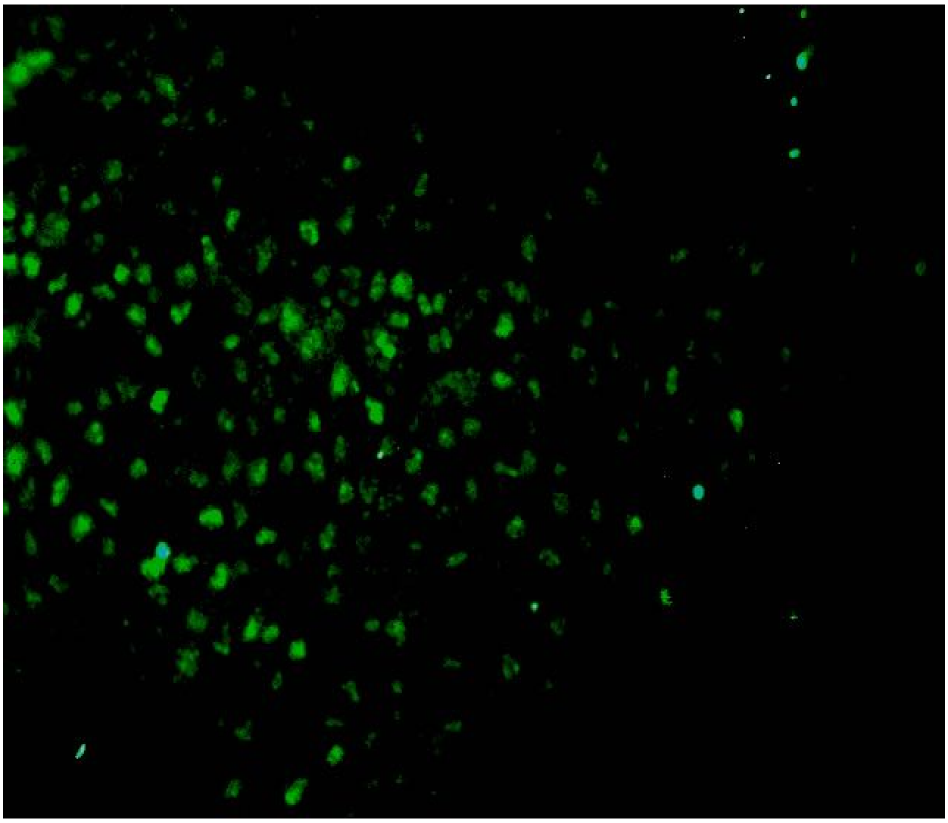

Raw image for Fig. 3f Uninjected stage 10.5 animal cap stained for DAPI

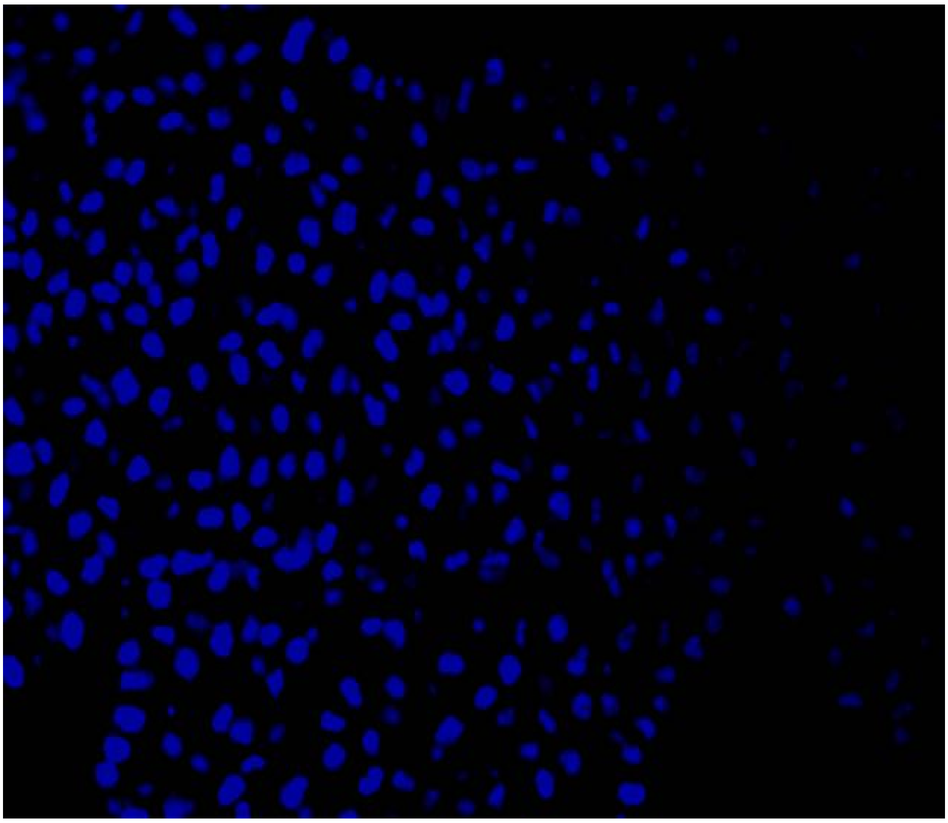

Raw image for Fig. 5c SB431542 treated stage 10.5 animal cap stained for H3K27ac

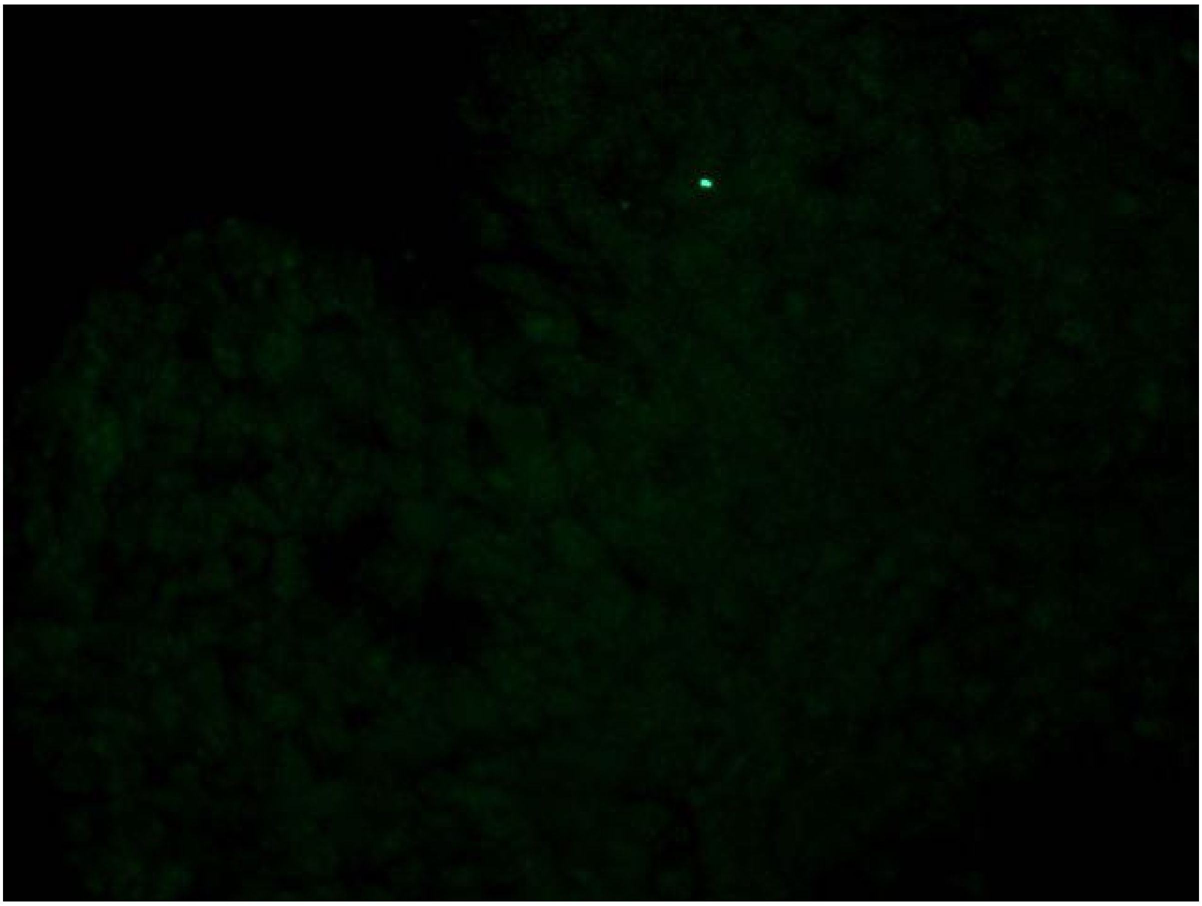

Raw image for Fig. 5c SB431542 treated stage 10.5 animal cap stained for DAPI

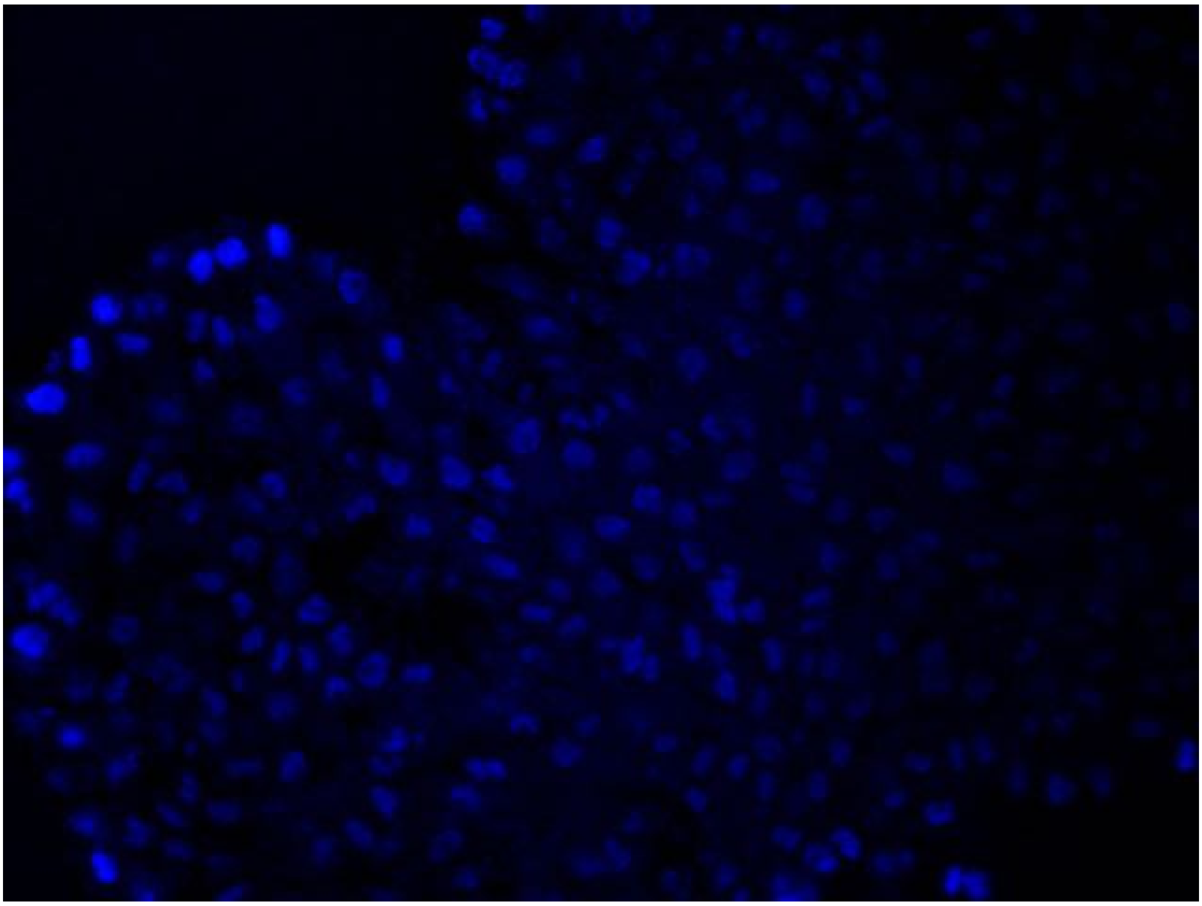

Raw image for Fig. 5c SB431542 + pSMAD2 treated stage 10.5 animal cap stained for H3K27ac

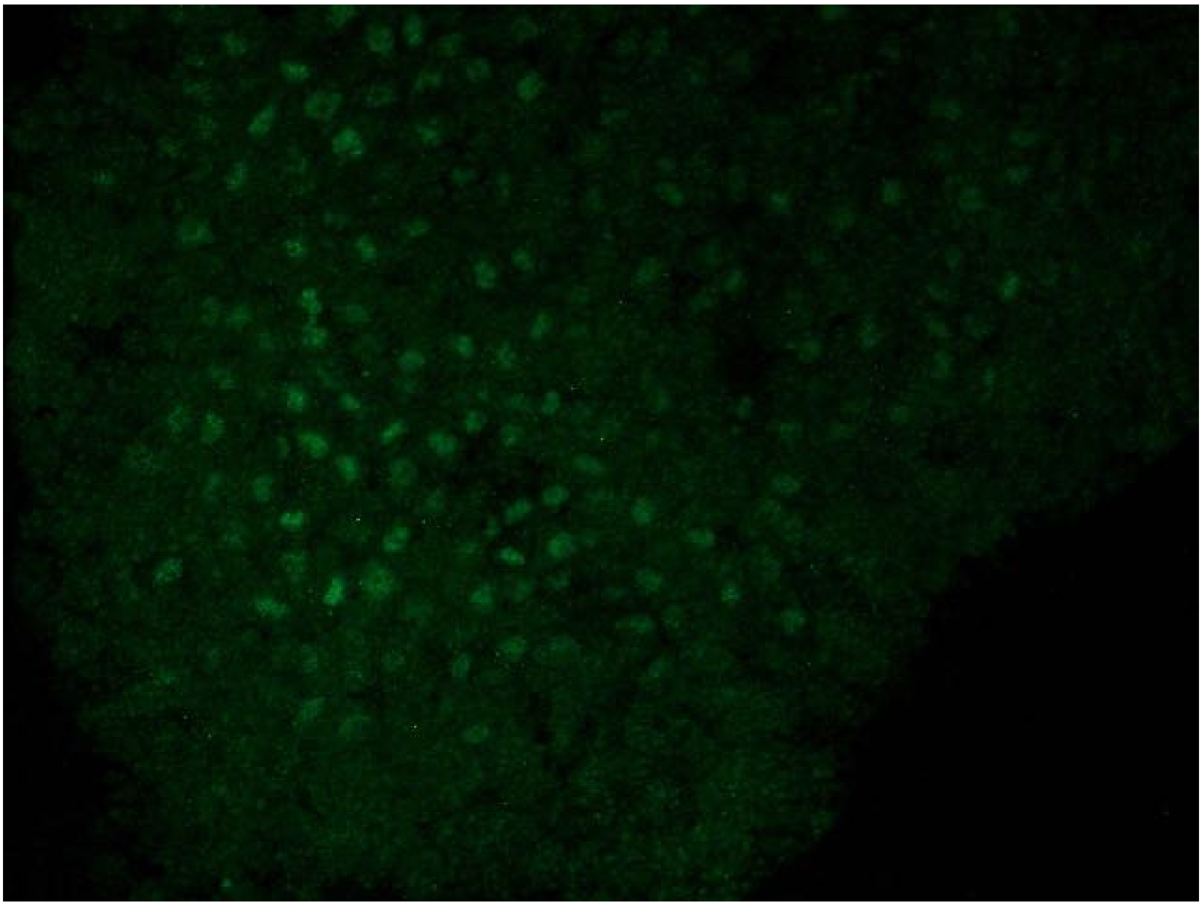

Raw image for Fig. 5c SB431542 + pSMAD2 treated stage 10.5 animal cap stained for DAPI

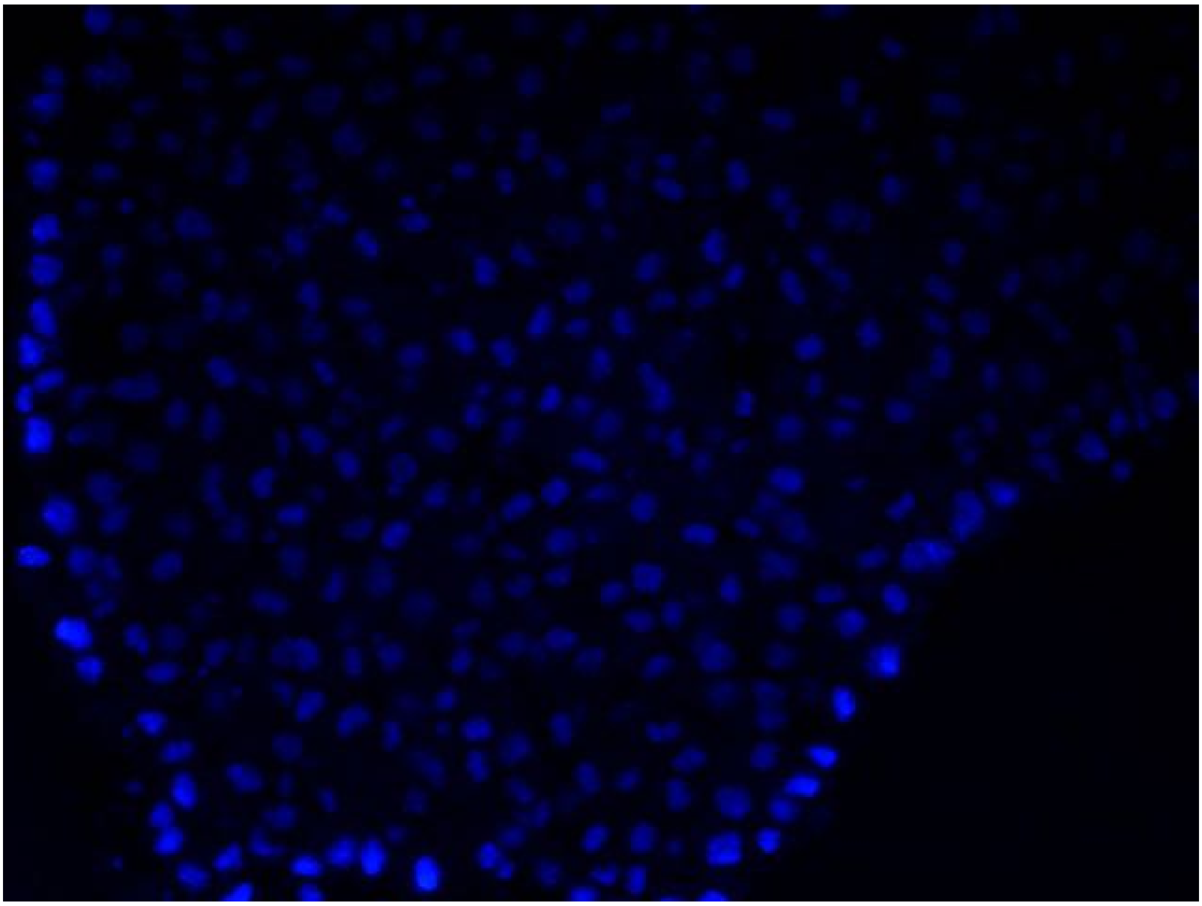

Raw image for Fig. 5c and Extended Data Fig.11e Untreated stage 10.5 animal cap stained for H3K27me3

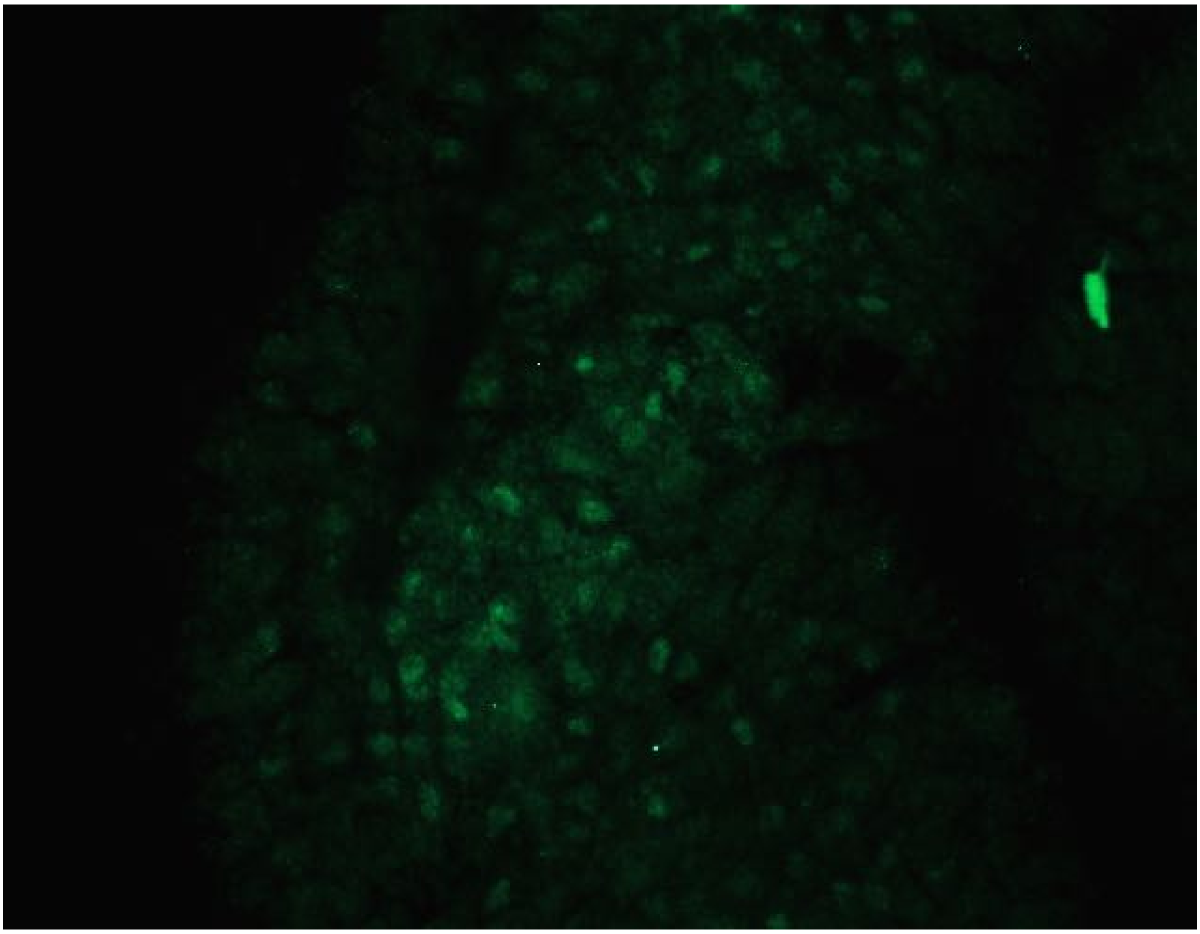

Raw image for Fig. 5c and Extended Data Fig.11e Untreated stage 10.5 animal cap stained forDAPI

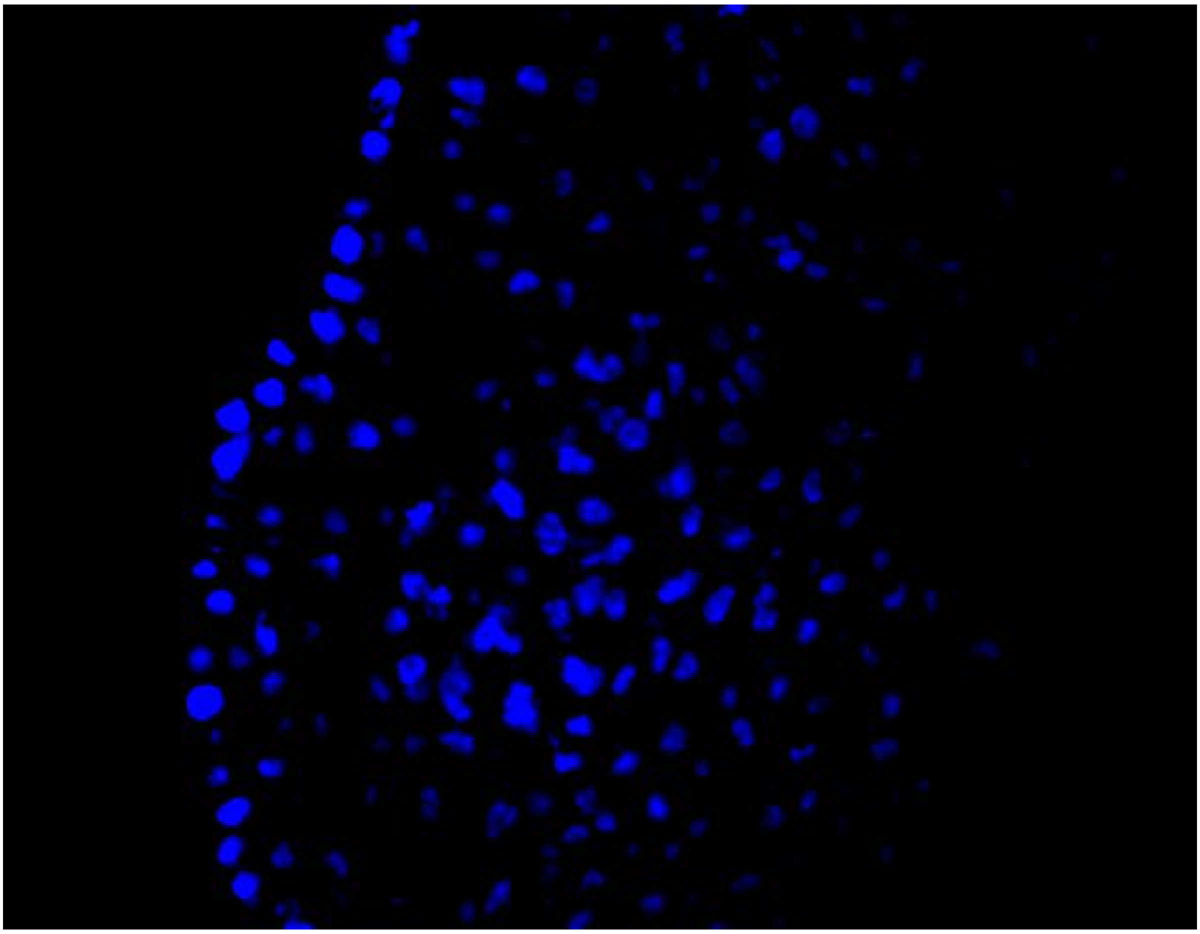

Raw image for Fig. 5c SB431542 treated stage 10.5 animal cap stained for H3K27me3

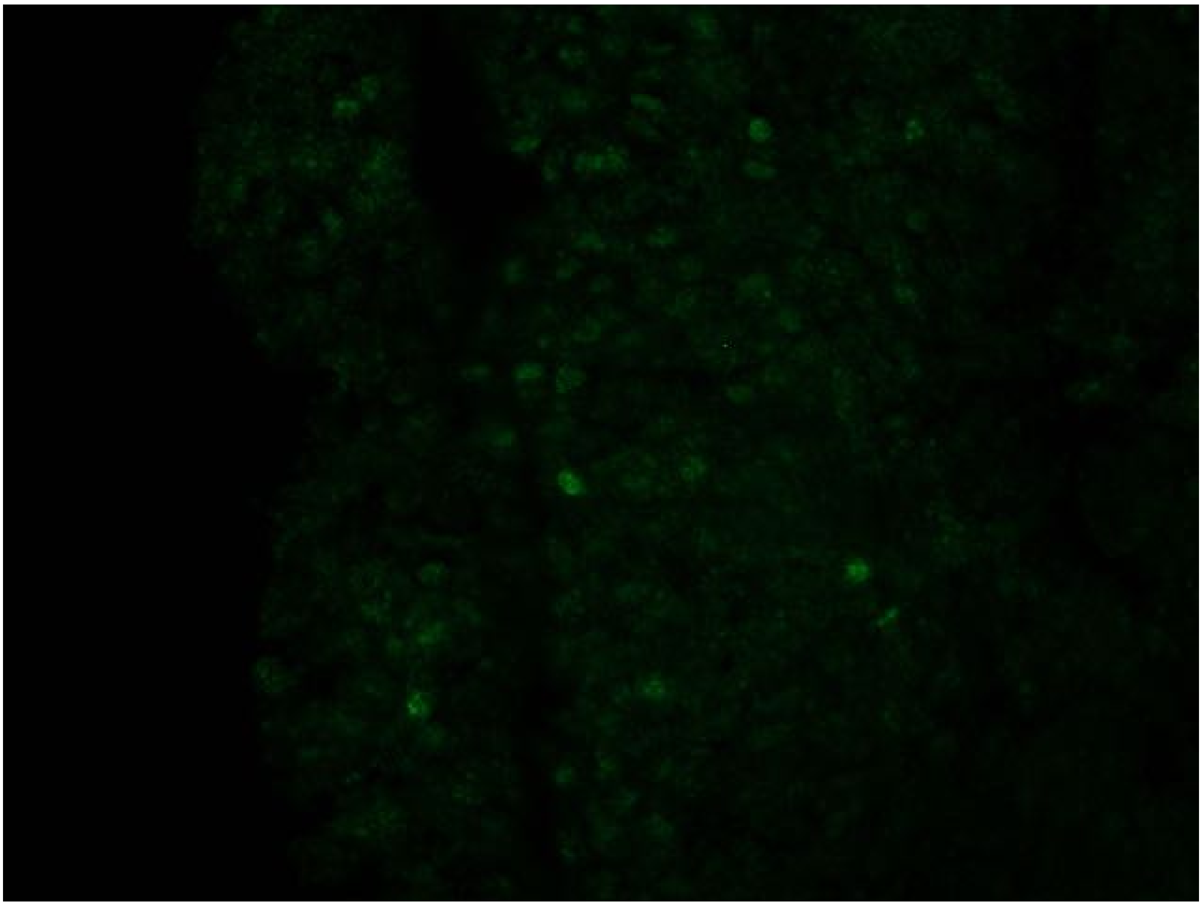

Raw image for Fig. 5c SB431542 treated stage 10.5 animal cap stained for DAPI

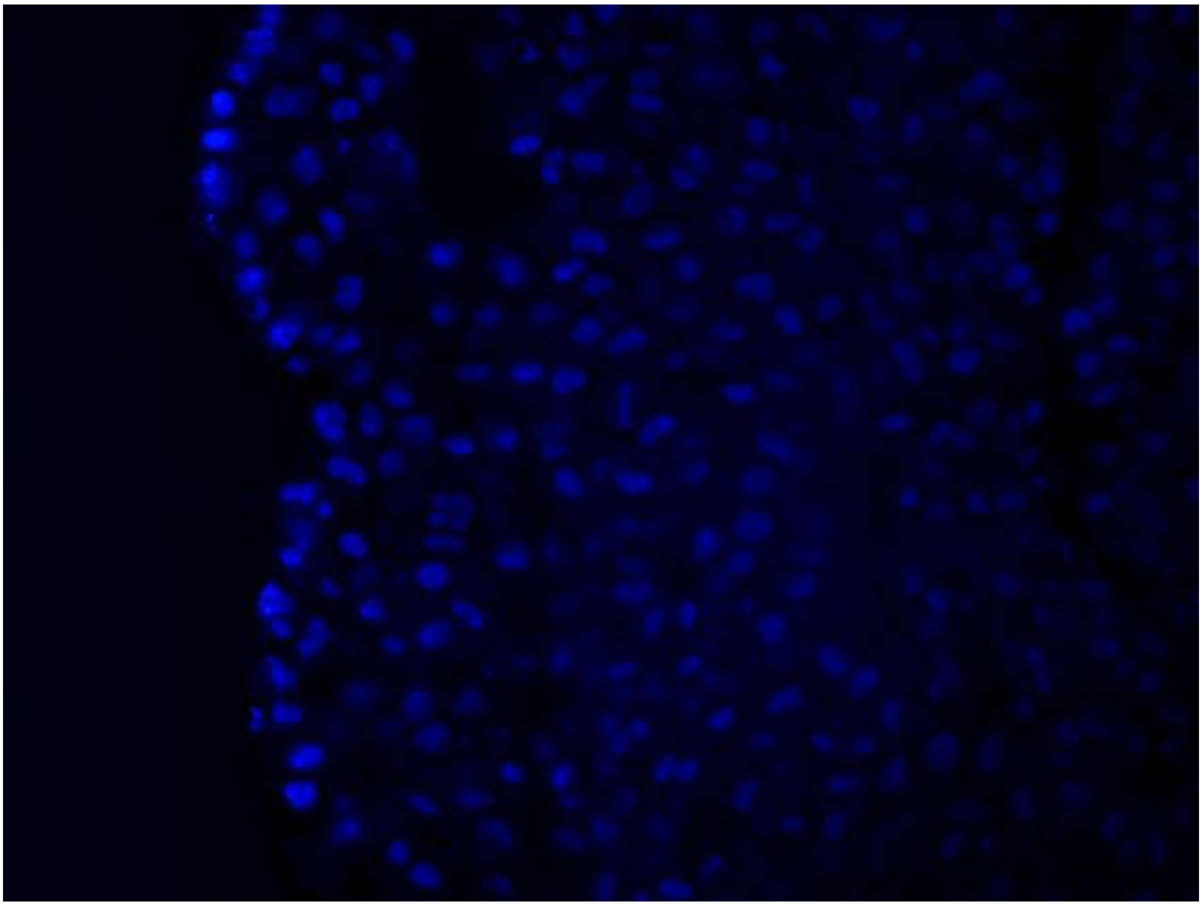

Raw image for Fig. 5c SB431542 + pSMAD2 treated stage 10.5 animal cap stained for H3K27me3

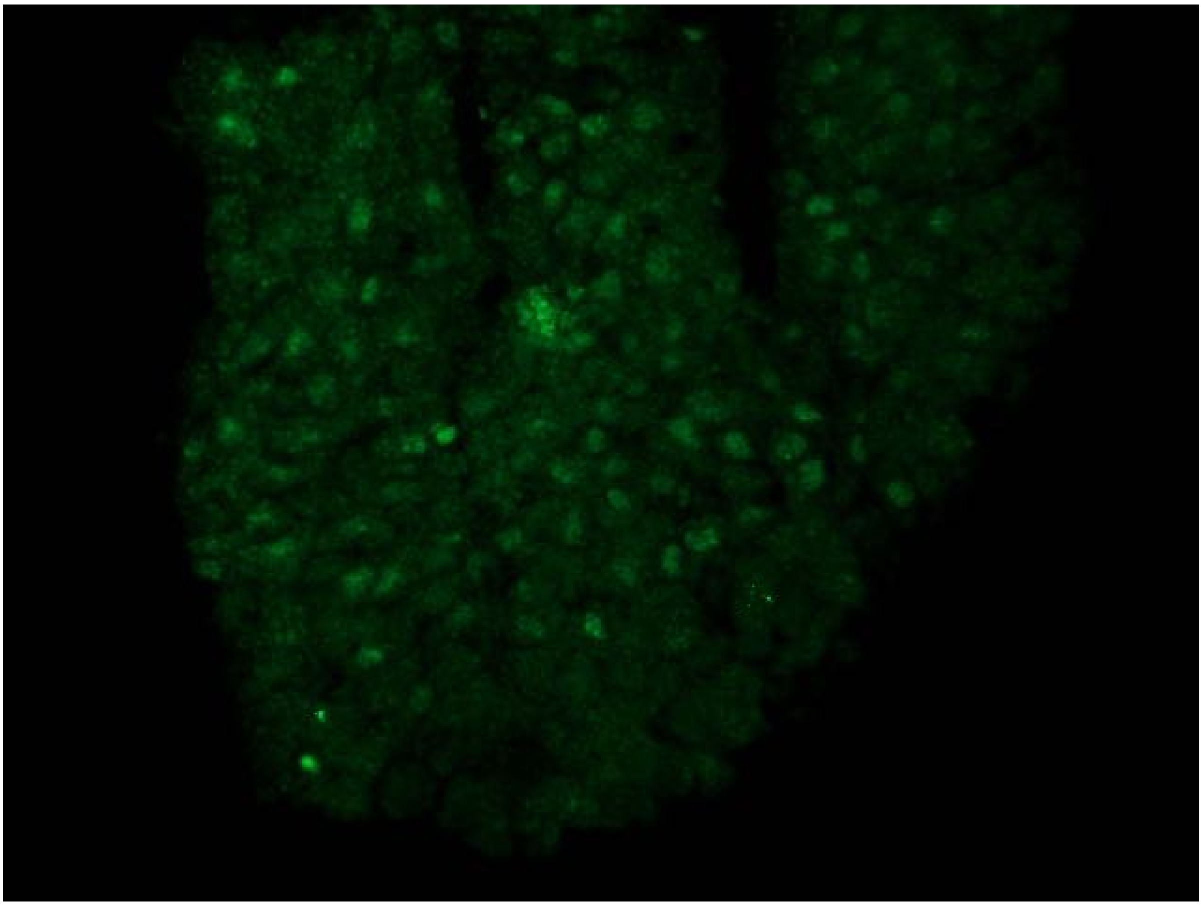

Raw image for Fig. 5c SB431542 + pSMAD2 treated stage 10.5 animal cap stained for DAPI

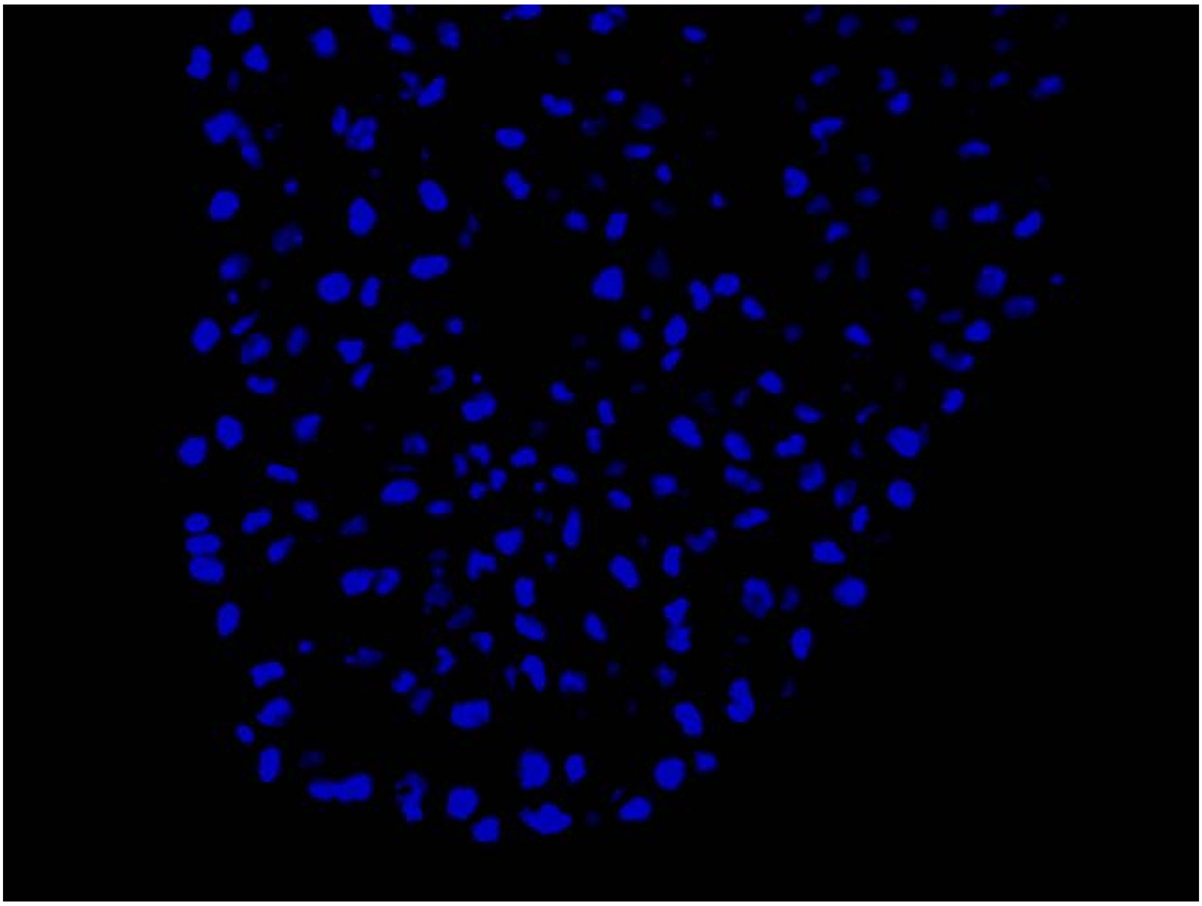

Raw image for Extended Data Fig. 1a western blot stained with Anti-HA
Lane 1 is loaded with Rainbow ladder
Lane 4 is loaded with a lysate made from Xenopus oocytes injected with NANOG-HA mRNA
Lane 5 is loaded with a lysate made from uninjected Xenopus oocytes
Lane 6 is loaded with a commercially available multiple tagged lysate with an HA tag
Cropped image shown in inset

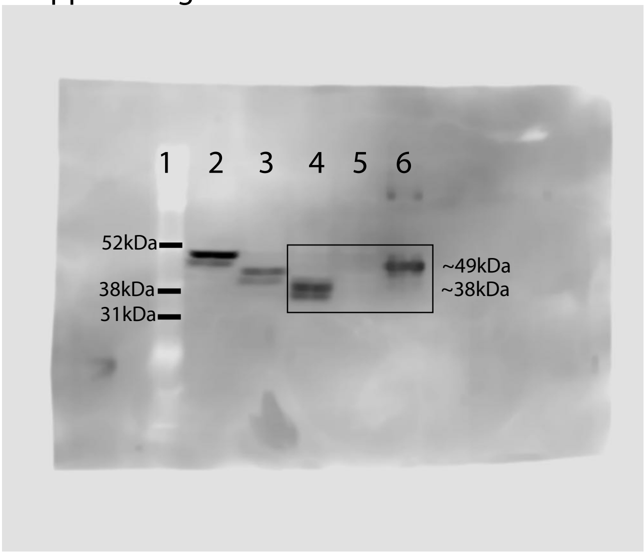

Raw image for Extended Data Fig. 1a western blot stained with Anti-NANOG
Lane 1 is loaded with Rainbow ladder
Lane 4 is loaded with a lysate made from Xenopus oocytes injected with NANOG-HA mRNA
Lane 5 is loaded with a lysate made from uninjected Xenopus oocytes
Lane 6 is loaded with a commercially available multiple tagged lysate with an HA tag
Cropped image shown in inset

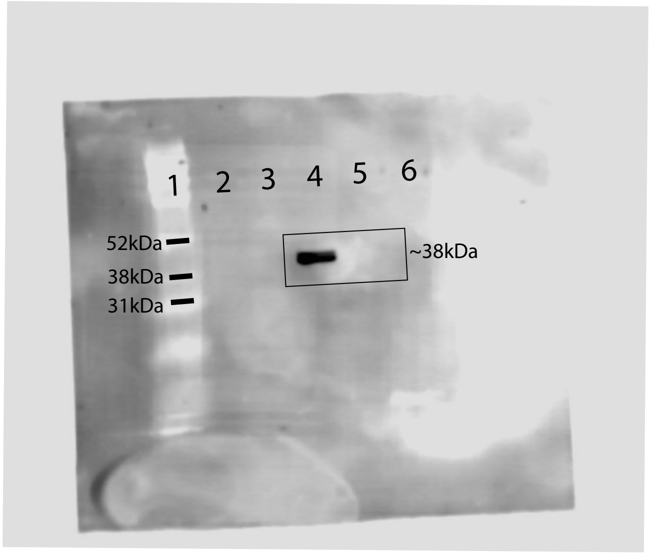

Raw image for Extended Data Fig. 1b western blot stained with Anti-NANOG
Lane 2 loaded with Rainbow ladder, Lane 3 loaded with stage 10.5 uninjected embryo lysate
Lane 4 loaded with stage 10.5 equivalent NANOG KD embryo lysate. Cropped image shown in inset.

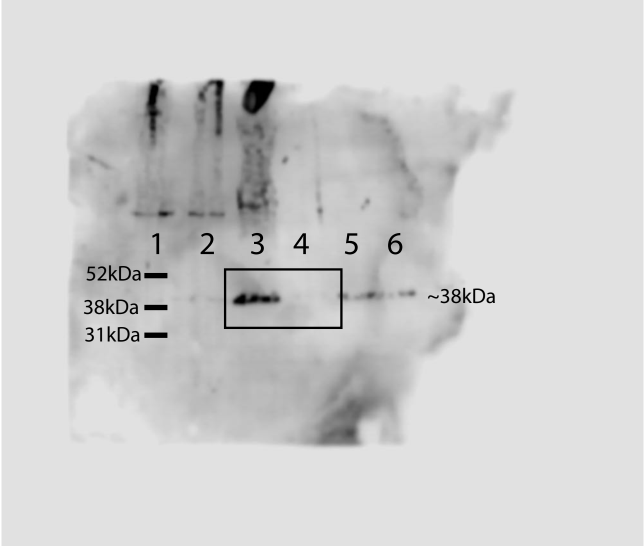

Raw image for Extended Data Fig. 1b western blot stained with Anti-Histone 3
Lane 1 loaded with Rainbow ladder, Lane 3 loaded with stage 10.5 uninjected embryo lysate
Lane 4 loaded with stage 10.5 equivalent NANOG KD embryo lysate. Cropped image shown in inset.

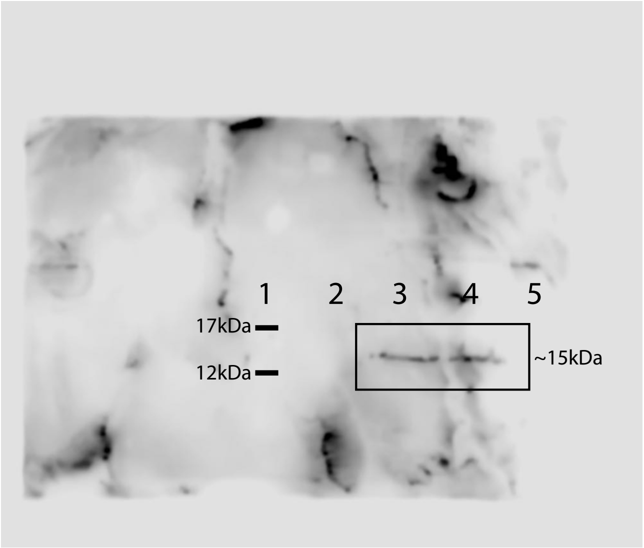

Raw image for Extended Data Fig. 1c Gel
Lane 1 loaded with DNA following PCR of stage 10.5 embryo cDNA using primers directed against NANOG
Lane 2 loaded with DNA following PCR of stage 10.5 control MO injected embryo cDNA using primers directed against NANOG
Lane 3 loaded with DNA following PCR of stage 10.5 equivalent NANOG KD (Splice morpholino) embryo cDNA using primers directed against NANOG
Band b represents the correctly spliced full length cDNA. Bands a, c and d are aberrant splice products

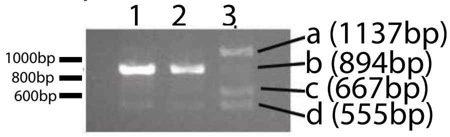

Raw image for Extended Data Fig. 1c Gel
Lane 1 loaded with DNA following PCR of stage 10.5 embryo cDNA using primers directed against ODC1
Lane 2 loaded with DNA following PCR of stage 10.5 control MO injected embryo cDNA using primers directed against ODC1
Lane 3 loaded with DNA following PCR of stage 10.5 equivalent NANOG KD (Splice morpholino) embryo cDNA using primers directed against ODC1
Band a represents the correctly spliced full length cDNA

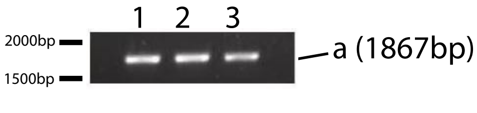

Raw image for Fig. Extended Data Fig.1c NANOG KD (Splice morpholino)

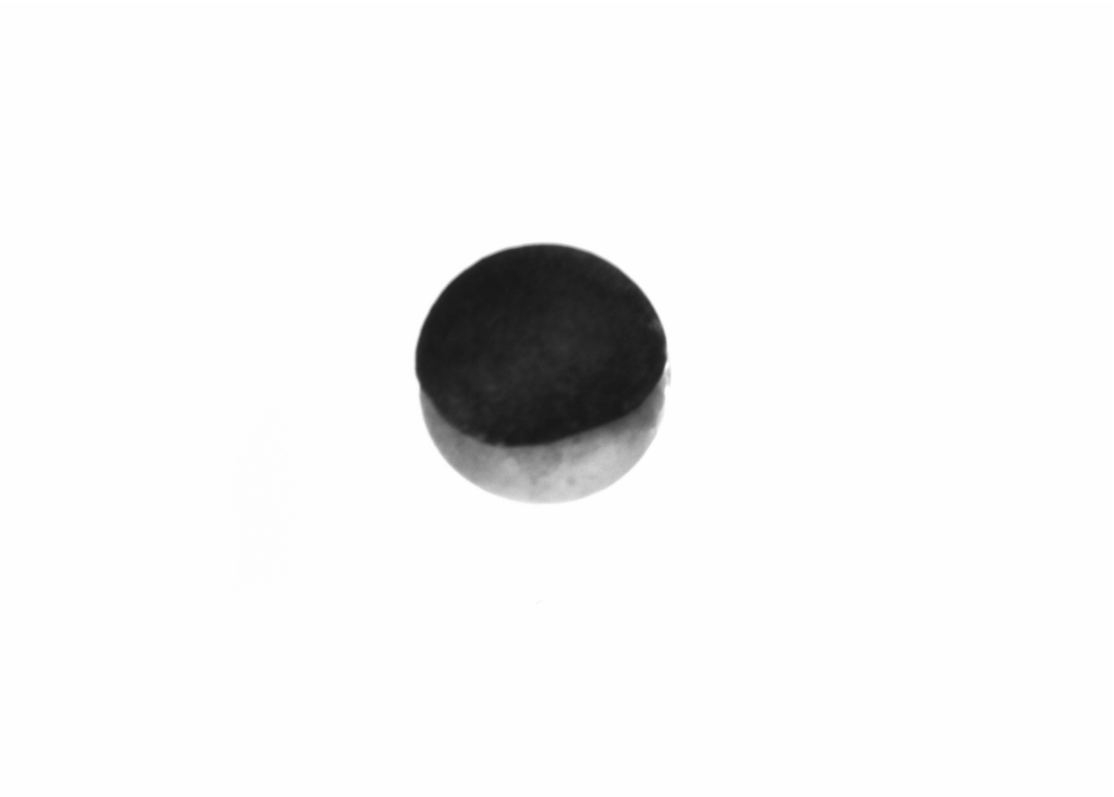

Raw image for Extended Data Fig. 1e Uninjected stage 10.5 embryo HREM 3D reconstruction

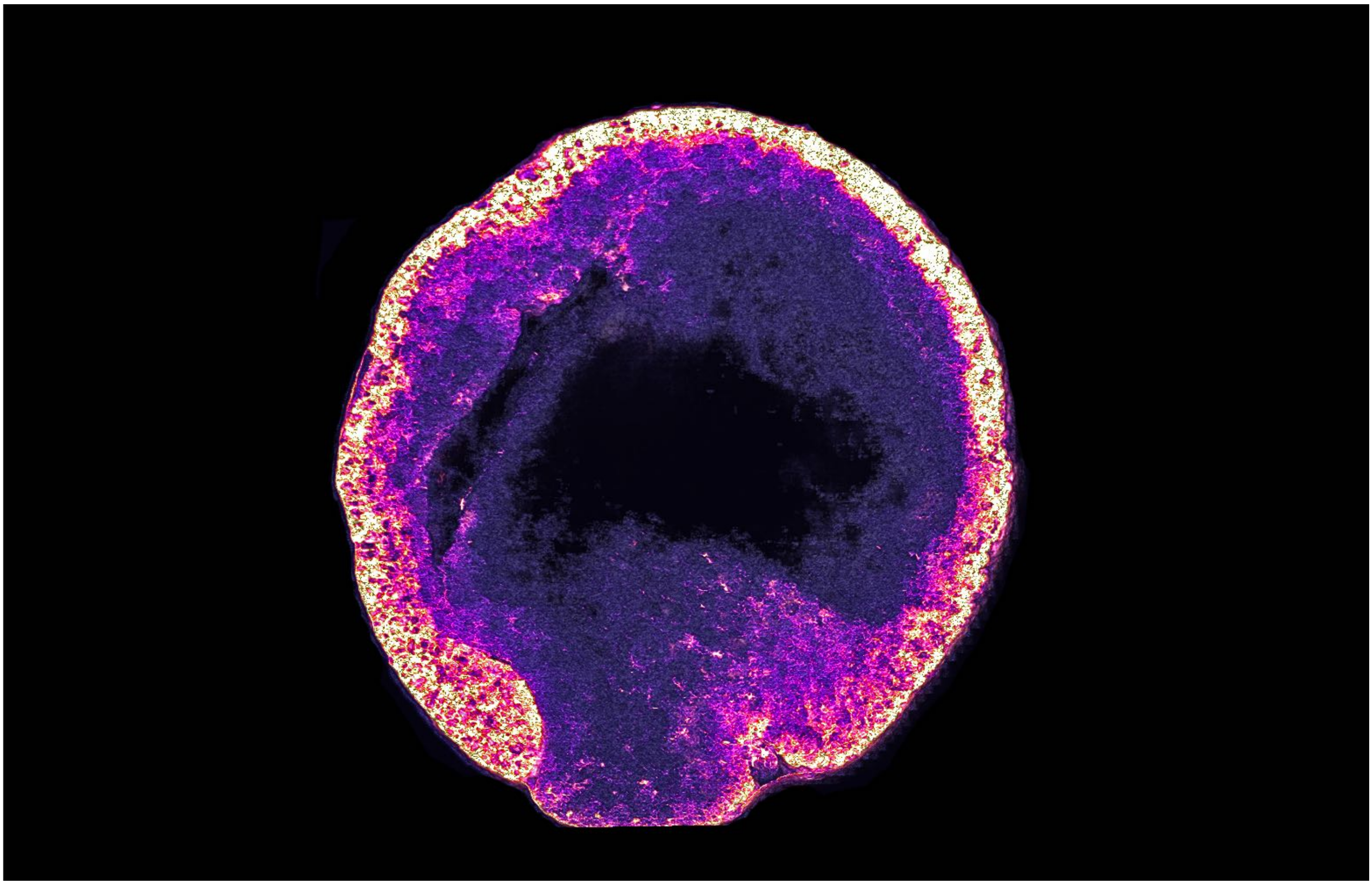

Raw image for Extended Data Fig. 1e equivalent stage 10.5 NANOG KD embryo HREM 3D reconstruction

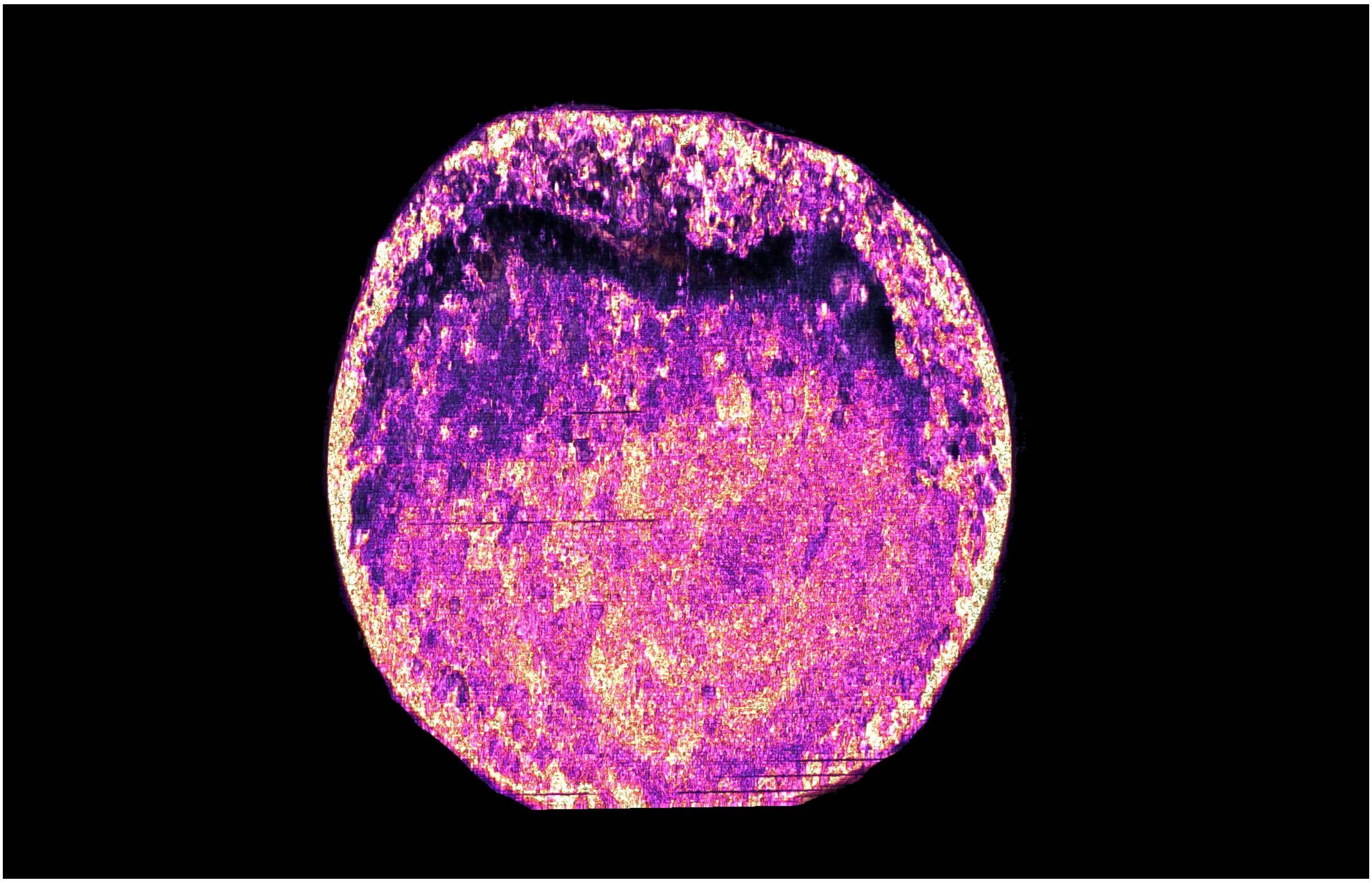

Raw image for Extended Data Fig. 1f Uninjected stage 40 embryo HREM 3D reconstruction

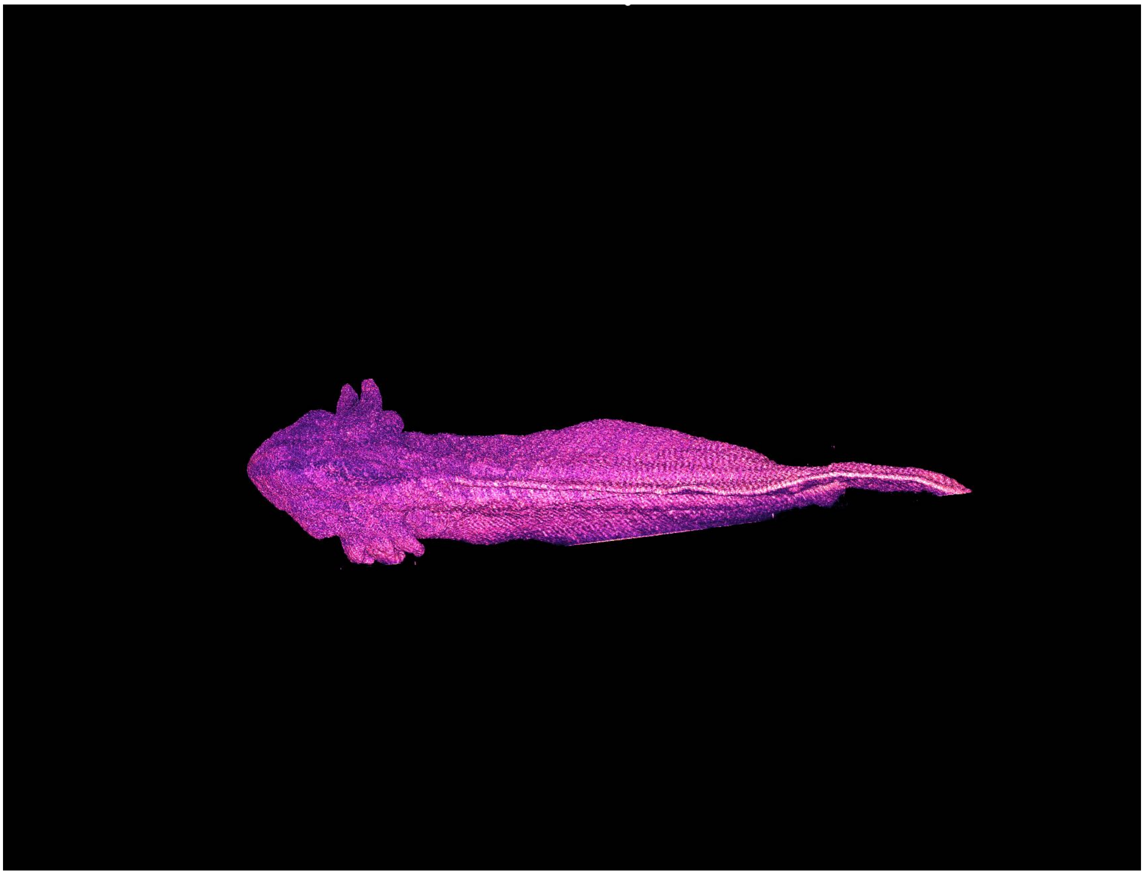

Raw image for Extended Data Fig. 1f equivalent stage 40 NANOG KD + HNANOG embryo HREM 3D reconstruction

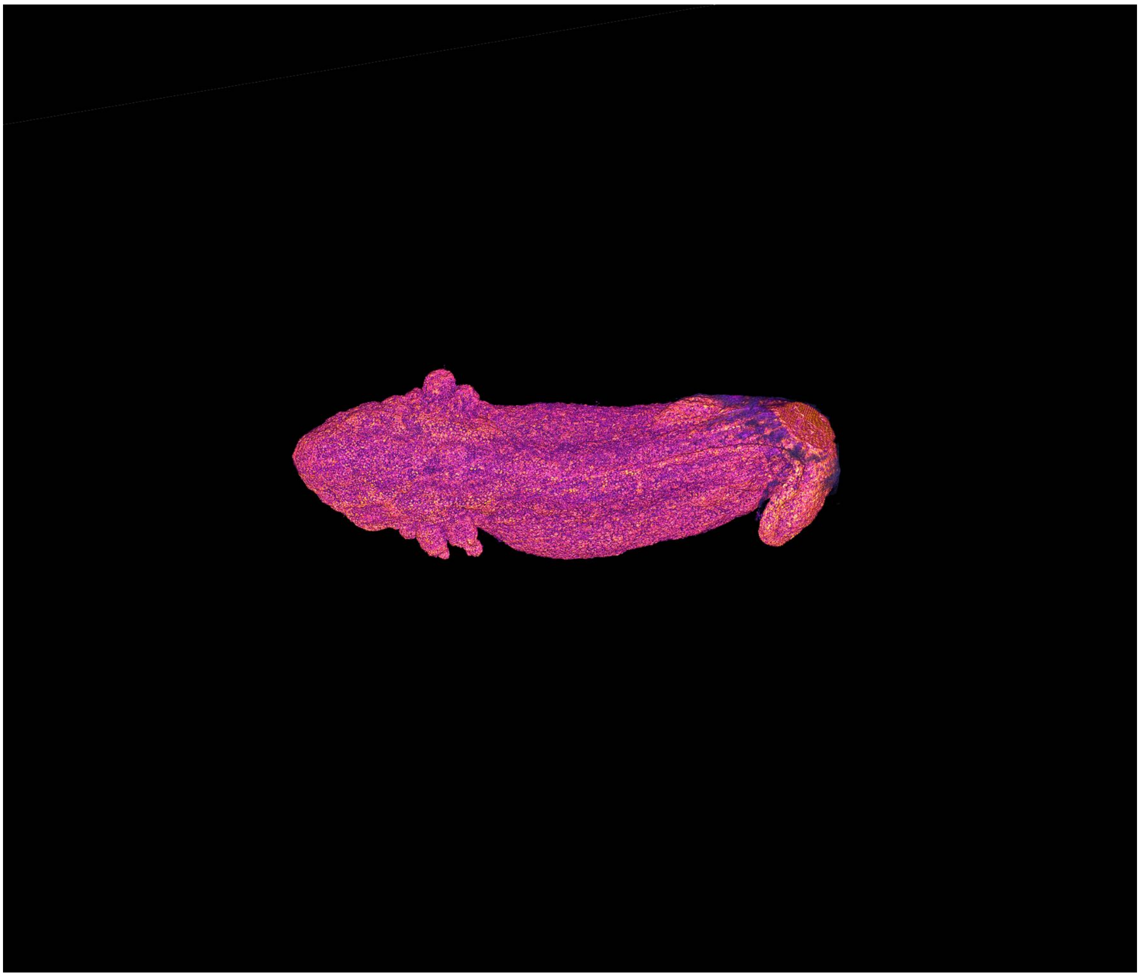

Raw image for Extended Data Fig. 5a Uninjected stage 10.5 animal cap stained for H3

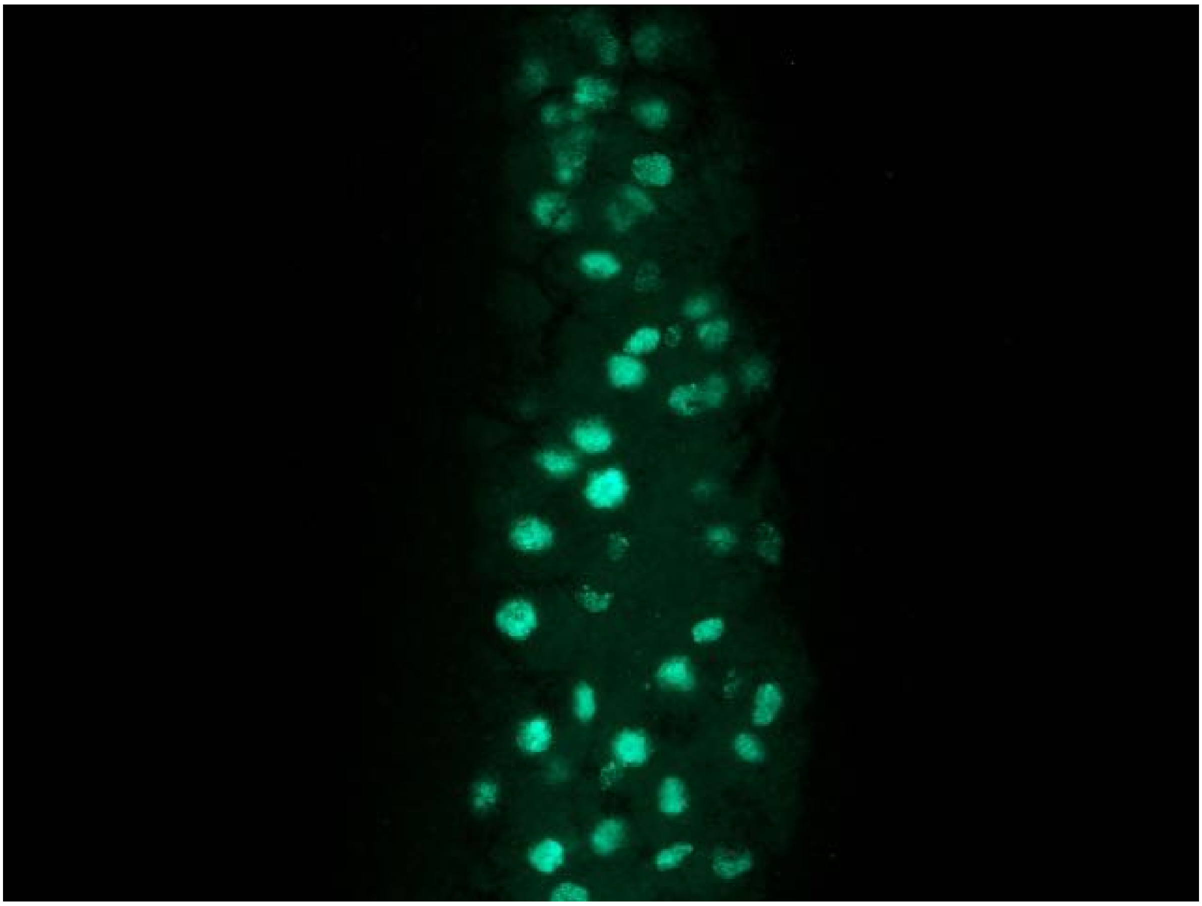

Raw image for Extended Data Fig. 5a Uninjected stage 10.5 animal cap stained for DAPI

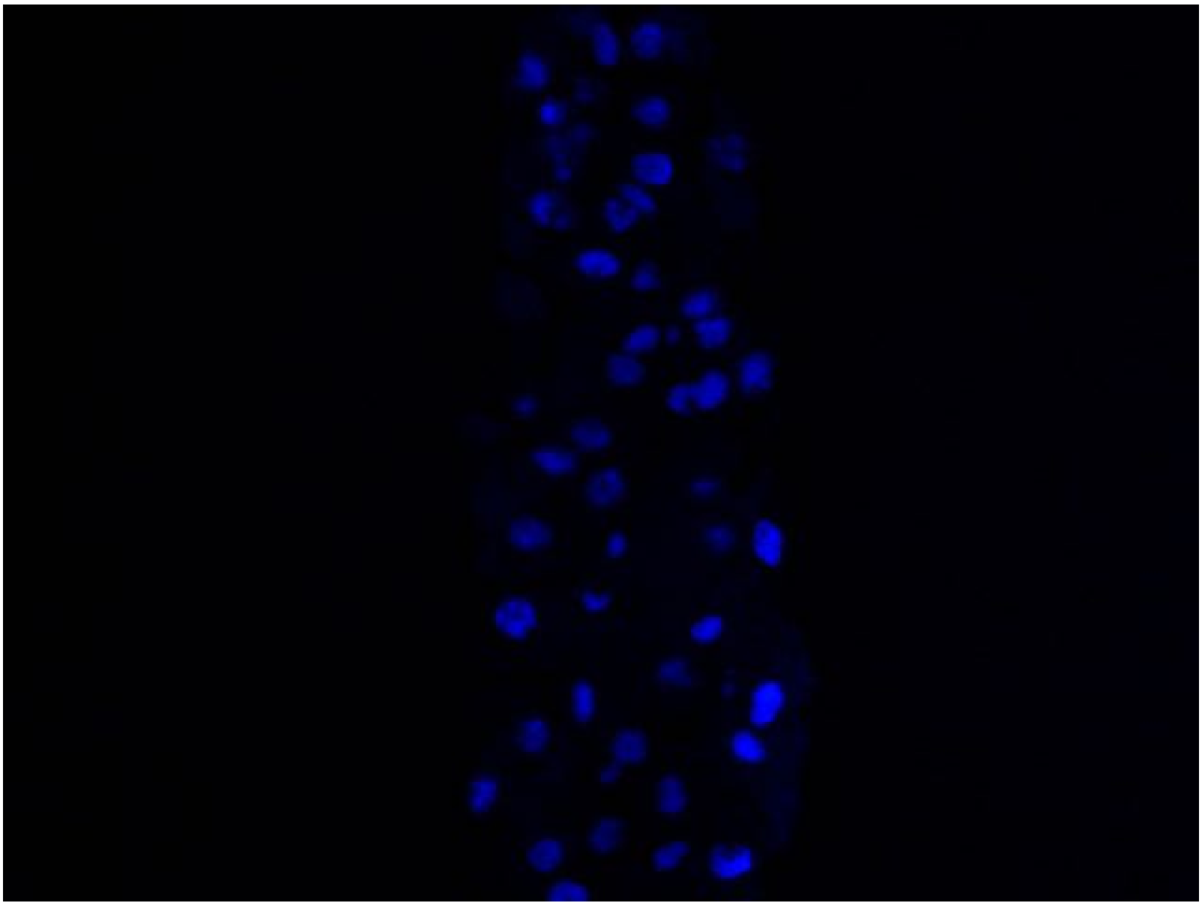

Raw image for Extended Data Fig. 5a stage 10.5 equivalent NANOG KD animal cap stained for H3

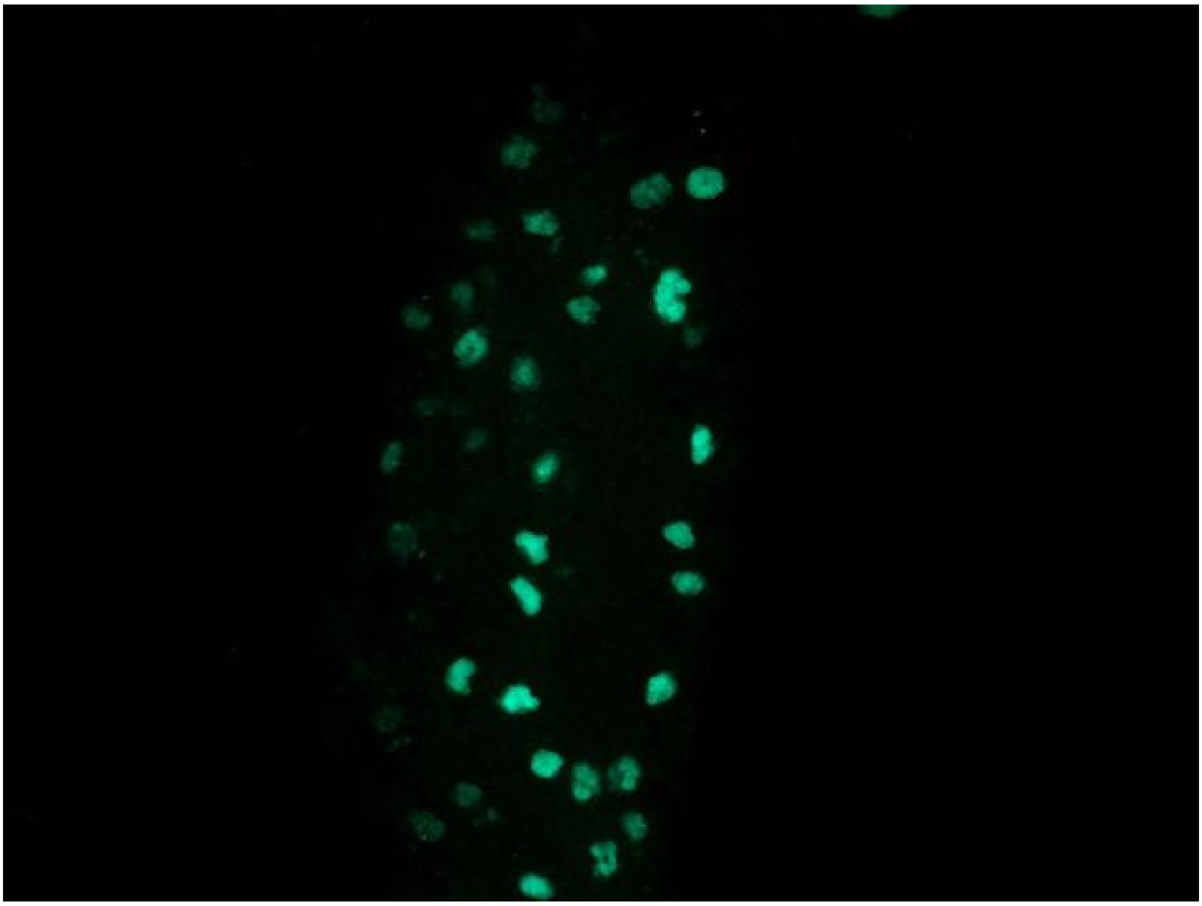

Raw image for Extended Data Fig. 5a stage 10.5 equivalent NANOG KD animal cap stained for DAPI

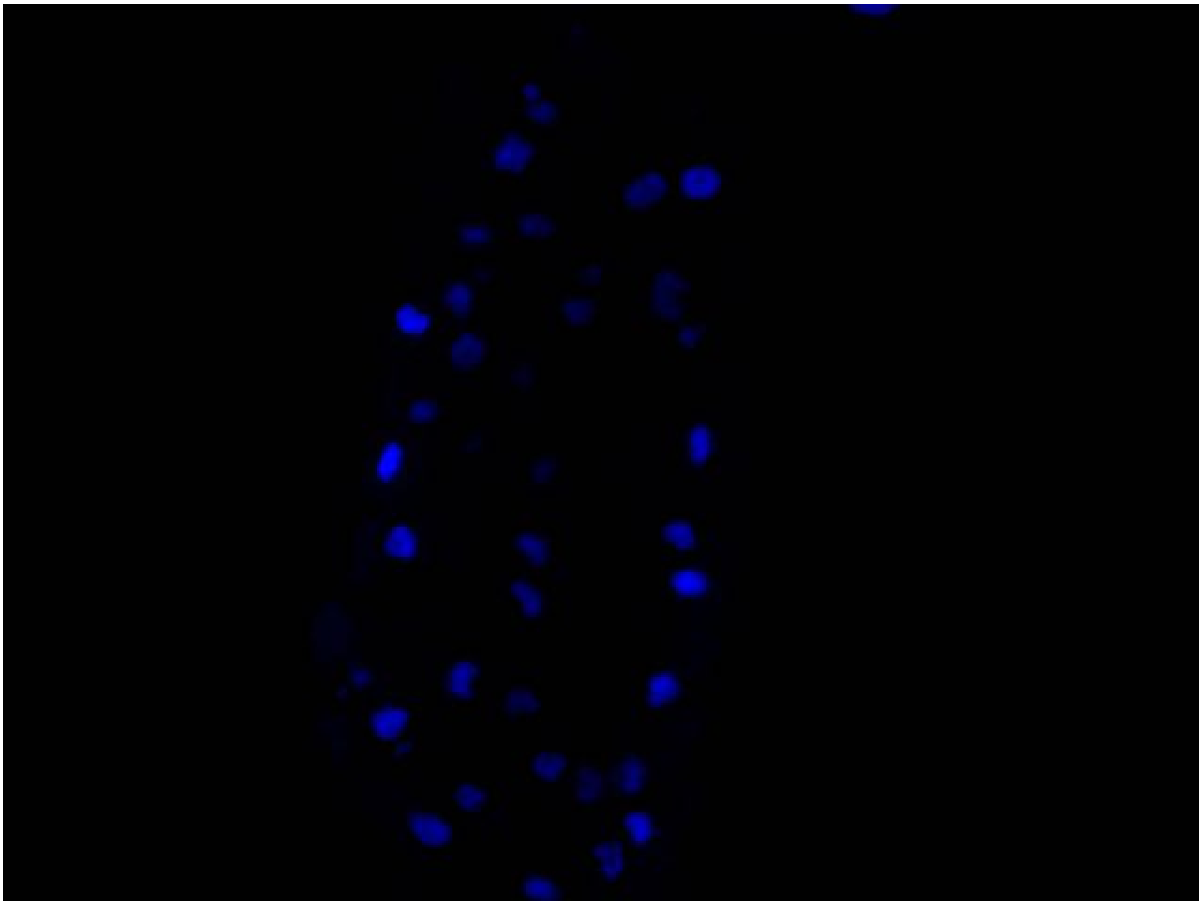

Raw image for Extended Data Fig. 5a stage 10.5 equivalent NANOG KD + HNANOG animal cap stained for H3

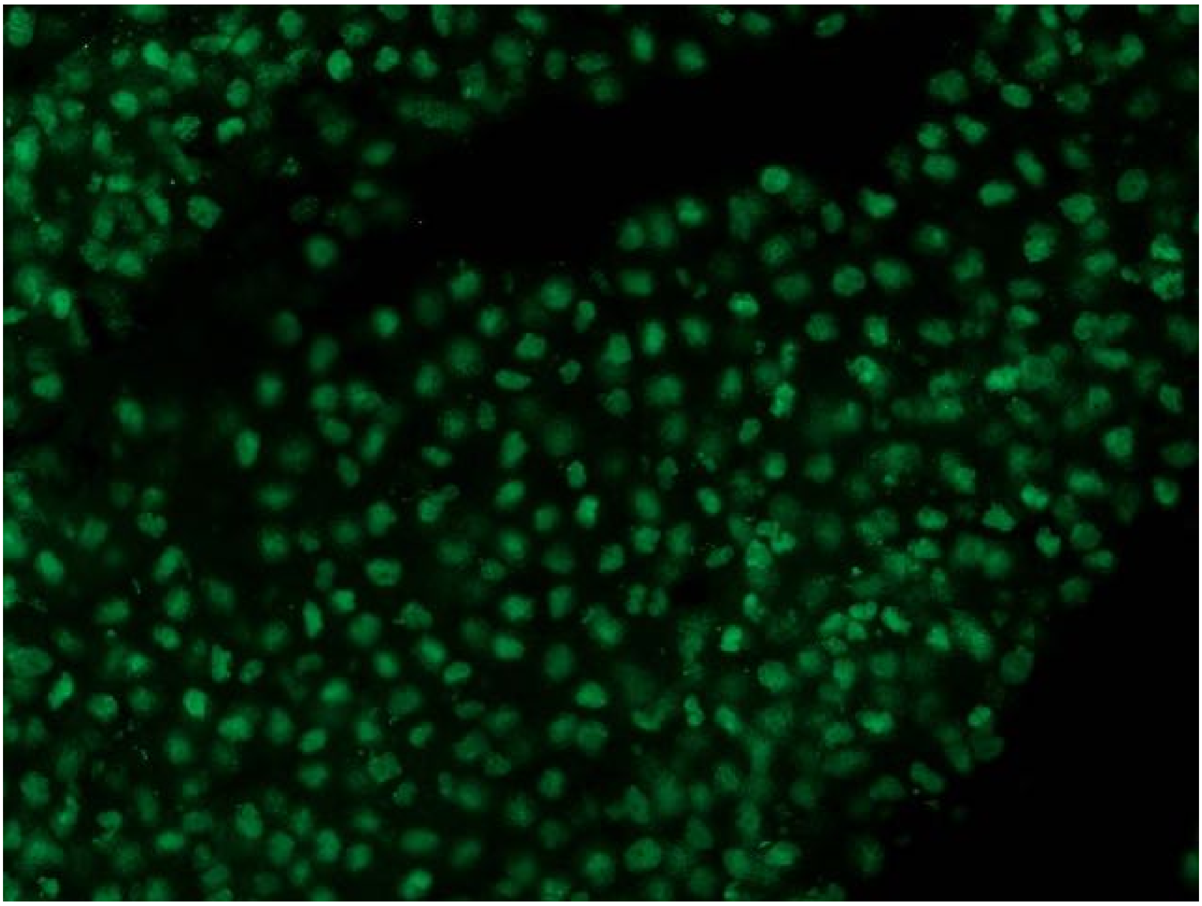

Raw image for Extended Data Fig. 5a stage 10.5 equivalent NANOG KD + HNANOG animal cap stained for DAPI

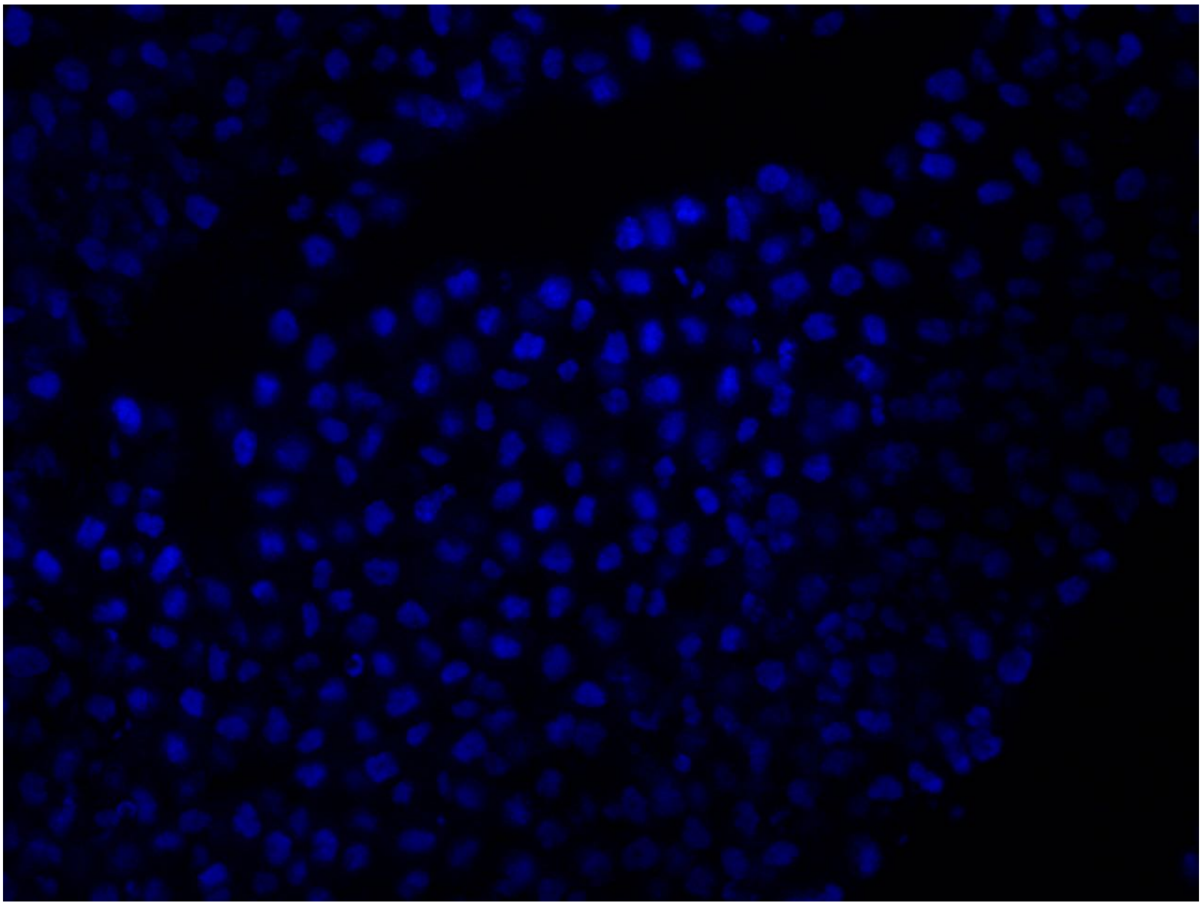

Raw image for Extended Data Fig. 5a Uninjected stage 10.5 animal cap stained for H3K36me3

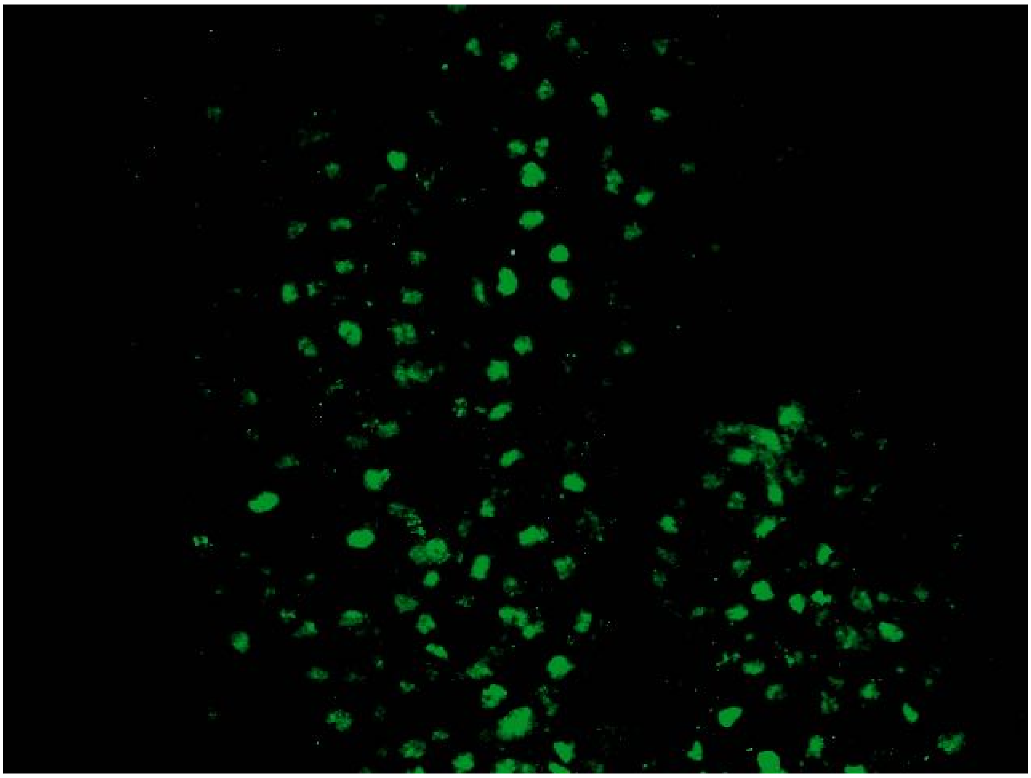

Raw image for Extended Data Fig. 5a Uninjected stage 10.5 animal cap stained for DAPI

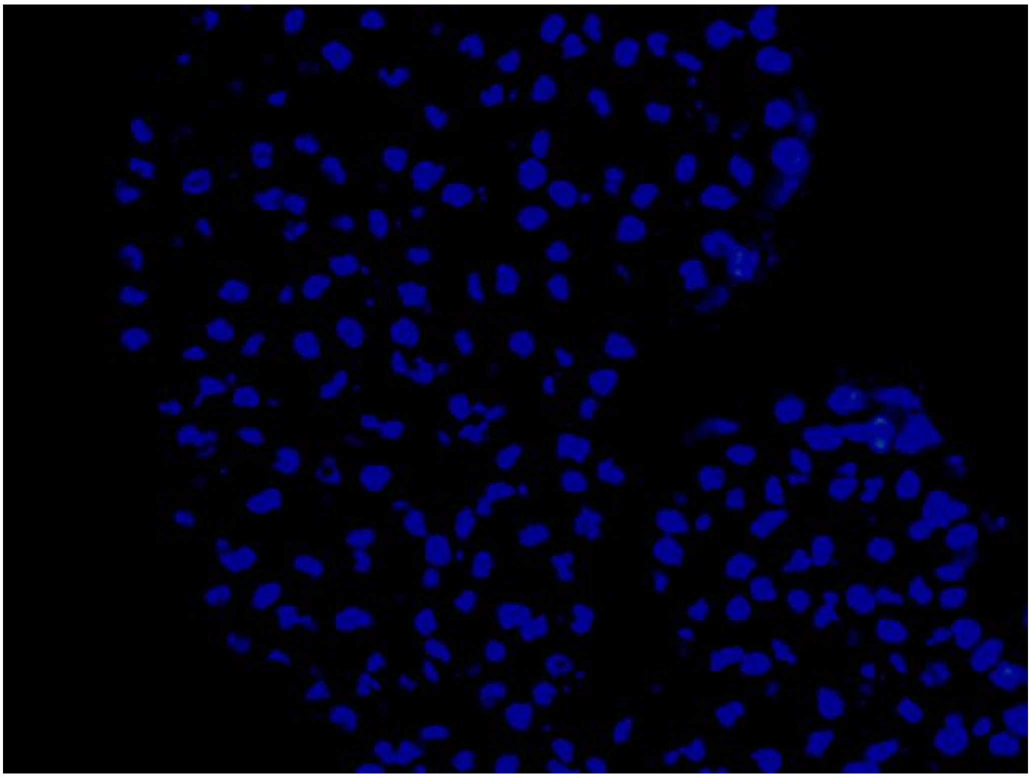

Raw image for Extended Data Fig. 5a NANOG KD 10.5 animal cap stained for H3K36me3

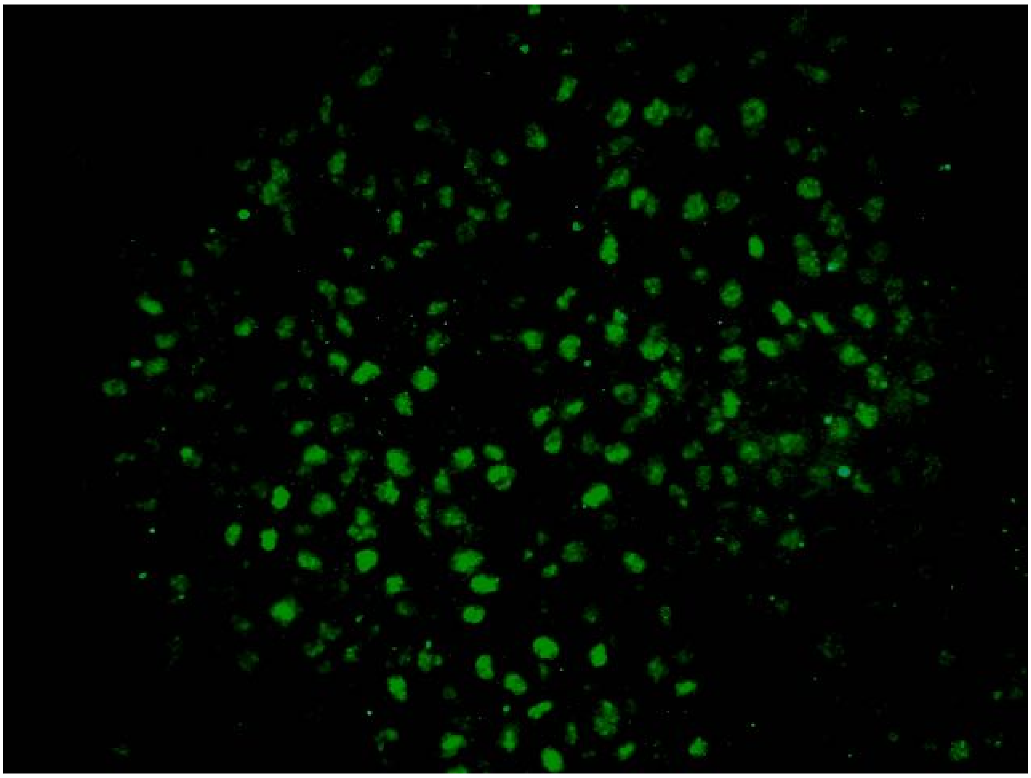

Raw image for Extended Data Fig. 5a NANOG KD stage 10.5 animal cap stained for DAPI

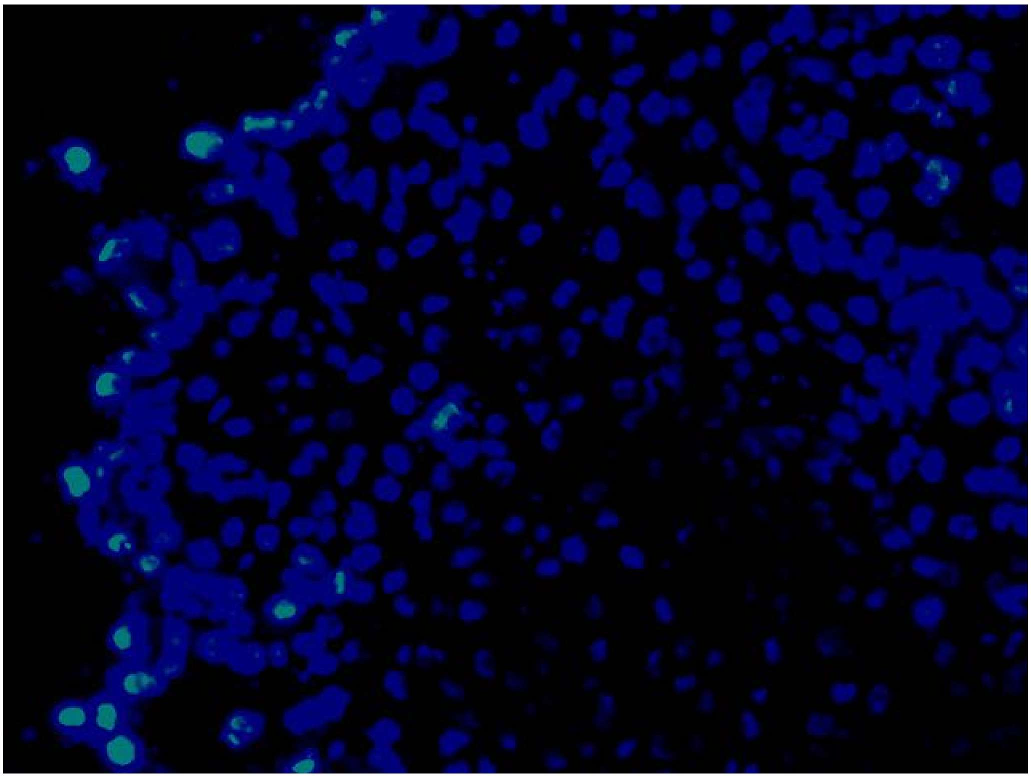

Raw image for Extended Data Fig. 5a NANOG KD 10.5 animal cap stained for H3K36me3

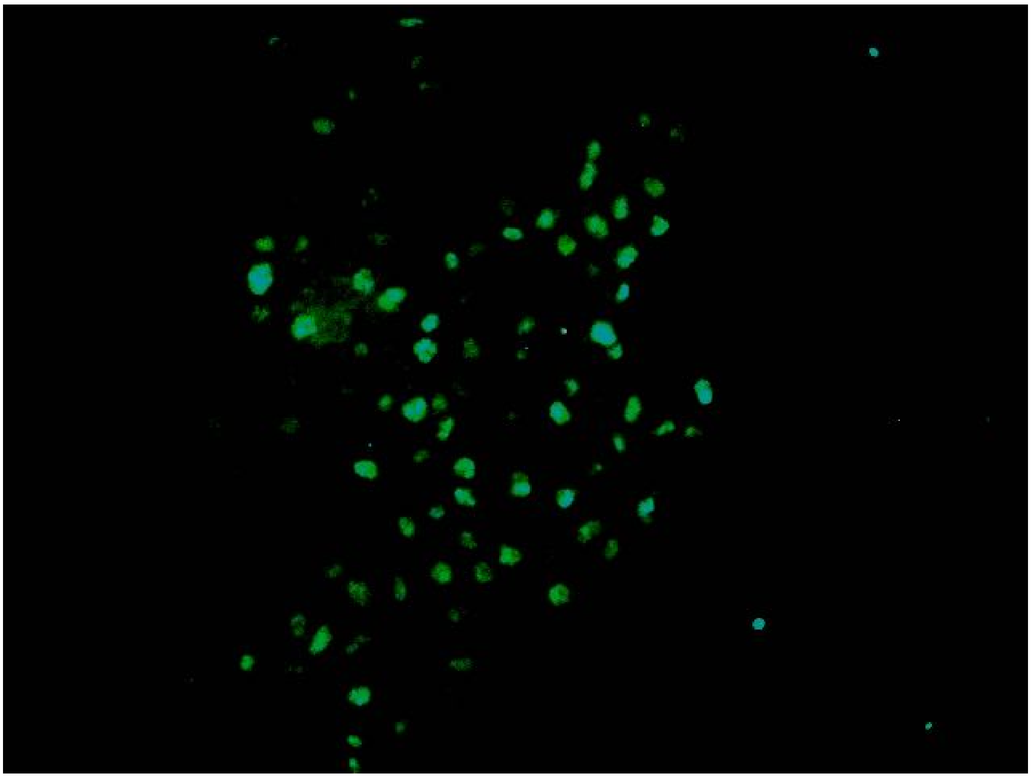

Raw image for Extended Data Fig. 5a NANOG KD stage 10.5 animal cap stained for DAPI

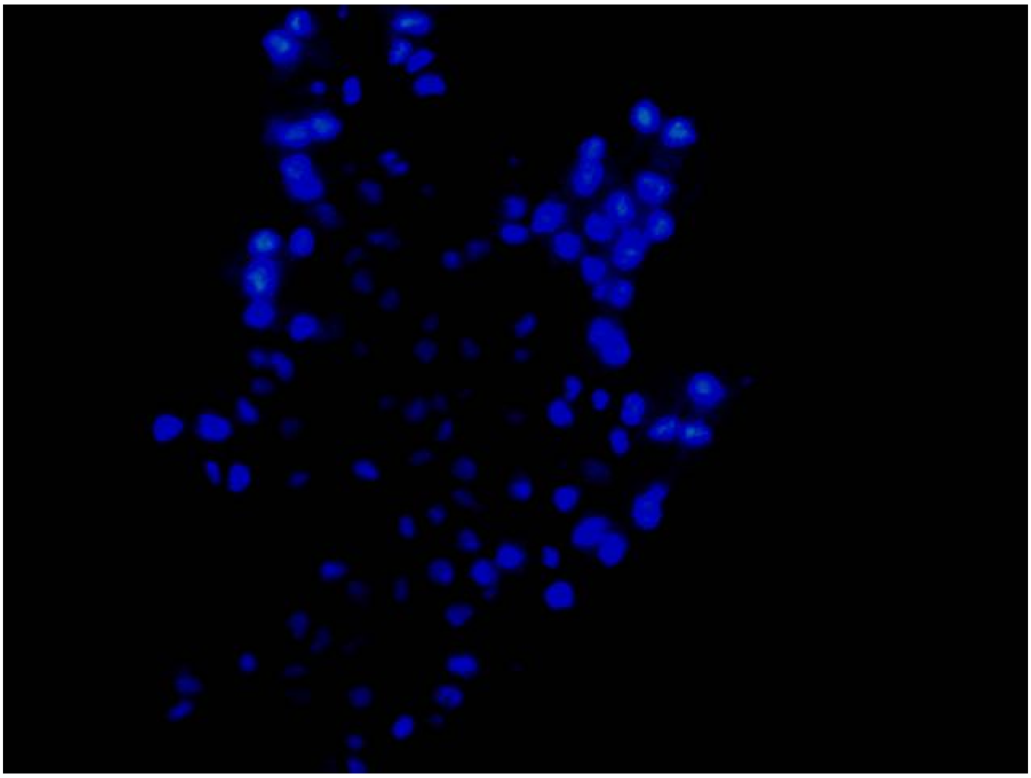

Raw image for Fig. 5b Untreated stage 10.5 animal cap stained for H3

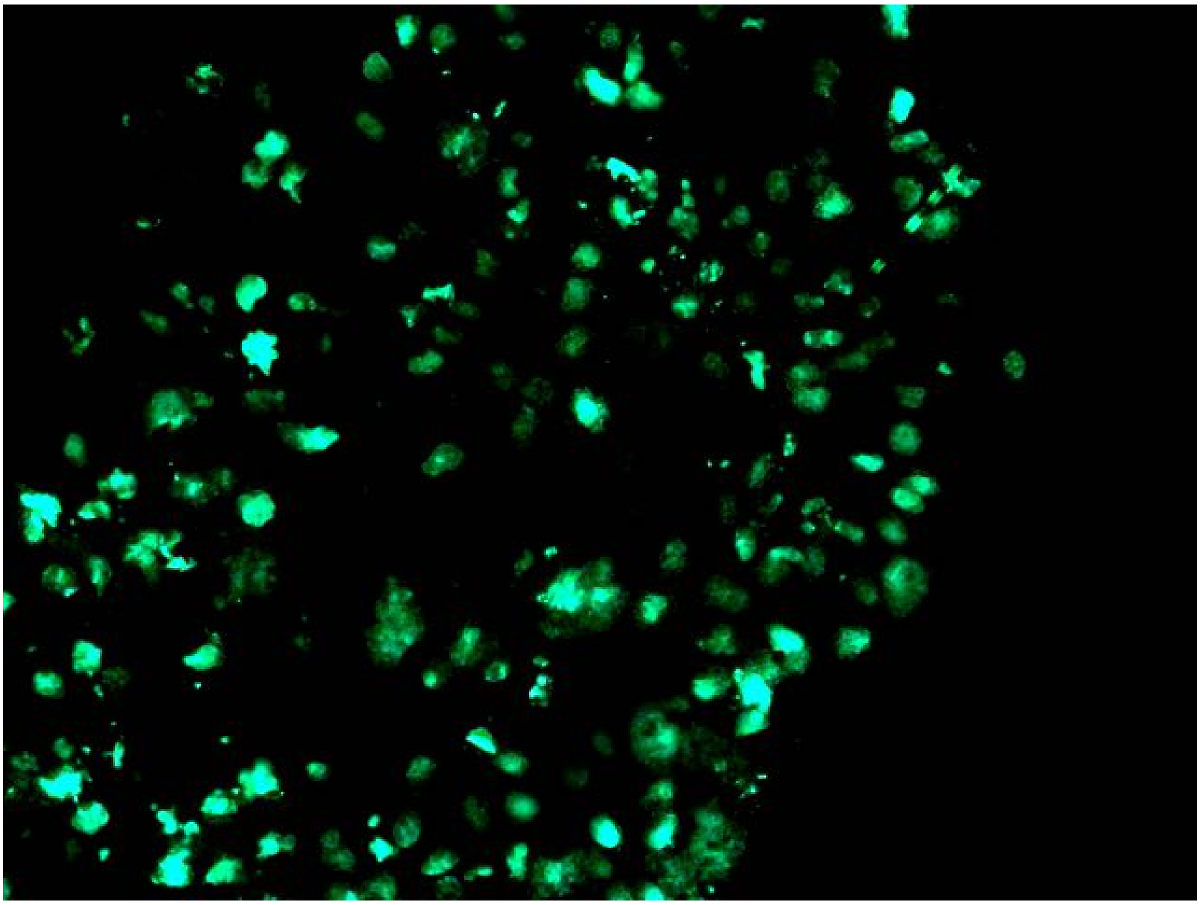

Raw image for Fig. 5b Untreated stage 10.5 animal cap stained for DAPI

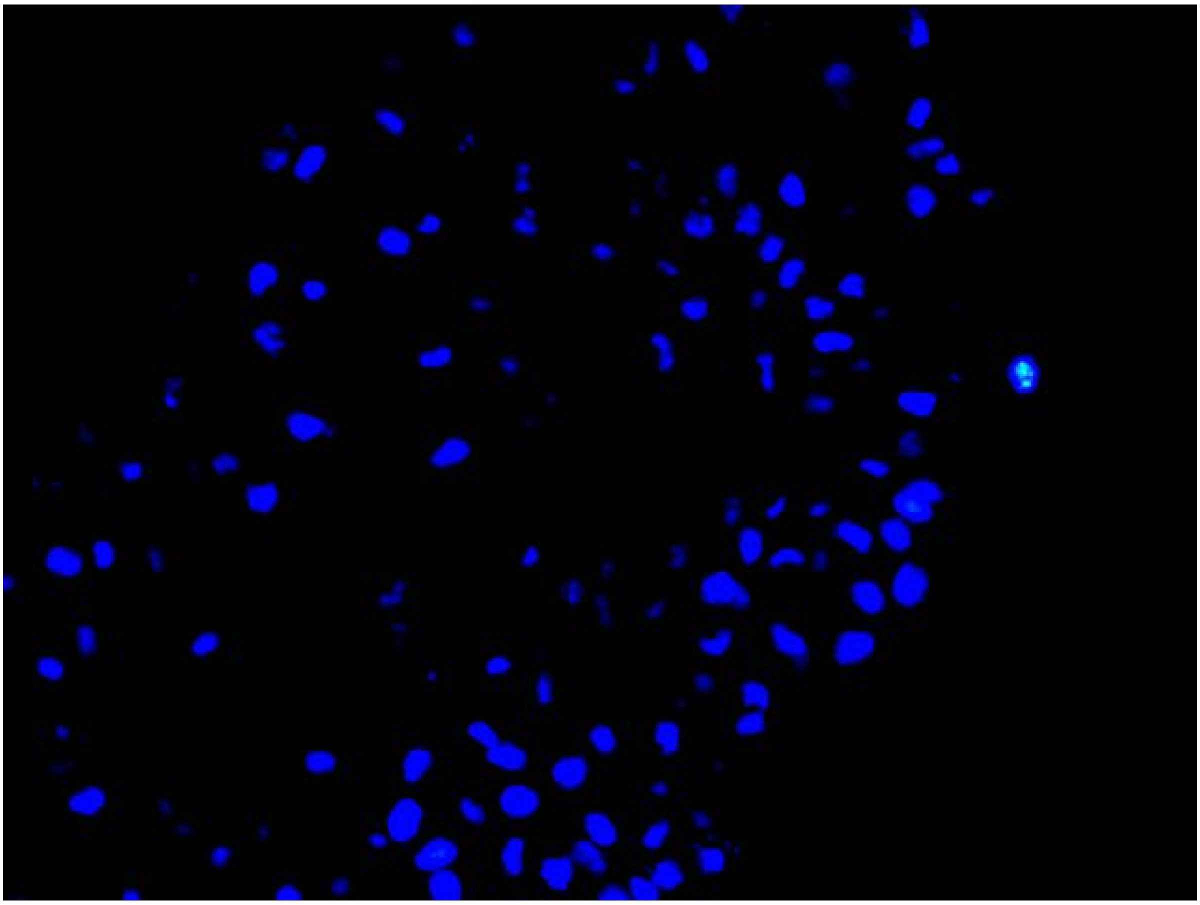

Raw image for Extended Data Fig. 5b stage 10.5 equivalent SB431542 treated animal cap stained for H3

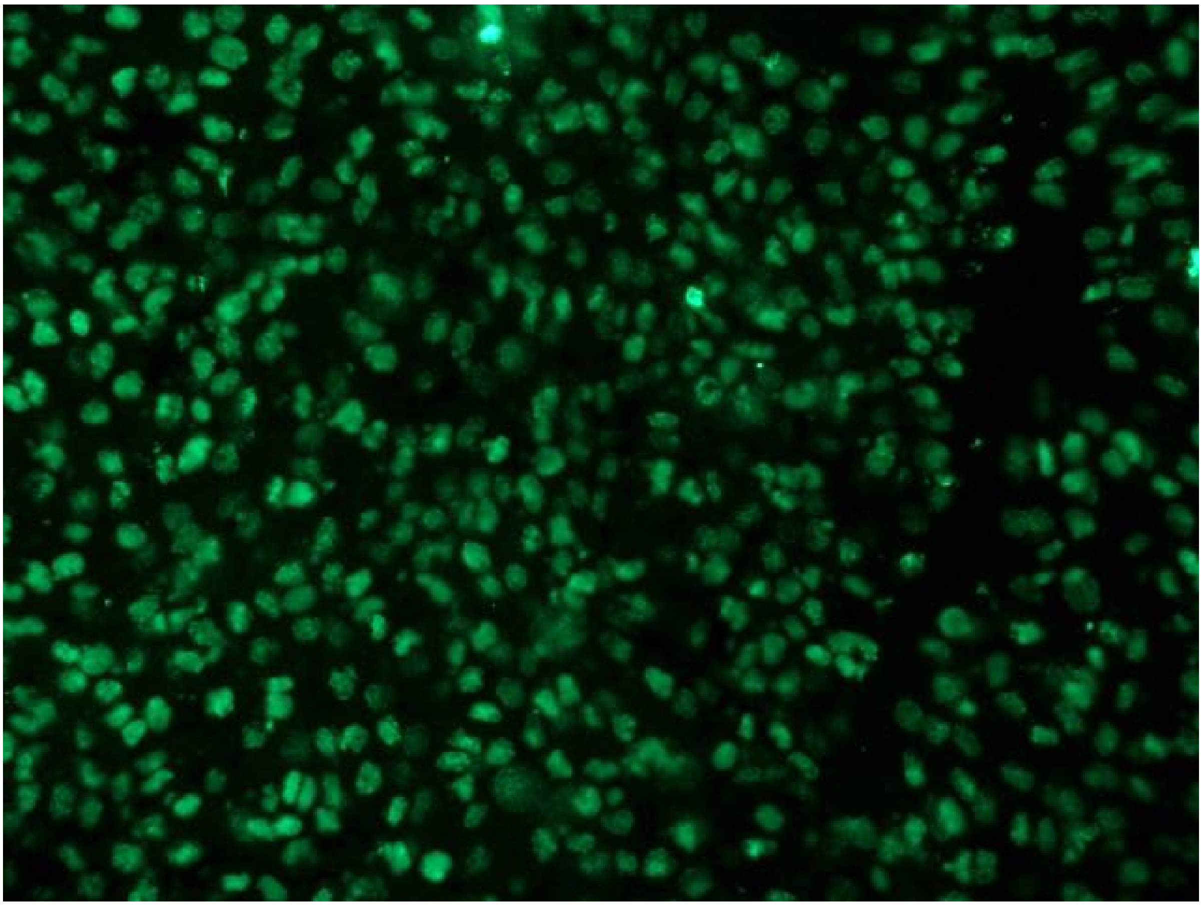

Raw image for Extended Data Fig. 5b stage 10.5 equivalent SB431542 treated animal cap stained for DAPI

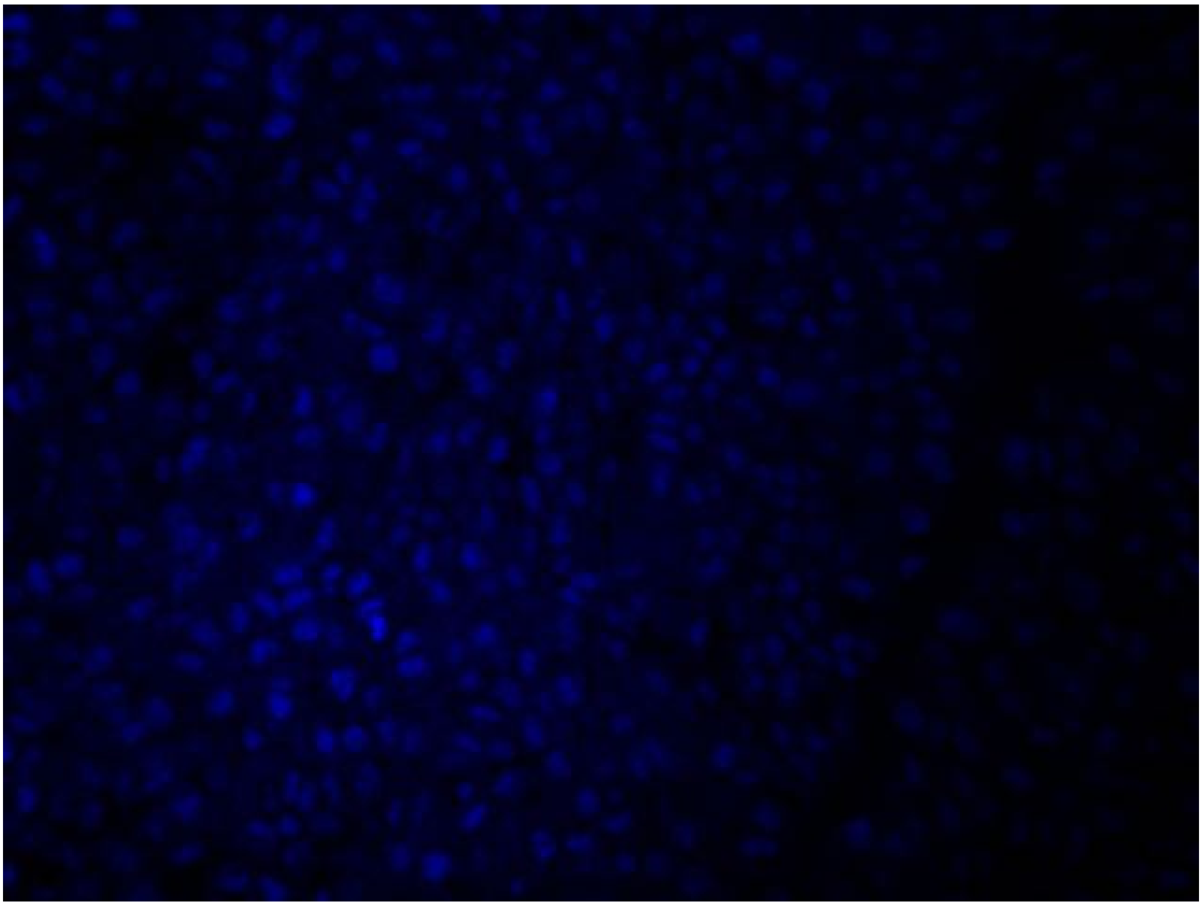

Raw image for Extended Data Fig. 5b stage 10.5 DPY30 KD animal cap stained for H3

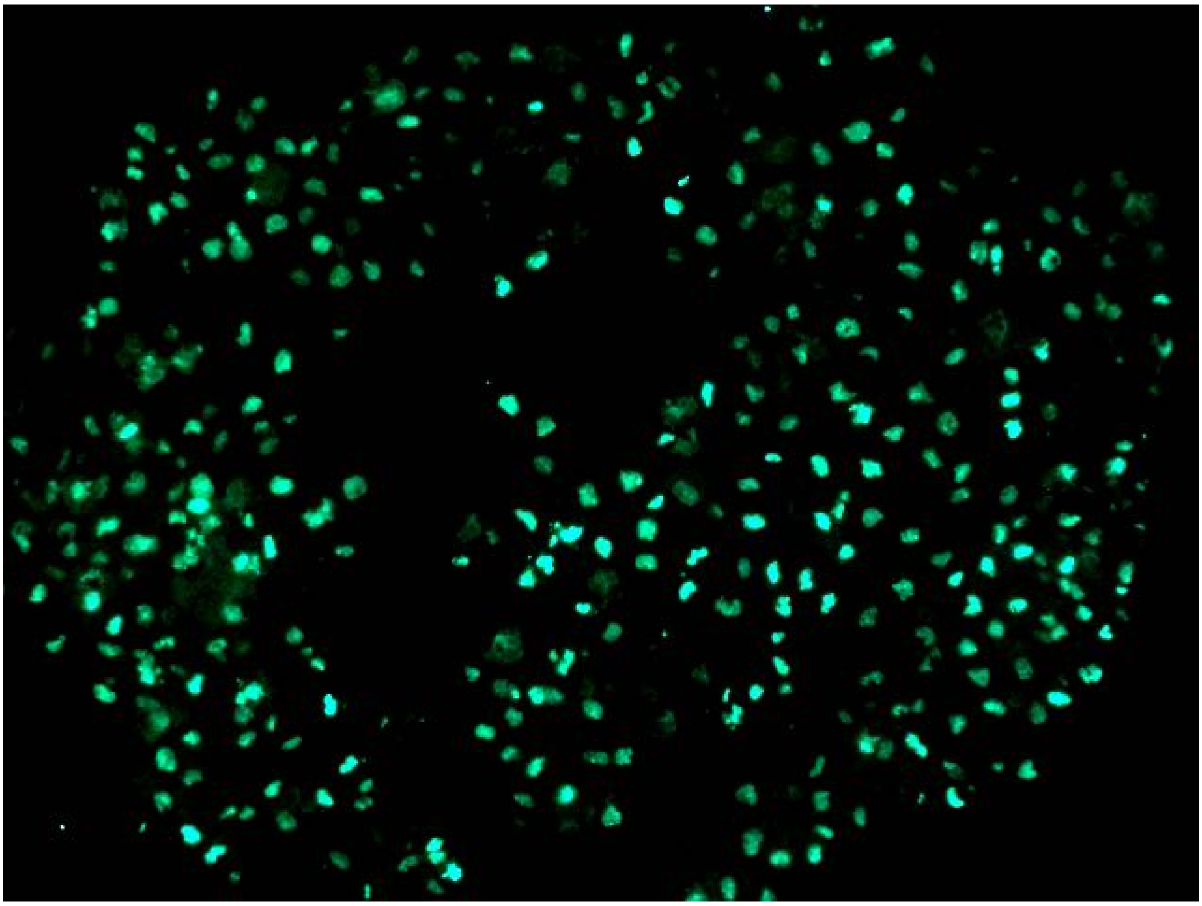

Raw image for Extended Data Fig. 5b stage 10.5 DPY30 KD animal cap stained for DAPI

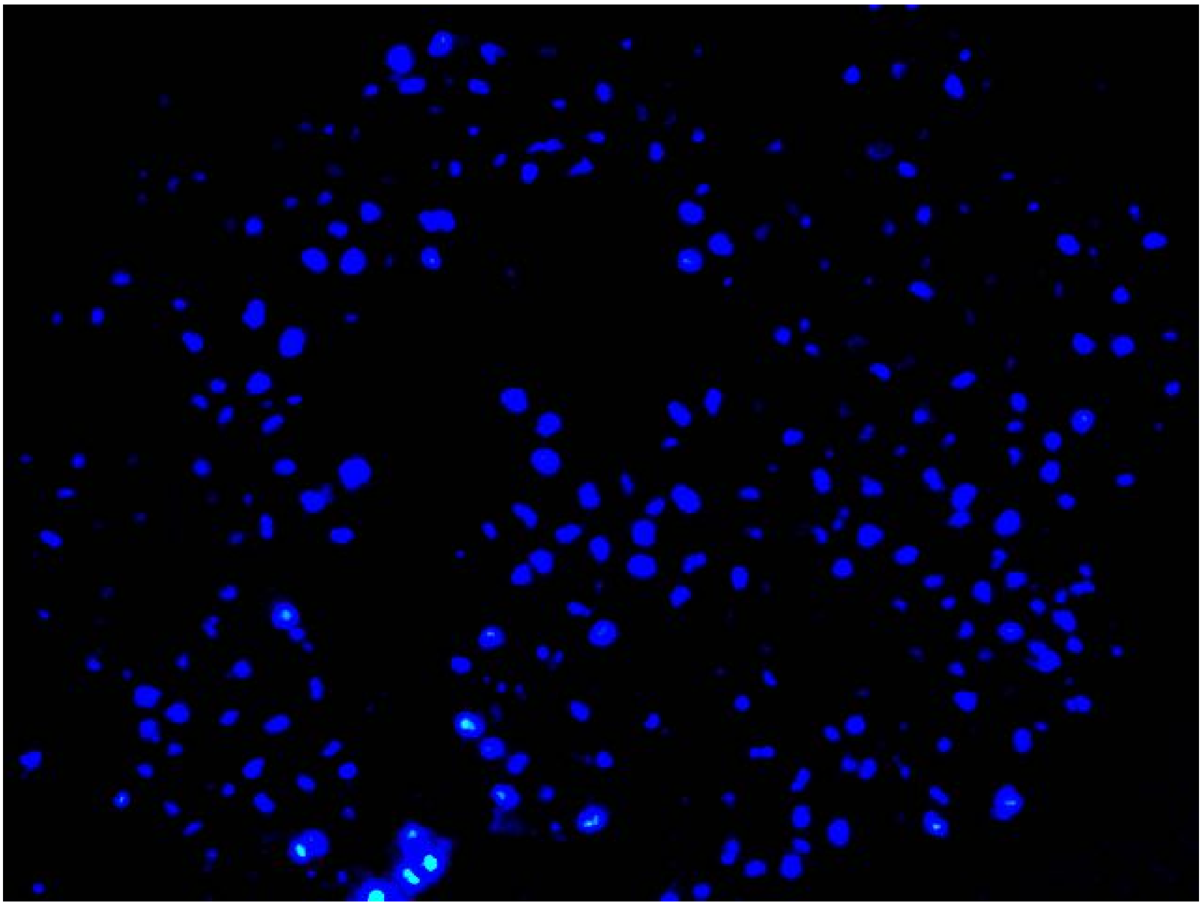

Raw image for Extended Data Fig. 5b Untreated stage 10.5 animal cap stained for H3K36me3

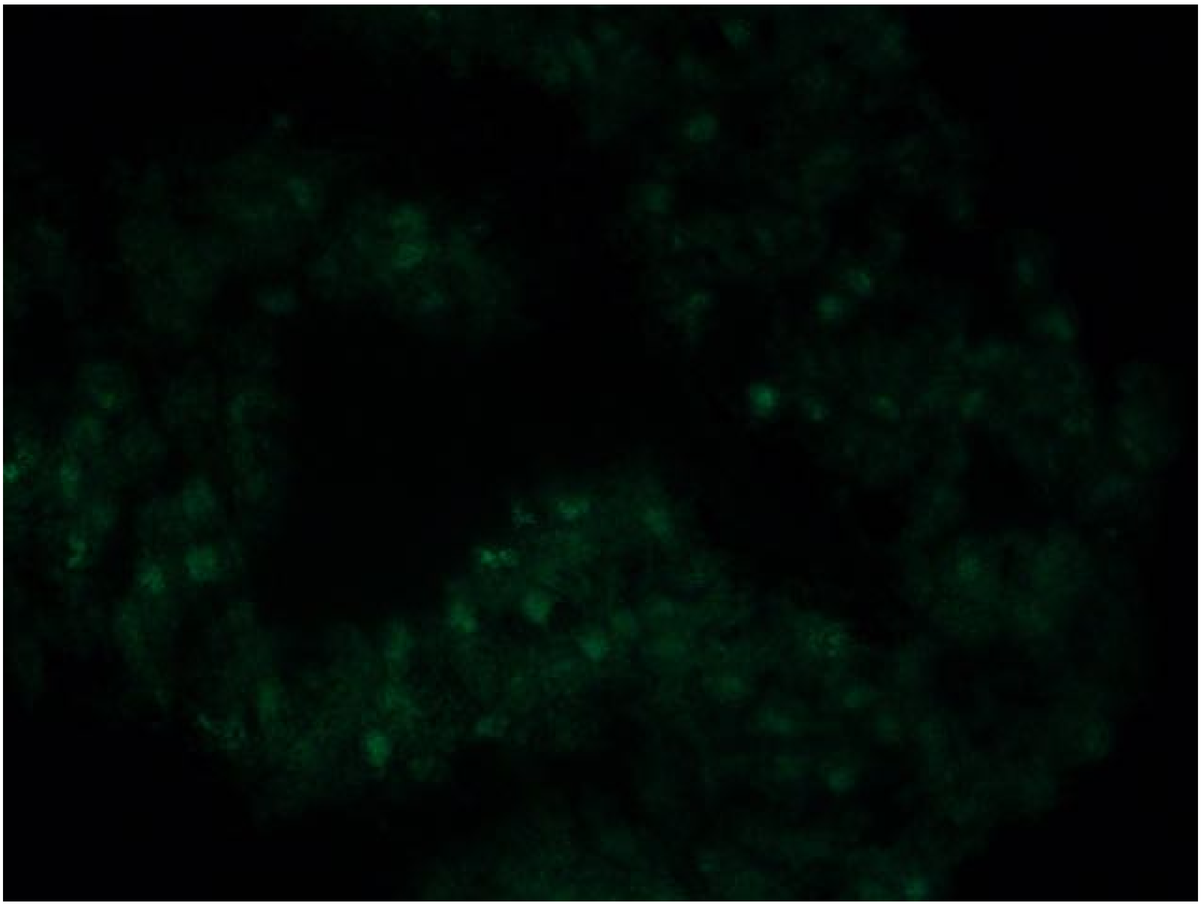

Raw image for Extended Data Fig. 5b Untreated stage 10.5 animal cap stained for DAPI

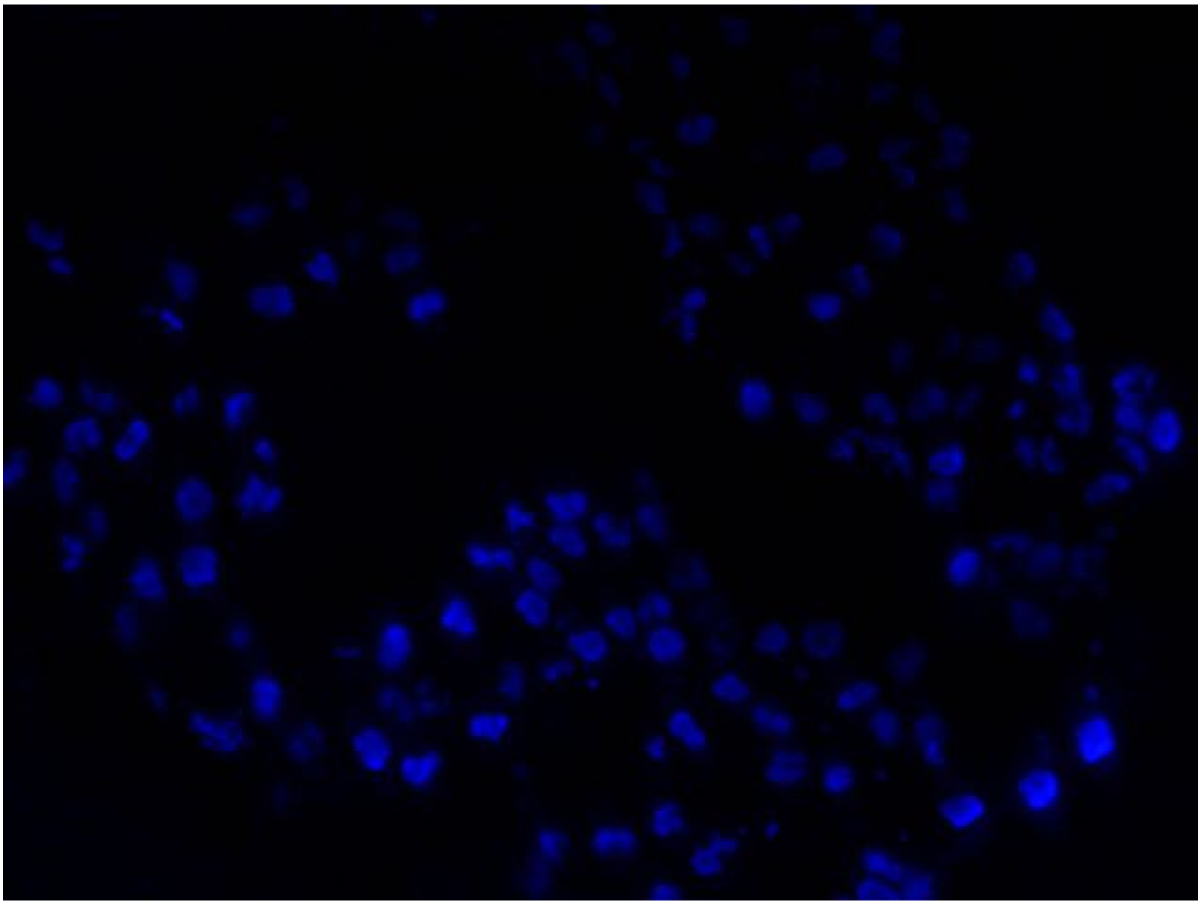

Raw image for Extended Data Fig. 5.b SB431542 treated stage 10.5 animal cap stained for H3K36me3

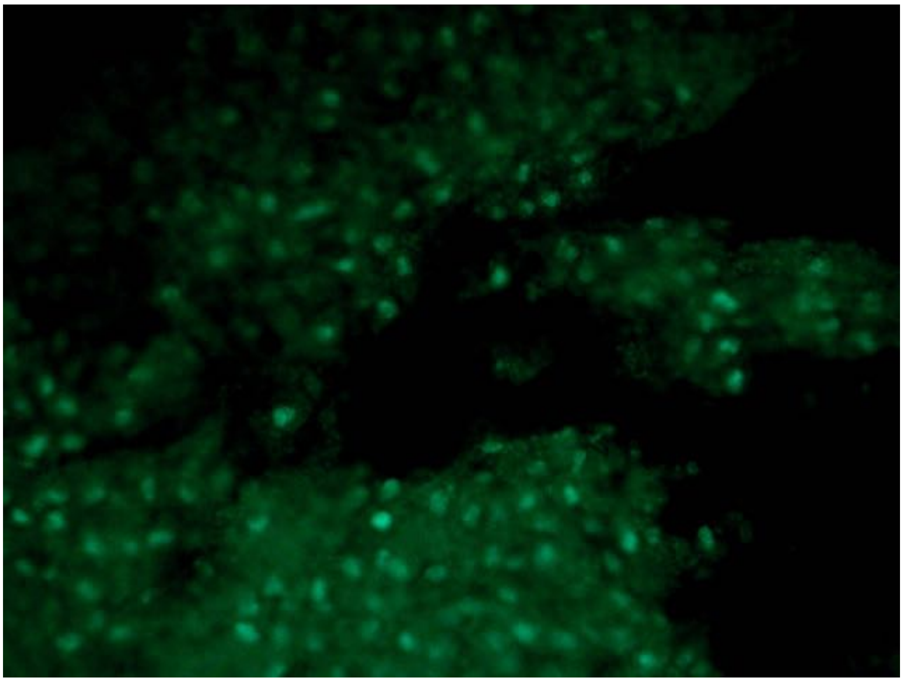

Raw image for Extended Data Fig. 5.b SB431542 treated stage 10.5 animal cap stained for DAPI

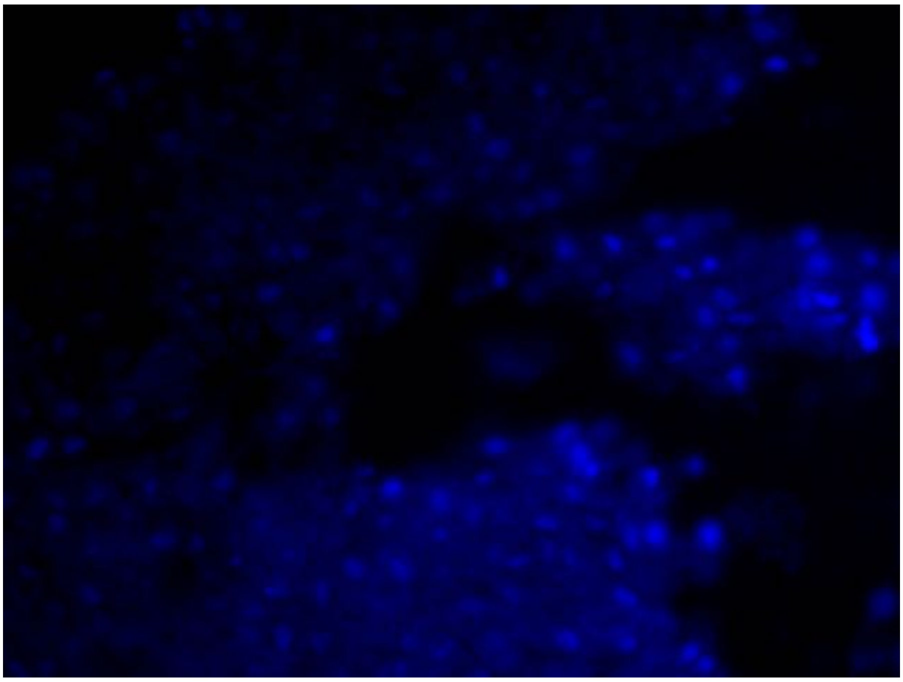

Raw image for Extended Data Fig. 5b stage 10.5 DPY30 KD animal cap stained for H3K36me3

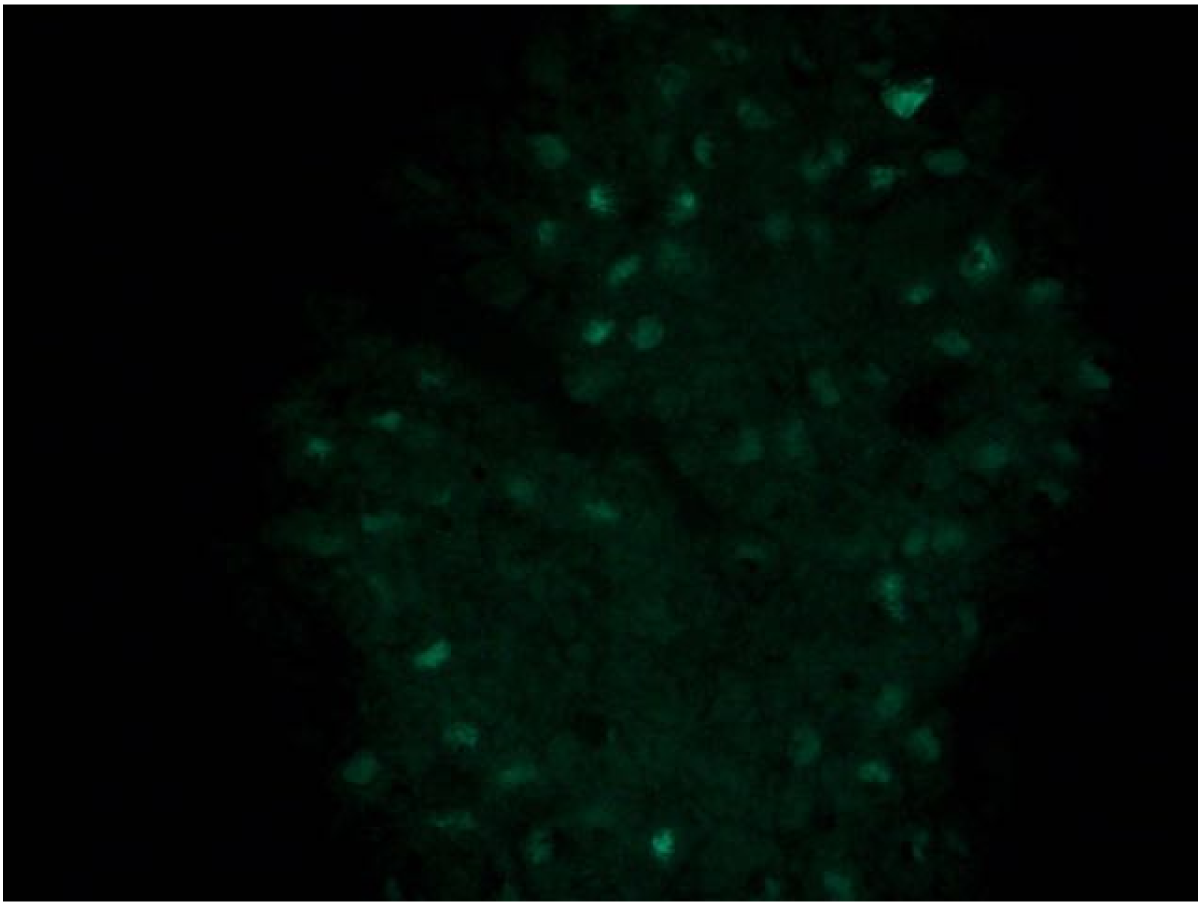

Raw image for Extended Data Fig. 5b stage 10.5 DPY30 KD animal cap stained for DAPI

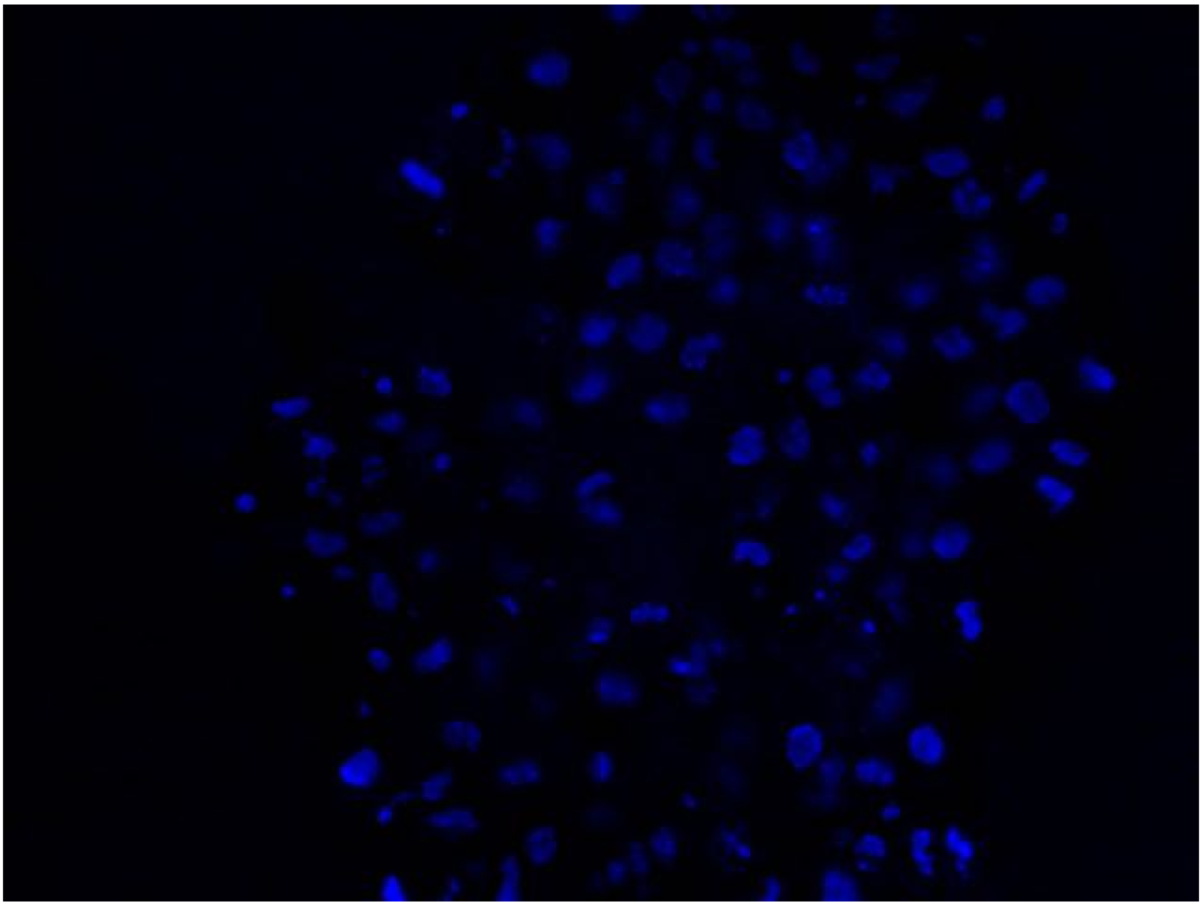

Raw image for Extended Data Fig. 5c Uninjected stage 10.5 animal cap stained for POLII

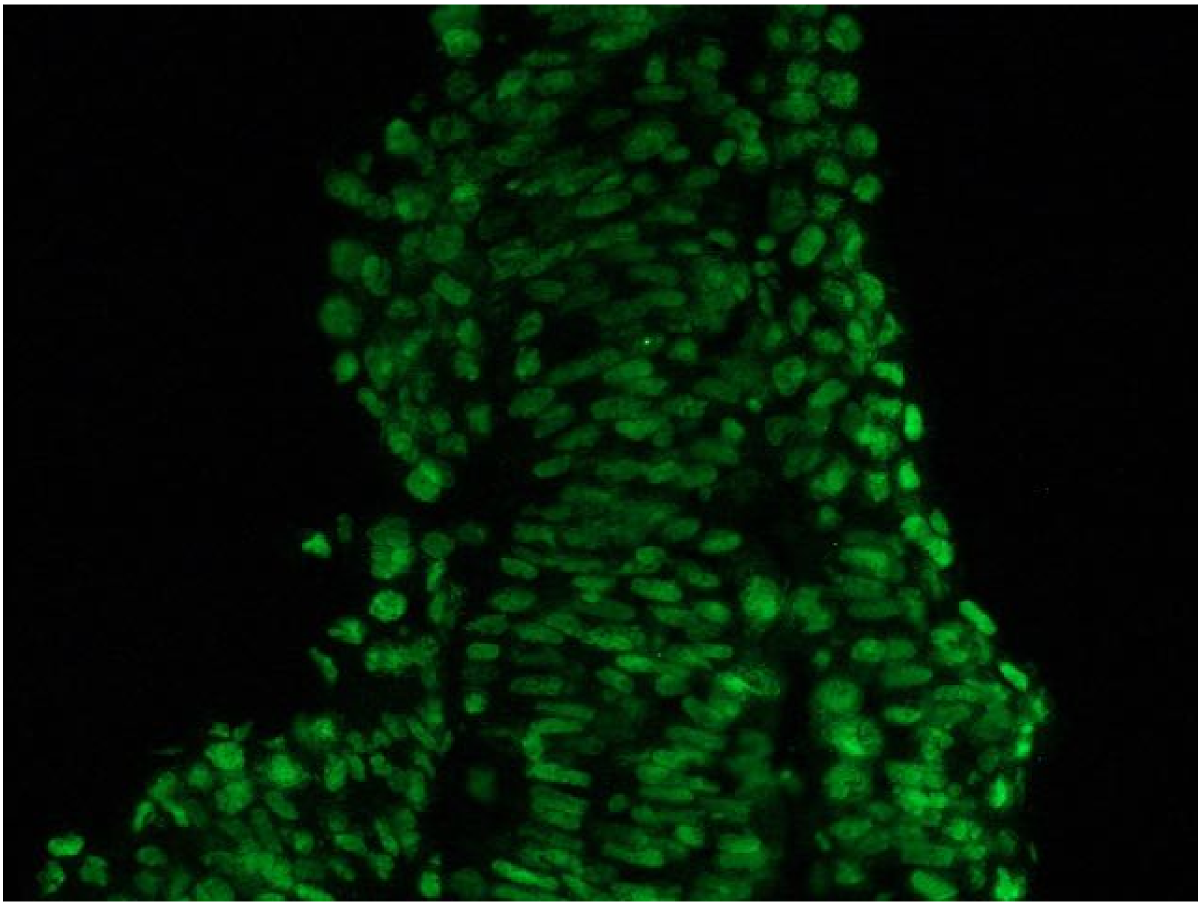

Raw image for Extended Data Fig. 5c Uninjected stage 10.5 animal cap stained for DAPI

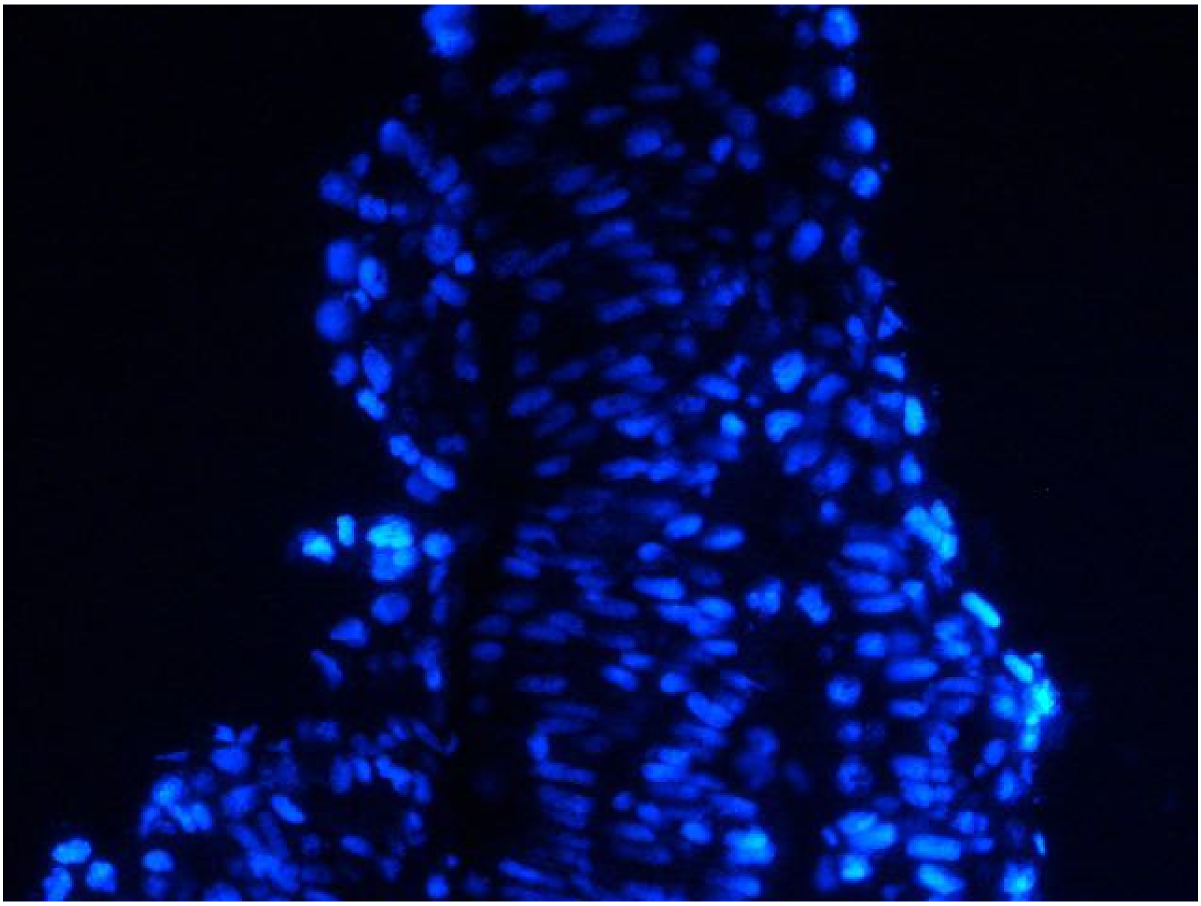

Raw image for Extended Data Fig. 5c stage 10.5 equivalent NANOG KD animal cap stained for POLII

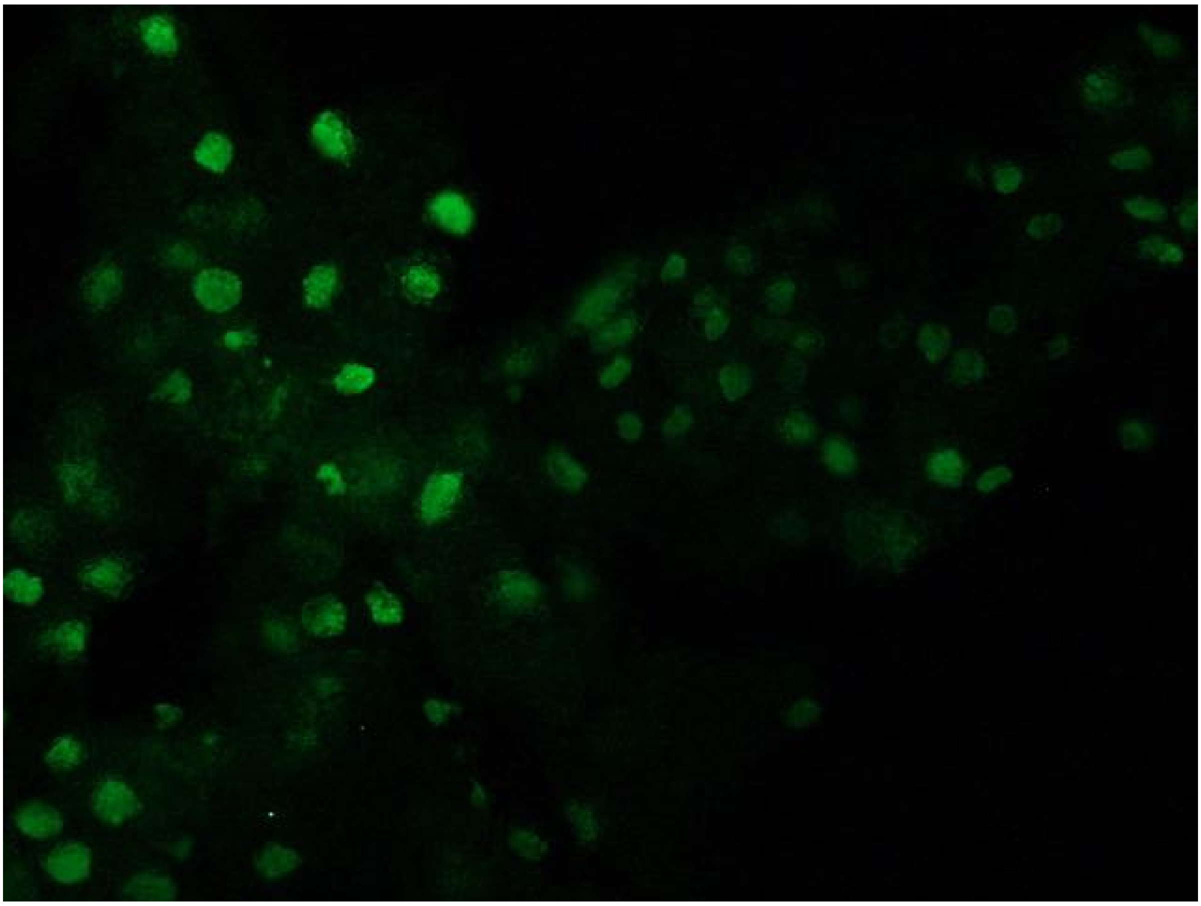

Raw image for Extended Data Fig. 5c stage 10.5 equivalent NANOG KD animal cap stained for DAPI

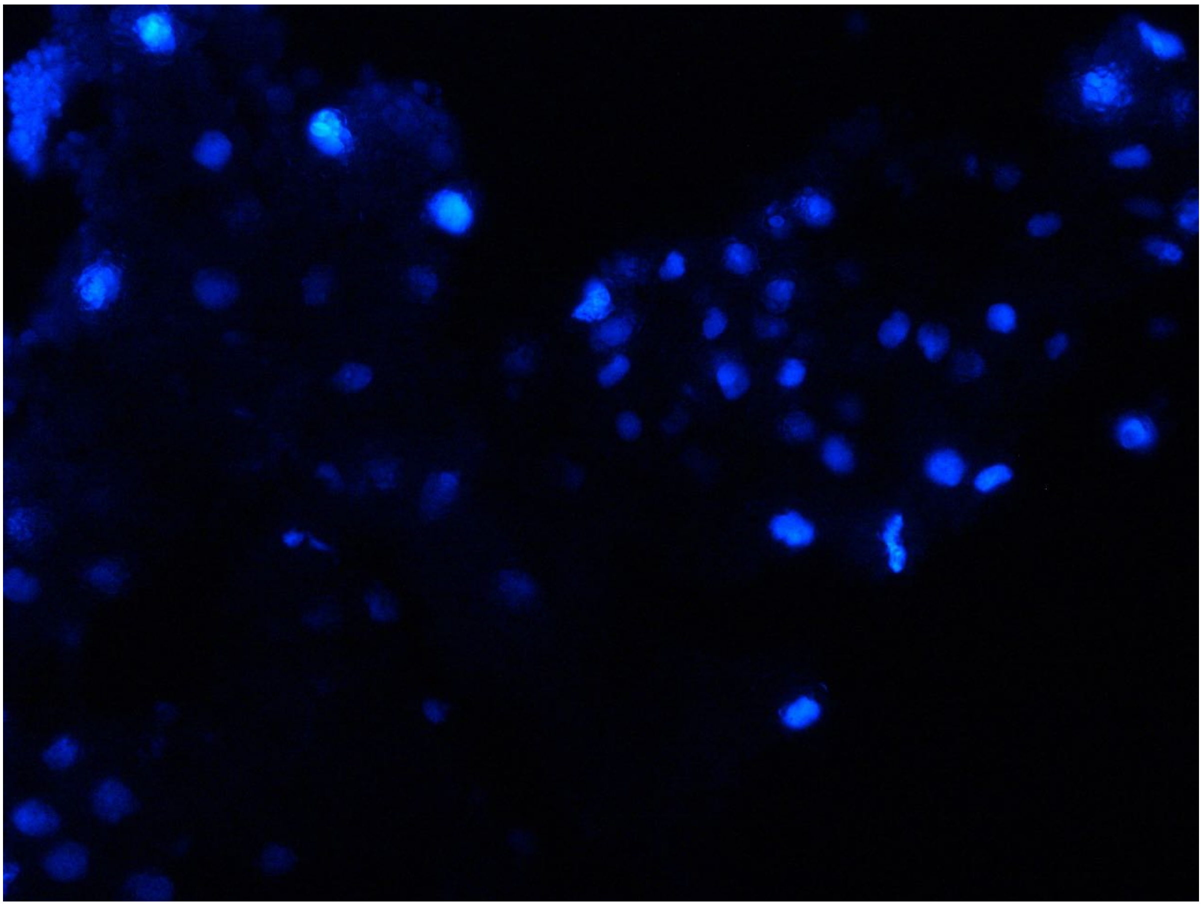

Raw image for Extended Data Fig. 5c SB431542 treated stage 10.5 animal cap stained for POLII

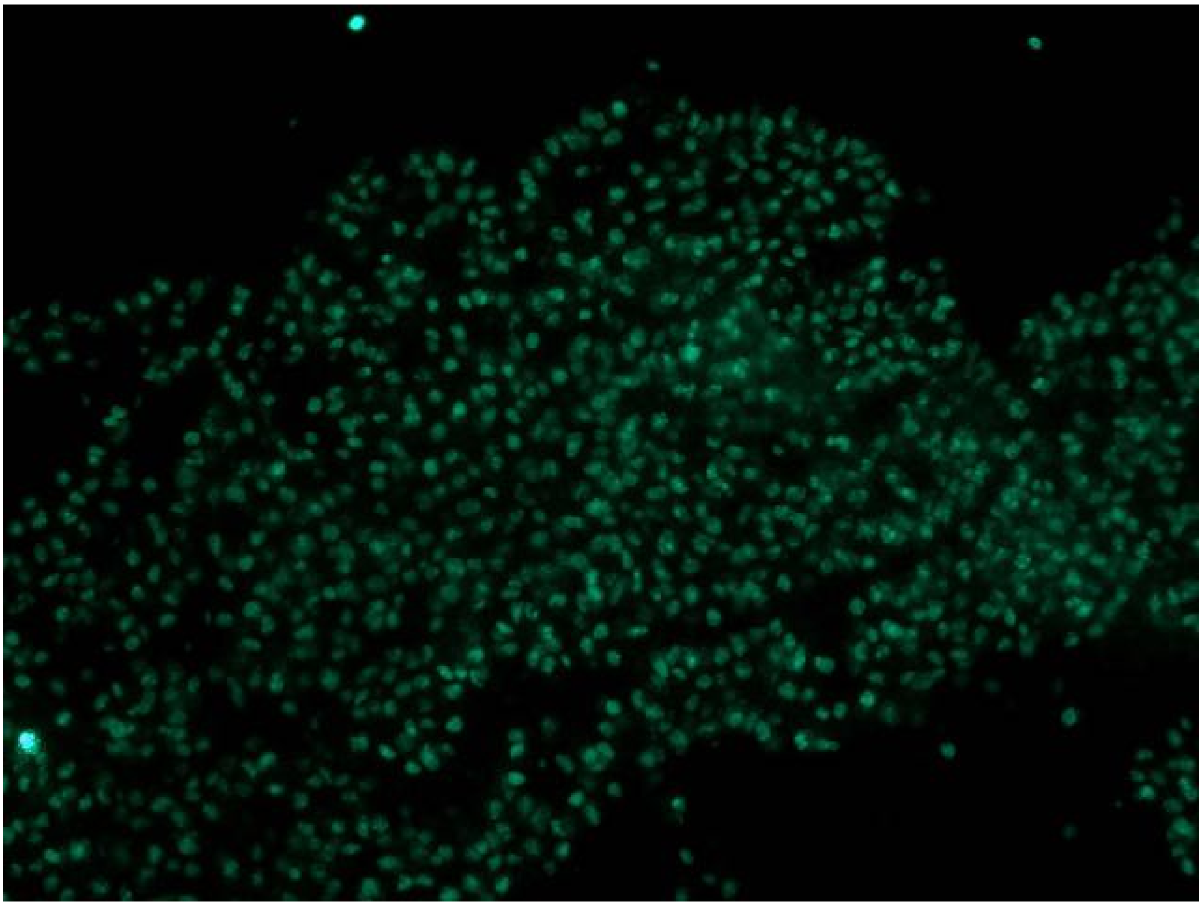

Raw image for Extended Data Fig. 5c SB431542 treated stage 10.5 animal cap stained for DAPI

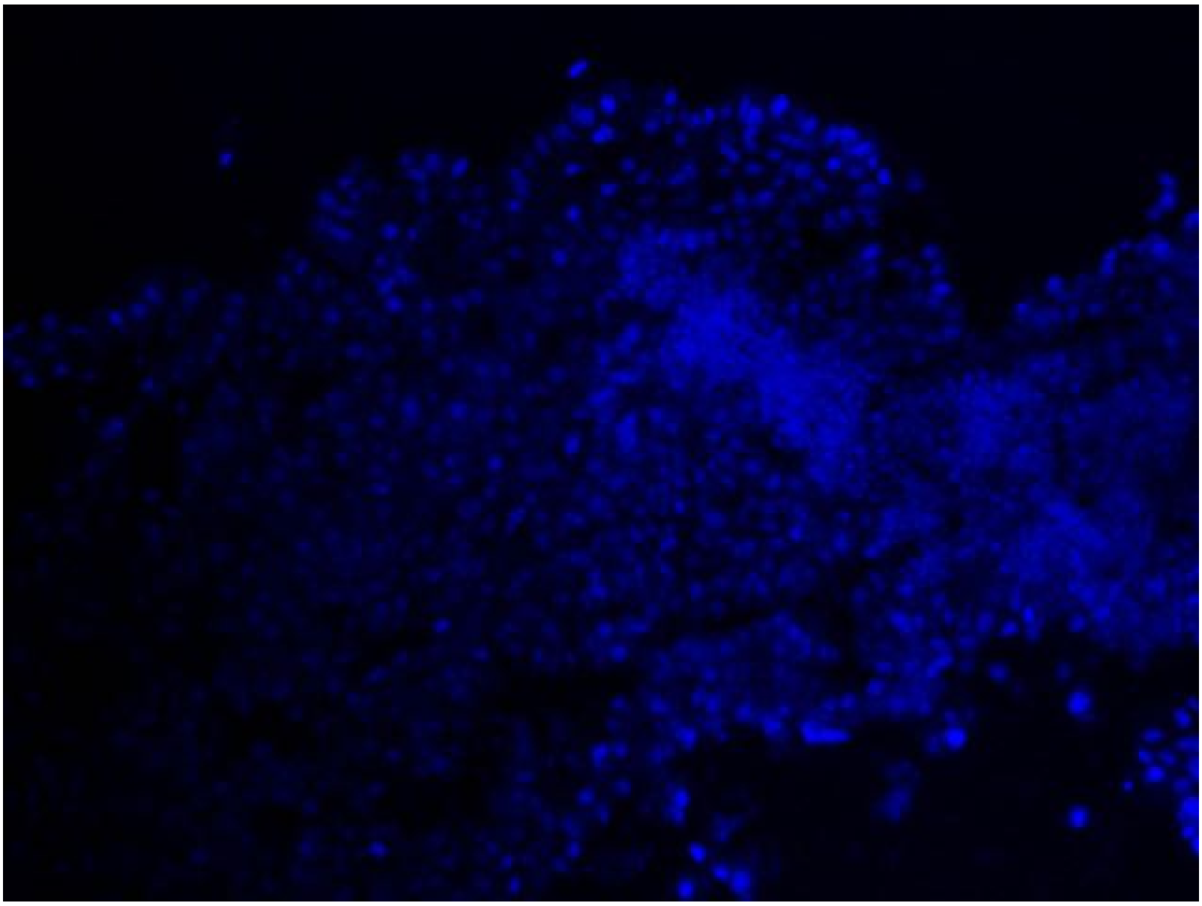

Raw image for Extended Data Fig. 5c DPY30 KD stage 10.5 animal cap stained for POLII

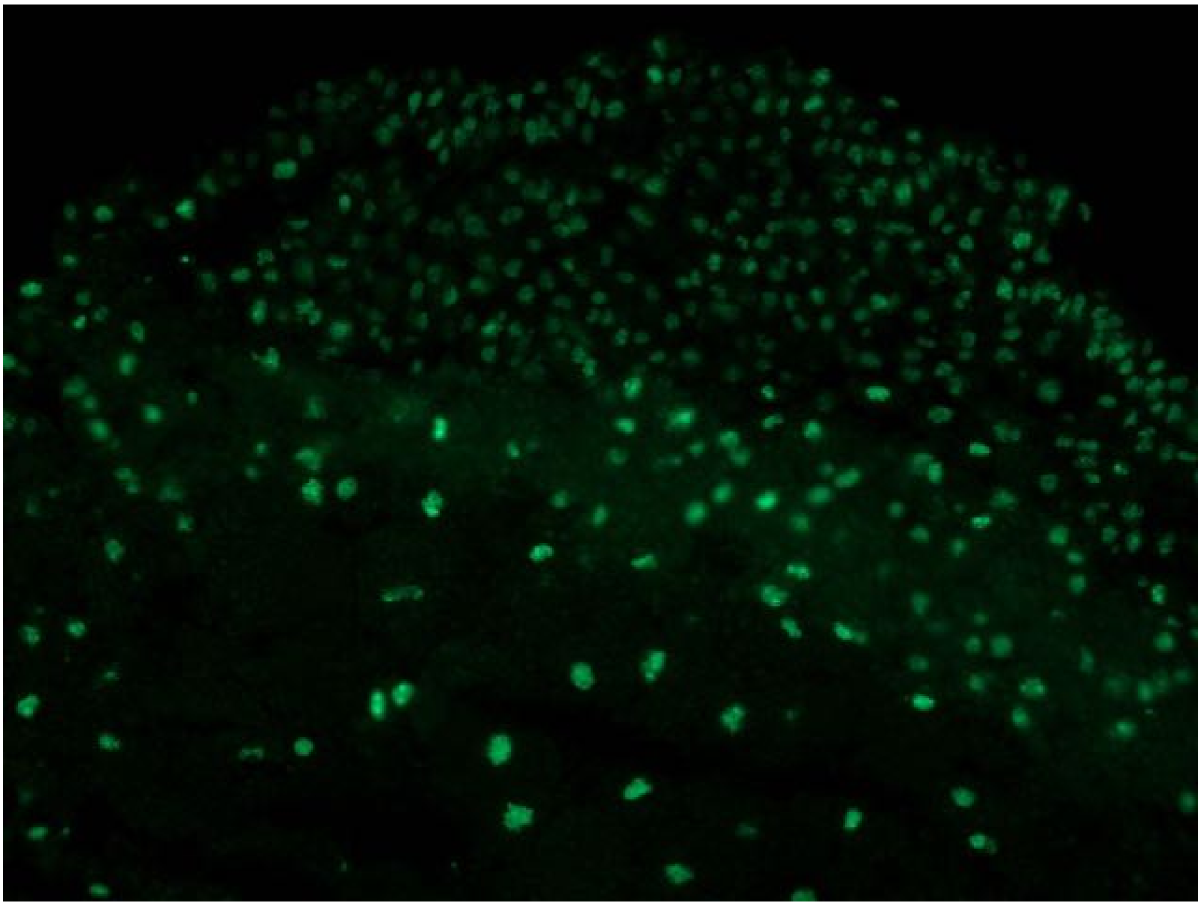

Raw image for Extended Data Fig. 5c DPY30 KD stage 10.5 animal cap stained for DAPI

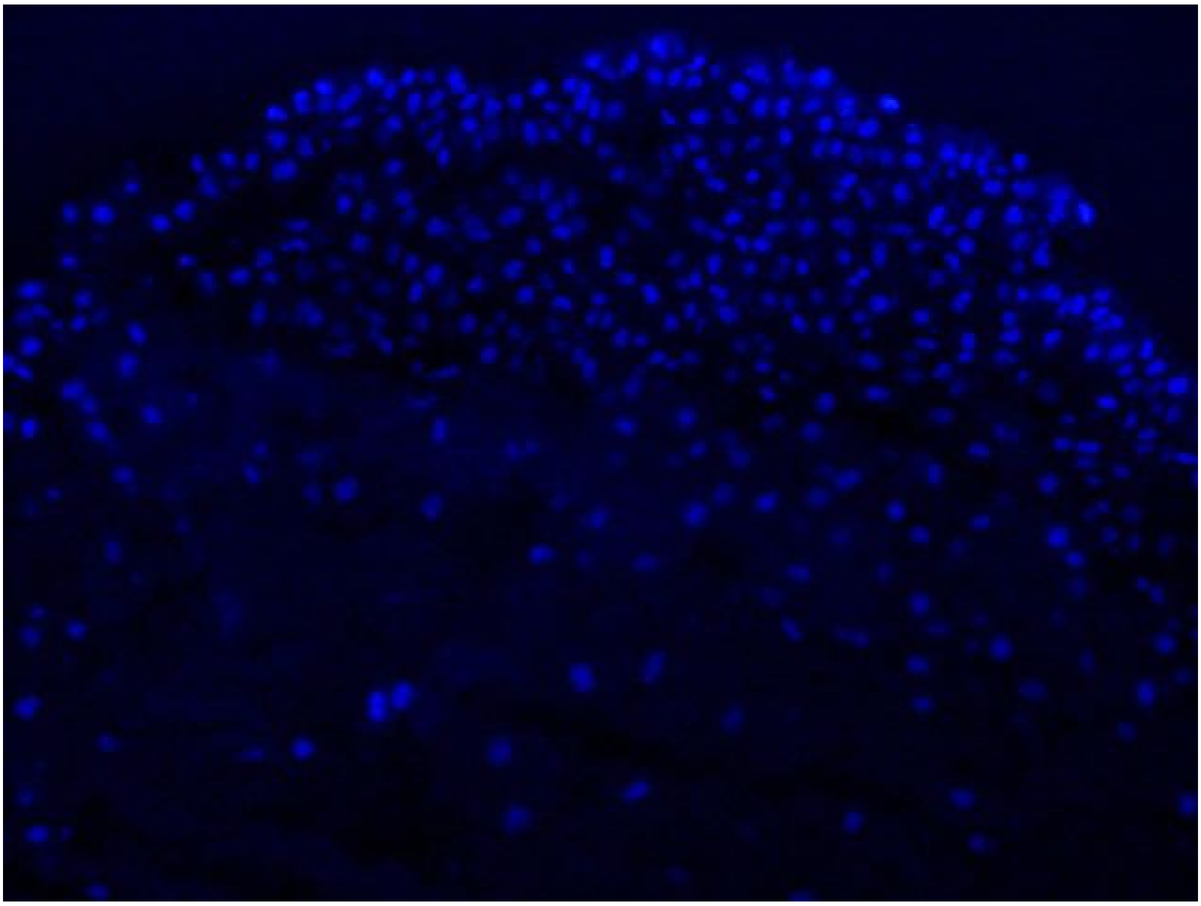

Raw image for Extended Data Fig. 5d stage 22 vegetal explants

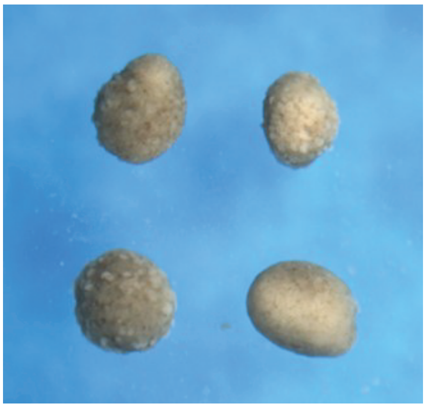

Raw image for Extended Data Fig. 5d stage 22 animal hemisphere explants

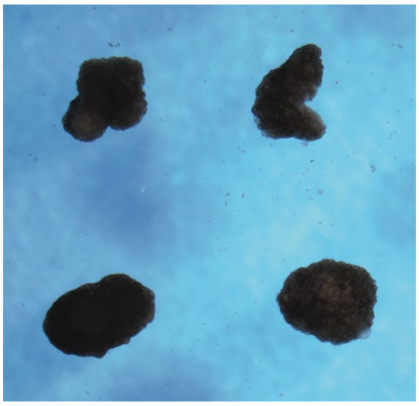

Raw image for Extended Data Fig. 9a DPY30 KD + HDPY30 stage 40 embryo brightfeild image

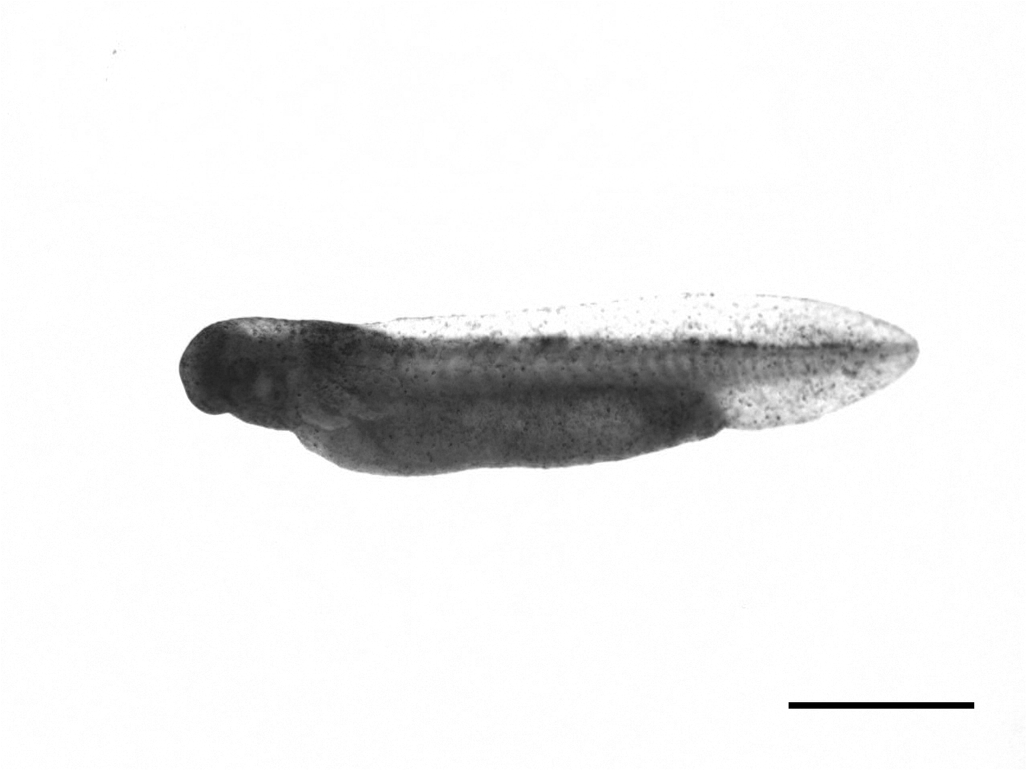

Raw image for Extended Data Fig. 9a equivalent stage 40 DPY30 KD embryo brightfeild image

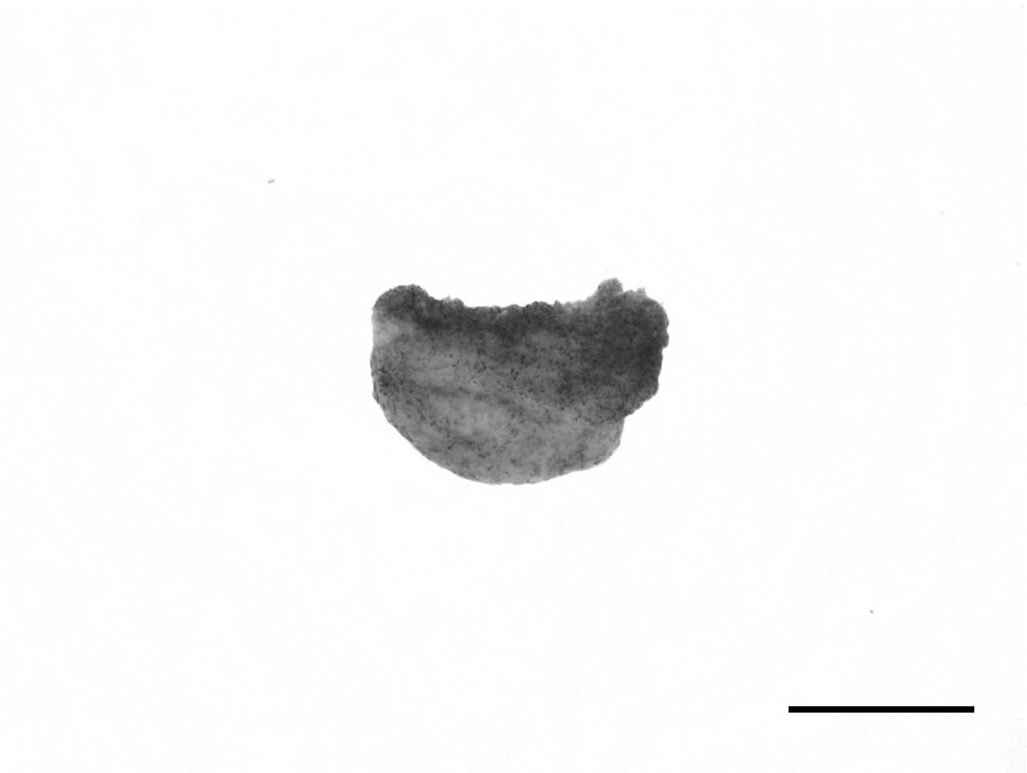

Raw image for Extended Data Fig. 9a DPY30 KD + HDPY30 stage 40 embryo HREM 3D reconstruction

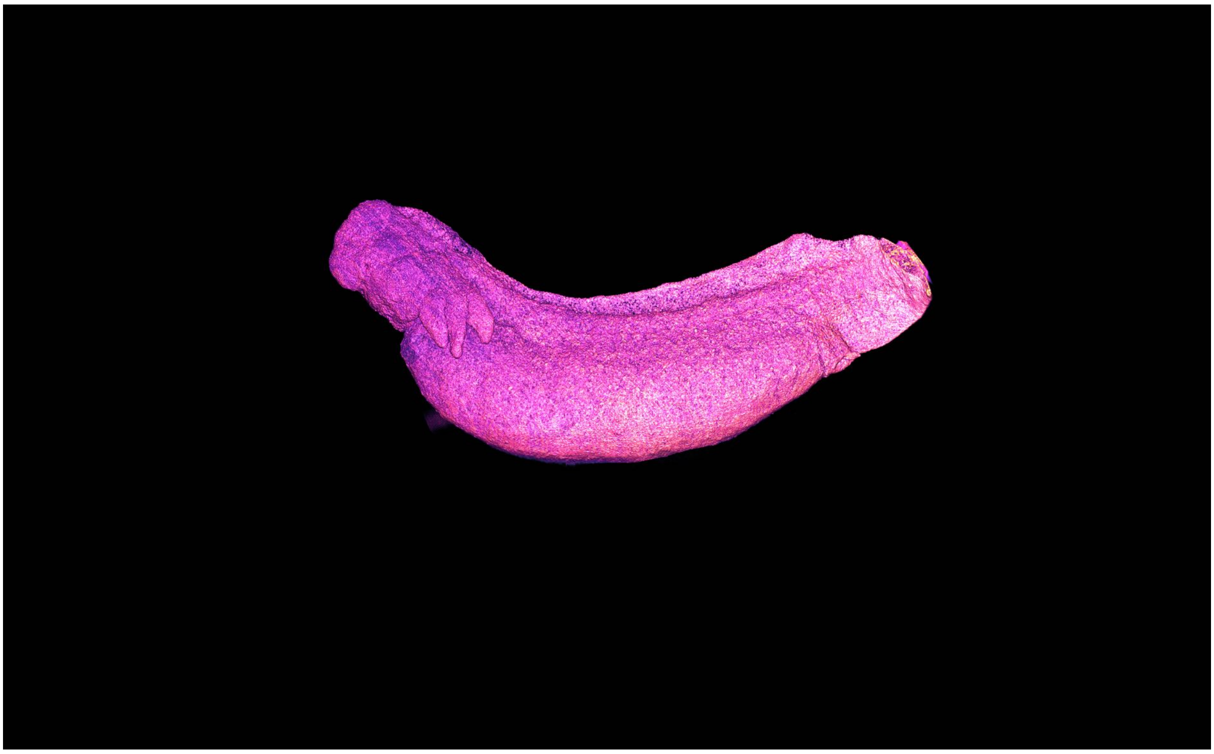

Raw image for Extended Data Fig. 9a equivalent stage 40 DPY30 KD HREM 3D reconstruction

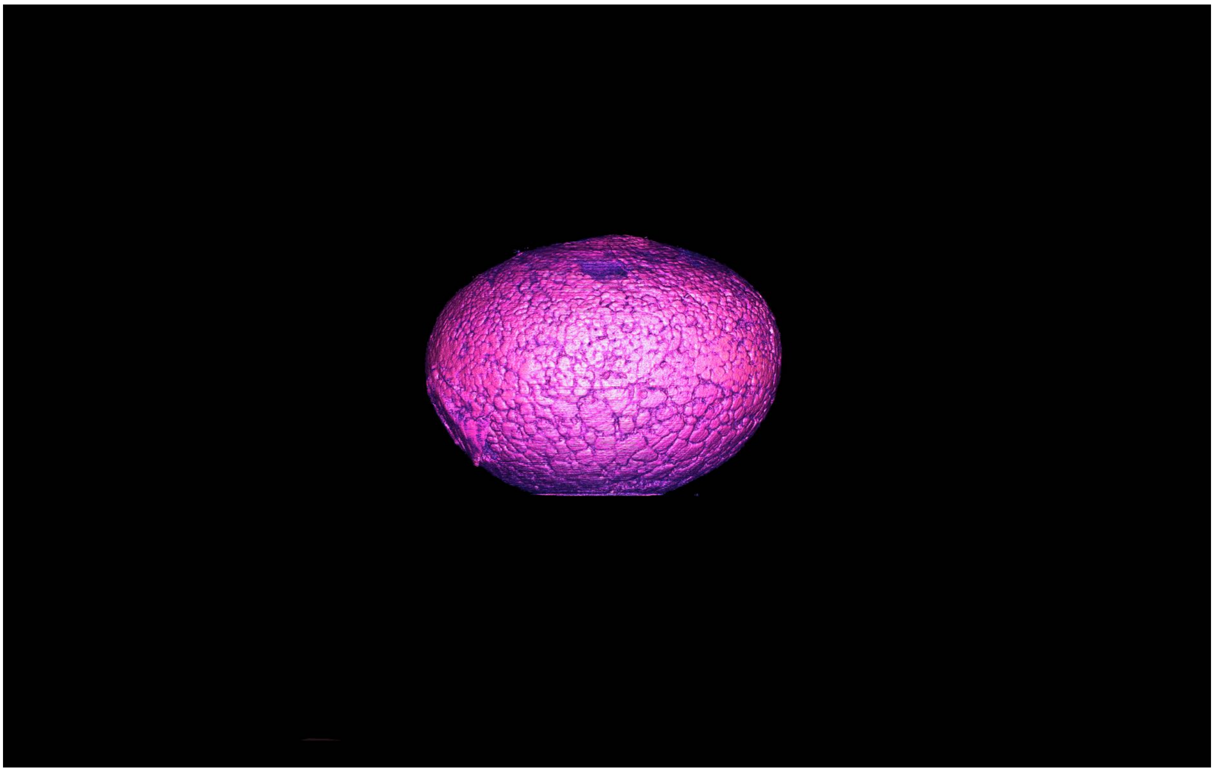

Raw image for Extended Data Fig. 9a DPY30 KD + HDPY30 stage 40 embryo HREM 3D reconstruction sagital section

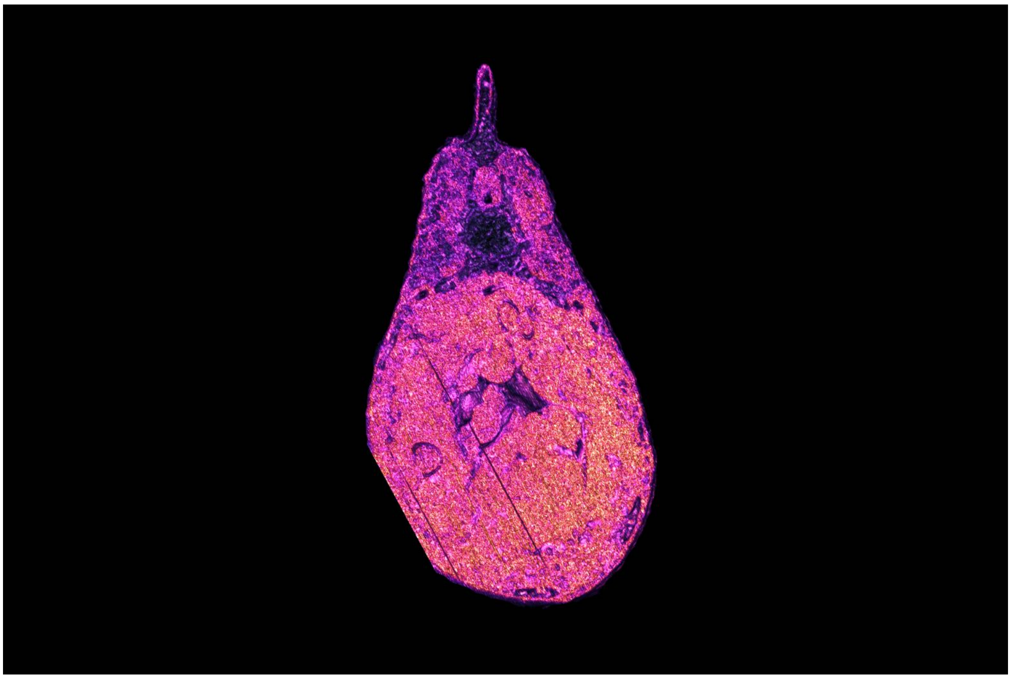

Raw image for Extended Data Fig. 9a equivalent stage 40 DPY30 KD HREM 3D reconstruction sagital section

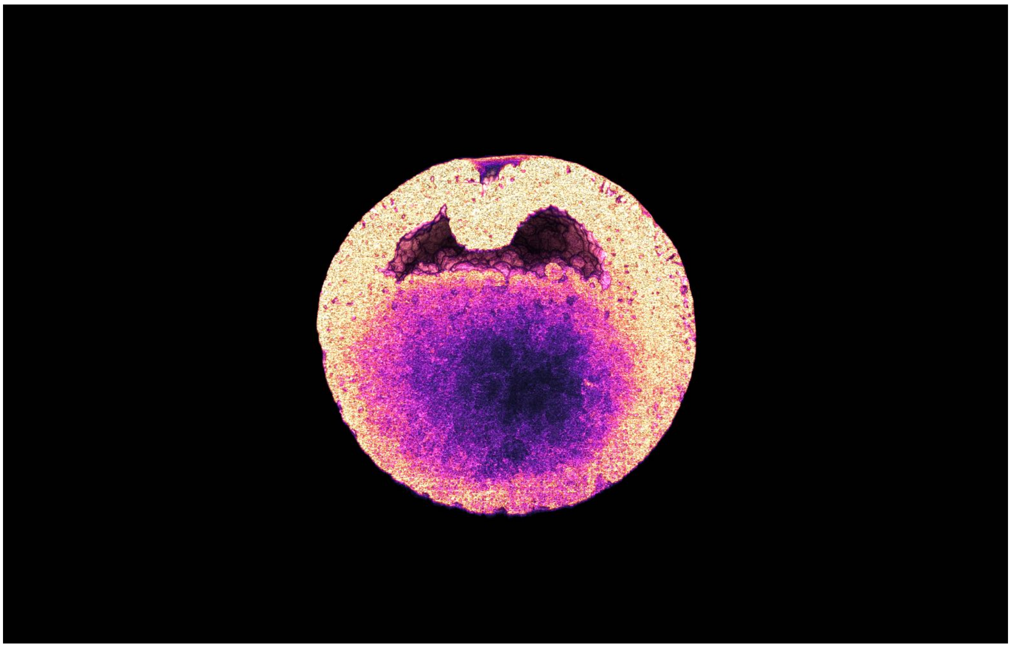

Raw image for Extended Data Fig. 11e Uninjected stage 10.5 animal cap stained for H3K4me3

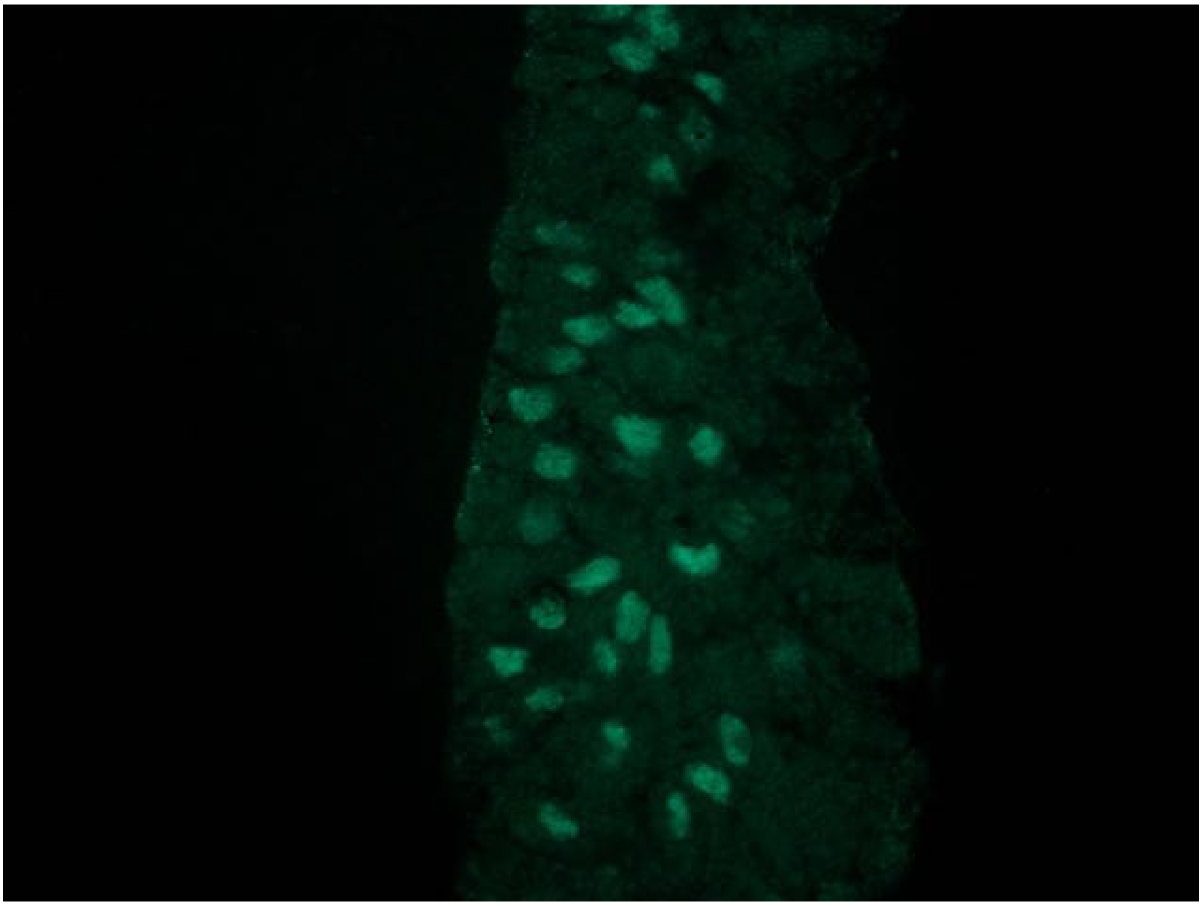

Raw image for Extended Data Fig. 11e Uninjected stage 10.5 animal cap stained for DAPI

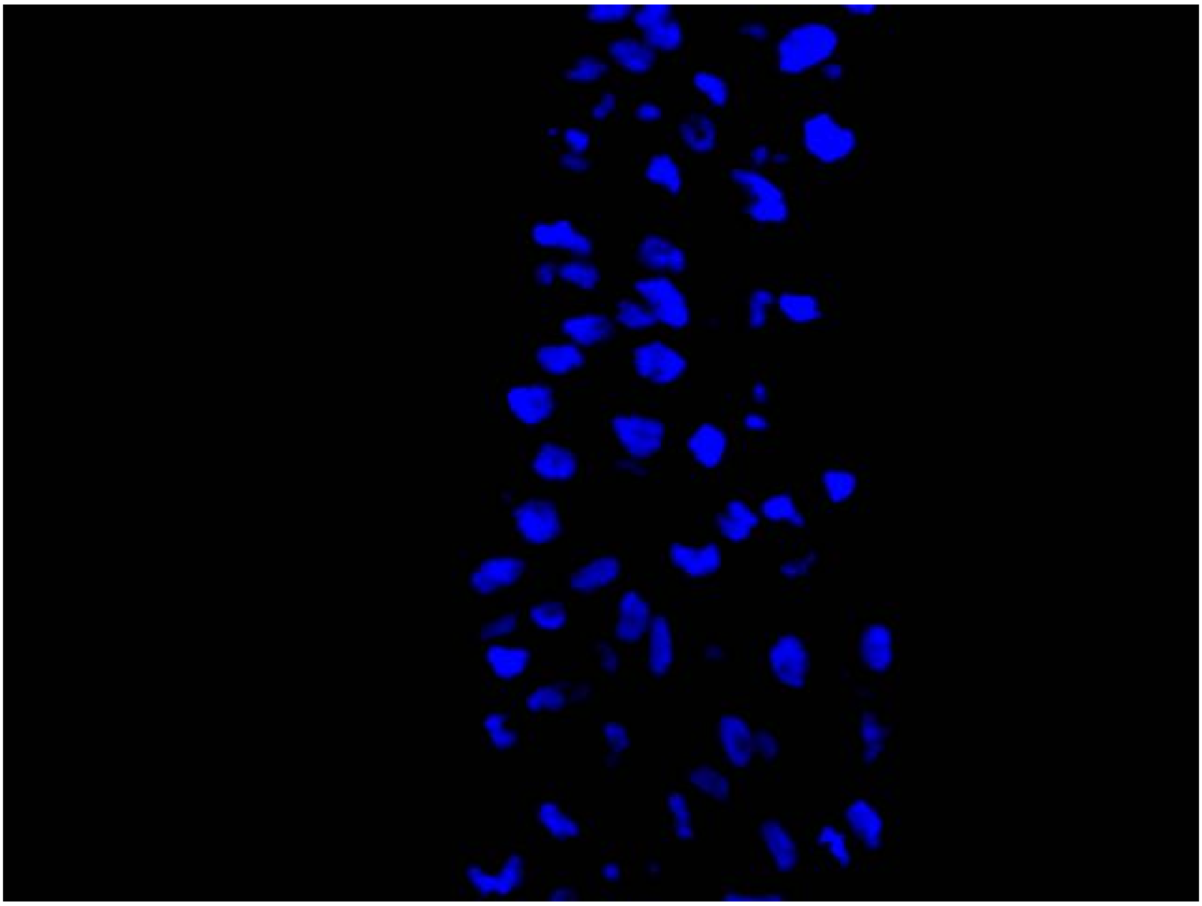

Raw image for Extended Data Fig. 11e DPY30 KD stage 10.5 animal cap stained for H3K4me3

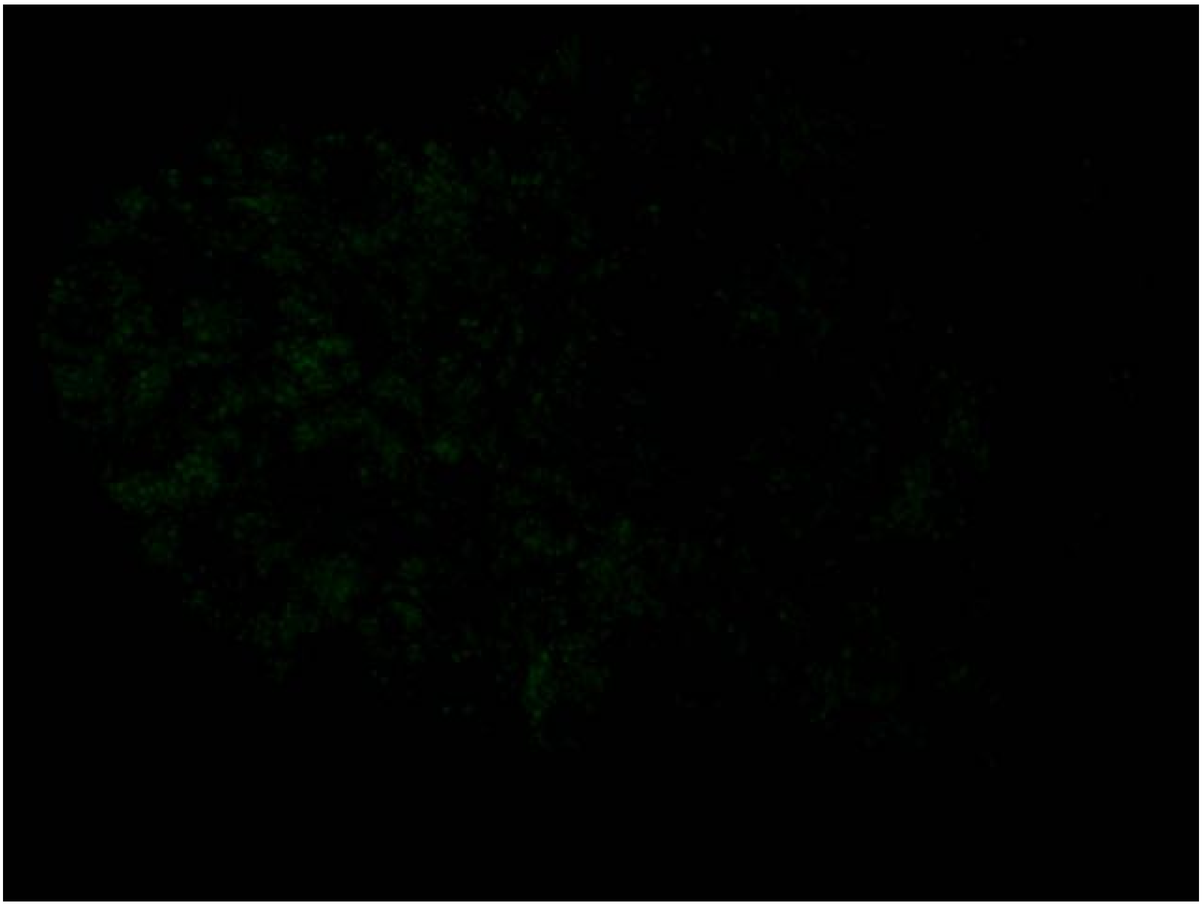

Raw image for Extended Data Fig. 11e DPY30 KD stage 10.5 animal cap stained for DAPI

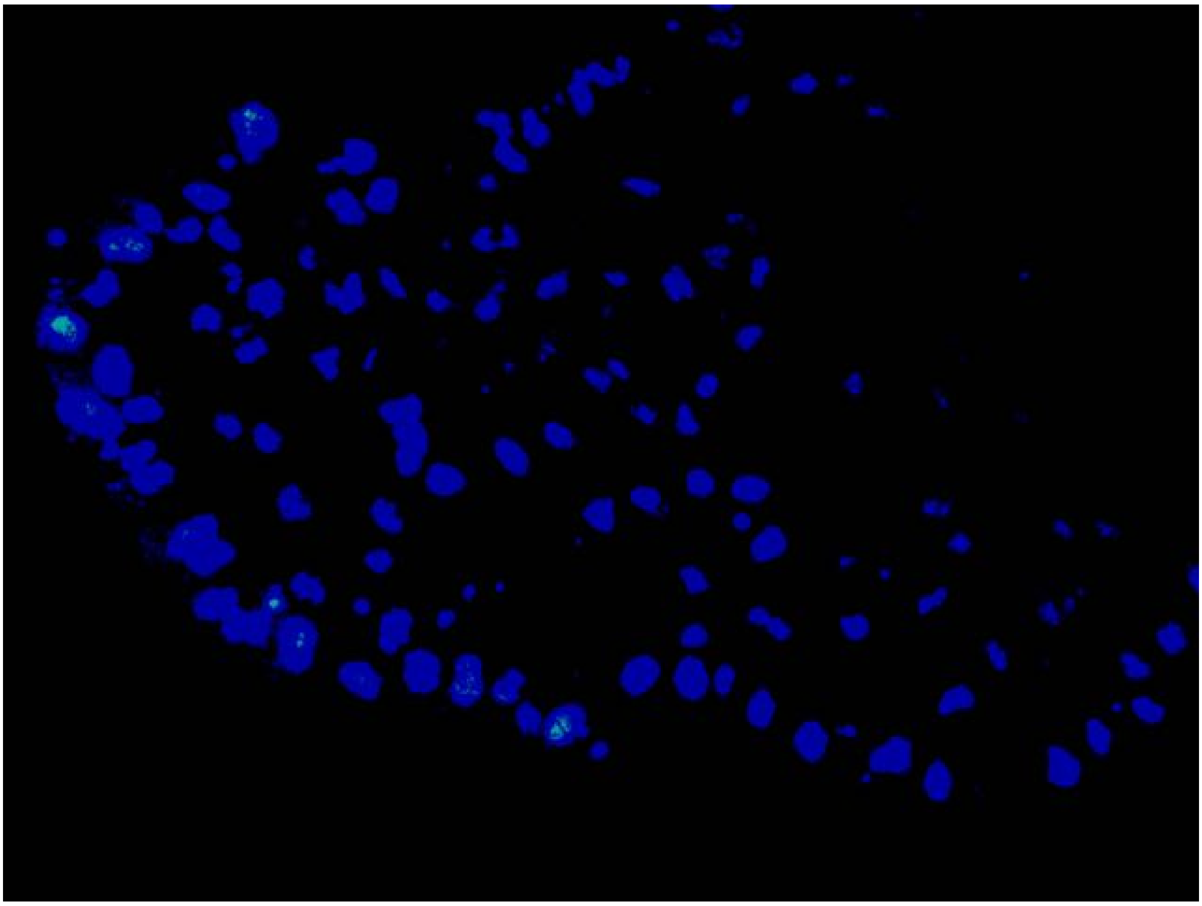

Raw image for Extended Data Fig. 11e DPY30 KD (2) stage 10.5 animal cap stained for H3K4me3

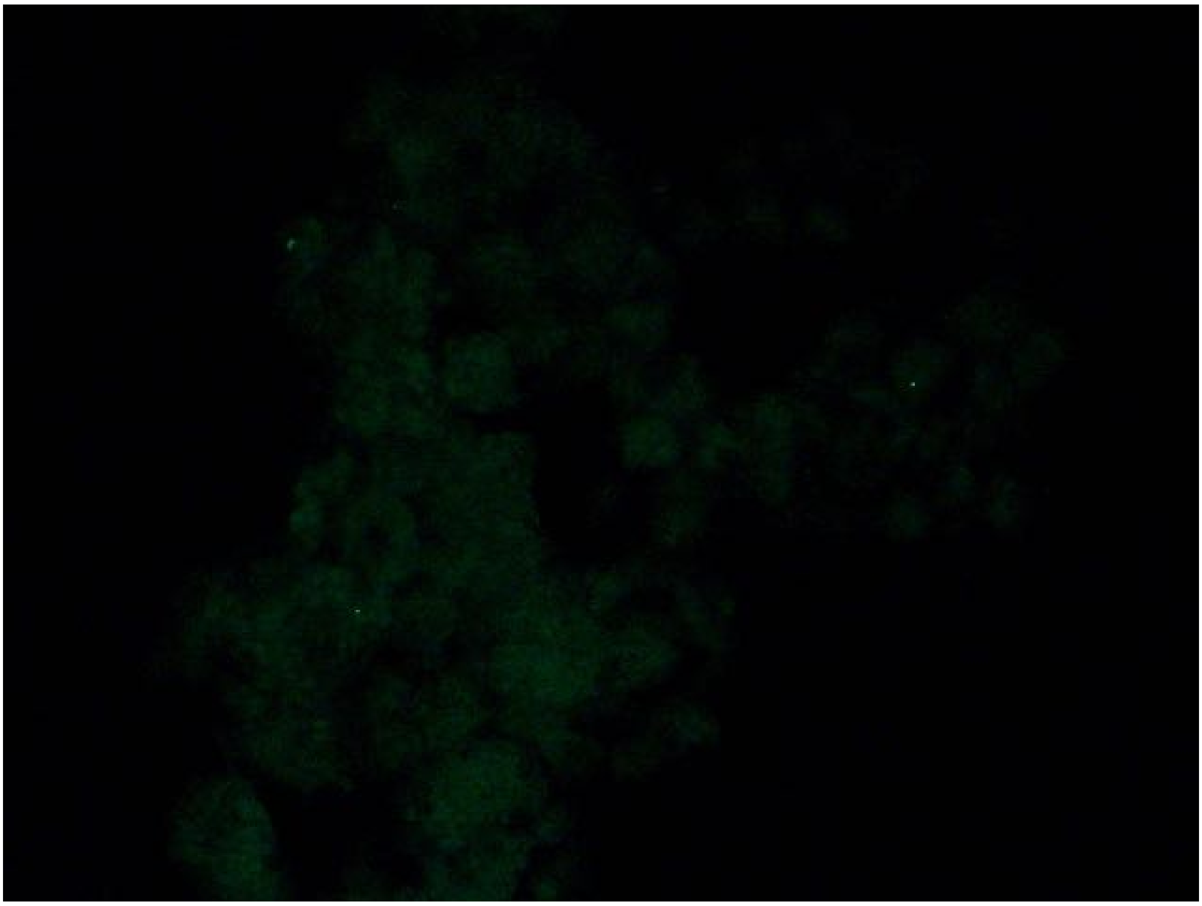

Raw image for Extended Data Fig. 11e DPY30 KD (2) stage 10.5 animal cap stained for DAPI

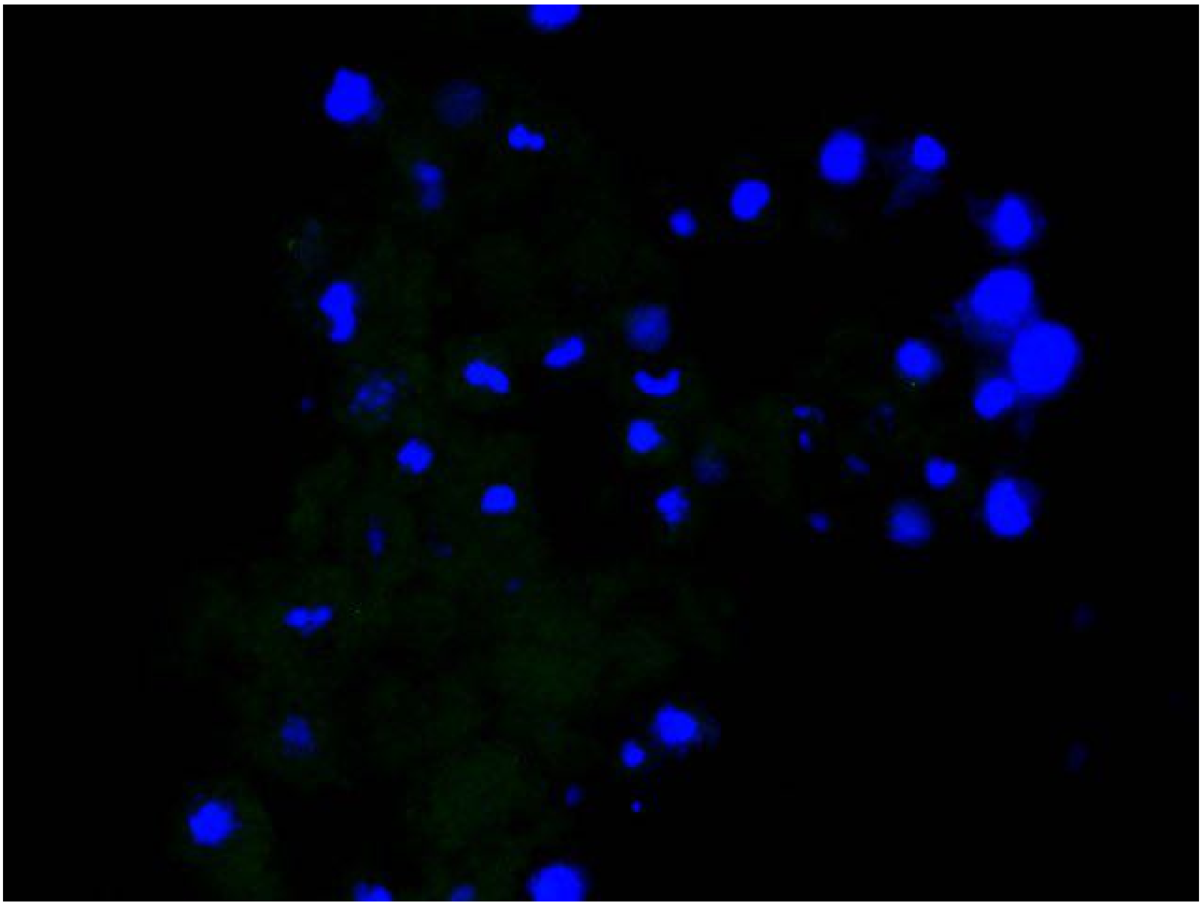

Raw image for Extended Data Fig. 11e Uninjected stage 10.5 animal cap stained for H3K27ac

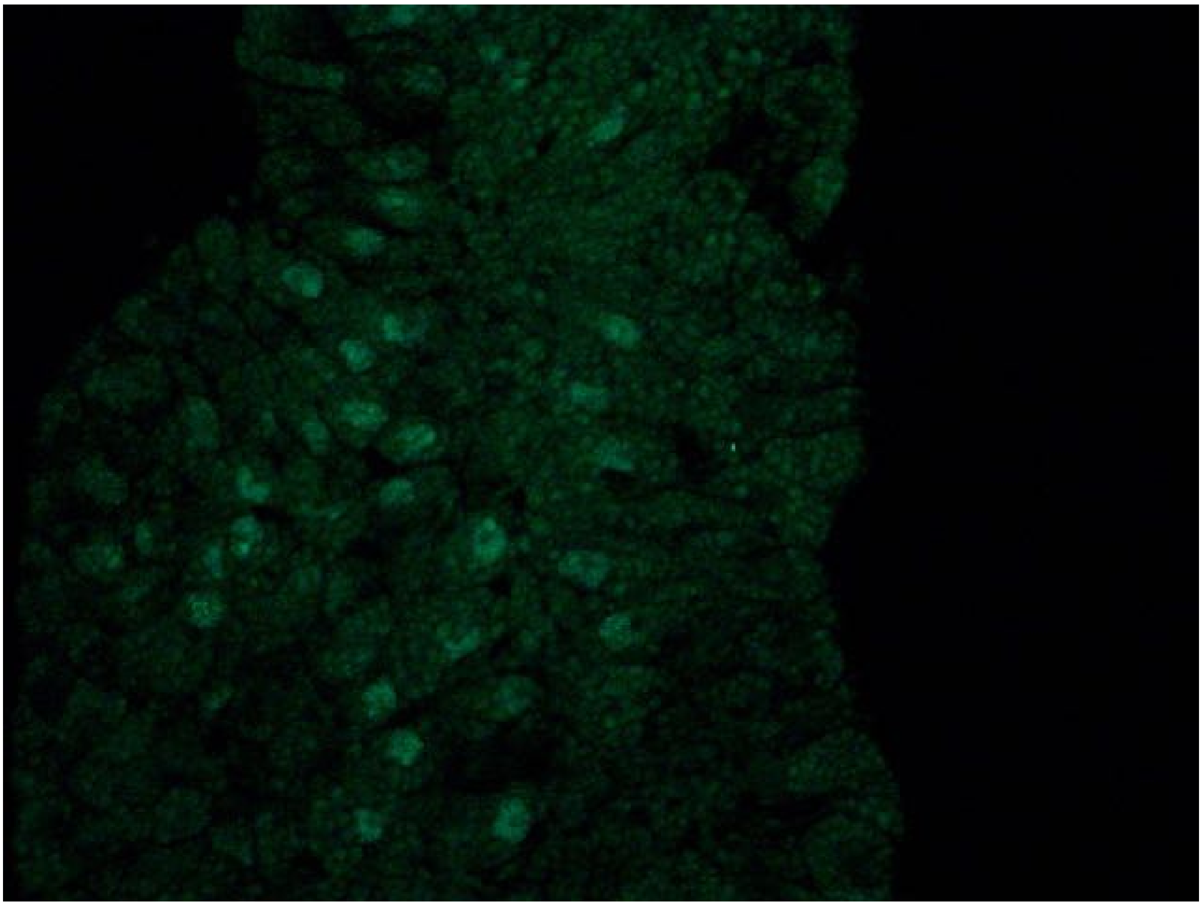

Raw image for Extended Data Fig. 11e Uninjected stage 10.5 animal cap stained for DAPI

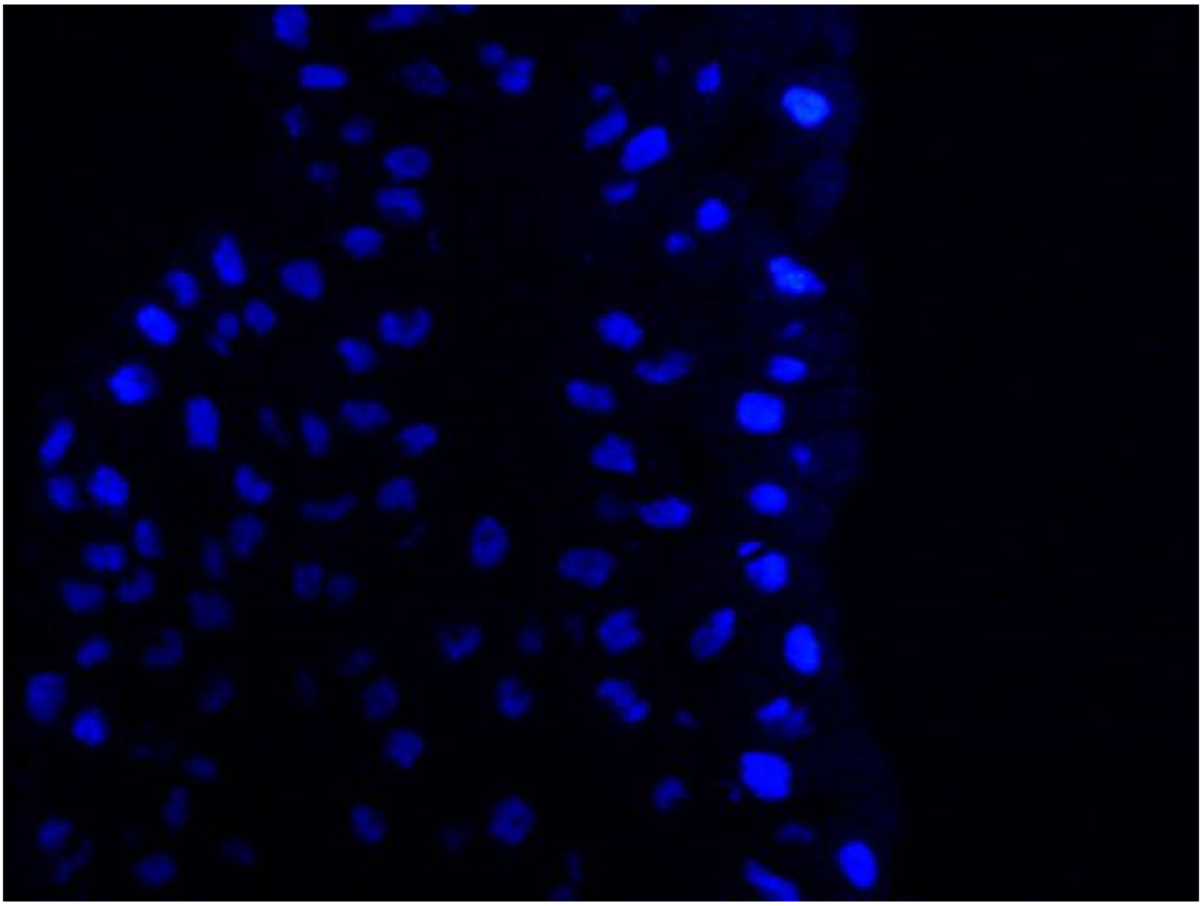

Raw image for Extended Data Fig. 11e DPY30 KD stage 10.5 animal cap stained for H3K27ac

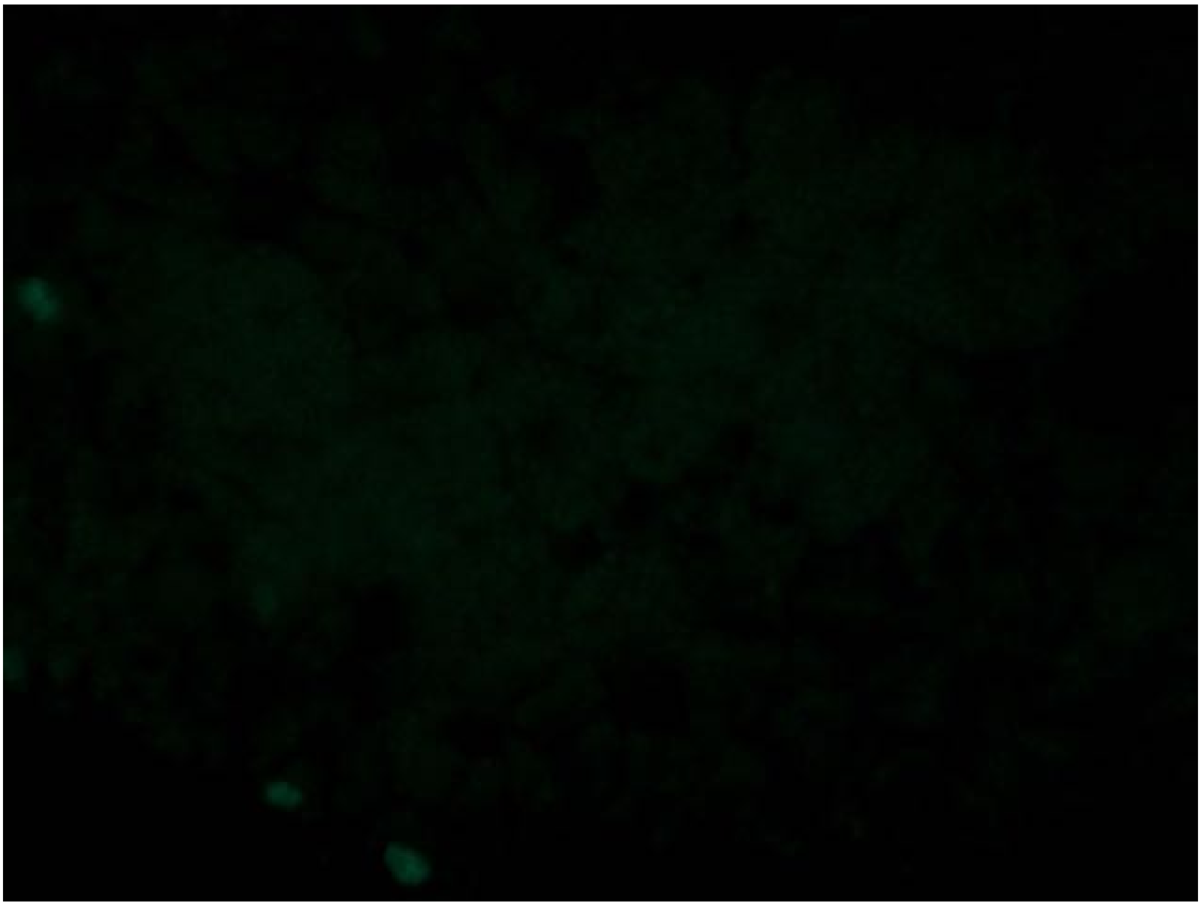

Raw image for Extended Data Fig. 11e DPY30 KD stage 10.5 animal cap stained for DAPI

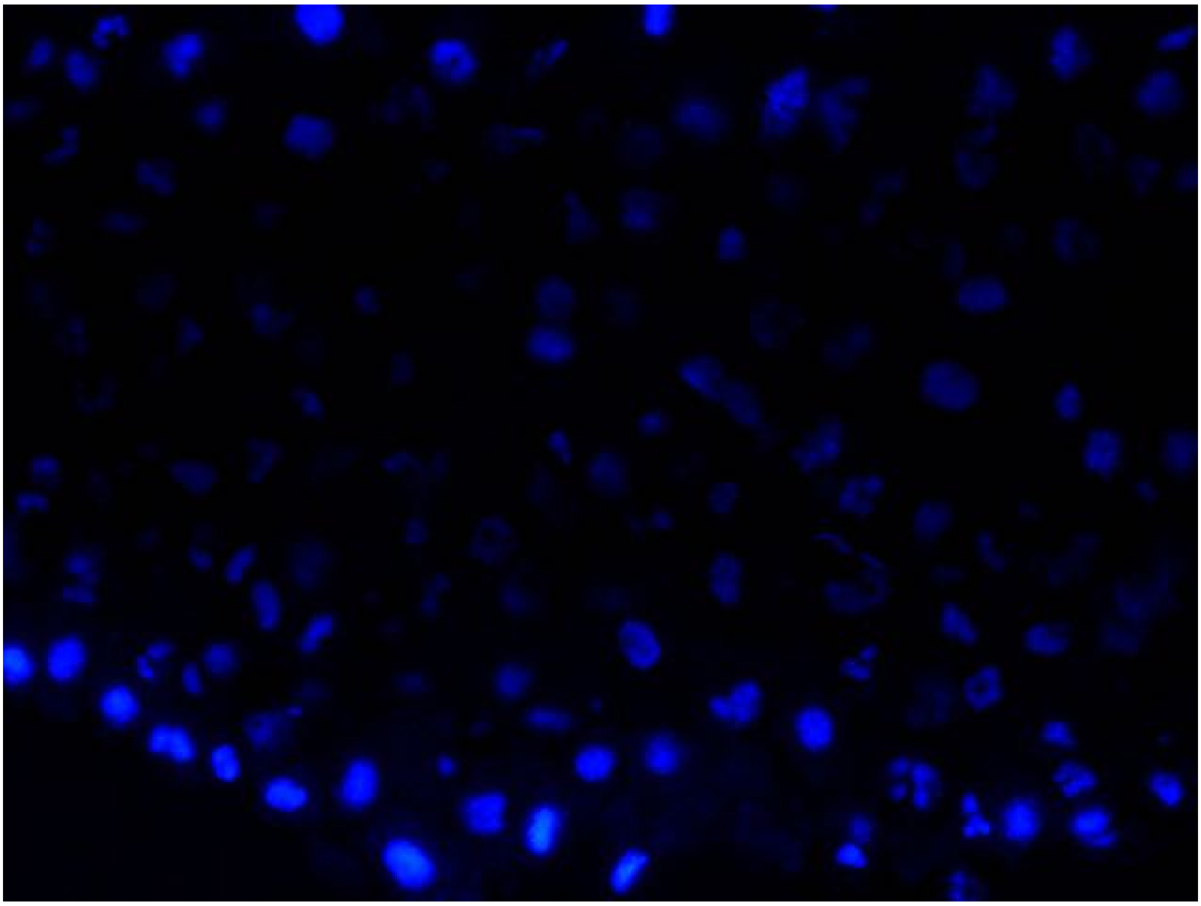

Raw image for Extended Data Fig. 11e DPY30 KD (2) stage 10.5 animal cap stained for H3K27ac

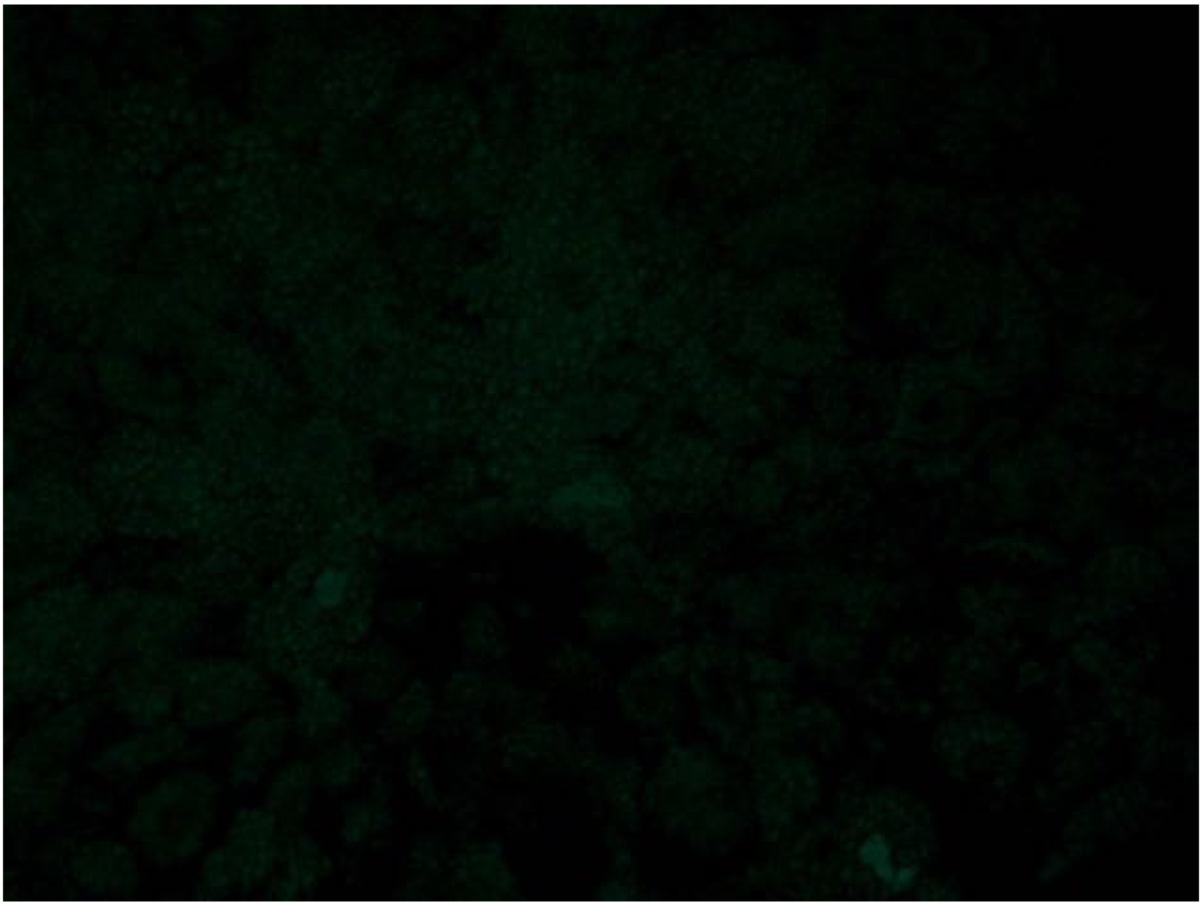

Raw image for Extended Data Fig. 11e DPY30 KD (2) stage 10.5 animal cap stained for DAPI

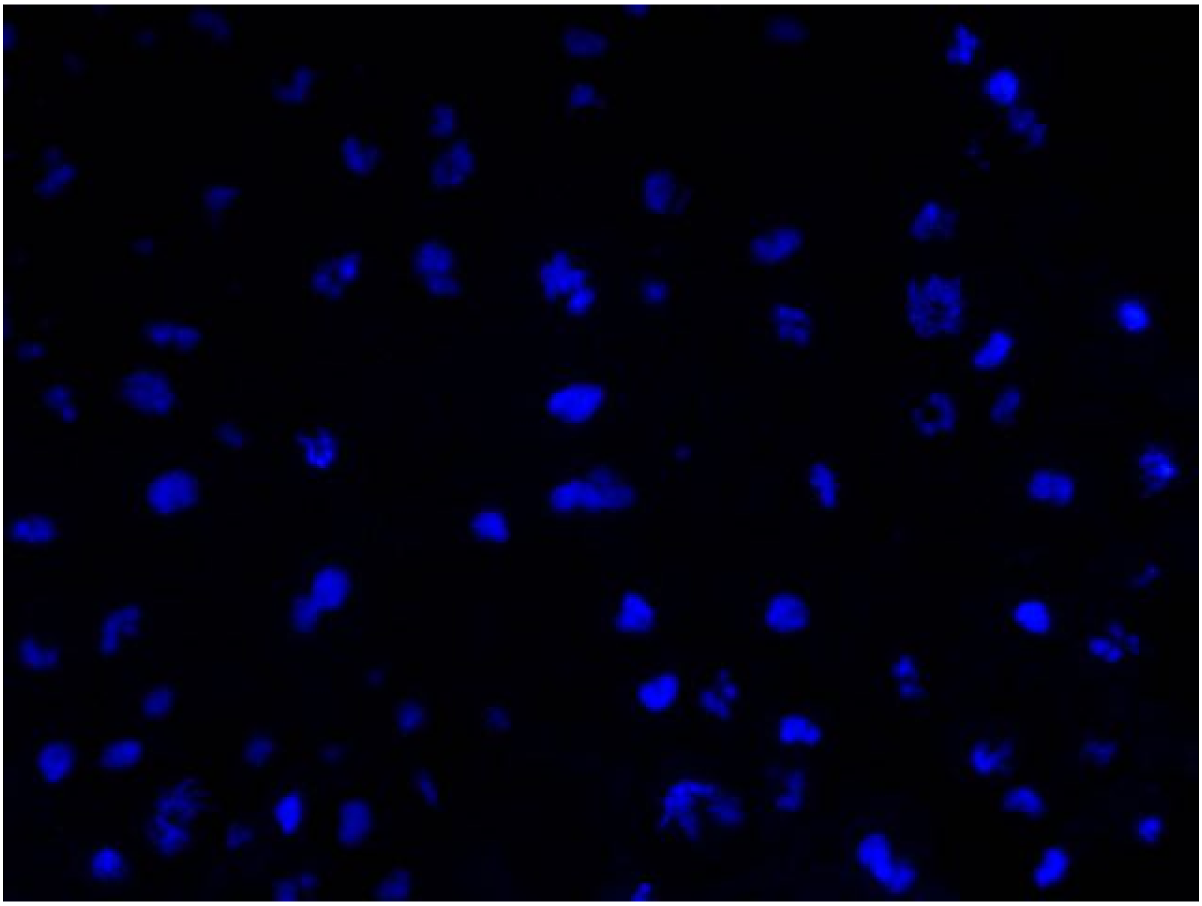

Raw image for Extended Data Fig. 11e DPY30 KD (1) stage 10.5 animal cap stained for H3K27me3

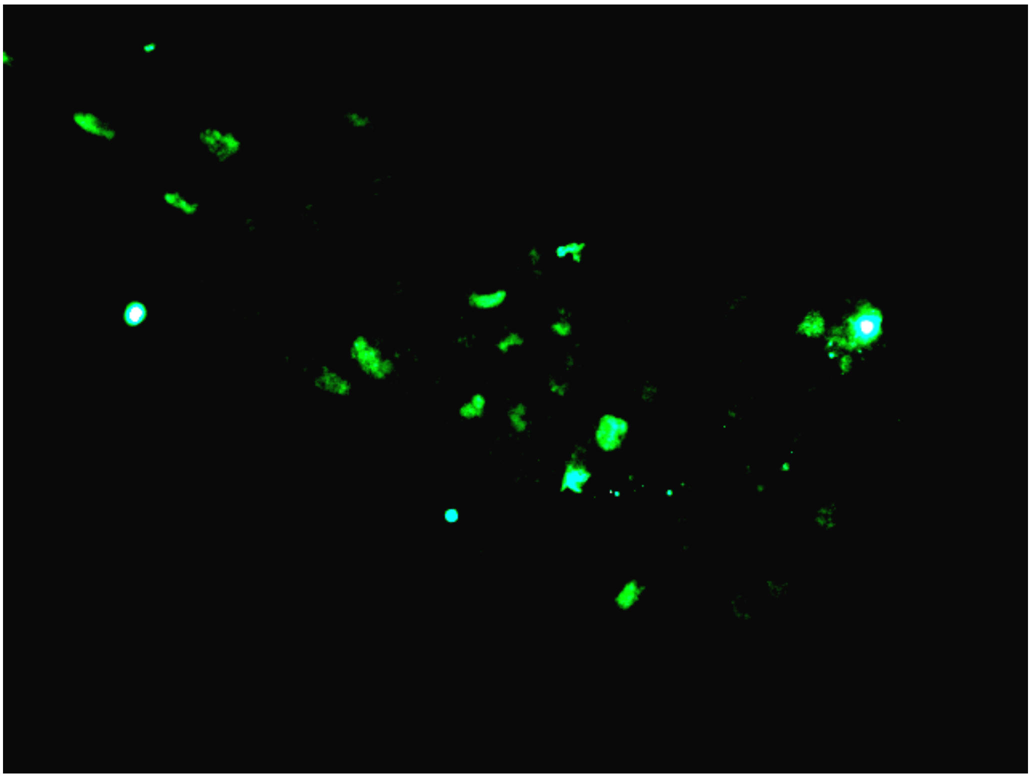

Raw image for Extended Data Fig. 11e DPY30 KD (1) stage 10.5 animal cap stained for DAPI

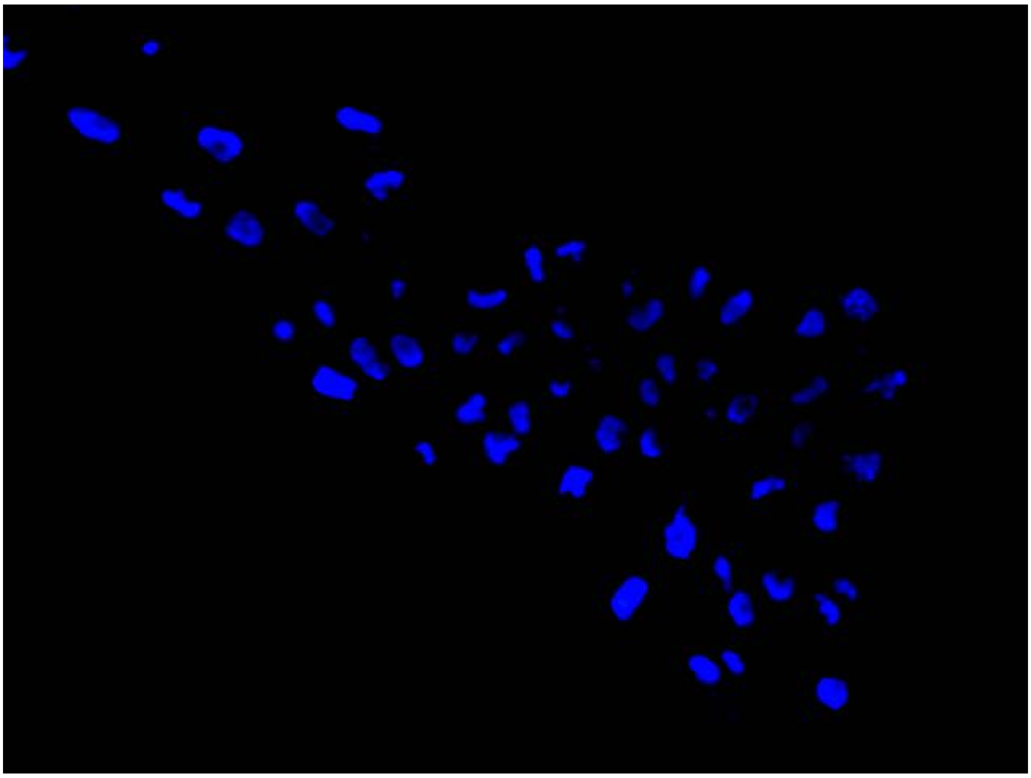

Raw image for Extended Data Fig. 11e DPY30 KD (2) stage 10.5 animal cap stained for H3K27me3

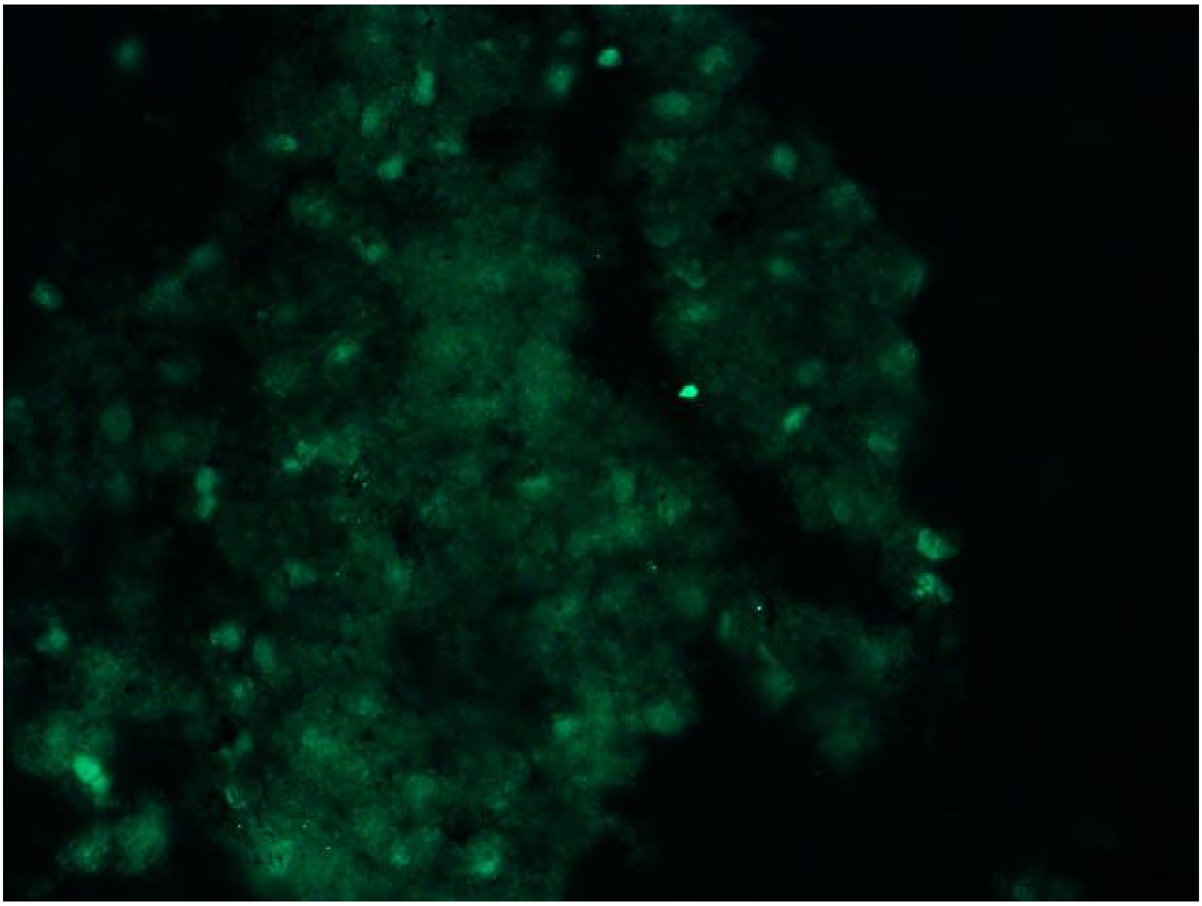

Raw image for Extended Data Fig. 11e DPY30 KD (2) stage 10.5 animal cap stained for DAPI

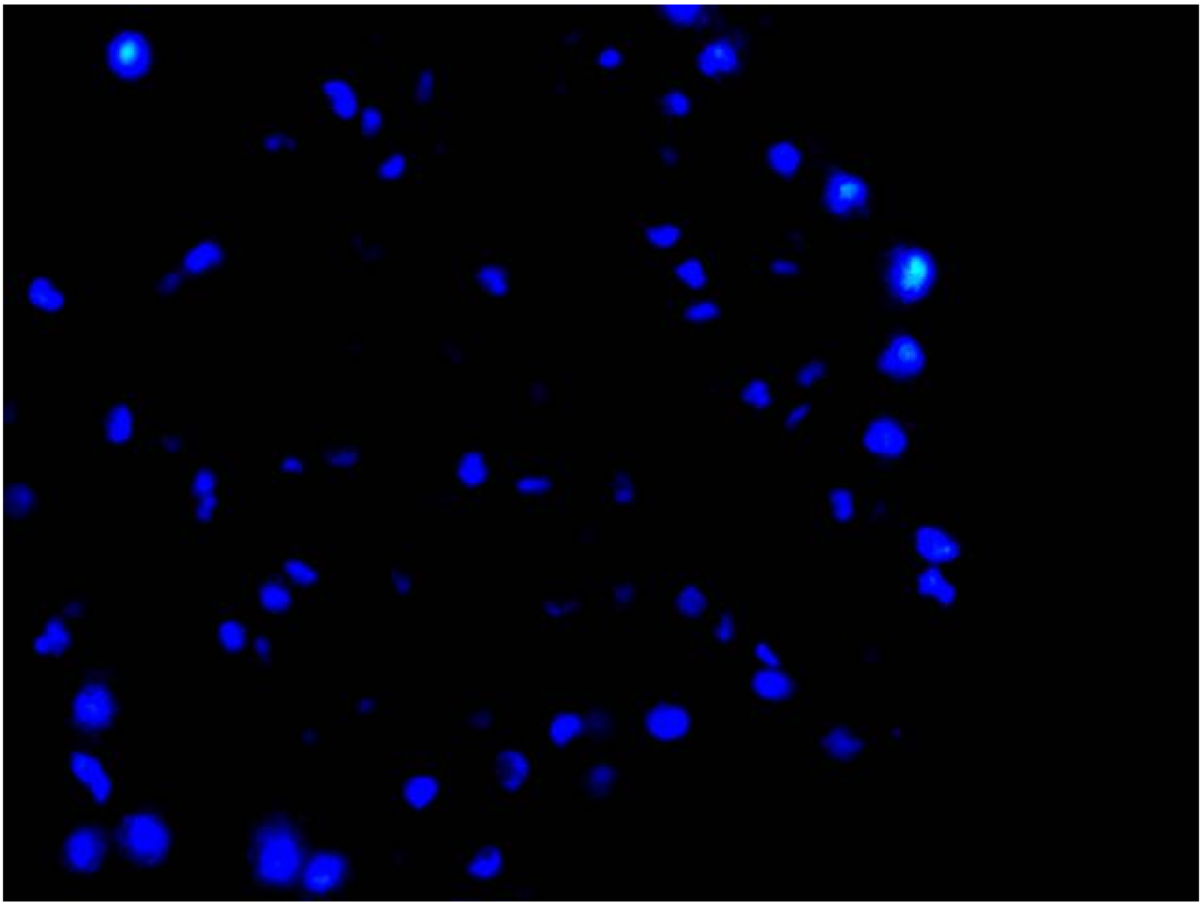

Raw image for Extended Data Fig. 13a stage 22 uninjected animal caps

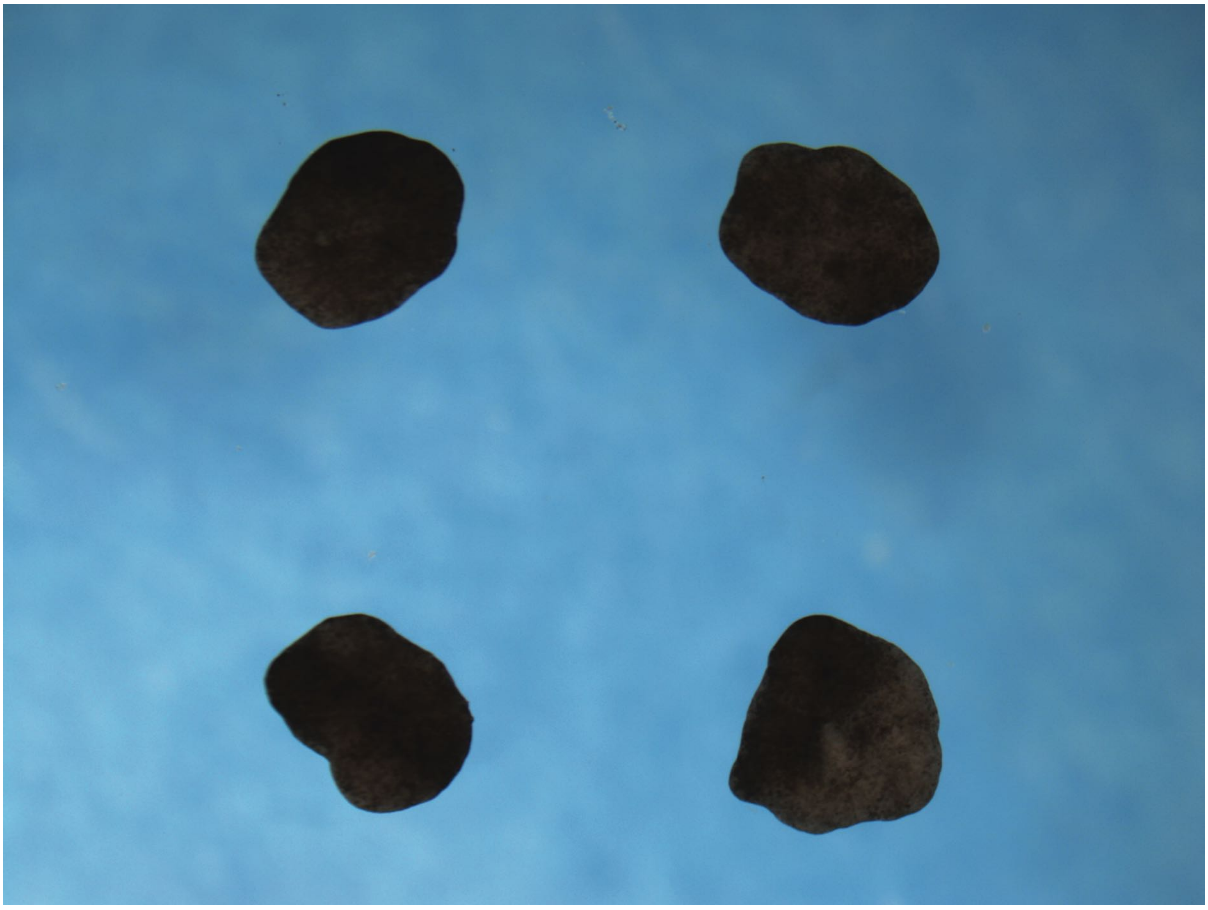

Raw image for Extended Data Fig. 13a stage 22 NANOG KD animal caps

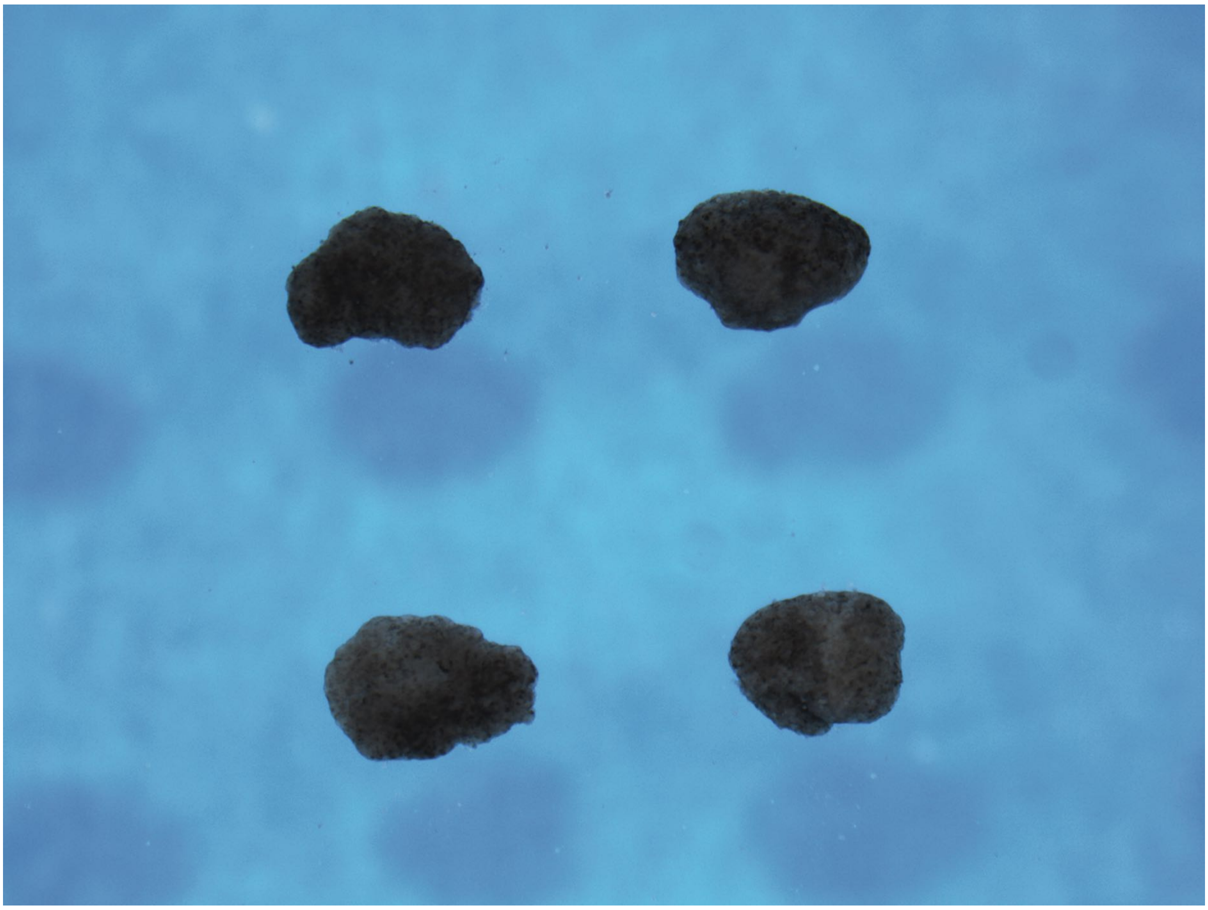

Raw image for Extended Data Fig. 13b stage 22 SB431542 treated animal caps

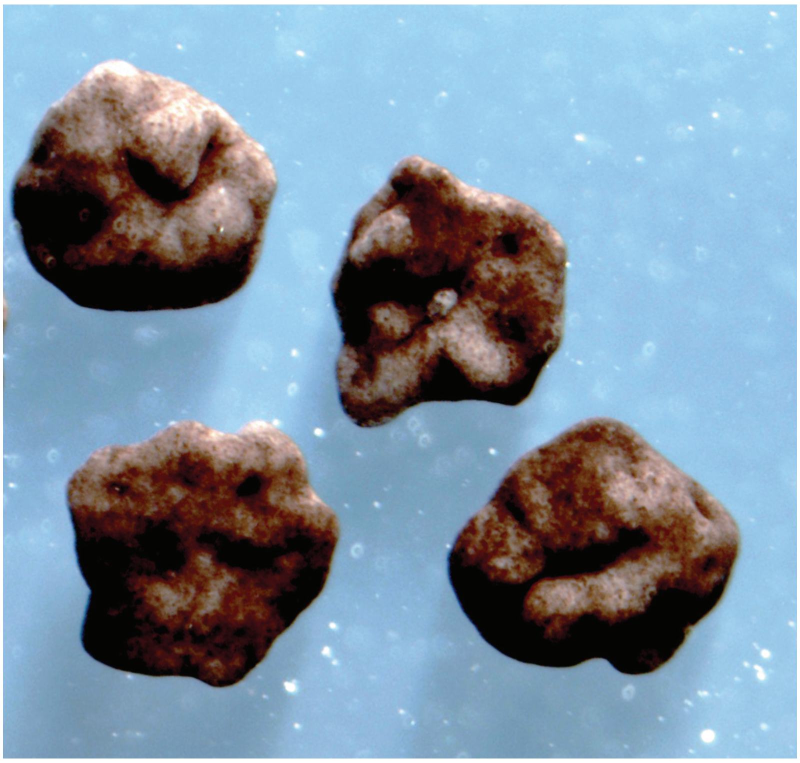

Raw image for Extended Data Fig. 13b stage 22 DPY30 KD animal caps

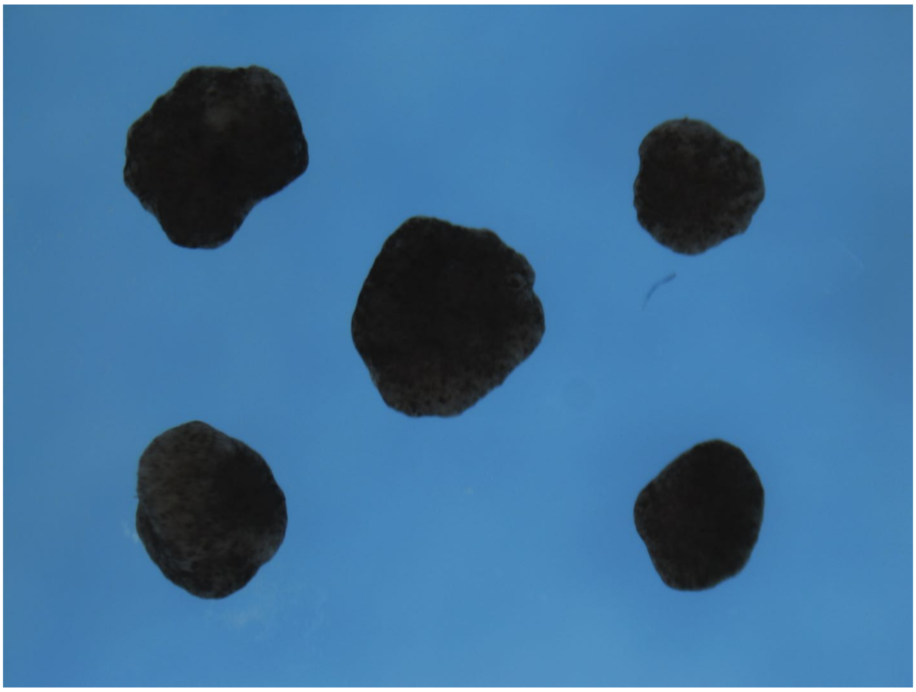

Raw image for Extended Data Fig. 13b stage 22 Uninjected animal caps

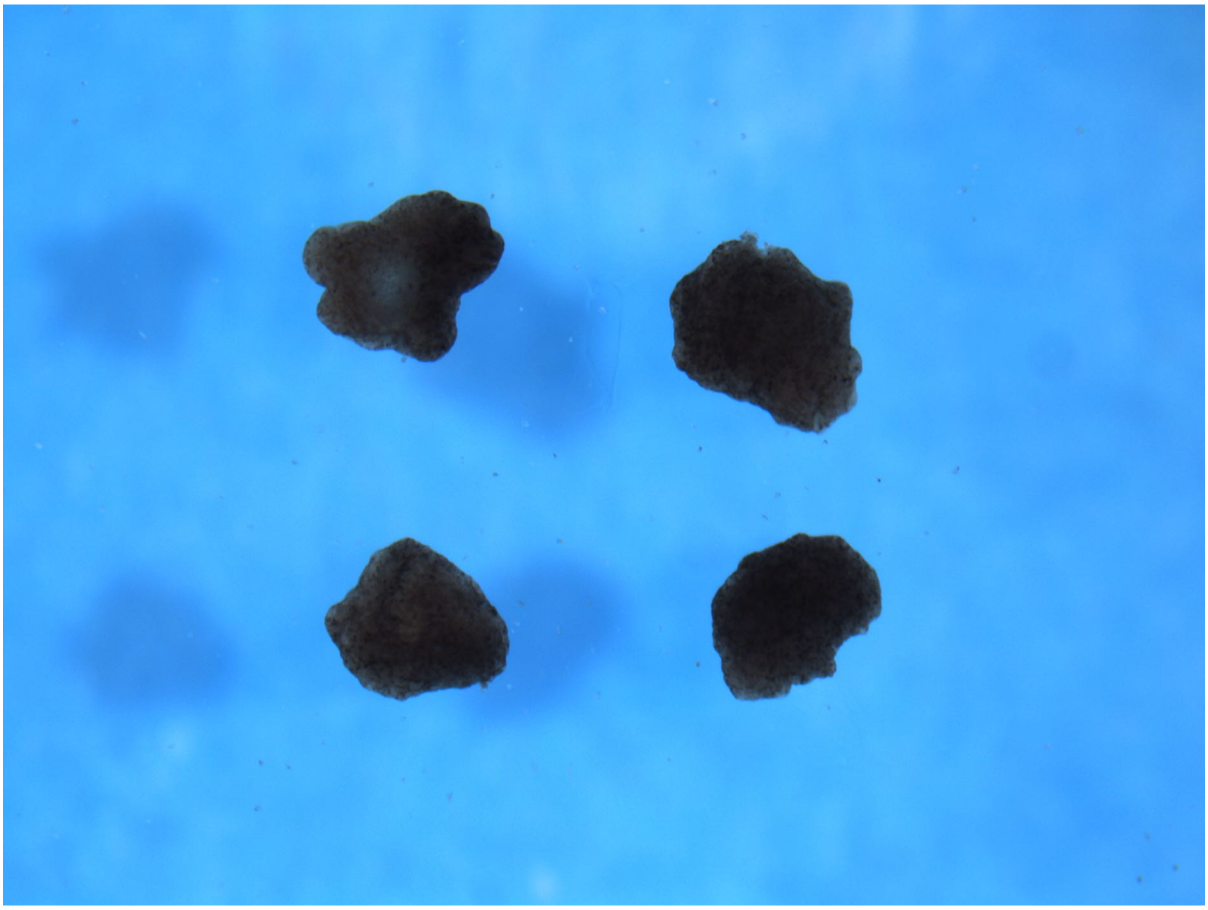

Raw image for Extended Data Fig. 13b stage 22 1pg activin animal caps

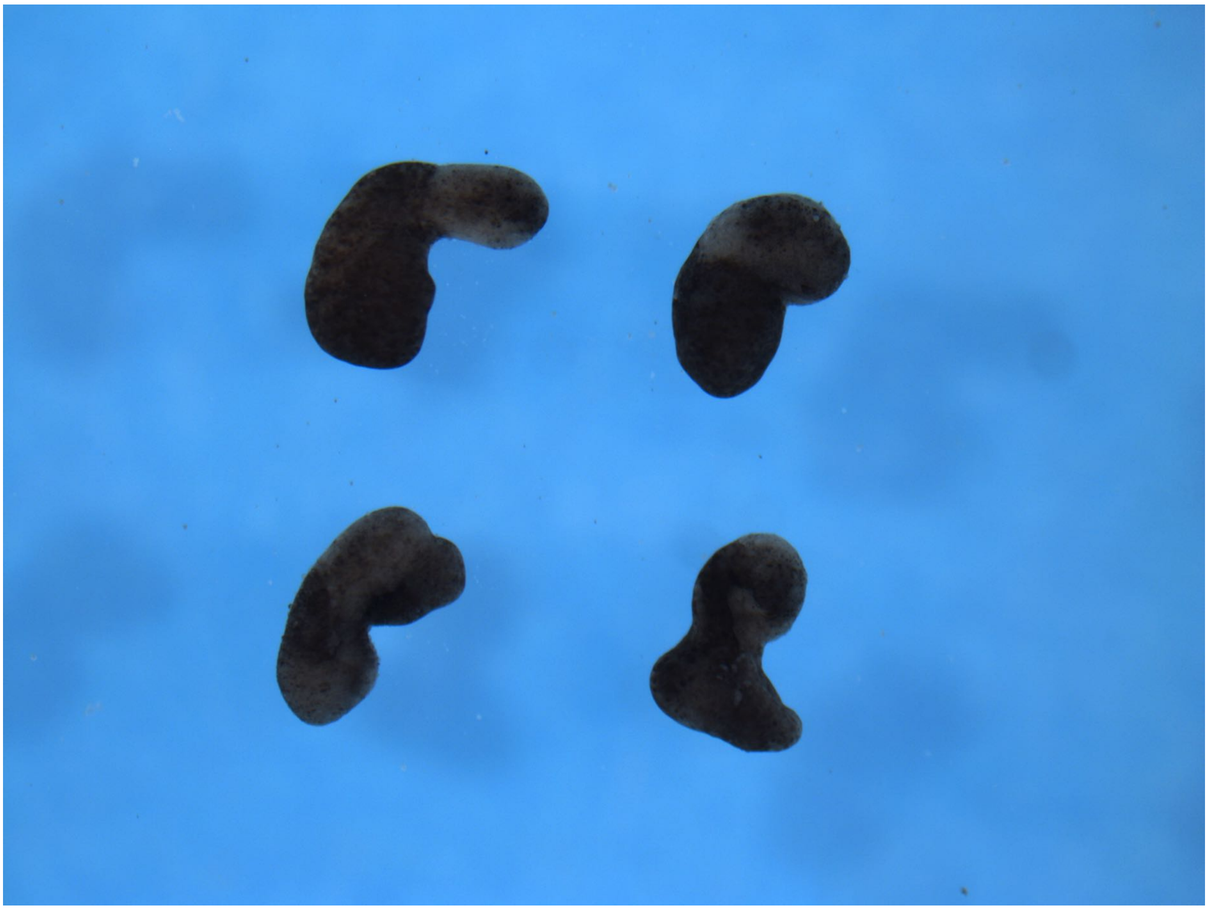

Raw image for Extended Data Fig. 13b stage 22 1pg activin NANOG KD animal caps

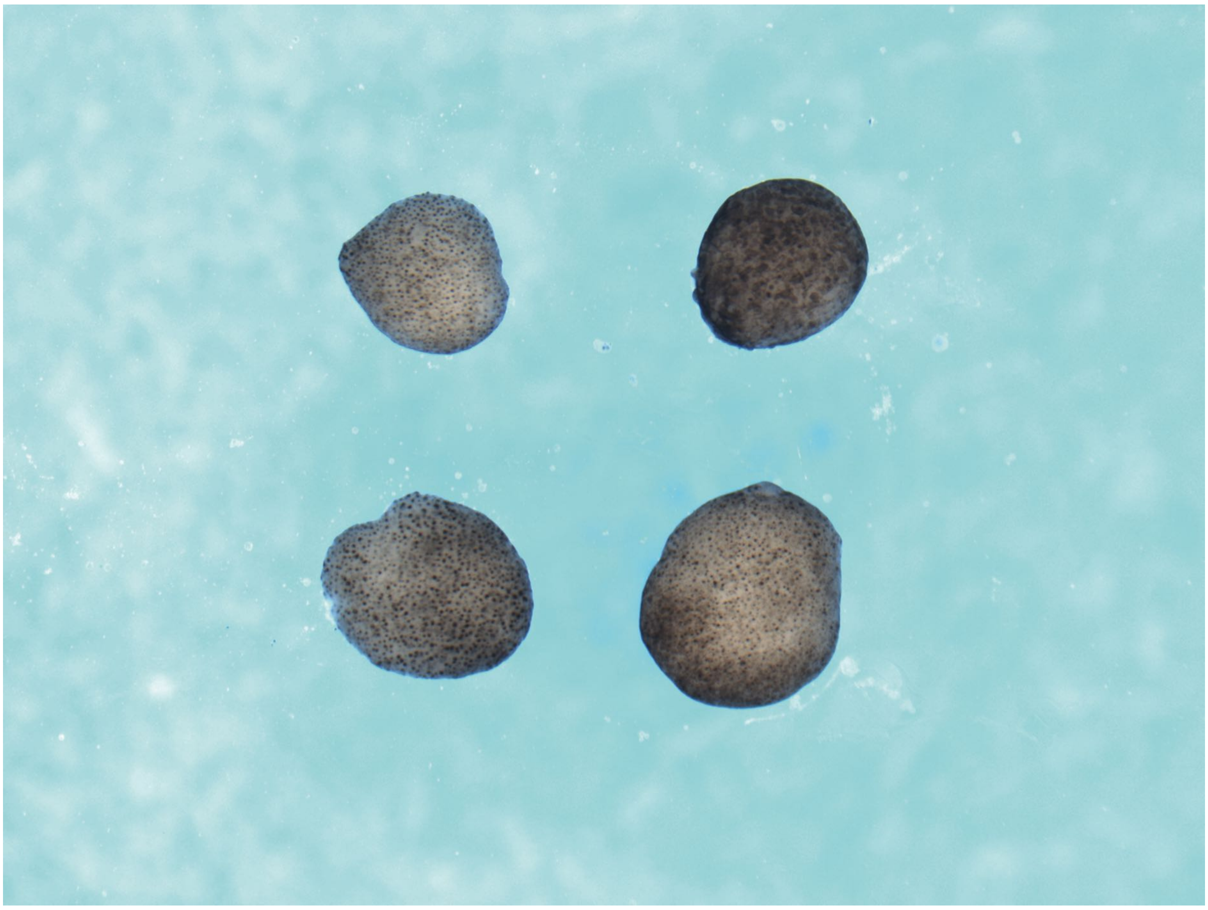

Raw image for Extended Data Fig. 13b stage 22 SB431542 animal caps

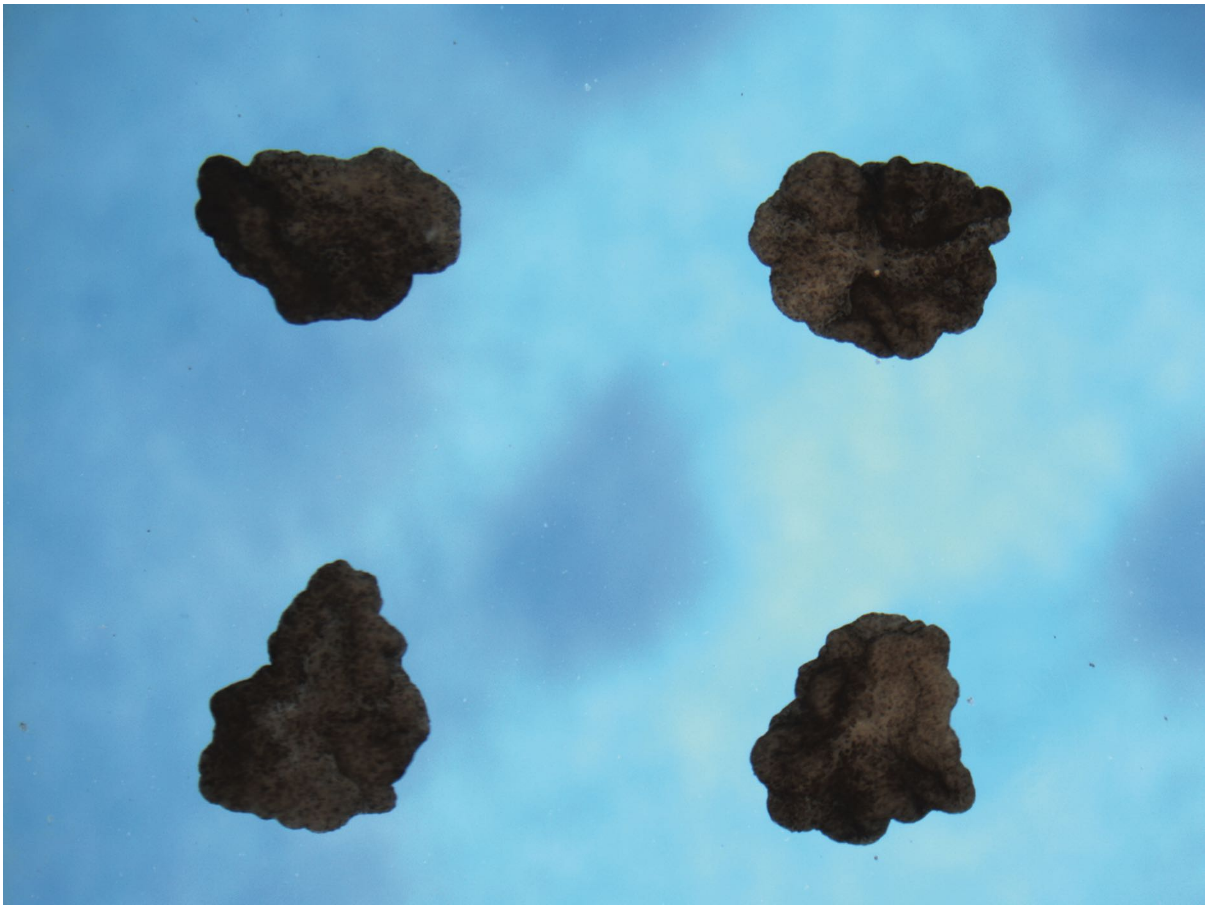

Raw image for Extended Data Fig. 13b stage 22 1pg activin DPY30 KD animal caps

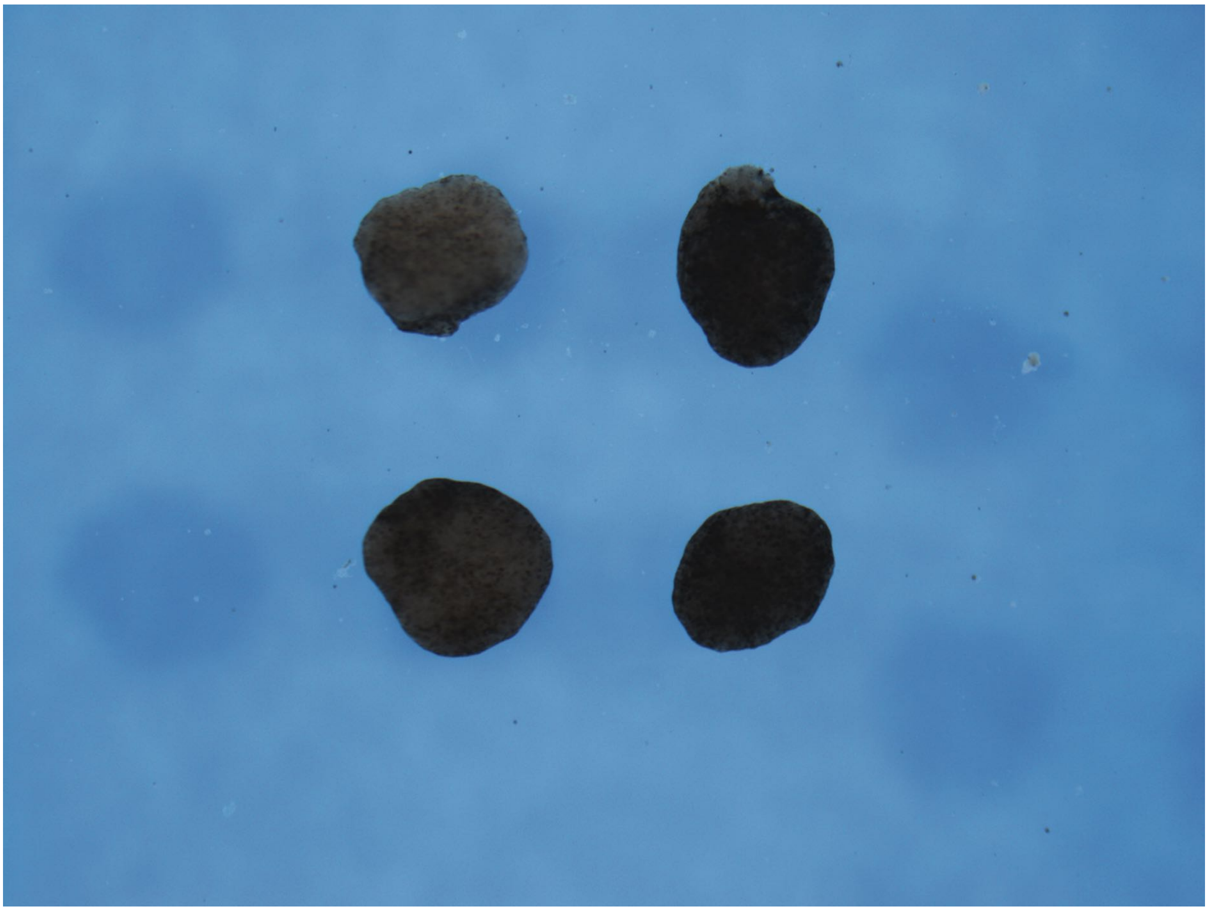

Supplement: S1 Images — (PDF) [file pbio.3002121.s023.pdf]
